# Precancerous pathways to gastric cancer: a review of experimental animal models recapitulating the correa cascade

**DOI:** 10.3389/fcell.2025.1620756

**Published:** 2025-07-02

**Authors:** Weiqing Li, Tai Zhang

**Affiliations:** ^1^ Institute of Burn Research, State Key Laboratory of Trauma, Burns and Combined Injury, Southwest Hospital, Third Military Medical University (Army Medical University), Chongqing, China; ^2^ Peking University Traditional Chinese Medicine Clinical Medical School (Xiyuan), Peking University Health Science Center, Beijing, China; ^3^ Institute of Digestive Diseases, Xiyuan Hospital, China Academy of Chinese Medical Sciences, Beijing, China

**Keywords:** gastric cancer, precancerous lesions, animal models, *Helicobacter* infection, genetically engineered mice, intestinal metaplasia, Correa cascade, Cre-loxP systems

## Abstract

Gastric cancer remains a significant global health challenge, representing the fifth most commonly diagnosed cancer and the fourth leading cause of cancer-related mortality worldwide. Understanding the pathogenesis of precancerous lesions is crucial for developing effective prevention and treatment strategies. This review provides a comprehensive analysis of animal models for gastric precancerous lesions, categorizing them into *Helicobacter* infection models, chemical carcinogen or diet-induced models, multifactorial induction models, chemical injury models, and genetically engineered mouse models. We evaluate the advantages and limitations of each model type, with particular focus on their ability to recapitulate the Correa cascade of human gastric carcinogenesis. While *Helicobacter* felis infection in C57BL/6 mice most closely mirrors the progression from chronic gastritis through metaplasia to dysplasia, these models primarily produce spasmolytic polypeptide-expressing metaplasia rather than true intestinal metaplasia, limiting translational relevance. Chemical carcinogen models reliably produce tumors but often bypass intermediate precancerous stages. Recent advances in genetic engineering, particularly stomach-specific inducible Cre recombinase systems targeting gastric progenitor cells, have yielded models that faithfully reproduce the spectrum of human gastric cancer subtypes with features of metastatic disease. We highlight the importance of standardized histopathological evaluation methodologies and discuss future research directions, including integration of advanced technologies such as single-cell RNA sequencing with existing animal models, development of organoid models, and investigation of interactions among genetic predisposition, *Helicobacter* infection, and environmental factors. This review provides a valuable reference for researchers investigating gastric precancerous lesions and offers insights for the development of more effective prevention and treatment strategies.

## 1 Introduction

Gastric cancer remains a major global health challenge, representing the fifth most commonly diagnosed cancer and the fourth leading cause of cancer-related mortality worldwide. In 2020, gastric cancer accounted for over 1.1 million new cases and approximately 769,000 deaths globally (Song et al., 2022). Despite declining age-standardized incidence rates in many regions over recent decades, the absolute burden continues to grow due to population aging and growth, posing substantial clinical and economic challenges (GBD, 2019 Asia and All Cancers Collaborators, 2024; Tan et al., 2024).

The geographical distribution of gastric cancer exhibits marked heterogeneity. The highest age-standardized incidence rates are observed in East Asia (particularly Mongolia, Japan, and South Korea), Eastern Europe, and parts of Latin America, while North America, Northern Europe, and Africa generally report lower rates (Song et al., 2022). In 2019, East Asia bore the greatest burden, accounting for approximately 53.8% of global cases and 48.2% of deaths, with China alone contributing 612,821 new cases and 421,539 deaths (GBD, 2019 Asia and All Cancers Collaborators, 2024). This geographical variation is largely attributed to differences in the prevalence of risk factors, particularly *Helicobacter pylori* (*Hp*) infection, which is responsible for approximately 89% of non-cardia gastric cancer cases globally.

Intestinal-type gastric adenocarcinoma represents the vast majority of gastric cancer cases and is linked to *Hp* infection (Tan and Yeoh, 2015). It follows the Correa cascade, progressing from chronic gastritis through a series of precursor lesions--atrophic gastritis, intestinal metaplasia, and dysplasia--before developing into invasive cancer (Morson et al., 1980). Gastric precancerous conditions refer to non-neoplastic conditions that have a risk of evolving into gastric cancer, while gastric precancerous lesions refer to pathological changes more likely to progress to invasive adenocarcinoma. The former includes atrophic gastritis, gastric ulcers, certain types of gastric polyps, and remnant stomach, with atrophic gastritis being the most prevalent. It is important to note that gastric polyps represent a heterogeneous group with varying malignant potential. Adenomatous polyps and hyperplastic polyps carry significant cancer risk, while fundic gland polyps are generally benign except in specific settings such as familial adenomatous polyposis (FAP) or other hereditary cancer syndromes where they may harbor dysplasia or progress to gastric cancer (Sano et al., 2021; Karpińska-Kaczmarczyk et al., 2016; Wang KK. et al., 2022). Inflammatory polyps typically have minimal malignant potential. The latter includes dysplasia and Type III intestinal metaplasia (Declich et al., 2013). Western researchers often group these conditions into the broader category of gastric precancerous lesions (Song et al., 2015). The degree of progression of gastric precancerous lesions is a significant risk factor for gastric cancer development. These precursor lesions represent key intervention points for prevention and early detection strategies. Recent systematic reviews and meta-analyses have provided important insights into the global prevalence and progression rates of these precancerous conditions. The worldwide prevalence of gastric intestinal metaplasia is estimated at 17.5%, with considerable variation across continents, ranging from 8.3% in Africa to 18.6% in the Americas (Soroorikia et al., 2024).

The progression rates of these precursor lesions to gastric cancer represent critical data for formulating surveillance strategies. Global pooled estimates indicate that the annual progression rate per 1,000 person-years is 2.09 (95% CI: 1.46–2.99) for atrophic gastritis, 2.89 (95% CI: 2.03–4.11) for intestinal metaplasia, and 10.09 (95% CI: 5.23–19.49) for dysplasia (Hahn et al., 2024). Interestingly, recent evidence suggests that progression rates of intestinal metaplasia and dysplasia to gastric cancer may be similar between low and medium/high incidence countries, challenging previous assumptions that progression rates would differ substantially across geographic regions (Hahn et al., 2024).

Although *Hp* eradication is the most important and controllable preventive measure for gastric cancer and serves as the primary prevention strategy, it does not completely eliminate the risk of gastric cancer (Rugge et al., 2017; Wu et al., 2019; Huang et al., 2023). The development of gastric cancer is a complex, multifactorial process. Understanding the natural history of gastric cancer precursor lesions is essential for developing effective prevention, screening, and management strategies (Zhang and Tang, 2025a). However, human studies face limitations in elucidating the molecular mechanisms driving progression. Animal models therefore play a crucial role in investigating the pathogenesis of these precursor lesions and testing potential interventions. By recapitulating the key steps of the Correa cascade, animal models provide insights into the complex interplay between genetic, environmental, and microbial factors in gastric carcinogenesis.

The establishment of simple and stable animal models for gastric epithelial transformation is essential for advancing our understanding of gastric carcinogenesis and for conducting intervention research on gastric precancerous lesions. In this context, the mouse model emerges as the most commonly utilized animal model for studying gastric precancerous lesions. Historically, early studies induced lesions using chemical carcinogens such as N-methyl-N′-nitro-N-nitrosoguanidine (MNNG), N-methyl-N-nitrosourea (MNU), and *Hp* infection. However, these traditional models often encounter significant challenges, including poor stability and extended development times. In light of recent advancements, the rapid progress in gene editing technologies has opened new avenues for the development of more effective gastric precancerous lesion mouse models. Therefore, this article aims to categorize and summarize the common animal models used for gastric precancerous lesions, analyze their respective advantages and disadvantages, and discuss key considerations in model evaluation. Before examining specific animal models, it is essential to establish the criteria and methodologies for evaluating these models’ ability to recapitulate human gastric carcinogenesis and their translational relevance.

## 2 Gastric precancerous lesion animal model evaluation

Given the diversity of animal models available for studying gastric precancerous lesions, establishing standardized evaluation criteria is crucial for meaningful comparison and translation to human disease. The following section outlines the key considerations for model assessment before detailed examination of specific model types.

### 2.1 Model evaluation methods

Most studies currently refer to the visual analogues of the New Sydney System--a widely used classification for histopathological diagnosis of gastritis in humans. However, it is important to recognize that the anatomical structure of rodent stomachs differs significantly from that of humans. For instance, while intestinal metaplasia characterized by goblet cells and Paneth cells is a common precancerous lesion observed in humans, the predominant metaplastic lesion in mice is spasmolytic polypeptide-expressing metaplasia (SPEM) rather than true intestinal metaplasia (Dixon et al., 1996). Furthermore, rodent models often exhibit unique pathological features, such as mucous metaplasia and eosinophilic droplets (also referred to as hyalinosis), which are not typical components of human gastric pathology (Ward et al., 2001; Lee et al., 2000). In light of the diversity of animal models used and the often arbitrary interpretation of histopathological findings, there is a pressing need for greater clarity and the adoption of standardized approaches in this research field to ensure more accurate and reliable assessments.

To address these limitations, it is therefore recommended to utilize the internationally recognized “Histologic Scoring of Gastritis and Gastric Cancer in Mouse Models” system (Rogers, 2012). This system utilizes histopathological assessment as the gold standard and integrates both clinical manifestations and macroscopic mucosal changes (Rogers, 2012). In addition, this method comprehensively evaluates active inflammation, chronic inflammation, atrophy, SPEM, intestinal metaplasia, and dysplasia or cancer through a semi-quantitative scoring approach, thereby offering a more nuanced and accurate model evaluation compared to human-based classification systems (Rogers, 2012).

It is also crucial to acknowledge that most studies currently define dysplasia or invasive cancer based on the presence of submucosal invasive glands, often termed gastric cystica profunda. Nonetheless, research has demonstrated that these submucosal invasive glands are, in fact, glands that have protruded from adjacent tissue and become embedded within the submucosal layer--a phenomenon known as pseudoinvasion. Alternatively, these structures could represent proliferating metaplastic SPEM glands, which lack the definitive pathological features of true dysplasia or cancer seen in human gastric neoplasia. Consequently, the degree of structural atypia in rodent glands serves as a more reliable indicator of dysplasia than does cellular atypia alone (Rogers, 2012). Therefore, only clear evidence of invasion beyond the muscularis mucosae and extension into the submucosal layer should be considered important markers of invasive cancer in these animal models.

### 2.2 Model success evaluation points

As previously mentioned, gastric precancerous lesions refer to pathological changes that are prone to transforming into cancer. Strictly speaking, dysplasia is a direct precancerous lesion, while atrophic gastritis and intestinal metaplasia are precancerous conditions in the human Correa cascade. Most studies use the occurrence of metaplastic lesions to indicate successful model establishment, but they do not report whether these lesions eventually progress to intestinal-type gastric cancer with invasion and distant metastasis as observed in human disease. If such cancer cannot develop, it suggests that the metaplastic tissue may not be a true precancerous lesion with malignant transformation potential but rather may have a reparative and protective function (Graham and Zou, 2018). Therefore, model evaluation should encompass the entire process from non-atrophic gastritis to tumor formation, with attention to different pathological stages that mirror human gastric carcinogenesis. The ability to generate dysplasia with tumor-like growth characteristics and intestinal-type adenocarcinoma with invasion or distant metastasis--features characteristic of advanced human gastric cancer--is a key issue in the establishment of clinically relevant animal models.

## 3 Overview of animal models for gastric precancerous lesions

### 3.1 Helicobacter infection models

*Hp* infection is a well-established independent risk factor for gastric cancer, with approximately 80% of cases attributable to this pathogen. In humans, *Hp* infection initiates the Correa cascade, progressing from chronic active gastritis through atrophic gastritis, intestinal metaplasia, and dysplasia to invasive adenocarcinoma. In 1998, Watanabe T and colleagues reported the first *Hp*-induced gastric cancer animal model. Following 62 weeks of chronic infection, 10 out of 27 Mongolian gerbils (37%) developed gastric tumors with histological features similar to human intestinal-type gastric cancer (Watanabe et al., 1998).

C57BL/6 mice exhibit significant resistance to various *Hp* strains. In 1990, researchers isolated *Helicobacter felis* (*Hf*), a species closely related to *Hp*, from feline gastric tissue. *Hf* has been shown to effectively colonize the mouse glandular stomach and induce more severe mucosal inflammation than *Hp*. Unlike human gastric pathology where *Hp* infection leads to intestinal metaplasia characterized by goblet cells expressing mucin 2 and Paneth cells, *Hf* infection in mice leads to the development of spasmolytic polypeptide-expressing metaplasia (SPEM), widespread dysplasia along the lesser curvature of the gastric corpus, and large polypoid lesions in the antrum. Eventually, invasive tumors can form in the gastric corpus (Lee et al., 1990; Wang et al., 1998; Fox et al., 2002).

However, these invasive lesions primarily manifest as submucosal cystic changes, classified as gastritis cystica profunda, which possesses limited malignant potential. Gastritis cystica profunda is a rare gastric lesion increasingly recognized as a possible precancerous condition. It is characterized by ectopic gastric glands within the submucosal layer of the stomach, often leading to cystic dilation and hyperplasia. Although the exact pathogenesis of gastritis cystica profunda remains unclear, it is frequently associated with prior gastric surgery or chronic inflammatory conditions such as bile reflux (Deng et al., 2019). Despite its generally benign nature, the malignant potential of gastritis cystica profunda remains a topic of debate, as it has occasionally been found in association with gastric adenocarcinoma (Kuwahara et al., 2013). Several studies have investigated the relationship between gastritis cystica profunda and gastric cancer. For example, one study reported an association between gastritis cystica profunda and aberrant p53 expression as well as Epstein-Barr virus infection in gastric cancer, suggesting a potential link to malignancy (Itami et al., 2021). Notably, this study found no cancerous lesions within the gastritis cystica profunda lesions themselves; however, a significant proportion of gastric cancers were located near or above areas of gastritis cystica profunda, indicating that gastritis cystica profunda may act as a paracancerous condition.

It is important to understand that gastric carcinoma encompasses several distinct histological subtypes, with adenocarcinoma being the predominant type, accounting for over 95% of gastric cancers. Gastric adenocarcinomas are further classified according to the Lauren classification into two main subtypes: intestinal-type and diffuse-type, each with distinct morphological features, epidemiological patterns, and molecular characteristics (Shin et al., 2019; Ma et al., 2016). The Correa cascade specifically describes the stepwise progression pathway leading to intestinal-type gastric adenocarcinoma, proceeding sequentially from normal gastric mucosa through chronic gastritis, atrophic gastritis, intestinal metaplasia, dysplasia, and finally to invasive adenocarcinoma. This well-established cascade does not apply to diffuse-type gastric adenocarcinoma, which typically arises through alternative pathways and may develop without the preceding intestinal metaplastic changes that characterize the Correa sequence. In contrast, human intestinal-type gastric adenocarcinoma shows true invasive behavior, including submucosal and lymphovascular invasion, with potential for lymph node and distant metastases (Kim et al., 2011; Olsen et al., 2017). Consequently, this model does not fully replicate the invasive or metastatic capabilities characteristic of intestinal-type adenocarcinoma. Furthermore, under *Hf* infection, C57BL/6 mice exhibit a heightened T-helper 1 (Th1)-mediated immune response, whereas Balb/c mice predominantly mount a T-helper 2 (Th2)-skewed response. This immune response pattern mirrors the Th1-predominant inflammatory response observed in human *Hp* gastritis, which is associated with increased gastric cancer risk (Shi et al., 2010). As a result, C57BL/6 mice demonstrate increased susceptibility to *Hf*-induced gastric mucosal injury, expressing higher levels of pro-inflammatory cytokines, such as interferon-γ(24). Long-term colonization of *Hf* in C57BL/6 mice exhibited significant gastric inflammation, with notable neutrophil and monocyte infiltration (Lee et al., 2014a). At 24 weeks, most infected mice developed gastric mucosal atrophy, metaplasia, and dysplasia, with adenocarcinoma observed in 1 mouse at 52 weeks. Pro-inflammatory cytokines, particularly interleukin-1β(34). In contrast, tumor necrosis factor-alpha did not show significant changes, indicating it may not contribute to the inflammatory processes in *Hf*-induced gastric pathology (Lee et al., 2014a).

Another study investigates the host-dependent nature of gastric pathology induced by *Hf* in a mouse model. In this study, six inbred mouse strains were infected with *Hf*, and the resulting gastric changes were assessed at one, two, and 6 months post-infection (Sakagami et al., 1996). The results revealed significant variation in responses across different strains. This strain-dependent variability reflects the genetic diversity observed in human populations, where host genetic factors influence susceptibility to *Hp*-induced gastric pathology and cancer development. Notably, strains such as SJL, C3H/He, DBA/2, and C57BL/6 developed moderate to severe chronic gastritis in the body of the stomach, which eventually progressed to atrophic changes (Sakagami et al., 1996). In contrast, strains like BALB/c and CBA exhibited minimal gastritis and no atrophic changes (Sakagami et al., 1996).

Importantly, atrophy was typically observed only in the body of the stomach, while bacterial colonization was more pronounced in the antrum (Sakagami et al., 1996). As corpus damage progressed over time, *Hf* density in the antrum declined, suggesting that corpus atrophy does not require the local presence of bacteria. This pattern parallels human *Hp* gastritis, where pangastritis (involving both antrum and corpus) is associated with higher gastric cancer risk compared to antral-predominant gastritis (Naylor et al., 2006). These findings suggest that the development of gastric atrophy is not directly related to the presence of *Hf* in the antrum, but rather may be influenced by host-specific factors, possibly including an autoimmune response. This classic observation--corpus atrophy occurring alongside predominant antral colonization--has been consistently replicated in subsequent murine models. For instance, long-term *Hf* infection in C57BL/6 mice reliably induces corpus lesions, including parietal cell loss and mucosal thinning, within 6–24 weeks (Lee et al., 2014a). Similarly, Burkitt MD et al. (Burkitt et al., 2013; Duckworth et al., 2012) report that in their *Hf* mouse model, the gastric antrum is reliably colonized and pathology develops in the gastric corpus 6 weeks after infection. Taken together, multiple murine studies have demonstrated that *Hf*-induced atrophy predominantly affects the corpus, even as bacterial load is concentrated in the antrum.

Host-specific factors appear to play a critical role in mediating this regionally distinct pathology. As shown by Sakagami et al. (1996), different mouse strains exhibit markedly different responses to *Hf* infection. Some strains mount a robust inflammatory response in the corpus, resulting in atrophy, while others show minimal damage under identical conditions. Immune regulatory pathways also significantly influence these outcomes. For example, IL-10 knockout mice develop rapid and severe corpus atrophy upon *Hf* infection; by 4 weeks, they exhibit near-complete loss of parietal and chief cells, accompanied by hyperplastic gastritis and metaplasia (Berg et al., 1998). In contrast, wild-type mice under the same conditions display only focal gastritis (Berg et al., 1998).

Interestingly, corpus atrophy can also occur in the absence of bacterial infection. In a neonatal thymectomy model of autoimmune gastritis, BALB/c mice developed loss of corpus glands and anti-parietal cell antibodies without any exposure to *Hp*. Paradoxically, introducing *Hp* into this autoimmune model reduced both corpus atrophy and autoantibody levels (Ohana et al., 2003). This counterintuitive finding highlights the importance of the immune context in shaping gastric pathology.

Recent murine studies reaffirm the classical observation that *Helicobacter*-induced atrophy localizes primarily to the stomach corpus, even when bacterial colonization is more intense in the antrum (Johnson et al., 2023). Notably, the severity and location of atrophy do not correlate directly with antral bacterial burden. Instead, they are largely determined by host-specific factors—including genetic background, cytokine expression profiles, and autoimmune predispositions. For instance, enhanced Th1- or IL-1β–mediated inflammation, as seen in *H. felis* infection or IL-10 deficiency, promotes corpus injury, whereas Th2-skewed or regulatory immune environments may confer relative protection.

To highlight further differences, a recent study provides a direct and systematic comparison of the gastric pathology induced by *Hp* and *Hf* in a murine model. Despite both bacteria similarly activating macrophages *in vitro*, their *in vivo* effects diverge significantly (Druffner et al., 2024). Specifically, 2 months post-infection, *Hp* induces only mild inflammation and limited epithelial changes in the gastric corpus, whereas *Hf* triggers extensive immune cell infiltration, severe atrophic gastritis, and widespread pyloric metaplasia (Druffner et al., 2024). This includes the loss of chief and parietal cells, as well as the upregulation of metaplastic markers such as Cluster of Differentiation 44 variant (CD44v)-9 (42). The loss of specialized gastric epithelial cells (chief and parietal cells) in *Hf*-infected mice recapitulates the atrophic changes observed in human corpus-predominant gastritis, which is strongly associated with gastric cancer development (Cheng et al., 2017; Motta et al., 2008). Thus, these findings highlight that *Hp*, being a poor immunogen in mice, may be more suited for studies focused on colonization and microbiota interactions, while *Hf* better models inflammation-driven gastric remodeling and early neoplastic events. Additionally, another study reports that after 10 months of infection, the gastric corpus of the mice exhibited cells with the morphology characteristic of intestinal metaplasia, identified through H&E and alcian blue-periodic acid-Schiff staining (Chen et al., 2019). However, this murine “intestinal metaplasia” differs from human intestinal metaplasia, which is characterized by the presence of goblet cells expressing mucin 2 and absorptive cells expressing villin. The intestinal metaplasia observed in the *Hf*-infected mouse model shares key features of precancerous tissue and demonstrates a clonal nature of genetic instability within the intestinal metaplasia glands (Chen et al., 2019). This underscores its potential as a valuable model for investigating the transformation of metaplastic cells to malignant cells in gastric carcinogenesis.

In 1997, Lee A and colleagues isolated the *Hp* SS1 strain (Sydney strain), which harbors both cytotoxin-associated gene A (CagA) and vacuolating cytotoxin A (VacA), from the gastric mucosa of a patient with a duodenal bulb ulcer. These virulence factors are clinically relevant as CagA-positive Hp strains are associated with increased gastric cancer risk in humans (Gwack et al., 2006; Chuang et al., 2011). This strain effectively colonizes the glandular stomach of mice, achieving high colonization levels in C57BL/6 mice, while colonization remains comparatively low in Balb/c, DBA/2, and C3H/HeJ strains (Lee et al., 1997). Although SS1 infection induces chronic active gastritis and atrophic changes after 8 months, it fails to induce gastric cancer in wild-type C57BL/6 mice even after 2 years of infection (Lee et al., 1997). This limitation contrasts with human disease, where chronic *Hp* infection can progress to gastric cancer over decades. However, in C57BL/129 mice, *in situ* carcinoma has been observed after 15 months of SS1 infection (Lee et al., 1997).

Notably, the metaplastic changes resulting from *Helicobacter* infection in murine models are limited to SPEM, which differs fundamentally from human intestinal metaplasia There is no evidence of intestinal metaplasia, as indicated by the absence of mucin 2 and trefoil factor 3 labeling in cells characteristic of intestinal goblet cell-like morphology and villin-expressing absorptive cells. In humans, true intestinal metaplasia is characterized by the replacement of gastric epithelium with intestinal-type epithelium containing goblet cells, Paneth cells, and absorptive enterocytes.

Research has shown that *Hp* eradication can improve or even reverse atrophic gastritis. However, in humans, intestinal metaplasia is considered an irreversible stage, beyond which *Hp* eradication offers no histological reversal or reduction in gastric cancer risk (Wang et al., 2011). In studies using *Hf*-infected C57BL/6 mice, eradication therapy administered at either 2 months (early) or 6 months (advanced) post-infection facilitated the resolution of mucosal inflammation, restoration of parietal cell populations, and reconstruction of glandular architecture. Furthermore, eradication at 1 year post-infection was able to halt progression to dysplasia (Cai et al., 2005). This reversibility of metaplastic changes in mice contrasts with the irreversible nature of intestinal metaplasia in humans, highlighting a key limitation of murine models for studying advanced precancerous lesions.

In p27-deficient (p27^−/−^) mice, eradication therapy administered during both early (15 weeks) and late (45 weeks) stages of infection significantly reduced lesion progression, even in the presence of established SPEM. These findings collectively suggest that Hp eradication remains beneficial in reducing the risk of gastric cancer, even when administered at advanced pathological stages of gastric precancerous lesions (Zhang et al., 2014). While this finding supports the clinical practice of *Hp* eradication, the reversibility observed in mouse SPEM may not accurately reflect the persistence of intestinal metaplasia in humans following eradication therapy (Wang et al., 2011; Satoh et al., 1998). The summary of *Helicobacter* infection models for gastric precancerous lesions is shown in Supplementary Table 1.

### 3.2 Chemical carcinogen or diet induced models

Before *Hp* infection was definitively established as a cause of gastric cancer, researchers in the 1930s explored the effects of various chemical carcinogens, including benzo [a]pyrene, 3-methylcholanthrene, and 2-acetylaminofluorene. These early studies aimed to replicate the environmental carcinogen exposure that was suspected to contribute to the high gastric cancer rates observed in certain human populations, particularly in regions with dietary exposure to nitrosamines and polycyclic aromatic hydrocarbons (Correa et al., 1979; Wu et al., 2022; Picetti et al., 2022). However, they found that the incidence of gastric cancer induced by these chemicals was relatively low. It was not until 1967 that Saito et al. (1970) successfully demonstrated that MNNG could efficiently induce gastric adenocarcinoma in rats. Further studies revealed that MNNG primarily induced squamous cell carcinoma in the forestomach of rats, with only gastric adenomas observed (Saito et al., 1970; Abe et al., 2003; Schoental, 1966). The forestomach, which is present in rodents but absent in humans, represents a major anatomical difference that complicates translation of findings to human gastric cancer. When Mongolian gerbils were given 400 ppm MNNG in drinking water for 50 weeks, the incidence of gastric adenocarcinoma reached 64% (Tatematsu et al., 1998). However, due to the lack of stable genetic strains in rats and gerbils, their use was limited, prompting subsequent studies on MNNG in inbred mice, where it was found that the glandular stomach in mice exhibited resistance to MNNG.

Subsequently, MNU was used to induce gastric adenocarcinoma in mice and was found to be more effective than MNNG. However, administering 0.5 mg of MNU weekly via gavage to Balb/c mice resulted in the development of squamous cell carcinoma of the forestomach, leading to premature death. When the forestomach was surgically removed before MNU treatment, the incidence of gastric adenocarcinoma in these mice reached 100% by 40 weeks (Tatematsu et al., 1992). Therefore, although the mouse glandular stomach is more sensitive to MNU, the greater sensitivity of the forestomach to MNU, which causes squamous cell carcinoma-related mortality, hindered further observation of gastric adenocarcinoma.

Through repeated experimentation, researchers found that low doses of MNU (30–120 ppm) in drinking water could effectively induce gastric adenocarcinoma without causing forestomach tumors. They demonstrated that the induction efficiency of gastric adenocarcinoma was dependent on the concentration of MNU rather than the total dose. Ultimately, a standardized protocol for inducing gastric adenocarcinoma in mice was developed, involving free access to 240 ppm MNU in drinking water every 2 weeks for five consecutive weeks (Tatematsu et al., 1992; Yamachika et al., 1998; Li K. et al., 2021).

Tumors induced by MNU and MNNG predominantly occur in the gastric antrum and mainly consist of well-differentiated to moderately differentiated adenocarcinomas with a significant stromal cell component, resembling human intestinal-type adenocarcinomas (Yamamoto et al., 2002). However, a critical limitation is that unlike the human Correa cascade, which is characterized by chronic *Hp* infection progressing through chronic gastritis, atrophic gastritis, intestinal metaplasia, and dysplasia before developing into invasive cancer (Correa et al., 1975), gastric cancer induced by MNNG and MNU does not follow the classical sequence of atrophy-metaplasia-dysplasia. Instead, these chemical carcinogens typically induce direct neoplastic transformation, bypassing the precancerous stages that are clinically relevant in human gastric cancer development. Some researchers suggest that the low-acid or achlorhydric environment caused by chronic *Hp* infection facilitates bacterial overgrowth in the stomach, which further metabolizes ingested nitrates and nitrites, ultimately producing N-nitroso compounds. This mechanism is clinically relevant as epidemiological studies have shown that dietary nitrates and nitrites, particularly in preserved and processed foods, are associated with increased gastric cancer risk in humans (Liu S-J. et al., 2022; Lin et al., 2014). Therefore, the carcinogenic pathways associated with MNNG and MNU may, to some extent, participate in the *Hp*-associated gastric epithelial transformation process. The N-nitroso compounds mouse model of gastric cancer has been utilized to investigate various signaling pathways in gastric carcinogenesis, including the roles of p53 (Yamamoto et al., 2000), nuclear factor-kappa B (Sakamoto et al., 2010), the mitogen-activated protein kinase pathway (Shibata et al., 2008; Hayakawa et al., 2011), cyclooxygenase-2 (Takasu et al., 2008), β-catenin (Takasu et al., 2008), E-cadherin (Humar et al., 2009), and Kruppel-like factor 4 (Li et al., 2012); however, it is not considered a typical precancerous model. While these molecular pathways are indeed dysregulated in human gastric cancer, the rapid, direct carcinogenic effect of MNU limits its utility for studying the gradual progression of precancerous lesions observed in human disease. The MNU-based chemical carcinogen model has several shortcomings, including uncertainty in the timing, location, number, and size of tumor formation, as well as significant genetic heterogeneity of the tumors.

High salt and pickled diets are significant risk factors for the development of gastric cancer, with epidemiological studies showing strong associations between high dietary sodium intake and gastric cancer incidence, particularly in East Asian populations where traditional diets include high levels of salted and pickled foods (Yu et al., 2025; Avila-Nava et al., 2025; Yu W. et al., 2024; Wang et al., 2009). Elevated concentrations of sodium chloride reduce the viscosity of gastric mucin, thereby compromising the gastric mucosal barrier (Gaddy et al., 2013). This mechanism is thought to contribute to gastric cancer development in humans by enhancing susceptibility to *Hp* colonization and increasing mucosal damage. Long-term high-salt diets induce atrophic gastritis in Mongolian gerbils and C57BL/6 mice; however, they do not lead to tumor formation (Bergin et al., 2003; Fox et al., 1999; Kodama et al., 1984). In contrast, when sodium chloride is co-administered with MNNG or nitroquinoline-1-oxide, it promotes the development of gastric cancer in rats in a dose-dependent manner (Tatematsu et al., 1975; Takahashi et al., 1983; Takahashi et al., 1994). This synergistic effect mirrors epidemiological observations in humans, where populations with both high salt intake and exposure to N-nitroso compounds show elevated gastric cancer rates. A high-salt diet combined with *Hp* strain SS1 infection results in more pronounced atrophy and epithelial hyperplasia in C57BL/6 mice. Additionally, high-salt diets increase gastric tumor formation in mice treated with MNU (Fox et al., 1999; Kodama et al., 1984). Therefore, while high salt intake alone does not induce gastric cancer, it acts synergistically with other factors to elevate the risk of gastric cancer development.

In B6129 mice, high-salt ingestion increased bacterial colonization and altered the immune response, shifting from a Th1 to a Th2 pattern; however, it did not enhance the progression of gastric cancer. Mice infected with *Hp* developed chronic gastritis, intestinal metaplasia, and dysplasia by 15 months, consistent with the development of gastric intraepithelial neoplasia (Rogers et al., 2005). Nevertheless, the high-salt diet did not promote or accelerate the onset of gastric cancer (Rogers et al., 2005). These findings contrast with human epidemiological data suggesting a synergistic effect between high salt intake and *Hp* infection in gastric cancer development, highlighting potential species-specific differences in dietary carcinogenesis (Yamaguchi and Kakizoe, 2001). These findings suggest that while *Hp* infection is a key driver of gastric neoplasia, high-salt intake alone does not synergistically increase the risk of gastric cancer in this mouse model.

A subsequent study investigated the effects of *Hp* colonization and the inflammatory response in C57BL/6 mice fed a high-salt diet (Lee et al., 2014b). The mice were inoculated multiple times with *Hp* strain SS1 to evaluate the infection rate and subsequent inflammation (Lee et al., 2014b). Results indicated that the high-salt diet significantly increased bacterial colonization but did not correlate with higher levels of inflammation, as measured by neutrophil and mononuclear cell infiltration (Lee et al., 2014b). Despite the enhanced colonization in the high-salt diet group, the degree of gastric inflammation remained similar to that of mice on a basal diet (Lee et al., 2014b). This study suggests that while high-salt intake can increase *Hp* colonization in mice, it does not exacerbate the inflammatory response or influence the severity of infection-associated gastritis, challenging previous hypotheses regarding a synergistic effect between salt intake and *Hp*-induced gastric inflammation. This discrepancy with human disease patterns underscores the limitations of rodent models in fully recapitulating the complex dietary and environmental interactions that contribute to human gastric carcinogenesis. Supplementary Table 2 presents a summary of animal models for gastric precancerous lesions induced by chemical carcinogens or dietary interventions.

### 3.3 Helicobacter infection, chemical carcinogens, diet, and other multifactorial composite induction models

Single-factor modeling methods are limited in scope, with long modeling durations, and do not fully align with the understanding that atrophic gastritis and its precancerous lesions are complex diseases involving multiple factors. This multifactorial nature reflects human gastric carcinogenesis, where the interaction of *Hp* infection, dietary factors (such as high salt intake and N-nitroso compounds), genetic susceptibility, and environmental exposures collectively contribute to cancer development through the Correa cascade. As a result, researchers have attempted to combine additional carcinogenic factors with MNU and MNNG for composite induction to better simulate and approximate the human pathogenic process.

Takahashi et al. (1994) used different concentrations of NaCl solution (10%, 5%, 2.5%, and 0%) in combination with 100 ppm MNNG to treat rats. They found that 10% and 5% NaCl significantly increased the incidence of adenocarcinomas and adenomas induced by MNNG (84). Although 2.5% NaCl also showed a trend toward increased tumor formation, it did not reach statistical significance (Takahashi et al., 1994). This dose-dependent relationship mirrors epidemiological observations in human populations, where regions with extremely high dietary salt intake (such as parts of Japan and Korea) show correspondingly elevated gastric cancer rates. The lesions induced by the MNNG-NaCl combination were primarily located in the pyloric and cardia regions of the stomach, including adenomas and adenocarcinomas, with adenocarcinomas invading deeper layers of the gastric wall (Takahashi et al., 1994). However, no metastasis was observed (Takahashi et al., 1994). The absence of metastasis represents a limitation compared to human gastric cancer, where intestinal-type adenocarcinoma frequently metastasizes to lymph nodes, liver, and peritoneum.

Yin et al. (2021) administered 200 mg/kg MNNG via gavage to each rat on days 0 and 14. Following this, MNNG was administered at a dose of 600 μg/kg every other day (Yin et al., 2021). A saturated NaCl solution was concurrently administered, with 1 mL given three times a week during the first 3 weeks (Yin et al., 2021). From week 5 onward, MNNG and NaCl were alternated every other day, with 1 mL per dose (Yin et al., 2021). After 25 weeks, mild gastric atrophy was observed, and intestinal metaplasia was successfully induced by week 35 (Yin et al., 2021). This timeline is considerably accelerated compared to human disease, where the progression from chronic gastritis to intestinal metaplasia typically occurs over decades of chronic *Hp* infection.

Nakamura et al. (2002) administered 200 ppm MNU to mice on a weekly alternating schedule for 5 weeks, in addition to infecting them with the *Hp* SS1 strain via oral inoculation three times per week. The results showed that the incidence of dysplastic lesions and adenomatous hyperplasia was lower in the MNU plus *Hp*-infected mice compared to those treated with MNU alone (Nakamura et al., 2002). This suggests that *Hp* infection did not enhance the carcinogenicity of MNU but instead exhibited an inhibitory effect (Nakamura et al., 2002). This finding contrasts with human epidemiological data, where *Hp* infection is a well-established promoter of gastric cancer development, suggesting that the interaction between chemical carcinogens and bacterial infection may differ between mice and humans.

Toyoda et al. (2013) administered 120 ppm MNU to mice through free drinking water on an alternating weekly basis, along with a 10% NaCl high-salt diet for 5 weeks. *Hp* infection was introduced via intragastric inoculation every other week, for a total of 7 infections (Toyoda et al., 2013). By week 40, 100% of the mice developed gastric antral tumors, with 88.8% of them being adenocarcinomas, and an average of 2.6 tumors per mouse (Toyoda et al., 2013). In contrast, only 61.9% of the mice treated with MNU alone developed tumors, primarily adenocarcinomas, with an average of 0.9 tumors per mouse (Toyoda et al., 2013). Among the MNU plus 10% NaCl high-salt diet group, 50% of the mice developed gastric tumors, with 42.9% being adenocarcinomas, and an average of 1.0 tumor per mouse (Toyoda et al., 2013). In the MNU plus *Hp*-treated group, 100% of the mice developed adenomas or adenocarcinomas, with an average of 3.3 tumors per mouse (Toyoda et al., 2013). This synergistic effect between high salt intake and *Hp* infection aligns with epidemiological evidence from human populations, particularly in East Asia, where both dietary factors and high *Hp* prevalence contribute to elevated gastric cancer incidence. This study suggests that the synergistic effects of *Hp* infection and/or a high-salt diet significantly increased the tumorigenicity of MNU. However, there is some debate over whether *Hp* infection promotes gastric cancer development in the MNU model and whether a high-salt diet accelerates the progression of gastric lesions induced by *Hp*.

Researchers have investigated the combined effects of N-nitroso compounds in drinking water, high-salt diets, and *Hp* infection, alongside additional stimuli such as food deprivation and refeeding, ethanol gavage, ranitidine hydrochloride, sodium deoxycholate, and sodium salicylate, to model the mechanisms underlying human gastric precancerous lesions. These complex protocols attempt to replicate the multifactorial exposures that characterize human gastric carcinogenesis, including dietary carcinogens, acid suppression, bile reflux, and inflammatory stimuli that are observed in clinical settings. For instance, some studies utilized MNNG at a concentration of 100 μg/mL in drinking water, ranitidine hydrochloride at 0.05% for free feeding, sodium salicylate at 5 mL/kg via gavage, and employed a food deprivation/refeeding regimen to simulate atrophic gastritis, gastric precancerous lesions, and gastric cancer in rats over several weeks (Li et al., 2022; Chu et al., 2021). However, these methodologies are primarily employed by a limited number of researchers in the fields of herbal medicine and complementary and alternative medicine (Zeng et al., 2018; Xie et al., 2024; Liao et al., 2023). They encounter limitations due to the absence of standardized protocols, which result in varying combinations and concentrations of N-nitroso compounds. This lack of standardization contributes to a dearth of corroborating studies and introduces additional variables that may compromise the validity of the findings (Zhang and Tang, 2024).

Importantly, the intestinal metaplasia described in these studies is characterized by Alcian blue pH 2.5 positive lesions in the antrum, without the induction of goblet cell-containing intestinal metaplasia that characterizes human disease. It is crucial to recognize that Alcian blue staining indicates the presence of specific classes of sugar residues on mucins, which can vary significantly among species. In humans, true intestinal metaplasia is defined by the presence of goblet cells expressing MUC2, absorptive enterocytes expressing villin, and sometimes Paneth cells, which are not typically observed in rodent models. Notably, deep glandular cells in the murine antrum are typically Alcian blue positive. While Alcian blue-positive mucins are absent in the normal corpus, they are present in the basal cells of normal antral glands as well as in intestinal goblet cells.

Recent research has examined the effects of chronic cigarette smoke exposure on *Hp* infection and gastric cancer initiation in mice. This approach is clinically relevant as cigarette smoking is an established risk factor for gastric cancer in humans, with meta-analyses showing increased risk particularly for cardiac and non-cardiac gastric cancers (Jayalekshmi et al., 2015; Rota et al., 2024). C57BL/6 mice were exposed to cigarette smoke after being infected with *Hp*, resulting in significant changes in gastric pathology (Morris et al., 2025). While cigarette smoke exposure did not affect *Hp* colonization, it suppressed the typical inflammatory response associated with infection, leading to reduced gastric atrophy and pyloric metaplasia (Morris et al., 2025). However, cigarette smoke exposure resulted in increased DNA damage and accelerated the onset of dysplasia, a precancerous condition (Morris et al., 2025). This paradoxical finding—where smoking reduces inflammation but accelerates dysplasia—may reflect the complex dual effects of smoking observed in human studies, where smoking both impairs immune responses and directly induces DNA damage. These findings suggest that smoking obscures some classic pathological features of *Hp* infection while promoting mutations and accelerating the initiation of gastric cancer. This mechanism is consistent with human studies showing that smoking increases gastric cancer risk through direct mutagenic effects, even in the presence of reduced inflammatory responses (Hatta et al., 2024; Kinoshita et al., 2025). Supplementary Table 2 presents a summary of animal models for gastric precancerous lesions induced by multifactorial composite interventions.

### 3.4 Acute chemical injury induced models

Recent studies suggest that SPEM may play a critical role in gastric tumorigenesis, potentially serving as the origin of dysplasia or cancer. In humans, SPEM has been identified in gastric biopsies from patients with chronic atrophic gastritis and is considered an early metaplastic change that may precede intestinal metaplasia in the Correa cascade, though its exact role as a precancerous lesion remains debated (Dilaghi et al., 2021). Consequently, researchers have investigated acute drug-induced SPEM models to gain a deeper understanding of the mechanisms underlying the development and progression of SPEM.

Acute drug models include DMP-777, L635, and high-dose tamoxifen. These models attempt to replicate the parietal cell loss that occurs in human chronic atrophic gastritis, a key step in the Correa cascade that leads to achlorhydria and subsequent metaplastic changes (Weis and Goldenring, 2009). DMP-777 is a proton carrier for the parietal cell membrane that inhibits the accumulation of aminopyridine, thereby suppressing acid secretion by parietal cells (Goldenring et al., 2000). DMP-777 was administered at doses of 15, 50, and 200 mg/kg/day for periods of up to 6 months in CD-1 rats (Goldenring et al., 2000). At the highest dose (200 mg/kg/day), DMP 777 caused a profound loss of gastric parietal cells, leading to a rapid increase in serum gastrin levels and a marked foveolar hyperplasia in the gastric mucosa (Goldenring et al., 2000). This hypergastrinemia mimics the elevated serum gastrin levels observed in humans with chronic atrophic gastritis and achlorhydria, which is thought to promote epithelial proliferation and potentially contribute to carcinogenesis (Zhang and Tang, 2025b; Waldum and Modlin, 2025). The treatment induced alterations in gastric cell lineages, including a reduction in chief cells, enterochromaffin-like cells, and somatostatin cells, while foveolar mucous cells expanded significantly (Goldenring et al., 2000). Despite the significant histological changes, no neoplastic transformation was observed, and the effects were fully reversible (Goldenring et al., 2000). Upon cessation of DMP-777 treatment for 3 or 6 months, the gastric mucosa restored its normal architecture and cellular composition within 3 months (Goldenring et al., 2000). Within 3 days of DMP-777 treatment in mice, there is a rapid loss of parietal cells, and continuous administration for 10–14 days leads to the development of SPEM (Goldenring et al., 2000). Notably, after discontinuing DMP-777 for 7–14 days, all lesions are completely reversed (Goldenring et al., 2000). Further investigations have demonstrated that DMP-777 exerts an intracellular elastase inhibitory effect, which may help prevent tumor-like lesions by inhibiting neutrophil function and innate immune responses, resulting in a diminished inflammatory response in the gastric mucosa (Ogawa et al., 2006).

L635, which is structurally similar to DMP-777, does not inhibit neutrophil elastase, leading to a significant inflammatory response when parietal cells are lost. This results in the appearance of highly proliferative and intestinal-type SPEM (Nam et al., 2010). The inflammatory component observed with L635 more closely mimics human chronic atrophic gastritis, where ongoing inflammation drives epithelial damage and metaplastic transformation. However, due to the limited availability of L635, it remains unclear whether chronic administration may lead to dysplasia.

High-dose tamoxifen acts as a protonophore for parietal cells, resulting in acid reflux into these cells and ultimately causing their death. While tamoxifen is primarily known as a selective estrogen receptor modulator used in breast cancer treatment, its gastric effects in this model are unrelated to its clinical use but rather exploit its protonophoric properties to induce acute parietal cell injury. Administration of 5 mg of emulsified tamoxifen daily for 3 days reduces parietal cell numbers by 90%. Notably, high-dose tamoxifen (5 mg/20 g mouse) induces SPEM within 3 days, with lesions reversing after 2–3 weeks of discontinuation (Keeley et al., 2019; Saenz et al., 2016). Further studies indicate that tamoxifen induces parietal cell loss and increases cellular proliferation, with the extent of injury correlating with the dosage (Keeley et al., 2019). Specifically, a dose of 25 mg/kg tamoxifen did not cause parietal cell loss, whereas higher doses (50, 100, and 200 mg/kg) resulted in significant gastric damage (Keeley et al., 2019). The proliferative response was most pronounced at the highest doses, with proliferating cells expanding downward in the gastric corpus glands. Interestingly, the mode of tamoxifen administration—be it subcutaneous, oral gavage, or intraperitoneal injection—did not significantly affect the gastric injury response, and the inclusion of ethanol in the tamoxifen preparation did not alter the observed damage (Keeley et al., 2019). Furthermore, gastrin was found to be dispensable for both the induction of tamoxifen-induced damage and subsequent tissue repair, indicating that these processes are independent of gastrin signaling (Keeley et al., 2019). This gastrin-independent mechanism differs from human disease, where hypergastrinemia secondary to achlorhydria is thought to play a role in driving epithelial proliferation and metaplastic changes. Using rabbit gastric tubulovesicles, researchers demonstrated that tamoxifen disrupts the proton gradient, a mechanism that is also shared with DMP-777 and L635. This disruption leads to the backflow of acid into parietal cells, likely contributing to their loss following high-dose tamoxifen administration (Manning et al., 2020). At concentrations ranging from 1 to 10 mM, tamoxifen induces a rapid quenching of acridine orange fluorescence, indicating a disruption of acid sequestration (Manning et al., 2020).

Single-cell RNA sequencing of cells from the gastric corpus of healthy, drug-injured (tamoxifen-induced), and inflammation-induced (TxA23) mice revealed that the metaplastic changes in both drug-induced and chronic inflammation-induced SPEM cells are transcriptionally similar (Bockerstett et al., 2020). This finding suggests that SPEM may represent a common metaplastic response regardless of the initiating injury, which has implications for understanding the role of SPEM in human gastric pathology where multiple factors (*Hp* infection, autoimmunity, drugs) can lead to similar metaplastic changes. However, the reversible nature of SPEM’s contribution to tissue regeneration, particularly in the acute short-term context involving high-dose tamoxifen, DMP-777, and L-635, raises controversy regarding whether acute SPEM should be considered a true precancerous condition (Huang et al., 2024). This reversibility is a major limitation of these models compared to human disease, where metaplastic changes in chronic atrophic gastritis are typically irreversible and may progress to dysplasia and adenocarcinoma. The rapid reversibility of drug-induced SPEM suggests it may represent an adaptive response rather than a true precancerous lesion, limiting the translational relevance of these acute injury models for understanding human gastric carcinogenesis (Sáenz and Mills, 2018). Supplementary Table 3 provides an overview of acute chemical injury–induced animal models used to study gastric precancerous changes.

### 3.5 Genetically engineered mouse models

Parietal cell loss is a critical prerequisite for the occurrence and progression of gastric precancerous lesions (Ye et al., 2024; Liu X. et al., 2022). In humans, parietal cell loss occurs in the setting of chronic atrophic gastritis, leading to achlorhydria, hypergastrinemia, and progression through the Correa cascade toward intestinal metaplasia and dysplasia. However, the ability of *Helicobacter* infection to induce parietal cell loss varies across mouse strains due to differences in immune system responses (Druffner et al., 2024). This inter-strain variability results in inconsistent lesion development timelines, with most lesions emerging slowly and asynchronously as they advance toward dysplasia.

Notably, lesion progression in standard *Hp* models is protracted. Studies have shown that SPEM and extensive oxyntic atrophy typically appear around 6 months post-infection, whereas dysplastic changes may require over 10 months of sustained infection to manifest (Fox et al., 2002; Jeong et al., 2021). Although this timeline closely parallels human disease, it presents practical challenges for experimental research. Combining *Helicobacter* infection with N-nitroso compounds, such as MNU or MNNG, can accelerate tumorigenesis and increase tumor incidence compared to either agent alone. However, lesions induced by this approach often arise in the gastric antrum and may not fully align with the Correa model of gastric carcinogenesis, as the resulting tumors frequently develop *de novo* from mutational events rather than through progressive metaplasia and dysplasia (Ansari and Yamaoka, 2022).

Genetically engineered mouse models have emerged as valuable tools for gastric cancer research. These models enable targeted investigation of molecular pathways implicated in human gastric tumorigenesis, including dysregulation of oncogenes, tumor suppressor genes, and inflammatory mediators identified through human genetic and molecular studies (Tu et al., 2008; Leibold et al., 2024; Soutto et al., 2011). These models offer a more accurate simulation of intestinal-type gastric cancer by allowing controlled manipulation of gene expression, inflammatory pathways, or specific oncogenic mutations. Importantly, these models can recapitulate distinct molecular subtypes of gastric cancer—such as chromosomal instability and genomically stable (diffuse) types—as classified by The Cancer Genome Atlas (TCGA) (Seidlitz et al., 2019). For example, one study demonstrated that somatic editing of key genes in the gastric epithelium could generate tumors representative of the full spectrum of non-EBV-associated human gastric cancers, including classical intestinal-type adenocarcinomas with invasive glandular architecture (Leibold et al., 2024).

Compared to *Helicobacter*-induced and chemical carcinogen models, genetically engineered mouse models offer significant advantages, including synchronized lesion development and controlled experimental timelines. These features make genetically engineered mouse models particularly valuable for dissecting the functional roles of specific genes and signaling pathways in the initiation and progression of gastric precancerous lesions.

#### 3.5.1 Gastrin-related models

Gastrin, produced by G cells in the gastric antrum, plays a crucial role in regulating glandular homeostasis by stimulating enterochromaffin-like cells to release histamine, which in turn promotes acid secretion from parietal cells. In humans, elevated serum gastrin levels are observed in patients with chronic atrophic gastritis due to achlorhydria-induced loss of negative feedback, and hypergastrinemia has been associated with increased risk of gastric neuroendocrine tumors and adenocarcinoma (Waldum and Mjønes, 2023; Waldum and Mjønes, 2020). Endogenous gastrin levels can increase due to the loss of parietal cells, thereby stimulating glandular cell proliferation. Some researchers have hypothesized that gastrin contributes to the occurrence and progression of gastric precancerous lesions (Dockray et al., 2001). The gastrin-deficient (GAS^−/−^) model demonstrated a sequence of gastric changes leading to cancer (Zavros et al., 2005). At 12 months, these mice exhibited significant atrophy of the parietal cells, particularly in the gastric glands, followed by antral inflammation characterized by an increased number of polymorphonuclear neutrophils (Zavros et al., 2005). This progression mirrors aspects of human autoimmune gastritis, where parietal cell loss leads to achlorhydria and compensatory antral inflammation. This was accompanied by intestinal metaplasia, as indicated by the expression of mucin two and villin in the antral region (Zavros et al., 2005). Importantly, this model is one of the few that develops true intestinal metaplasia with goblet cells expressing mucin 2 and absorptive cells expressing villin, closely resembling human intestinal metaplasia in the Correa cascade. Over time, these changes progressed to severe dysplasia in the antral region, marked by the loss of glandular architecture. Gastric carcinoma was observed in 60% of the GAS^−/−^ mice, with tumors exhibiting invasive behavior, including submucosal invasion in the affected areas; however, these tumors were localized to the antral region and did not metastasize (Zavros et al., 2005). The lack of metastasis represents a limitation compared to human gastric adenocarcinoma, which frequently metastasizes to lymph nodes, liver, and peritoneum. Inflammation, characterized by increased neutrophil infiltration, was a prominent feature in the GAS^−/−^ mice, especially in the antrum (Zavros et al., 2005). Additionally, T lymphocytes were found in greater numbers in the antral region of GAS^−/−^ mice (Zavros et al., 2005). In wild-type mice, DMP-777-induced SPEM required at least 10 days of intervention, while SPEM occurred within 1–3 days in GAS^−/−^ mice treated with DMP-777 (Nomura et al., 2005a). Further studies using N-nitroso compounds (MNU) to induce gastric cancer in various genetic backgrounds revealed that GAS^−/−^ mice exhibited greater susceptibility to gastric cancer following MNU treatment, while gastrin overexpression conferred resistance (Tomita et al., 2011). This suggests that gastrin may inhibit the occurrence and progression of MNU-induced gastrin-dependent gastric adenocarcinoma, which is associated with its regulation of trefoil factor 1 gene silencing and associated epigenetic changes (Tomita et al., 2011). This protective effect of gastrin contrasts with clinical observations where hypergastrinemia is associated with increased gastric cancer risk, highlighting species-specific differences in gastrin signaling.

In another model, researchers expressed human gastrin in mouse pancreatic β-cells, creating a hypergastrinemia model known as INS-GAS. This model was designed to mimic the hypergastrinemia observed in human patients with achlorhydria secondary to chronic atrophic gastritis, Zollinger-Ellison syndrome, or chronic proton pump inhibitor use (Rais et al., 2022; Lee et al., 2019). In the early stages (1–4 months), slight increases in gastrin levels were observed, accompanied by increased gastric acid secretion and elevated parietal cell numbers (Wang et al., 2000). However, in the later stages (5 months and beyond), gastrin levels rose significantly, while parietal cell numbers decreased and gastric acid secretion diminished (Wang et al., 2000). This biphasic response mirrors the progression observed in human chronic atrophic gastritis, where initial hypergastrinemia stimulates acid production until progressive parietal cell loss leads to achlorhydria. By 20 months, the mice exhibited gastric atrophy, intestinal metaplasia, dysplasia, and invasive gastric cancer, though no significant metastasis was noted (Wang et al., 2000).

The synergistic effects of hypergastrinemia and *Hf* infection were later demonstrated in INS-GAS mice. This combination models the clinical scenario where patients with achlorhydria and hypergastrinemia are at increased risk for *Hp* colonization and subsequent gastric cancer development. Infection with *Hf* significantly accelerated the progression to intramucosal carcinoma, with submucosal and intravascular invasion occurring within 6–7 months of infection (Wang et al., 2000). Fox et al. (2003) later incorporated *Helicobacter* infection into the model and observed that *Hf* infection in INS-GAS mice led to adenocarcinoma development within 24 weeks, with most lesions showing submucosal and vascular/lymphatic invasion. Compared to *Hp* infection, *Hf* infection resulted in more severe lesions, including moderate to severe inflammation, cystic dilation, and dysplasia. Moreover, *Hp* infection in male mice led to significant SPEM and *in situ* carcinoma, while no tumors were observed in female mice. All male mice infected with *Hf* developed adenocarcinomas, most with submucosal and vascular invasion; conversely, female mice exhibited cystic dilation and dysplasia but did not develop tumors (Fox et al., 2003). This gender difference parallels epidemiological observations in humans, where gastric cancer incidence is approximately twice as high in males compared to females, particularly for intestinal-type gastric cancer (Jemal et al., 2011). Thus, the pathological changes induced by *Hp* and *Hf* infections in the INS-GAS mouse model differ significantly, and gender plays an important role in lesion progression.

Subsequent research investigating the role of *Hp* infection and the microbiota in gastric inflammation and the development of gastric lesions in the INS-GAS mouse model has revealed that *Hp* infection in germ-free INS-GAS mice led to a significant reduction in gastric inflammation and delayed the onset of gastric metaplasia and dysplasia (Lertpiriyapong et al., 2014). These findings support emerging clinical evidence that the gastric microbiome composition influences *Hp*-induced pathology in humans, with certain bacterial species potentially modulating inflammation and cancer risk (Fu et al., 2024). These findings suggest that the absence of commensal bacteria in germ-free mice mitigates the escalation of *Hp*-induced inflammation, thereby reducing the risk of developing dysplasia.

Further studies investigated the role of *Hp* infection and microbial colonization in gastric carcinogenesis using the INS-GAS mouse model. The study included six experimental groups of INS-GAS mice: germ-free (GF), restricted Altered Schaedler’s flora microbiota (rASF; consisting of three commensal bacterial species), intestinal flora (IF; complex microbiota), and three Hp-infected groups - mHp (monoassociated with *Hp*), rASFHp (*Hp* plus restricted microbiota), and IFHp (*Hp* plus complex intestinal flora) (Lertpiriyapong et al., 2014).

At 7 months post-infection, male INS-GAS mice colonized with rASF or IF alone developed gastric pathology characterized by mild to moderate gastric corpus inflammation, epithelial hyperplasia, oxyntic gland atrophy, and pseudopyloric metaplasia. While some of these mice exhibited mild to moderate dysplasia, none developed high-grade dysplasia. In contrast, male INS-GAS mice cocolonized with rASFHp or IFHp developed the most severe gastric pathology, with 69% of rASFHp mice and 93% of IFHp mice progressing to high-grade dysplasia, demonstrating the synergistic effect between *Hp* infection and microbial colonization in accelerating gastric carcinogenesis (Lertpiriyapong et al., 2014). This synergistic effect aligns with clinical studies showing that patients with *Hp* infection and altered gastric microbiome composition have higher rates of precancerous lesions and gastric cancer (Conti et al., 2021; Conti et al., 2020; Wang S. et al., 2022).

Invasive neoplasms developed exclusively in *Hp*-infected male mice, with 23% of rASFHp mice and 40% of IFHp mice developing either intramucosal carcinoma (invasion into the lamina propria or muscularis mucosa) or carcinoma extending into or beyond the submucosal margins (Lertpiriyapong et al., 2014). The invasive lesions were characterized by progressive dysplasia with loss of glandular organization, crowding, atypia, and variable globoid dysplasia featuring disorderly stratification of proliferating gastric glandular epithelial cells expanded by large cytoplasmic mucus vacuoles with nuclear margination (Lertpiriyapong et al., 2014). These histological features resemble human intestinal-type gastric adenocarcinoma. Notably, no invasive neoplasms developed in any female INS-GAS mice, consistent with the male predominance observed in human gastric cancer epidemiology (Lertpiriyapong et al., 2014).

The inflammatory response was most pronounced in rASFHp and IFHp-colonized male mice, with significantly elevated expression of proinflammatory genes including TNF-α, IL-17, Nos2, and chemokines such as Cxcl1 and Ccl2. IFHp mice exhibited the highest inflammatory responses, while *Hp* infection was identified as the main driver of elevated systemic inflammatory markers (Lertpiriyapong et al., 2014). This inflammatory cytokine profile mirrors that observed in human chronic gastritis and gastric cancer, where elevated IL-17, IL-6, and TNF-α levels are associated with increased cancer risk and poor prognosis (Bie et al., 2021; Yu B. et al., 2024; Yu et al., 2023; Nie et al., 2023; Low et al., 2020). Supplementary Table 4 provides an overview of gastrin-related animal models used to study gastric precancerous changes.

#### 3.5.2 Inflammatory mediator-induced models

Chronic, uncontrollable inflammation is a critical microenvironment for the occurrence and progression of gastric cancer. This concept is strongly supported by human studies showing that chronic inflammatory conditions, including *Hp* gastritis and autoimmune gastritis significantly increase gastric cancer risk through sustained production of inflammatory mediators, reactive oxygen species, and growth factors (Waldum and Fossmark, 2023; Zhang et al., 2023). By constructing gene-edited mice to regulate specific molecules in inflammatory signaling pathways and observing changes in disease phenotypes, researchers can analyze the role of these inflammatory molecules in promoting the development and progression of gastric precancerous lesions.

Tu et al. (2008) created a high-expression transgenic mouse model for interleukin-1β by specifically expressing human interleukin-1β in gastric parietal cells. This model is particularly relevant as interleukin-1β polymorphisms in humans, especially the IL-1B-31C and IL-1RN2/2 genotypes, are associated with increased gastric cancer risk, particularly in *Hp*-infected individuals, by promoting hypochlorhydria and enhanced inflammatory responses (de Brito et al., 2018; Sultana et al., 2018; Raza et al., 2017; Nezamzadeh et al., 2021; Jafrin et al., 2021). Over 70% of mice older than 1 week developed gastric mucosal atrophy, intestinal metaplasia, and dysplasia, with 30% eventually progressing to high-grade dysplasia or gastric adenocarcinoma, although no submucosal invasion was observed (Tu et al., 2008). The rapid progression observed in this model contrasts with human disease, where interleukin-1β-mediated gastric pathology typically develops over decades, suggesting that the high-level transgenic expression may not accurately reflect physiological interleukin-1β effects in humans.

In studies involving mice that overexpress murine interferon-γ specifically in gastric parietal cells, researchers observed a progression of pathophysiological changes (Syu et al., 2012). Interferon-γ is clinically significant as it is the predominant cytokine produced by Th1 cells in human *Hp* gastritis and is strongly associated with the development of gastric atrophy rather than duodenal ulcer disease (Lenti et al., 2022; Canedo et al., 2008). Initially, these mice developed chronic inflammation, which was accompanied by a significant loss of both parietal and chief cells (Syu et al., 2012). This atrophy was followed by the expansion of SPEM within the gastric corpus, a metaplastic lineage characterized by Tff2 expression and linked to pre-neoplastic transformation (Syu et al., 2012). Over time, these alterations led to the emergence of dysplasia as early as 3 months of age. Of 39 mice over 1 year of age, four developed more advanced lesions in the antrum, including one adenoma, two polyps, and one adenocarcinoma (Syu et al., 2012). The hyperplastic polyps were characterized by elongated and cystically dilated glands lined by hyperplastic epithelial cells, consistent with regenerative responses to chronic injury. The adenocarcinoma was characterized by marked dysplasia, cellular atypia, and a high mitotic index, representing the most advanced lesion observed in this model (Syu et al., 2012). Although these advanced lesions were predominantly located in the antrum, the initial pathological changes were localized to the gastric corpus, mirroring the corpus-predominant gastritis seen in humans that is associated with increased gastric cancer risk (Syu et al., 2012).

Moreover, the study highlighted that the overexpression of interferon-γ initiated a robust inflammatory response, characterized by an increased presence of inflammatory cells, such as neutrophils, macrophages, and T lymphocytes (Syu et al., 2012). In parallel, key cytokines, including tumor necrosis factor alpha, Interleukin-6, and Interleukin-1β, were significantly upregulated, thereby promoting a pro-inflammatory environment that contributed to the early stages of tumorigenesis. Dysplasia was common in transgenic mice, occurring in 46%–50% of mice at 3–5 months of age and 44%–65% of mice over 12 months of age, while no dysplasia was detected in control mice (Syu et al., 2012). The sole adenocarcinoma that developed showed nuclear β-catenin accumulation, suggesting activation of the canonical Wnt/β-catenin signaling pathway. Nuclear β-catenin accumulation is frequently observed in human gastric adenomas and early gastric cancers, particularly in the intestinal-type, making this an important translational finding (Ebert et al., 2002; Clements et al., 2002). However, the study did not provide evidence of distant metastasis in the examined mice, likely because systematic evaluation for metastatic disease was not performed using methods such as comprehensive histological examination of distant organs or imaging studies (Syu et al., 2012).

Oshima et al. (2004) developed K19-C2mE transgenic mice that express cyclooxygenase-2 (COX-2) and microsomal prostaglandin E synthase-1 (mPGES-1) under the control of the cytokeratin 19 (K19) gene promoter. This model addresses the clinical relevance of COX-2 overexpression in human gastric cancer, where COX-2 is upregulated in approximately 60%–70% of gastric carcinomas and is associated with angiogenesis, invasion, and poor prognosis (Ristimäki et al., 1997; Saukkonen et al., 2003; Wang Z. et al., 2014). The transgenic mice showed approximately twice the normal levels of PGE2 in the glandular stomach.

At 12 weeks of age, the transgenic mice displayed abnormal gastric histology with elongated gastric pits and expanded mucous cell populations throughout the gastric glands. The mucous cells contained acidic mucins that were not normally produced in wild-type gastric mucosa. Their findings revealed increased proliferation with BrdU-labeled cells found not only in the normal neck region but also in the gland bottom, resulting in a twice higher labeling index compared to wild-type mice (Oshima et al., 2004). By 48 weeks of age, large tumorous growths were found in the proximal glandular stomach, with histopathological examinations showing benign metaplastic hyperplasia consisting of Alcian blue-positive mucous cells and other types of differentiated epithelial cells (Oshima et al., 2004). In subsequent experiments, the researchers created K19-Wnt1 transgenic mice to express Wnt1 specifically in the gastric mucosa. These K19-Wnt1 mice were crossed with K19-C2mE mice to generate K19-Wnt1/C2mE compound transgenic mice (Oshima et al., 2006). The combination of COX-2 and Wnt pathway activation reflects the cooperative oncogenic effects observed in human gastric cancer, where both pathways are frequently dysregulated and contribute to the adenoma-carcinoma sequence (Echizen et al., 2016). Notably, by 5 weeks of age, these compound transgenic mice exhibited the presence of SPEM (Oshima et al., 2006). By 10 weeks, irregularly branching proliferative cells displaying dysplastic features were identified in the metaplastic regions (Oshima et al., 2006). At 20 weeks, there was a marked increase in dysplastic cells, leading to the formation of more prominent tumor-like tissues (Oshima et al., 2006). By 30 weeks, the dysplastic areas had expanded, and cancer cells began to emerge, with tumors in this compound transgenic model demonstrating the capability for local invasion into the muscular layer (Oshima et al., 2006). The progression to invasive cancer in this model represents a significant advantage over many other models and more closely approximates human disease progression.

In a separate study, Leung et al. (2008) constructed a COX-2 high-expression transgenic mouse model. Their observations indicated that at 50 weeks of age, these transgenic mice did not develop gastric cancer (Leung et al., 2008). In stark contrast, 25% of wild-type mice treated with MNU developed gastric cancer, while 47.5% of the MNU-treated transgenic mice exhibited gastric cancer development (Leung et al., 2008). This finding demonstrates that COX-2 alone is insufficient for gastric carcinogenesis but acts as a tumor promoter when combined with mutagenic insults, reflecting the multistep nature of human gastric cancer development where COX-2 upregulation typically occurs in the context of chronic inflammation and genetic alterations.

Nguyen et al. (2013) developed transgenic mice expressing CD4-positive T cells specific to the gastric parietal cell antigen, hydrogen/potassium adenosine triphosphatase (H^+^/K^+^ ATPase), to model autoimmune gastritis. This model specifically recapitulates human autoimmune gastritis, which affects approximately 8% of the general population but up to 20% of elderly individuals, and is characterized by autoantibodies against parietal cells and intrinsic factor. This model demonstrated several pathological stages akin to those observed in human gastric cancer development: chronic gastritis, parietal cell atrophy, mucous neck cell hyperplasia, SPEM, and dysplasia (Nguyen et al., 2013). Importantly, these stages appeared sequentially between 2 and 4 months of age, with all mice developing high-grade dysplasia by 12 months (Nguyen et al., 2013). The rapid and predictable progression in this model contrasts with human autoimmune gastritis, where progression to gastric cancer occurs in only 1%–3% of patients over many years, and carcinoid tumors are more common than adenocarcinoma.

Mice genetically modified to express transforming growth factor alpha (TGFα) by inserting the TGFα cDNA under the control of the metallothionein promoter have exhibited significant alterations in cellular differentiation within the stomach, particularly affecting the surface mucous cell population (Sharp et al., 1995). This model is designed to replicate Ménétrier’s disease, a rare human gastropathy characterized by massive foveolar hyperplasia, protein-losing enteropathy, and increased gastric cancer risk, which is associated with TGF-α overexpression and epidermal growth factor receptor activation. At 12 weeks of age, these transgenic mice demonstrated an age-dependent increase in stomach weight due almost exclusively to an accumulation of surface mucous cells in the gastric pits. The vast majority of these cells demonstrated positivity for diastase-resistant PAS staining, indicating neutral mucin-producing cells (Sharp et al., 1995). By 3 months of age, the transgenic mice displayed a pronounced accumulation of surface mucous cells with a dramatic increase in gastric mass, with the pit region thickness increasing from 0.09 ± 0.0 mm in controls to 1.00 ± 0.19 mm in transgenic mice. This was accompanied by a notable depletion of parietal and chief cells, with H^+^,K^+^-ATPase RNA reduced by 15-fold and pepsinogen C RNA reduced by 14-fold compared to controls.

The functional consequences included complete achlorhydria in adult transgenic mice, which failed to respond to pentagastrin stimulation, whereas control mice showed clear acid production in response to the same stimulus (Sharp et al., 1995). Significantly, parietal cell loss occurred without a concomitant loss of their less differentiated precursors, and mature chief cells never developed in the transgenic mucosa, being replaced by their precursors, mucous neck cells (Sharp et al., 1995). The faithful recapitulation of Ménétrier’s disease histology makes this model valuable for studying this specific human condition, though its relevance to common forms of gastric cancer is limited. The study did not report any evidence of malignant transformation, invasive carcinoma, or metastatic disease in these transgenic mice, with the pathological changes remaining as benign hyperplastic and metaplastic alterations (Sharp et al., 1995).

Further investigations revealed that at 13 days, TGFα expression was localized at the base of the gastric glands; however, by 28 days, expression had spread throughout the mucosa, particularly in the foveolar mucous cells (Goldenring et al., 1996a). This expansion was accompanied by a significant increase in the foveolar mucous cell compartment, with these cells expressing the trefoil peptide pS2 (Goldenring et al., 1996a). The progressive expansion of TFF1 (pS2)-expressing foveolar cells mirrors the foveolar hyperplasia observed in human Ménétrier’s disease and reflects the growth-promoting effects of TGF-α on gastric epithelial progenitor cells. Early alterations in cell differentiation were noticeable from 1 to 4 days, particularly in the basal progenitor zone (Goldenring et al., 1996b). By the middle stage (6–11 days), the foveolar compartment exhibited signs of increased proliferation, and by the later stage (13–17 days), hypertrophic changes became evident, marked by significant expansion of the foveolar mucous cell population (Goldenring et al., 1996b).

IL-8Tg mice, which express human interleukin-8, were studied in gastric carcinogenesis models. In one experiment, IL-8Tg mice were infected with *Hf*, and in a separate experiment, IL-8Tg mice were crossed with INS-GAS mice (Asfaha et al., 2013). Human IL-8 (CXCL8) is particularly relevant as it is significantly upregulated in gastric cancer tissues and correlates with tumor invasion, metastasis, and angiogenesis, while also serving as a neutrophil chemoattractant in chronic gastritis (Xu and Yan, 2025; Liu et al., 2021).

In the *Hf* infection model, mice were examined at 6, 12, and 18 months following inoculation. At 6 and 12 months postinfection, no differences in histologic scores were observed. However, at 12 months, gastric dysplasia was detected only in IL-8Tg mice, and at 18 months postinfection, pseudopyloric metaplasia, foveolar hyperplasia, and dysplasia were significantly increased in the stomach of IL-8Tg mice (Asfaha et al., 2013).

Serum IL-8 levels remained undetectable in uninfected controls and *Hf*-infected wild-type mice throughout the 18-month period. In contrast, *Hf*-infected IL-8Tg mice showed significantly increased serum IL-8 levels by 6 months postinfection that remained elevated at 12 and 18 months. Gastric IL-8 mRNA expression was also elevated in *Hf*-infected IL-8Tg mice compared to controls (Asfaha et al., 2013).

In the separate INS-GAS model, double transgenic INS-GAS/IL-8 mice showed accelerated tumor progression and increased invasive tumors compared with INS-GAS mice alone. At 12 months of age, both serum IL-8 levels and gastric IL-8 mRNA expression were significantly increased in INS-GAS/IL-8 mice compared with controls (Asfaha et al., 2013). These gastric carcinogenesis effects were associated with enhanced mobilization of CD11b^+^Gr-1^+^ immature myeloid cells, which contributed to gastrointestinal carcinogenesis by remodeling the tumor microenvironment (Asfaha et al., 2013; Li H. et al., 2024).

Further studies involving the overexpression of stromal cell-derived factor-1 (SDF-1) specifically in gastric parietal cells, coupled with *Hf* infection to induce chronic inflammation, produced noteworthy findings (Shibata et al., 2013). SDF-1 (CXCL12) is clinically significant as it promotes cancer cell migration, invasion, and metastasis in human gastric cancer through interaction with its receptor CXCR4, and high SDF-1 expression is associated with poor prognosis and increased lymph node metastasis (Torruella-Loran et al., 2019).

Analysis of the H/K-ATPase/SDF-1 transgenic (SDF-Tg) mice showed that SDF-1 overexpression results in significant gastric epithelial hyperproliferation, mucous neck cell hyperplasia and spontaneous gastric dysplasia (wild-type mice 0/15 (0%) vs. SDF-Tg mice 4/14 (28.6%), p = 0.042) but has minimal effects on inflammation (Shibata et al., 2013). SDF-Tg mice aged between 3 and 12 months showed minimal inflammation in the stomach but nevertheless exhibited gastric hyperplasia and metaplasia. By 18 months of age, the SDF-Tg mice exhibited dysplasia with marked cystic dilation of glands in corpus and antral tumors (Shibata et al., 2013).

When SDF-Tg mice were infected with *Hf*, at time points between 15 and 18 months post-infection there were significantly greater degrees of pseudopyloric metaplasia, oxyntic atrophy and foveolar hyperplasia. Only the *Hf*-infected SDF-Tg mice exhibited gastric dysplasia and mucous metaplasia at 15–18 months post-infection, while no *Hf*-infected wild-type mice developed severe dysplasia. The study revealed that SDF-1 overexpression resulted in a dramatic expansion of α-smooth muscle actin-positive myofibroblasts and CXCR4-expressing gastric epithelial cells in the progenitor zone, both of which preceded the development of significant gastritis or dysplasia This finding is particularly relevant as cancer-associated fibroblasts and CXCR4^+^ cells contribute to the desmoplastic stroma characteristic of human gastric cancer and promote tumor progression through paracrine signaling (Qin et al., 2021; Li P. et al., 2024; Zhang and Peng, 2018). Gremlin 1-expressing mesenchymal stem cells, the putative precursors of myofibroblasts, were also increased within the dysplastic stomachs of SDF-Tg mice and showed chemotaxis in response to SDF-1 stimulation (Shibata et al., 2013).

SDF-1 overexpression alone resulted in minimal recruitment of hematopoietic cells to the gastric mucosa, although macrophages were increased late in the disease. However, when SDF-Tg mice were crossed with H/K-ATPase-IL-1β mice or infected with *Hf*, there were dramatic synergistic effects on recruitment of bone marrow-derived cells and progression to preneoplasia (Shibata et al., 2013). Supplementary Table 5 summarizes inflammatory mediator–driven models for studying gastric precancerous lesions.

In summary, these inflammatory mediator-induced models successfully recapitulate key aspects of human gastric cancer pathogenesis by targeting specific inflammatory pathways that are dysregulated in human disease. However, the accelerated timelines and often limited invasive potential represent important limitations compared to the decades-long progression and metastatic capability of human gastric cancer. The models are most valuable for understanding the mechanistic roles of individual inflammatory mediators and testing targeted therapeutic interventions.

#### 3.5.3 Oncogene mutation–induced models

Mouse models with oncogenic activation of K-ras have been invaluable in elucidating the stepwise development of gastric carcinoma and the interplay between epithelial transformation and the inflammatory microenvironment. KRAS mutations occur in approximately 10%–15% of human gastric cancers, predominantly in the intestinal-type and microsatellite-unstable subtypes, making these models directly relevant to a significant subset of human disease (Won and Choi, 2022). In one early model, a point mutation at codon 12 (Gly12Val) of the K-ras gene was specifically expressed in gastric mucous neck cells using the cytokeratin 19 (K19) promoter (Brembeck et al., 2003). The G12V mutation is the common KRAS alteration in human gastric cancer, typically occurring in early stages of tumorigenesis and associated with enhanced proliferation and resistance to apoptosis (Fu et al., 2019). Mucous neck cell hyperplasia was observed as early as 3–6 months in the transgenic mice, and this hyperplasia persisted up to 18 months (Brembeck et al., 2003). Concurrently, there was a significant decrease in the number of parietal cells (Brembeck et al., 2003).

Subsequent studies indicated that mutant K-ras expression led to gastric hyperplasia, mucous metaplasia, and pseudopyloric metaplasia as early as 3 months, signifying a shift in cell types from normal gastric cells to mucus-producing cells, particularly at the progenitor cell sites (Okumura et al., 2010). This metaplastic transformation parallels the early changes observed in human gastric carcinogenesis, where KRAS-mutated cells show enhanced mucin production and altered differentiation patterns that precede frank neoplasia. Over time, this progression was associated with gastric inflammation, characterized by the development of oxyntic atrophy and gastric dysplasia between 6 and 12 months, ultimately advancing to high-grade dysplasia and intramucosal carcinoma by 18 months (Okumura et al., 2010).

Despite the progression to carcinoma, there was no evidence of tumor invasion or metastasis, indicating that the observed changes were in the early stages of cancer progression. This limitation reflects a key difference from human KRAS-mutated gastric cancers, which typically demonstrate invasive behavior and metastatic potential, suggesting that additional oncogenic events are required for full malignant transformation. Inflammatory responses were pivotal in this process, with lymphocytic infiltration, particularly of T lymphocytes and macrophages, noted in the gastric mucosa (Okumura et al., 2010). Proinflammatory cytokines, such as interleukin-6 and C-X-C motif chemokine ligand 1, were significantly upregulated, contributing to a chronic inflammatory microenvironment that likely facilitated the progression to gastric dysplasia (Okumura et al., 2010). This inflammatory signature mirrors the tumor microenvironment of human KRAS-mutated gastric cancers, which often exhibit elevated interleukin-6 signaling and enhanced C-X-C motif chemokine ligand 1-mediated neutrophil recruitment.

A refined approach was developed wherein endogenous Kirsten rat sarcoma viral oncogene homolog (Kras) G12D mutations were activated in cytokeratin 19–expressing tissues (CK19CreERT; LSL-KrasG12D) via tamoxifen induction (Ray et al., 2011). This inducible system better recapitulates the timing of KRAS activation in human tumorigenesis, where oncogene activation typically occurs during adulthood rather than during development. Gastric changes were observed beginning 4–6 months post-tamoxifen administration. These changes included gastric metaplasia in the fundus, particularly along the lesser curvature of the stomach (Ray et al., 2011). The lesser curvature localization is clinically significant, as this anatomical region shows higher incidence of gastric cancer in humans, particularly in East Asian populations (Hayashi et al., 2024). Affected areas exhibited foveolar hyperplasia, an extension of mucin-producing cells, and a deeper proliferative zone within the glands (Ray et al., 2011). This metaplasia was associated with a reduction in parietal cell presence and a shift in trefoil factor 2 (Tff2) expression deeper into the glands (Ray et al., 2011). The lesions were predominantly located in the gastric fundus, especially along the lesser curvature, where gastric mucous neck cell hyperplasia and foveolar hyperplasia were notably pronounced (Ray et al., 2011).

Furthermore, the effects of systemic activation of the K-rasG12D gene were investigated using a transgenic model that employed tamoxifen-induced activation of K-ras in the Ubc9-CreERT2 background (Matkar et al., 2011). Within 13–18 days after tamoxifen treatment, K-rasG12D/+ mice started dying and showed enlarged stomachs with large masses along the lesser curvature near the gastroesophageal junction. Histological examination revealed hyperplasia of squamous epithelium in the forestomach and mucus gland metaplasia in the glandular stomach, more prominent at the junction of forestomach and glandular stomach (Matkar et al., 2011). The rapid onset of changes in this systemic model contrasts with human disease, where KRAS-driven gastric carcinogenesis typically evolves over years to decades, highlighting the limitations of ubiquitous oncogene activation (Matkar et al., 2011).

Intestinal metaplasia was confirmed by Alcian blue staining, which identified acidic mucins normally found in intestinal goblet cells. While control mice contained no Alcian blue-stained cells, a significant number of positively stained cells were found in K-ras activated mice, indicating replacement of gastric mucosa with intestinal-type columnar epithelium (Matkar et al., 2011). CDX2, a transcription factor involved in intestinal cell proliferation and differentiation often upregulated in intestinal metaplasia, was also significantly increased in K-ras activated mice (Matkar et al., 2011).

Importantly, no obvious tumor formation was detected in the pancreas, lung, liver, kidney, small intestine and colon within the examined timeframe, except for 1 mouse showing oral papilloma (Matkar et al., 2011). Chronic inflammation was characterized by infiltration of hematopoietic cells throughout the squamous epithelium, with the presence of macrophages in areas of chronic inflammation. Additionally, COX-2 expression was upregulated in hematopoietic cells at sites of inflammation and in surrounding forestomach squamous epithelial cells (Matkar et al., 2011). COX-2 upregulation is a consistent finding in human KRAS-mutated gastric cancers and represents a potential therapeutic target for prevention (Matkar et al., 2011).

Innovative lineage-tracing strategies using inducible Cre-loxP systems targeted to specific gastric stem or progenitor populations have shed further light on the cell-of-origin for K-ras–driven lesions. Understanding the cellular origin of KRAS-driven transformation is crucial for human gastric cancer, as different stem cell populations may give rise to distinct tumor subtypes with varying clinical behaviors. The primary method utilized Mist1-CreERT2 mice, in which Cre recombinase is induced by tamoxifen specifically in cells expressing the bHLH transcription factor Mist1 (Hayakawa et al., 2015). This approach allowed for targeted genetic modifications in Mist1^+^ cells, identified as quiescent stem cells in the gastric corpus isthmus (Hayakawa et al., 2015). The principle behind this method is to selectively activate or knock out specific genes in these stem cells to investigate their roles in normal gastric epithelium maintenance and gastric cancer development (Hayakawa et al., 2015).

In the Mist1-CreERT2; LSL-KrasG12D mice, the formation of Ki67^+^ dysplastic foci containing metaplastic cells was observed by day 14 (Hayakawa et al., 2015). By day 28, these metaplastic and dysplastic foci expanded from the isthmus to the bottoms of the glands, ultimately replacing the entire glands with intestinal metaplasia and dysplasia. In the Mist1-CreERT2; Apc^fl/fl^ mice, nuclear accumulation of β-catenin was detected in Mist1^+^ lineage cells by day 240, yet no dysplasia developed (Hayakawa et al., 2015). This finding demonstrates that APC loss alone is insufficient for gastric tumorigenesis, contrasting with colorectal cancer where APC mutations are typically initiating events (Fearon, 2011).

In the Mist1-CreERT2; LSL-KrasG12D; Apc^fl/fl^ mice, intramucosal intestinal-type gastric cancer with expansion of nuclear β-catenin^+^ cells was observed by day 120. The combination of KRAS activation and APC loss recapitulates the cooperative effects observed in human gastric cancers with both alterations, which typically show more aggressive behavior. In the Mist1-CreERT2; Cadherin 1 (Cdh1)^fl/fl^ mice (Cdh1DMist1), atypical foci with E-cadherin loss appeared in the isthmus by day 10 (Hayakawa et al., 2015). CDH1 (E-cadherin) loss is the hallmark genetic alteration in human diffuse-type gastric cancer, and this model provides insight into the earliest events in this aggressive cancer subtype. In the absence of *Hf* infection, these atypical foci gradually declined and disappeared by day 180. However, in the presence of *Hf* infection, lineage-traced diffuse-type gastric cancer with numerous signet-ring cells was detected at 18 months (Hayakawa et al., 2015). This demonstrates that chronic inflammation is essential for progression of CDH1-deficient cells to frank diffuse-type gastric cancer, mirroring the role of *Hp* infection in human diffuse-type gastric cancer development.

Chronic inflammation was induced in several models, particularly in the Cdh1DMist1 mice infected with *Hf*. This inflammation was critical for the development of diffuse-type gastric cancer (Hayakawa et al., 2015). The study identified C-X-C motif chemokine ligand 12^+^ endothelial cells and C-X-C chemokine receptor type 4^+^ innate lymphoid cells as key components of the perivascular niche that supports gastric stem cells. The CXCL12/CXCR4 axis is highly relevant to human gastric cancer, where it promotes invasion, metastasis, and therapeutic resistance (Dong et al., 2020; Tang et al., 2021; Daniel et al., 2020). These cells were found to be upregulated in the isthmus during the development of diffuse-type gastric cancer (Hayakawa et al., 2015).

By inserting the 2A-CreERT2 cassette into the leucine-rich repeat-containing G-protein coupled receptor 5 (Lgr5) gene locus, a specific animal model was developed to enable the labeling and induction of Lgr5-expressing cells (Leushacke et al., 2017). Lgr5^+^ cells are well-characterized intestinal stem cells, and their presence in the gastric antrum makes this model particularly relevant for studying antral-predominant gastric cancers (Jang et al., 2013). Mice with the Lgr5-2A-CreERT2 construct, along with the KrasG12D mutation, were used to investigate the effects of oncogenic Kras activation in these Lgr5-expressing cells (Leushacke et al., 2017). Four months after tamoxifen induction, metaplastic lesions were observed in the corpus epithelium, characterized by the formation of SPEM(188). These lesions exhibited high levels of phosphorylated mitogen-activated protein kinase and mucin 5AC (Muc5ac) expression, which indicated the development of SPEM (Leushacke et al., 2017). Furthermore, an increased infiltration of macrophages was observed in the submucosal layer of these metaplastic lesions, suggesting an immune response associated with tumor progression (Leushacke et al., 2017). The predominance of tumor-associated macrophages in KRAS-driven lesions reflects the immunosuppressive microenvironment characteristic of human gastric cancers with mitogen-activated protein kinase pathway activation.

Composite genetically engineered models underscore the synergistic effects of K-ras activation with additional tumor suppressor deletions. Till JE et al. (Till et al., 2017) developed a genetically engineered mouse model of gastric adenocarcinoma. This approach models the multi-hit hypothesis of human gastric carcinogenesis, where multiple genetic alterations accumulate to drive progression from normal epithelium to invasive cancer. This model was based on the expression of oncogenic KrasG12D combined with the inactivation of E-cadherin (Cdh1) and tumor protein 53 (p53) in the gastric parietal cell lineage (Till et al., 2017). The model was created by cross-breeding mice with the following genotypes: Tcon mice (Atp4b-Cre; Rosa26LSL-YFP; Cdh1^fl/fl^; Tumor protein p53 (Trp53)^fl/fl^; KrasLSL^G12D/+^) had a complete loss of E-cadherin and p53, with the expression of oncogenic KrasG12D (190). In contrast, Dcon-Ecad Het mice (Atp4b-Cre; Cdh1^fl/+^; Trp53^fl/fl^; KrasLSL^G12D/+^) retained one wild-type allele of Cdh1, which allowed for partial expression of E-cadherin (Till et al., 2017). Three weeks post-birth, Tcon mice showed few or no identifiable gastric parietal cells, accompanied by high-grade dysplastic lesions and intramucosal carcinomas in 100% of the mice (Till et al., 2017). By 6 weeks post-birth, a dramatic increase in lesions was observed, with invasive carcinomas appearing in 40% of the mice (Till et al., 2017). At 9 weeks post-birth, 100% of Tcon mice had developed invasive carcinomas, with tumors spread throughout the stomach and metastases documented in the lymph nodes, lung, and liver (Till et al., 2017). This aggressive phenotype with distant metastases closely recapitulates human diffuse-type gastric cancer, which typically presents at advanced stages with peritoneal and systemic metastases. In contrast, Dcon-Ecad Het mice, up to 123 days post-birth, only showed 20% of mice developing invasive tumors, with no apparent metastasis in the lung or liver (Till et al., 2017). The tumors in these mice were more focal and less invasive, with no distant metastases observed (Till et al., 2017). This dose-dependent effect of CDH1 loss demonstrates the critical role of E-cadherin in maintaining epithelial integrity and preventing metastasis in human gastric cancer.

In another set of experiments, three types of metaplasia models were established using the trefoil factor 1 (Tff1)-Cre bacterial artificial chromosome (BAC) transgenic mouse line to induce gene modifications specifically in the gastric pit lineage (Kinoshita et al., 2019). TFF1 is specifically expressed in gastric surface epithelial cells and is frequently lost in human gastric cancer, making this an appropriate cellular target for modeling gastric tumorigenesis (Mao et al., 2012; Feng et al., 2014). These models included: (Song et al., 2022): Tff1-Cre; LSL-KrasG12D mice, created by crossing Tff1-Cre mice with LSL-KrasG12D mice to express constitutively active Kras in the gastric pit lineage; (GBD, 2019 Asia and All Cancers Collaborators, 2024); Tff1-Cre; Pten^flox/flox^ mice, generated by crossing Tff1-Cre mice with Pten^flox/flox^ mice to delete phosphatase and tensin homolog (PTEN) in the gastric pit lineage; and (Tan et al., 2024) Tff1-Cre; Cdh1^flox/flox^ mice, created by crossing Tff1-Cre mice with Cdh1^flox/flox^ mice to delete Cdh1 in the gastric pit lineage (Kinoshita et al., 2019). PTEN loss occurs in approximately 20% of human gastric cancers and is associated with phosphoinositide 3-kinase/protein kinase B pathway activation and poor prognosis (Tamura, 2006; Tran et al., 2013; Zhao et al., 2019; Chiappini et al., 2017).

In the Tff1-Cre; LSL-KrasG12D mice, 3 weeks post-birth, abnormal glands with ectopic Alcian blue-positive mucins were observed, along with foveolar cell expansion and a loss of normal parietal and chief cells (Kinoshita et al., 2019). The early appearance of Alcian blue-positive mucins reflects the mucin phenotype switching that occurs in human gastric precancerous lesions, where normal gastric mucins are replaced by intestinal-type mucins during the progression through the Correa cascade. By 3 months post-birth, oxyntic atrophy, pseudopyloric metaplasia with SPEM, and increased proliferation of foveolar cells were observed (Kinoshita et al., 2019). However, by 12 months post-birth, no gastric cancer was observed, but persistent metaplastic changes remained (Kinoshita et al., 2019). This finding demonstrates that KRAS activation alone is insufficient for malignant transformation, consistent with human gastric cancer where KRAS mutations are typically accompanied by additional genetic alterations such as TP53, APC, or phosphatidylinositol-4,5-bisphosphate 3-kinase catalytic subunit alpha mutations.

For the Tff1-Cre; Pten^flox/flox^ mice, at 3 months post-birth, similar changes to those observed in the Tff1-Cre; LSL-KrasG12D mice were noted, including foveolar cell expansion, oxyntic atrophy, and pseudopyloric metaplasia with SPEM(191). The similar phenotypes between KRAS activation and PTEN loss reflect the convergent effects of these alterations on PI3K/AKT and MAPK signaling pathways, both of which are frequently dysregulated in human gastric cancer and promote epithelial proliferation and survival (Baghery Saghchy Khorasani et al., 2021; Fattahi et al., 2020; Morgos et al., 2024). In the Tff1-Cre; Cdh1^flox/flox^ mice, 1 week post-birth, epithelial shedding and the presence of signet ring-like cells were observed (Kinoshita et al., 2019). The rapid appearance of signet ring-like cells following CDH1 loss precisely recapitulates the hallmark cellular morphology of human diffuse-type gastric cancer, where loss of E-cadherin-mediated cell adhesion leads to the characteristic discohesive growth pattern. Four weeks post-birth, the epithelium became thickened and dysplastic, with a loss of normal gastric lineages and increased inflammatory cell infiltration (Kinoshita et al., 2019). By 12 weeks post-birth, there was complete replacement of glandular epithelium with squamous epithelium, originating from the forestomach (Kinoshita et al., 2019). The squamous metaplasia observed represents a rodent-specific response not seen in human gastric pathology, highlighting a species difference that limits the translational relevance of this particular finding.

Additionally, in the Tff1-Cre; LSL-KrasG12D mice, there was increased infiltration of F4/80-positive macrophages, along with upregulation of chemokine (C-X-C motif) ligand 2 and amphiregulin mRNA levels (Kinoshita et al., 2019). CXCL2 upregulation is clinically relevant as this neutrophil chemoattractant is elevated in human gastric cancer and correlates with increased angiogenesis and tumor progression, while amphiregulin promotes epithelial proliferation and is frequently overexpressed in human gastric adenocarcinomas (Kasashima et al., 2017; Sawant et al., 2023; Li et al., 2017). In the Tff1-Cre; Pten^flox/flox^ mice, there was increased infiltration of F4/80-positive macrophages, along with upregulation of phosphorylated extracellular signal-regulated kinase 1/2 and protein kinase B (Kinoshita et al., 2019). The activation of ERK1/2 and AKT pathways reflects the PI3K/PTEN signaling disruption that occurs in approximately 15%–20% of human gastric cancers and is associated with enhanced cell survival, proliferation, and therapeutic resistance (Zhao et al., 2019; Xu et al., 2018). Lastly, in the Tff1-Cre; Cdh1^flox/flox^ mice, there was increased infiltration of F4/80-positive macrophages, myeloperoxidase-positive neutrophils, and α-smooth muscle actin-positive myofibroblasts (Kinoshita et al., 2019). This inflammatory infiltrate, particularly the presence of cancer-associated fibroblasts (α-SMA^+^ myofibroblasts), closely mirrors the desmoplastic stroma characteristic of human diffuse-type gastric cancer, which contributes to treatment resistance and poor prognosis (Fuyuhiro et al., 2011; Fuyuhiro et al., 2010).

Thiem et al. (2016) utilized Tff1-CreERT2 mice crossed with mice harboring conditional oncogenic mutations in Kras (KrasLSL-G12D) or Braf (BrafLSL-V600E). BRAF V600E mutations occur in approximately 2%–5% of human gastric cancers and are associated with microsatellite instability and better response to immunotherapy, making this comparison between KRAS and BRAF particularly relevant for understanding pathway-specific effects (Sim et al., 2019). In the Tff1-CreERT2; KrasLSL-^G12D/+^ mice, at 9 months post-tamoxifen induction, large adenomas were detected in one-third of the mice (Thiem et al., 2016). These adenomas were characterized by glandular structures, goblet-like cells, and extensive staining for activated signal transducer and activator of transcription 3 (STAT3) (Thiem et al., 2016). STAT3 activation is a key oncogenic pathway in human gastric cancer, particularly in intestinal-type tumors, where it promotes proliferation, angiogenesis, and immune evasion through IL-6 and IL-11 signaling (Qi et al., 2020; Wang YC. et al., 2014; Wang et al., 2013). Tumors showed signs of metaplasia, including SPEM and pseudointestinal metaplasia. In the Tff1-CreERT2; BrafLSL-V600E/+ mice, at 8 months post-tamoxifen induction, large adenomas were detected in the antrum of 10/15 mice, with similar histopathologic features to those in the KrasLSL-^G12D/+^ mice (Thiem et al., 2016). The similar phenotypes between KRAS and BRAF mutations reflect their convergent effects on MAPK pathway activation, though the slightly higher penetrance and faster kinetics with BRAF may reflect the stronger oncogenic signal of V600E mutations compared to G12D.

The Iqgap3-2A-CreERT2; Kras^G12D/+^ mouse model was used to induce the expression of KrasG12D specifically in Iqgap3^+^ rapidly proliferating isthmus stem cells in the stomach corpus (Matsuo et al., 2021). IQGAP3 is overexpressed in human gastric cancer and promotes cell migration and invasion through regulation of the actin cytoskeleton, making it a relevant target for studying cancer stem cell behavior (Jinawath et al., 2020; Oue et al., 2018). This model was developed to study the effects of oncogenic Ras activation. Three months post-tamoxifen injection, the mice developed pseudopyloric metaplasia, which was characterized by a massive induction of Muc5ac^+^ surface mucous cells, a reduction of parietal cells, and a Ki67^+^ proliferative cell zone that was restricted to the lower neck zone of the gastric glands (Matsuo et al., 2021). Additionally, the expression of cancer stem cell markers, such as CD44v10, was observed at the base of the metaplastic glands (Matsuo et al., 2021). CD44 variant isoforms, particularly CD44v8-10, are established cancer stem cell markers in human gastric cancer and are associated with chemotherapy resistance and metastatic potential (Lau et al., 2014). Pancreatic and duodenal homeobox 1 (Pdx1), which is frequently expressed in pseudopyloric glands and intestinal metaplasia, was detected in corpus units with oncogenic Ras signaling (Matsuo et al., 2021). PDX1 expression in gastric metaplasia is significant as this transcription factor is normally restricted to pancreatic and duodenal tissues, and its ectopic expression in human gastric intestinal metaplasia indicates aberrant developmental programming. Moreover, the induction of Tff2 was observed throughout the aberrant glandular structure (Matsuo et al., 2021). The induced characteristics in this model resembled features of Menetrier’s disease, a condition marked by gastric mucosal changes and protein loss. While Ménétrier’s disease is rare in humans, the molecular mechanisms involving TGF-α/EGFR signaling and foveolar hyperplasia provide insights into growth factor-driven gastric pathology.

In a separate study, Choi et al. (2016) expressed Kras G12D in mouse gastric chief cells and found that SPEM appeared after 1 month. This finding supports the emerging concept that gastric chief cells may serve as cells-of-origin for metaplastic transformation, particularly in the context of chronic atrophic gastritis where chief cell loss precedes SPEM development. By 3–4 months, the condition progressed to intestinal metaplasia, and by 4 months, 13% of the mice exhibited invasive lesions (Choi et al., 2016). The progression from SPEM to intestinal metaplasia to invasion recapitulates the metaplasia-dysplasia-carcinoma sequence observed in human gastric carcinogenesis, though the timeline is accelerated compared to the decades-long progression in humans. Further investigation used the Mist1-Kras mouse model to explore the effects of sustained *Hp* infection on gastric dysplasia (O'Brien et al., 2021). At 2 weeks post-induction, Hp^+^/KRAS^+^ mice showed a slight increase in inflammation. At 6 weeks post-induction, there was severe inflammation, altered macrophage polarization, and increased CD44 expression, with decreased trefoil factor 3 expression (O'Brien et al., 2021). The loss of trefoil factor 3 expression is clinically significant as this protective factor is frequently downregulated in human gastric cancer and its loss is associated with increased epithelial damage and tumor progression (Taniguchi et al., 2018; Latorre et al., 2023). At 12 weeks post-induction, increased trophoblast antigen 2 expression was noted, along with higher proliferation of dysplastic glands and a significant increase in Ki67^+^ cells (O'Brien et al., 2021). Trophoblast antigen 3 is an emerging biomarker in human gastric cancer that correlates with poor prognosis and is being investigated as a therapeutic target for antibody-drug conjugates (Zhang et al., 2016; Riera et al., 2020; Jang et al., 2024). In terms of immune cell infiltration, T cells showed an increased number of cluster of differentiation 3 (CD3)^+^, CD4^+^, and CD8α^+^ T cells in the Hp^+^/KRAS^+^ mice, with many T cells expressing forkhead box protein P3 and programmed cell death protein 1 (O'Brien et al., 2021). The presence of FOXP3^+^ regulatory T cells and PD-1^+^ exhausted T cells reflects the immunosuppressive microenvironment characteristic of human gastric cancer, which contributes to immune evasion and may predict response to checkpoint inhibitor therapy (Wang et al., 2024; Nagase et al., 2017; Kim KJ. et al., 2014). Regarding macrophages, there was an increased presence of F4/80^+^ cells, with fewer M2 macrophages in the Hp^+^/KRAS^+^ mice compared to the Hp^−^/KRAS^+^ mice (O'Brien et al., 2021). The shift away from M2 macrophages suggests that KRAS activation combined with chronic infection promotes a more inflammatory M1-like phenotype, which may have both tumor-promoting and tumor-suppressive effects depending on the stage of carcinogenesis.

Contrasting with the findings of Choi et al. (2016), Huang et al. (2024) used the Mist1-CreERT; KrasLSL-G12D model to induce SPEM and metaplastic transformation within 3 months. However, their study revealed that Kras mutation alone did not induce gastric dysplasia or cancer, emphasizing the necessity of additional genetic hits for tumorigenesis (Huang et al., 2024). This discrepancy highlights the importance of cellular context and genetic background in determining oncogene effects, reflecting the heterogeneity observed in human KRAS-mutated gastric cancers where additional cooperating mutations determine clinical behavior.

In another study, a Tff2-T2A-CreERT2 knock-in mouse line was employed, where tamoxifen-inducible Cre recombinase is driven by the Tff2 promoter (Tu et al., 2025). TFF2^+^ cells represent a distinct population of gastric progenitor cells that normally reside in the isthmus and are expanded in human gastric metaplasia, making this model particularly relevant for studying the cellular origins of gastric cancer. This model facilitated lineage tracing and conditional genetic manipulation specifically in Tff2^+^ isthmus progenitor cells of the gastric corpus (Tu et al., 2025). In Tff2-CreERT; KrasLSL-^G12D/+^ mice with Kras activation, the following observations were made: during the first 5 days post-induction, no significant increase in Tff2^+^ cells or SPEM formation occurred unless injury was present (Tu et al., 2025). However, by day 5 after high-dose tamoxifen induction, there was a rapid formation of tdTomato^+^ CD44v9^+^ SPEM cells. At 2 months, metaplasia (SPEM) peaked, with increased numbers of Ki-67^+^ and Tff2^+^ cells (Tu et al., 2025). By 3 months, the initial appearance of dysplasia was noted from tdTomato^+^ isthmus-resident cells, and by 6 months, there was an expansion of dysplasia and increased trophoblast cell surface antigen 2 (Trop2)^+^ lesions (Tu et al., 2025). At 12 months, there was a further increase in dysplastic lesions. In the Hp^+^ Kras group, moderate-to-severe dysplasia was accelerated by 3 months (Tu et al., 2025). The acceleration of dysplasia by *Helicobacter* infection mirrors the clinical observation that *Hp*-positive patients with gastric precancerous lesions have higher rates of progression to gastric cancer compared to *Hp*-negative patients. At 6 months, 72.3% of TROP2^+^ dysplasia traced back to Tff2^+^ progenitors (Tu et al., 2025). This lineage-tracing data provides direct evidence that TFF2^+^ isthmus progenitor cells can serve as cells-of-origin for gastric dysplasia, supporting the stem cell theory of cancer development in human gastric carcinogenesis. By 12 months, the prevalence of dysplasia was high, with both metaplasia and dysplasia coexisting, although spatially separated (Tu et al., 2025). The spatial separation of metaplasia and dysplasia reflects the field effect observed in human gastric cancer, where different areas of the stomach may show varying degrees of progression along the Correa cascade. Supplementary Table 6 summarizes oncogene mutation–induced animal models for gastric precancerous lesions.

Collectively, K-ras gastric cancer models--especially when combined with additional pathway aberrations and chronic infection--provide unprecedented insight into the cellular and molecular mechanisms of human gastric tumorigenesis. The lineage-tracing capabilities allow identification of cancer stem cells and their differentiation programs, while the combination models demonstrate the cooperative effects of multiple oncogenic hits. However, the accelerated timelines and variable invasive potential highlight the need for longer-term studies and additional genetic modifications to fully recapitulate the decades-long progression and metastatic capability characteristic of human gastric cancer.

#### 3.5.4 Tumor suppressor gene mutation–induced models

Mouse models with targeted mutations in tumor suppressor genes have provided valuable insights into the mechanisms underlying gastric carcinogenesis. These models are particularly relevant as tumor suppressor gene inactivation is a hallmark of human gastric cancer, with frequent alterations in genes such as TP53, RUNX3, APC, and CDKN1B/p27 depending on the tumor subtype (Choi et al., 2018; Tsang et al., 2011; Tsang et al., 2010; Chen et al., 2010; Bencivenga et al., 2021). Ito et al. (2011) constructed a Runx3 gene knockout mouse model and observed significant changes in the gastric epithelium. RUNX3 is frequently inactivated in human gastric cancer through hypermethylation, chromosomal deletion, or protein mislocalization, and its loss is associated with both intestinal-type and diffuse-type gastric cancers, making this model highly clinically relevant (Chen et al., 2010; Na et al., 2015). At 6 months of age, the gastric epithelium exhibited hyperplasia, loss of chief cells, and the development of mucin 6- and TFF2-expressing metaplasia (Ito et al., 2011). The loss of specialized gastric cell types (chief and parietal cells) coupled with expansion of TFF2^+^ cells recapitulates the atrophic gastritis and SPEM development observed in human gastric precancerous lesions, representing early events in the Correa cascade. Additionally, the gastric epithelium showed an intestinal phenotype, characterized by the expression of caudal-type homeobox 2 (Ito et al., 2011). Caudal-type homeobox 2 expression is a key marker of intestinal metaplasia in humans and is considered a reliable indicator of progression toward gastric adenocarcinoma, making its appearance in this model particularly significant for translational research (Chen et al., 2020). At 10 months of age, no gastric cancer formation was detected, but the precancerous changes persisted (Ito et al., 2011). Treatment with MNU accelerated the progression to gastric adenocarcinoma (Ito et al., 2011). In MNU-treated Runx3^−/−^ mice, adenocarcinomas developed in the fundic and pyloric glands, migrated into the submucosa, and, in some cases, further invaded the muscle layer (Ito et al., 2011). The development of invasive adenocarcinoma with submucosal and muscular invasion closely mirrors the progression pattern of human gastric cancer, where loss of RUNX3 function is associated with more aggressive tumor behavior and deeper invasion (Hsu et al., 2009; Wang N. et al., 2016). At 6 months, no significant inflammatory cell infiltration was observed in the stomachs of Runx3^−/−^ mice, and the levels of pro-inflammatory cytokines such as interleukin-6, tumor necrosis factor-alpha, and cyclooxygenase-2 were comparable to those in wild-type mice (Ito et al., 2011). Moreover, no significant infiltration of inflammatory lymphocytes was detected, suggesting that inflammation did not play a significant role in initiating the precancerous changes in the Runx3^−/−^ gastric epithelium (Ito et al., 2011). This observation contrasts with most human gastric cancers, where chronic inflammation precedes malignant transformation, suggesting that RUNX3 loss may represent an inflammation-independent pathway to gastric carcinogenesis.

A subsequent study adopted a genetic knock-in approach to interrogate the effects of a clinically relevant RUNX3 mutant allele (Douchi et al., 2022). The R122C mutation was identified in human gastric cancer patients and represents a clinically relevant alteration that impairs RUNX3 DNA-binding activity, making this model more representative of human disease than complete knockout models. RUNX3R122C knock-in mice were generated by introducing a point mutation (R122C) into the Runx3 gene using homologous recombination. At 6–8 weeks of age, the RUNX3R122C homozygous mice showed normal gastric morphology (Douchi et al., 2022). However, by 6 months of age, precancerous lesions were observed in 7 out of 22 mice (Douchi et al., 2022). These lesions included mucous neck cell hyperplasia and a dramatic increase in isthmus stem/progenitor cells, with a significant reduction in mature parietal, chief, and pit cells (Douchi et al., 2022). The expansion of isthmus stem/progenitor cells reflects the stem cell dysfunction observed in human gastric cancer, where RUNX3 loss disrupts normal epithelial differentiation and promotes a more primitive, proliferative phenotype. In mice older than 1 year, 2 out of 5 mice showed similar precancerous phenotypes (Douchi et al., 2022). Notably, significant inflammatory cell infiltration was detected in the submucosa of the stomach in 6-month-old RUNX3^R122C/R122C^ mice, with macrophages being the predominant inflammatory cells, particularly of the M2-type (F4/80^+^/CD163^+^) (Douchi et al., 2022). The predominance of M2 macrophages is clinically significant as these alternatively activated macrophages are associated with tumor promotion, angiogenesis, and immunosuppression in human gastric cancer, and their presence correlates with poor prognosis. Additionally, increased expression of inflammatory markers such as interleukin-6, tumor necrosis factor-alpha, and interleukin-1 beta was observed in the corpus of RUNX3^R122C/R122C^ mice compared to wild-type mice (Douchi et al., 2022). Further elucidating the consequences of combined tumor suppressor loss, studies with Runx3^−/−^p53^−/−^ double knockout mice revealed significantly heightened tumorigenic potential. The combination of RUNX3 and TP53 loss is frequently observed in human gastric cancer and is associated with particularly aggressive tumor behavior, genomic instability, and poor patient outcomes (Hsu et al., 2009; Wang N. et al., 2016). Gastric epithelial cells from these mice, isolated at embryonic day 16.5, generated rapidly proliferating cell lines that were tumorigenic when injected into nude mice. Although these tumors were invasive at the injection site, they did not exhibit distant metastatic spread (Li et al., 2002). The lack of distant metastasis represents a limitation compared to human gastric cancer with dual RUNX3/TP53 loss, which typically shows high metastatic potential, suggesting that additional genetic alterations may be required for full metastatic competence.

Other tumor suppressor models have highlighted the interactions between genetic susceptibility and environmental factors such as *Hp* infection. This gene-environment interaction is crucial in human gastric cancer, where genetic polymorphisms in tumor suppressor genes significantly modify the cancer risk associated with *Hp* infection. Kuzushita et al. (2005) created a p27kip1 (p27^−/−^) knockout mouse model and infected the mice with *Hp* SS1 strain. CDKN1B/p27 is a cyclin-dependent kinase inhibitor that is frequently downregulated in human gastric cancer through various mechanisms including proteolytic degradation, and its loss is associated with increased proliferation and shorter overall survival (Minarikova et al., 2016). Thirty weeks post-infection, 40% of the p27^−/−^ mice developed intestinal metaplasia, and by 45 weeks, this increased to 67% (Kuzushita et al., 2005). By 60 weeks, 7 out of 12 mice developed significant dysplasia and gastric cancer (Kuzushita et al., 2005). This progressive timeline from intestinal metaplasia to dysplasia to cancer closely recapitulates the natural history of human gastric carcinogenesis, though compressed into a much shorter timeframe than the decades typically required in humans. Similarly, Costa L constructed a upstream stimulatory factor 1 (USF1) knockout model and demonstrated that, following *Hp* infection, these mice reliably developed gastric atrophy, intestinal metaplasia, dysplasia, and additional pre-neoplastic lesions (Kuzushita et al., 2005). USF1 is a transcription factor that regulates gastric acid secretion and epithelial differentiation, and its loss in humans is associated with hypochlorhydria and increased susceptibility to Hp-induced pathology (Costa et al., 2020).

Collectively, these models demonstrate that loss or mutation of key tumor suppressor genes profoundly predisposes the gastric mucosa to metaplasia, dysplasia, and cancer, accurately reflecting the tumor suppressor gene inactivation patterns observed in human gastric cancer. The progression of disease may be further exacerbated by environmental insults such as *Hp* infection or chemical carcinogens, and in some cases, is modulated by the presence or absence of inflammatory responses within the gastric microenvironment. However, the accelerated timelines and variable metastatic potential in these models highlight the complexity of translating findings to human disease, where tumor suppressor loss typically occurs over decades and is accompanied by additional genetic and epigenetic alterations that collectively determine clinical behavior. Supplementary Table 7 summarizes tumor suppressor gene mutation–induced animal models for gastric precancerous lesions.

#### 3.5.5 Glandular cell injury–induced models

Recent advances in understanding gastric carcinogenesis have employed glandular cell injury models, often utilizing genetic knockout strategies to observe the impact of loss of critical epithelial or mucus-associated proteins. These models are particularly relevant as they target genes that are frequently altered in human gastric cancer through genetic mutations, epigenetic silencing, or protein dysfunction, providing mechanistic insights into the role of epithelial barrier disruption in human gastric carcinogenesis. One such approach involved creating claudin-18 (Cldn18)-deficient mice, and significant changes in gastric function were observed. Cldn18 is a tight junction protein specifically expressed in gastric epithelial cells and is frequently downregulated in human gastric cancer, while also serving as a target for novel therapeutic approaches including CAR-T cell therapy and antibody-drug conjugates (Oshima et al., 2013; Moraes et al., 2024; Barrett et al., 2024; Jin et al., 2024). At postnatal day 3, a decrease in intragastric pH was noted, attributed to reduced hydrogen ion (H^+^) secretion from parietal cells, which coincided with the onset of atrophic gastritis (Hayashi et al., 2012). By postnatal day 4, there was a slight reduction in the number of H^+^,K^+^-ATPase-positive parietal cells, which became more pronounced by postnatal day 7 (Hayashi et al., 2012). The early parietal cell loss and acid hyposecretion mirror the pathophysiology of human autoimmune gastritis and advanced chronic atrophic gastritis, where loss of acid-producing capacity creates a permissive environment for bacterial overgrowth and carcinogenesis. At postnatal day 14, gastric acidification was detected and continued to increase. In adulthood, chronic gastritis was observed, marked by the upregulation of interleukin-1 beta, cyclooxygenase-2, and keratinocyte chemoattractant (KC) (a neutrophil chemoattractant), leading to the development of SPEM (Hayashi et al., 2012).

Further examination of Cldn18^−/−^ mice at later time points revealed a clear progression toward malignancy. By 7 weeks of age, there was evident gastric atrophy, foveolar hyperplasia, and increased cellular proliferation—premalignant lesions with significantly higher histopathology activity indices than in controls (Hagen et al., 2018). By 20 weeks, intraepithelial neoplasia with high-grade dysplasia and invasive glands in the submucosa developed (Hagen et al., 2018). At 30 weeks, large polypoid tumors with focal high-grade dysplasia and invasive glands were present in the serosal layer (Hagen et al., 2018). By 2 years, large polypoid tumors with invasive glands occupied approximately 85% of the glandular epithelium (Hagen et al., 2018). This progression from atrophy through dysplasia to invasive adenocarcinoma closely recapitulates the Correa cascade observed in human gastric cancer, though compressed into a much shorter timeframe than the typical decades-long progression in humans.

Significant inflammation was observed in the Cldn18 knockout mice, characterized by increased expression of interleukin-1 alpha and interleukin-1 beta, indicating neutrophil recruitment and activation (Hagen et al., 2018). There was also upregulation of interleukin-8 functional homologs, keratinocyte chemoattractant (C-X-C motif chemokine ligand 1) and lipopolysaccharide-induced chemokine (C-X-C motif chemokine ligand 5) (Hagen et al., 2018). The upregulation of IL-1α/β and CXCL1/5 reflects the inflammatory cascade observed in human gastric cancer, where these cytokines promote neutrophil infiltration, angiogenesis, and epithelial-mesenchymal transition (Li et al., 2014; Zhou et al., 2019). However, no significant changes were found in macrophage or other immune cell markers linked to interleukin-33 expression (Hagen et al., 2018). Neutrophils were the primary inflammatory cells, as indicated by the upregulation of neutrophil-related markers and the absence of significant macrophage markers (Hagen et al., 2018).

Further studies used stomach-type Cldn18-deficient mice (stCldn18^−/−^), which revealed that young mice (<8 weeks old) exhibited acute gastritis, characterized by gastric acid leakage through tight junctions (Suzuki et al., 2019). In middle-aged mice (40 weeks old), chronic active gastritis developed, marked by immune cell infiltration and expression of chemokine (C-C motif) ligand 28 (CCL28) (Suzuki et al., 2019). CCL28 is a mucosal chemokine that recruits T cells and eosinophils and is upregulated in human inflammatory gastric conditions, including Hp gastritis and gastric cancer (Hansson et al., 2008; Berri et al., 2014). In older mice (>60 weeks old), gastric tumors appeared, with 20%–30% of these mice developing tumors. These tumors were associated with the expression of C-X-C motif chemokine ligand 5, suggesting epithelial-mesenchymal transition (Suzuki et al., 2019). C-X-C motif chemokine ligand 5 upregulation is clinically significant as it promotes angiogenesis, invasion, and metastasis in human gastric cancer and correlates with poor prognosis (Zhou et al., 2019; Kasashima et al., 2016).

In Wnt1-overexpressing transgenic mice (Wnt1-Tg), mild gastric pathology was observed (Suzuki et al., 2019). These mice developed small gastric polyps without severe gastritis at a young age (8 weeks old) (Suzuki et al., 2019). However, when crossed with stCldn18−/− mice to generate double-mutant mice (Wnt1-Tg × stCldn18^−/−^), accelerated gastric tumorigenesis occurred (Suzuki et al., 2019). This synergistic effect between Wnt pathway activation and tight junction disruption models the cooperative oncogenic mechanisms observed in human gastric cancer, where Wnt signaling aberrations occur in approximately 30% of cases and often coexist with epithelial barrier dysfunction (Li G. et al., 2018; Yang et al., 2020; Li et al., 2020; Wu et al., 2016). At 40 weeks, all double-mutant mice developed gastric tumors, whereas none of the stCldn18^−/−^ mice developed tumors at this age (Suzuki et al., 2019). The tumors in the double-mutant mice were more aggressive, with increased expression of CXCL5 and other inflammatory markers (Suzuki et al., 2019).

Beyond the claudin family, loss of other epithelial and secretory cell genes also triggered metaplasia, dysplasia, and carcinoma. For example, Nam KT and colleagues created an amphiregulin knockout mouse model and observed that the mice developed SPEM by 10 months of age (Nam et al., 2009). Amphiregulin is an EGFR ligand that is frequently overexpressed in human gastric cancer and promotes epithelial proliferation and survival, making its loss an interesting paradoxical model for studying growth factor dependencies (Jiang et al., 2019; Yasumoto et al., 2011). By 18 months, over 70% of the mice exhibited SPEM, 42% developed intestinal metaplasia, and 28% developed invasive lesions in the gastric fundus (Nam et al., 2009). The development of true intestinal metaplasia in this model is particularly valuable as most murine models develop SPEM rather than the intestinal metaplasia characteristic of human gastric carcinogenesis.

Liu X and collaborators developed both systemic and parietal cell-specific solute carrier family 26 member (Slc26a9) knockout models (Liu X. et al., 2022). SLC26A9 is a chloride/bicarbonate exchanger expressed in gastric parietal cells that is important for acid secretion, and its dysfunction contributes to achlorhydria in human gastric diseases (Xu et al., 2008; Liu et al., 2018). In the systemic knockout mice, parietal cell loss was observed starting at 1 month of age, followed by gastric mucosal atrophy at 2 months (Liu X. et al., 2022). By 6 months, SPEM and intestinal metaplasia had developed, and by 18 months, high-grade epithelial dysplasia and moderately differentiated gastric cancer were present in the gastric body (Liu X. et al., 2022). A similar timeline was observed in the parietal cell-specific knockout model, where SPEM appeared at 6 months, and by 14 months, high-grade epithelial dysplasia and poorly differentiated cancer developed (Liu X. et al., 2022). The progression from parietal cell loss to atrophy, metaplasia, dysplasia, and cancer mirrors the natural history of human corpus-predominant gastric cancer, which is associated with the highest risk of malignant transformation.

Other studies have implicated mitochondrial and cytoskeletal regulators in gastric epithelial homeostasis. Knockout of gene associated with retinoid–IFN–induced mortality 19 (GRIM-19) led to spontaneous gastritis and SPEM, accompanied by robust inflammation primarily involving CD45^+^ leukocytes, myeloperoxidase (MPO)^+^ cells, F4/80^+^ macrophages, an upregulation of M1-like macrophages, and a decline in anti-inflammatory M2 phenotypes—suggesting a pro-inflammatory microenvironment conducive to disease progression (Zeng et al., 2023). GRIM-19 is a component of mitochondrial complex I and STAT3 signaling, and its alterations in human gastric cancer are associated with mitochondrial dysfunction and enhanced inflammatory signaling (Bu et al., 2013; Chen et al., 2012).

Similarly, deletion of Huntington protein-interacting protein 1 (HIP1) resulted in early parietal cell apoptosis, onset of SPEM by 5 weeks, and progressively severe gastric pathology with age (Keeley and Samuelson, 2010). HIP1 regulates endocytosis and cytoskeletal dynamics, and its dysfunction may contribute to the epithelial barrier defects observed in human gastric pathology (Qu et al., 2023).

The mucin family has also featured prominently in these models. For instance, muc6-deficient (Muc6^−/−^) mice were generated to observe gastric cancer progression. Muc6 is specifically expressed in gastric mucous neck cells and deep glands, and its loss or altered expression is frequently observed in human gastric cancer and precancerous lesions (Khattab et al., 2011; Yamanoi et al., 2015). Notably, moderate hyperplastic changes were observed as early as 3 months of age, particularly in the antral region of the stomach (Arai et al., 2024). As the mice aged, the pathological changes evolved; over time, all analyzed Muc6^−/−^ mice (100%) developed visible macroscopic gastric tumors in the antrum by 1 year of age (Arai et al., 2024). Furthermore, at this time point, these tumors exhibited invasion into the submucosal layer (Arai et al., 2024). The 100% penetrance of gastric cancer in this model demonstrates the critical protective role of Muc6 in maintaining gastric epithelial integrity, reflecting clinical observations where Muc6 loss is associated with increased cancer risk in humans.

In addition to tumor development, cellular proliferation increased gradually and significantly, as evidenced by rising numbers of Ki67-positive proliferating cells (Arai et al., 2024). Markers of metaplasia, such as CD44 variant and TFF2, also showed expanding expression patterns over time, indicating progression towards a precancerous phenotype (Arai et al., 2024). The expression of CD44 variants and TFF2 expansion are key markers of human gastric metaplasia and are associated with cancer stem cell properties and progression risk. A prominent inflammatory response characterized this model: there was a marked influx of F4/80-positive macrophages, especially in both the corpus and antrum regions (Arai et al., 2024). Moreover, various T-cell subtypes—including those expressing CD3, CD4, CD8, and forkhead box protein P3—were detected within established tumors, highlighting the complex immune environment associated with tumor progression (Arai et al., 2024). This diverse immune infiltrate, including regulatory T cells (FOXP3^+^), mirrors the immunosuppressive microenvironment of human gastric cancer and may influence therapeutic responses to immunotherapy (Hou et al., 2014; Saleh and Elkord, 2020; Yuan et al., 2011).

Katsha et al. (2013) employed a knockout mouse model lacking Tff1 to further elucidate the sequence of events leading to gastric cancer. Tff1 is specifically expressed in gastric surface epithelial cells and is frequently lost in human gastric cancer through genetic alterations or promoter hypermethylation, making this model highly relevant to human disease (Soutto et al., 2011; Feng et al., 2014; Ge et al., 2012; Cobler et al., 2013). The study demonstrated that AURKA overexpression was associated with inflammation in the gastric mucosa of Tff1-knockout mice, with a direct correlation between chronic inflammation scores and AURKA overexpression (Katsha et al., 2013). Additionally, there was a direct correlation between TNF-α and AURKA mRNA expression (Katsha et al., 2013).

In the context of gastric tumorigenesis, the study built upon previous work showing that development of gastritis was observed in all samples from Tff1-knockout mice irrespective of age and was detectable as early as 8 weeks of age, before the development of dysplastic lesions (Soutto et al., 2011). At 16 weeks of age, approximately half of the Tff1-knockout mice develop low-grade dysplasia, but none has high-grade dysplasia (Soutto et al., 2011). At 12 months of age, more than half of the mice have high-grade dysplasia with multifocal intraepithelial or intramucosal carcinomas (Soutto et al., 2011). Importantly, Katsha et al. (2013) demonstrated that pharmacological inhibition of AURKA with MLN8237 could reverse gastric tumor development in this model. Treatment with MLN8237 for 12 weeks resulted in gastric tumors becoming either substantially reduced in size or completely disappeared, with histological analysis showing a significant decrease in the percentage of high-grade dysplasia and adenocarcinoma compared to control animals (Katsha et al., 2013).

In addition to the TFF1 model, the role of Muc5ac in *Hp*-induced gastric pathology was investigated using Muc5ac-deficient (Muc5ac^−/−^) mice (Muthupalani et al., 2019). Muc5ac is the predominant gastric surface mucin and serves as a first-line defense against *Hp* colonization in humans, making its role in infection-related carcinogenesis particularly relevant. The study included several groups to analyze the specific contributions of Muc5ac in infection and disease progression. In the Muc5ac^−/−^ sham-infected group (without *Hp* infection), mild to moderate inflammation, epithelial defects, and early signs of hyperplasia were present at 16 weeks post-infection (Muthupalani et al., 2019). By 32 weeks post-infection, this group exhibited a significant increase in the total antral pathology index and dysplasia scores, with some mice developing adenomas (Muthupalani et al., 2019).

The Muc5ac^−/−^ *Hp*-infected group further highlighted the protective role of Muc5ac (Muthupalani et al., 2019). At 16 weeks post-infection, these mice demonstrated a significant increase in gastric corpus pathology; however, the extent of mucous metaplasia was notably reduced compared to the wild-type *Hp*-infected group (Muthupalani et al., 2019). This trend continued at 32 weeks post-infection, as the Muc5ac^−/−^ *Hp*-infected mice showed persistent gastric corpus pathology but again with significantly reduced mucous metaplasia compared to their wild-type counterparts (Muthupalani et al., 2019). This finding suggests that while Muc5ac loss increases susceptibility to initial epithelial damage, it may paradoxically reduce certain metaplastic responses, highlighting the complex role of mucins in gastric pathology (Magalhães et al., 2016; Gonciarz et al., 2019; Wang C. et al., 2014).

Furthermore, a separate study utilized knockout mice deficient in alpha-1,4-N-acetylglucosaminyltransferase (A4gnt) to explore the development of gastric dysplasia and its progression to gastric adenocarcinoma (Desamero et al., 2018). A4gnt is crucial for the biosynthesis of gastric-type mucin O-glycans, and its expression is altered in human gastric cancer, where it affects mucin structure and potentially influences bacterial adhesion and immune recognition (Gong et al., 2025; Yamada et al., 2020). The A4gnt gene encodes alpha-1,4-N-acetylglucosaminyltransferase, an enzyme crucial for the biosynthesis of α1,4-N-acetylglucosamine-capped O-glycans in gastric gland mucin (Desamero et al., 2018). Deletion of this gene led to spontaneous gastric cancer, progressing through a distinct sequence: hyperplasia was evident by 5 weeks of age, followed by low-grade dysplasia at 10 weeks. Subsequently, high-grade dysplasia appeared by 20 weeks, and by 50 weeks, adenocarcinoma was present in all A4gnt knockout (A4gnt^−/−^) mice (Desamero et al., 2018). This predictable progression through the dysplasia-carcinoma sequence with 100% penetrance makes this model particularly valuable for studying the molecular mechanisms of gastric cancer progression.

To further investigate the functional role of sulfomucins, researchers generated A4gnt/Chst4 double-knockout mice by crossing A4gnt-deficient mice with sulfotransferase glucosamine-6-sulfotransferase 2 (GlcNAc6ST-2, encoded by the Chst4 gene) knockout mice, the latter being required for sulfomucin biosynthesis (Kawakubo et al., 2019). Sulfomucins are characteristic of intestinal metaplasia in humans and are considered a marker of progression in the Correa cascade, making the study of their biosynthetic pathways clinically relevant (Can et al., 2020). In these double-knockout animals, severe gastric erosion appeared as early as 3 weeks of age, and by 10 weeks, the development of gastritis cystica profunda was observed beneath the erosion (Kawakubo et al., 2019). The frequency of gastritis cystica profunda increased with age, eventually affecting all mice by 60 weeks (Kawakubo et al., 2019). The sequence of histopathological changes, including hyperplasia, low-grade dysplasia, high-grade dysplasia, and adenocarcinoma, paralleled that observed in single A4gnt knockout mice, though adenocarcinoma occurred with slightly lower incidence (Kawakubo et al., 2019).

It is important to note that inflammation played a vital role in these pathological processes, especially in A4gnt/Chst4 double-knockout mice (Kawakubo et al., 2019). Severe gastric erosion was accompanied by an intense infiltration of inflammatory cells, particularly granulocytes, as demonstrated by immunohistochemical staining for Ly-6G/Ly-6C (Gr-1), which identifies lymphocyte antigen 6 complex, locus G/locus C (Kawakubo et al., 2019). Additionally, there was upregulation of inflammation-related genes, including C-X-C motif chemokine ligand 1, C-X-C motif chemokine ligand 5, C-C motif chemokine ligand 2, and C-X-C chemokine receptor type 2 in five-week-old A4gnt/Chst4 double-knockout mice, indicating an early and robust inflammatory response (Kawakubo et al., 2019). Interestingly, these inflammatory genes were downregulated by 50 weeks, coincident with the complete development of gastritis cystica profunda, suggesting a temporal evolution in immune response as the disease progressed (Kawakubo et al., 2019). This temporal shift in inflammatory responses may reflect the transition from an immune-mediated tumor suppressive environment to an immune-exhausted, tumor-permissive state observed in human gastric cancer progression.

In another line of investigation, researchers utilized several mouse models to explore the role of Gastrokine-2 (GKN2) in gastric carcinogenesis progression (Menheniott et al., 2016). GKN2 is a gastric-specific secreted protein that is frequently downregulated in human gastric cancer and may function as a tumor suppressor through regulation of epithelial differentiation and inflammatory responses. These included Gkn2^−/−^ mice, which are homozygous knockout mice lacking the Gkn2 gene; Gkn2^−/−^ gp130^F/F^ mice, compound mutant mice with both the Gkn2 knockout and a knockin mutation at the gp130 cytokine coreceptor locus; and gp130F/F mice, which possess the gp130^F/F^ mutation as a single mutation (Menheniott et al., 2016). The gp130^F/F^ mutation impairs SOCS3-mediated negative feedback of STAT3 signaling, leading to hyperactivation of the IL-6/STAT3 pathway that is frequently dysregulated in human gastric cancer (Ouyang et al., 2017; Kim et al., 2017; Ernst et al., 2008). In Gkn2^−/−^ mice, at baseline (6 weeks), focal hypertrophic lesions appeared in the corpus mucosa with no evidence of spontaneous gastric tumors (Menheniott et al., 2016). By 12 weeks, these lesions persisted with increased severity and included manifestations of atrophic gastritis and mucus metaplasia (Menheniott et al., 2016). At 30 weeks, although hypertrophic lesions remained, they did not progress to malignancy (Menheniott et al., 2016). However, *Hp* infection accelerated the development of atrophic gastritis and extensive mucus metaplasia (Menheniott et al., 2016). This finding demonstrates that Gkn2 loss sensitizes the gastric epithelium to infection-induced pathology, consistent with its proposed tumor suppressor function in humans (Menheniott et al., 2016; Kim O. et al., 2014).

In contrast, Gkn2^−/−^ gp130^F/F^ mice exhibited extensive focal tumorigenesis in the corpus and in the squamous epithelium overlying the limiting ridge of the corpus/forestomach junction by 12 weeks (Menheniott et al., 2016). These tumors were characterized by poorly differentiated and hyperplastic epithelium with increased corpus mucosal thickness (Menheniott et al., 2016). Meanwhile, gp130^F/F^ mice exhibited spontaneous tumorigenesis in the antrum, which gradually encompassed the entire secretory mucosa (Menheniott et al., 2016). By 12 weeks, these mice developed antral tumors with no significant difference in tumor load compared to Gkn2^−/−^ gp130^F/F^ mice (Menheniott et al., 2016). The cooperative effects between GKN2 loss and STAT3 hyperactivation demonstrate the multihit nature of gastric carcinogenesis and highlight the importance of both tumor suppressor inactivation and oncogenic pathway activation in human disease (Qi et al., 2020; Ouyang et al., 2017; Tye et al., 2012).

Moreover, an increased prevalence of macrophages and dendritic cells was observed in the gastric mucosa of Gkn2^−/−^ mice (Menheniott et al., 2016). Additionally, myeloid-derived suppressor cells were found to be reduced in Gkn2^−/−^ mice, potentially contributing to the enhanced inflammatory response (Menheniott et al., 2016). The altered immune cell composition, particularly the reduction in immunosuppressive MDSCs, may paradoxically create a more inflammatory but less tumor-permissive environment, which could explain why GKN2 loss alone was insufficient for tumor development. Supplementary Table 8 presents a summary of glandular cell injury–induced models for gastric precancerous lesions.

Altogether, these glandular cell injury models demonstrate that targeted disruption of key genes regulating barrier function, secretion, and mucosal integrity leads not only to overt inflammation and metaplasia but also to stepwise dysplastic and neoplastic transformation that closely mirrors human gastric carcinogenesis. This progression is strongly influenced by the type and timing of immune cell infiltration, with neutrophilic and macrophage-driven inflammation playing critical roles, thereby illuminating the interplay between epithelial integrity and the immune microenvironment in gastric tumorigenesis. The high penetrance and predictable progression of many of these models make them valuable tools for studying prevention and early intervention strategies relevant to human gastric cancer.

#### 3.5.6 Signaling pathway dysregulation–induced models

Signal pathway dysregulation plays a crucial role in the initiation and progression of gastric cancer, and several mouse models have been developed to elucidate the molecular mechanisms involved. These models target key signaling pathways that are frequently dysregulated in human gastric cancer, including nuclear receptor signaling, cytokine/STAT pathways, TGF-β signaling, innate immunity, and Wnt signaling, providing mechanistic insights into the oncogenic processes driving human disease. Zuo et al. (2019) investigated the oncogenic role of peroxisome proliferator-activated receptor delta (PPARD) in the gastric epithelium by overexpressing this receptor specifically in the villous epithelial cells of the mouse stomach. PPARD is frequently overexpressed in human gastric cancer and promotes lipid metabolism, angiogenesis, and epithelial-mesenchymal transition, while its activation has been associated with poor prognosis and therapeutic resistance. Their findings demonstrated a progressive sequence of precancerous and cancerous changes (Zuo et al., 2019). By 10 weeks of age, mice exhibited SPEM as well as intestinal metaplasia. The rapid progression to both SPEM and intestinal metaplasia is particularly significant as it demonstrates that PPARD overexpression can drive multiple metaplastic pathways simultaneously, reflecting the metabolic reprogramming observed in human gastric carcinogenesis. As the disease advanced, low-grade dysplasia appeared at 25 weeks, followed by high-grade dysplasia at 35 weeks (Zuo et al., 2019). Ultimately, by 55 weeks, invasive adenocarcinoma had developed, with the lesions initially localized to the gastric body—particularly the lesser curvature—before disseminating throughout the entire gastric body (Zuo et al., 2019).

In a related study, Judd et al. (2004) developed a mouse model with a deletion of the Src homology two domain-binding site on the glycoprotein 130 (gp130) subunit, a key component of the interleukin-6 family receptor complex. This gp130Y757F mutation impairs SOCS3-mediated negative feedback regulation, leading to hyperactivation of the IL-6/JAK/STAT3 pathway, which is one of the most frequently dysregulated signaling cascades in human gastric cancer and is associated with inflammation, proliferation, and immune evasion. This genetic modification led to enhanced activity of STAT3 (Judd et al., 2004). The pathological progression in these mice was rapid: as early as 4 weeks, proliferative gastric antral tumors emerged, accompanied by inflammation and ulceration (Judd et al., 2004). The early onset of antral tumors reflects the role of sustained STAT3 activation in human gastric cancer, where this pathway promotes epithelial proliferation, suppresses apoptosis, and creates a tumor-promoting inflammatory microenvironment. Over time, the lesions intensified; by 20 weeks, tumor growth reached its peak, and by 30 weeks, the mice developed gastric mucosal atrophy, intestinal metaplasia, dysplasia, and submucosal invasion (Judd et al., 2004). Although the primary lesions originated in the gastric antrum, they eventually spread to the gastric body as the disease progressed (Judd et al., 2004). The progression from antral to corpus involvement mirrors the field effect observed in human gastric cancer, where inflammatory and oncogenic signals can affect the entire gastric mucosa over time.

Similarly, Nam et al. (2012) utilized a Smad family member 3 knockout (Smad3^−/−^) mouse model to investigate gastric tumorigenesis. SMAD3 is a key mediator of TGF-β signaling that functions as a tumor suppressor in early gastric carcinogenesis but can promote invasion and metastasis in advanced stages, and its loss is observed in approximately 15%–20% of human gastric cancers. At 6 months, these mice developed gastric adenomas and SPEM, with lesions beginning at the distal antrum/body junction along the lesser curvature (Nam et al., 2012). The antrum/body junction is a critical anatomical region in human gastric pathology where different types of mucosa interface and where many gastric cancers arise, particularly in the setting of chronic atrophic gastritis. As the mice aged, tumor invasiveness increased; by 10 months, numerous cystic structures could be observed in the gastric muscle wall, indicating the progression to invasive gastric adenomas (Nam et al., 2012). The development of cystic structures invading the muscle wall resembles gastritis cystica profunda, a precancerous condition in humans characterized by herniation of gastric glands through the muscularis mucosae.

A recent study employed genetically modified mouse models, primarily Nfkb1^−/−^ mice, to investigate the progression of gastric tumorigenesis (Low et al., 2020). NF-κB1 (p50) is a critical component of the NF-κB signaling pathway that is frequently dysregulated in human gastric cancer, where it can function as either a tumor suppressor or oncogene depending on the cellular context and disease stage, making this model highly relevant for understanding the complex role of NF-κB signaling in human gastric carcinogenesis (Kravtsova-Ivantsiv et al., 2015; Yu et al., 2014). To further explore the contribution of inflammatory cytokines and signaling pathways, Nfkb1^−/−^ mice were crossbred with cytokine or receptor-deficient strains, including Il6^−/−^, Il11Ra−/−, Il22^−/−^, Tnf−/−, and Stat1^−/−^, generating compound mutants (Low et al., 2020). This comprehensive approach to dissecting inflammatory pathways mirrors the complex cytokine networks dysregulated in human gastric cancer, where multiple inflammatory mediators contribute to tumor initiation, progression, and immune evasion. Mice were aged and systematically monitored for gastric pathology over a span of up to 28 months.

In Nfkb1^−/−^ mice, gastritis and oxyntic atrophy were observed by 6 months of age. By 12 months, approximately 20% of these mice developed gastric dysplasia, with some showing evidence of invasive cancer. At 18 months, the severity of dysplasia and incidence of invasive cancer increased markedly, with roughly 50% of mice affected. By 22–28 months, the mice developed advanced invasive tumors, highlighting the progressive nature of gastric pathology in the absence of Nfkb1 (Low et al., 2020). This timeline demonstrates the tumor suppressor function of NF-κB1 in the gastric epithelium, contrasting with its oncogenic role in many other cancers, and reflects the slow progression observed in human gastric cancer where precancerous lesions may take decades to progress to invasive carcinoma (Soutto et al., 2011; O'Reilly et al., 2018).

In contrast, Nfkb1−/−Stat1−/− double knockout mice exhibited complete protection, showing no signs of gastric pathology even after 28 months (Low et al., 2020). STAT1 is a key mediator of interferon signaling and plays dual roles in cancer, functioning as a tumor suppressor through p53 activation and cell cycle arrest, but also promoting inflammation and tumor progression in certain contexts (Li X. et al., 2021; Gao et al., 2018); its complete protective effect in this model highlights the critical role of IFN-γ/STAT1 signaling in driving gastric carcinogenesis. Deletion of Tnf in Nfkb1^−/−^ mice significantly reduced the development of gastritis, atrophy, dysplasia, and invasion from 6 to 18 months, with notable suppression of gastric pathology observed through 28 months, although not to the extent seen with Stat1 deletion (Low et al., 2020). TNF-α is a key pro-inflammatory cytokine that is elevated in human gastric cancer and promotes tumor progression through multiple mechanisms including epithelial-mesenchymal transition, angiogenesis, and immune suppression (Zhou et al., 2020; Oshima et al., 2014; Lv et al., 2019); its protective deletion in this model underscores its pathogenic role in gastric carcinogenesis.

Deletion of Il6 in Nfkb1^−/−^ mice resulted in a slight reduction in dysplasia at 12 and 18 months but had no impact on the development of invasive cancer (Low et al., 2020). IL-6 is frequently elevated in human gastric cancer and activates STAT3 signaling to promote proliferation, survival, and metastasis; the modest protective effect of IL-6 deletion suggests that other cytokines may compensate for its loss in this model. In contrast, deletion of the Il11Ra led to a significant reduction in oxyntic atrophy, dysplasia, and invasion by 18 months (Low et al., 2020). IL-11 receptor alpha is increasingly recognized as an important mediator of gastric cancer progression in humans, where IL-11 signaling promotes epithelial proliferation, inflammation, and fibrosis, and represents a potential therapeutic target (Wang DQ. et al., 2016; Ma et al., 2019; Buzzelli et al., 2019). Meanwhile, deletion of Il22 slightly exacerbated gastric atrophy at 6 months but had no significant effect on dysplasia or invasion in later stages. IL-22 has complex roles in gastric pathology, as it can promote epithelial regeneration and barrier function but also drive inflammation and hyperplasia; its dual effects in this model reflect the complexity of IL-22 signaling in human gastric diseases (Lindemans et al., 2015; Keir et al., 2020; Chen et al., 2014). Overall, Nfkb1^−/−^ mice developed progressive gastric pathology, including gastritis, oxyntic atrophy, dysplasia, and invasive gastric cancer in both the gastric body (corpus) and antrum (Low et al., 2020). The pan-gastric distribution of lesions mirrors the field effect observed in human gastric cancer, where inflammatory and genetic alterations affect the entire gastric mucosa.

Immune cell infiltration played a key role in disease progression. The main inflammatory cell types included CD4^+^ and CD8^+^ T cells, regulatory T cells, B cells, CD11b^+^ myeloid cells, and neutrophils (Low et al., 2020). This diverse immune infiltrate mirrors the complex tumor microenvironment of human gastric cancer, where multiple immune cell populations contribute to either tumor suppression or promotion depending on their activation state and cytokine production profile. Notably, TNF deficiency led to reduced infiltration of T and B cells, as well as lower neutrophil counts (Low et al., 2020). Additionally, programmed death-ligand 1 (PD-L1) expression was elevated on both epithelial and myeloid cells in Nfkb1^−/−^ mice but was significantly decreased following Tnf or Stat1 deletion, indicating the involvement of TNF and STAT1 signaling in modulating the immune microenvironment during gastric tumorigenesis (Low et al., 2020). PD-L1 upregulation is a key mechanism of immune evasion in human gastric cancer and is associated with response to checkpoint inhibitor therapy (Wang et al., 2023; Chiu et al., 2018; Feng et al., 2025); its regulation by TNF and STAT1 signaling in this model provides insights into the inflammatory control of immune checkpoint expression.

The critical role of inflammation in gastric cancer development was further explored by Banerjee et al. (2014), who generated a myeloid differentiation primary response 88 gene (MyD88) knockout mouse model (Banerjee et al., 2014). MyD88 is an essential adaptor protein for most Toll-like receptors and IL-1 family receptors, and its genetic variants are associated with increased gastric cancer risk in humans, particularly in the context of *Hp* infection where bacterial recognition and inflammatory responses are critical. Following infection with *Hp*, these MyD88-deficient mice developed gastric mucosal atrophy, intestinal metaplasia, and dysplasia at both 25 and 47 weeks post-infection, thereby highlighting the importance of immune signaling in gastric carcinogenesis (Banerjee et al., 2014). This finding demonstrates that while innate immune signaling is generally protective against infection, its dysregulation can paradoxically promote carcinogenesis, reflecting the complex dual role of inflammation in human gastric cancer where chronic inflammation both combats infection and promotes malignant transformation.

In addition, Neumeyer V et al. (Neumeyer et al., 2019) explored the contribution of Wnt pathway activation to gastric precancerous conditions by introducing two point mutations (H292R and H295R) into the Ring Finger Protein 43 (RNF43) gene. RNF43 is a negative regulator of Wnt signaling that is frequently mutated in human gastric cancers, particularly in microsatellite-unstable tumors, and its loss leads to β-catenin stabilization and enhanced proliferative signaling. These mutations led to enhanced Wnt signaling (Neumeyer et al., 2019). Notably, 6 months after infection with *Hp* strain PMSS1, RNF43 mutant mice exhibited more severe gastric mucosal inflammation, atrophy, and intestinal metaplasia compared to uninfected control mice, demonstrating the synergistic effect of Wnt activation and *Hp* infection in promoting precancerous gastric changes (Neumeyer et al., 2019). This synergy between Wnt pathway activation and bacterial infection mirrors clinical observations in humans, where patients with both *Hp* infection and genetic alterations in Wnt signaling components show accelerated progression through the Correa cascade and higher gastric cancer incidence. Supplementary Table 9 summarizes signaling pathway dysregulation–induced animal models for gastric precancerous lesions.

Together, these models highlight how dysregulation of specific signaling pathways--including PPARD-mediated metabolic reprogramming, gp130/STAT3-driven inflammatory signaling, TGF-β/Smad3 tumor suppressor loss, NF-κB1 knockout-induced cytokine dysregulation and immune cell infiltration, innate immune signaling disruption through MyD88, and hyperactivation of the Wnt signaling pathway--can individually or synergistically drive the development and progression of gastric cancer *in vivo*. These findings are particularly relevant to human gastric cancer, where multiple signaling pathways are typically dysregulated simultaneously, and the models provide platforms for testing targeted therapeutic interventions that could be translated to clinical applications. However, the accelerated timelines and simplified genetic backgrounds in these models highlight the need for additional studies to fully recapitulate the complexity and heterogeneity of human gastric cancer.

#### 3.5.7 Autoimmune gastritis animal model

Autoimmune gastritis is a chronic inflammatory condition where the immune system mistakenly targets the gastric mucosa, particularly the parietal cells. In humans, autoimmune gastritis is characterized by autoantibodies against parietal cells and intrinsic factor, leading to achlorhydria, vitamin B12 deficiency, and increased risk of gastric cancer and carcinoid tumors (Neumann et al., 2013; Lenti et al., 2020; Kitamura et al., 2021). This results in a sequence of pathological changes, including atrophy, metaplasia, and dysplasia, and may increase the risk of developing gastric cancer. To explore the pathogenesis and underlying mechanisms of this disease, several animal models have been developed. These models, which include genetically modified mouse strains and mice subjected to specific experimental protocols, closely mimic human autoimmune responses. They provide valuable platforms for investigating the roles of immune cells, autoantibodies, and cytokines in driving gastric inflammation and subsequent malignant transformation.

One classic model of experimental autoimmune gastritis is established through neonatal thymectomy (NTx), whereby BALB/c nu/+ mice undergo surgical removal of the thymus at 3 days after birth (Fukuma et al., 1988). This model exploits the critical role of thymic selection in preventing autoimmunity, mimicking the breakdown of self-tolerance that occurs in human autoimmune gastritis where regulatory T cell dysfunction contributes to loss of immune tolerance to gastric antigens (Shirafkan et al., 2024; Lee et al., 2012). In the early period (one to 2 months post-NTx), chief cells are largely depleted, whereas parietal cells persist but display clear signs of damage. As the disease progresses (four to 6 months post-NTx), parietal cells are entirely lost, and compensatory hyperplasia leads to an increase in mucous neck-like cells and epithelial cells, resulting in thickening of the gastric mucosa (Fukuma et al., 1988). This progression from chief cell loss to complete parietal cell atrophy mirrors the natural history of human autoimmune gastritis, where progressive loss of specialized gastric cells leads to achlorhydria and hypergastrinemia. The gastric mucosa of NTx mice shows pronounced mononuclear cell infiltration, consisting mainly of lymphocytes and plasma cells. In the early phase, T lymphocytes—especially those expressing the thymus cell antigen 1.2 (Thy1.2)—are the predominant infiltrating cells. As the disease advances (around 6 months post-NTx), a greater presence of B lymphocytes can be observed (Fukuma et al., 1988). Plasma cells, which contribute to the humoral immune response, are consistently abundant throughout the infiltration (Fukuma et al., 1988). The progression from T cell-predominant to B cell and plasma cell-rich infiltrates reflects the evolution from cellular to humoral autoimmunity observed in human autoimmune gastritis, where anti-parietal cell and anti-intrinsic factor antibodies are key diagnostic markers (Nguyen et al., 2013; Harakal et al., 2016; Kametaka et al., 2022).

To build on this model, researchers have developed a more aggressive form of experimental autoimmune gastritis by administering polyinosinic:polycytidylic acid (poly I:C)—a synthetic analogue of double-stranded RNA that simulates viral infection—to neonatal thymectomized BALB/c mice (Kobayashi et al., 2004). This approach models the potential role of viral infections as environmental triggers in human autoimmune gastritis, where molecular mimicry between viral antigens and self-antigens may initiate autoimmune responses in genetically susceptible individuals (Maguire et al., 2024; Hussein and Rahal, 2019). After thymectomy, mice are given intraperitoneal injections of polyinosinic:polycytidylic acid at 5 mg/kg on days five, six, twelve, thirteen, nineteen, twenty, twenty-six, and twenty-seven after birth (Kobayashi et al., 2004). Polyinosinic:polycytidylic acid acts by inducing the production of proinflammatory cytokines such as interleukin-6, interleukin-12p70, interferon-gamma, and tumor necrosis factor-alpha, while also reducing the number of regulatory T lymphocytes, thereby intensifying the autoimmune reaction (Kobayashi et al., 2004). This cytokine profile closely resembles that observed in human autoimmune gastritis, where Th1-predominant responses with elevated IFN-γ and TNF-α drive epithelial damage and perpetuate inflammation (Tu et al., 2012; Iwamoto et al., 2012; Cascetta et al., 2024).

In this enhanced model, pathological changes become evident as early as 4 weeks following thymectomy and polyinosinic:polycytidylic acid treatment (Kobayashi et al., 2004). Mononuclear cell infiltration begins around the subglandular region and progresses upward within the mucosal layers (Kobayashi et al., 2004). Simultaneously, there is dramatic loss of parietal cells and increasing atrophy of gastric glands (Kobayashi et al., 2004). Hypertrophy of the gastric mucosa occurs due to proliferation of immature epithelial cells. By the fourth week post-treatment, all mice in this group develop autoantibodies against H^+^/K^+^ ATPase, a marker of parietal cell destruction (Kobayashi et al., 2004). The rapid development of anti-H^+^/K^+^ ATPase antibodies mirrors the serological progression in human disease, where these autoantibodies are present in >95% of patients with autoimmune gastritis and correlate with the degree of parietal cell loss (Tonegato et al., 2024; Herrmann et al., 2007).

To further dissect the immunological pathways contributing to autoimmune gastritis, Nishiura et al. (2012) have also studied the function of thymic stromal lymphopoietin (TSLP). TSLP is an epithelial-derived cytokine that regulates immune tolerance, and its dysregulation has been implicated in various human autoimmune diseases, making this pathway relevant to understanding autoimmune gastritis pathogenesis (Hanabuchi et al., 2012; Nakajima et al., 2020). Mice deficient in TSLP were crossed with neonatal thymectomized mice to generate neonatal thymectomized TSLP receptor–deficient mice (NTx-TSLPR^−/−^) (Nishiura et al., 2012). Compared to neonatal thymectomized TSLP receptor–sufficient controls (NTx-TSLPR^+/+^), these mice displayed significantly elevated levels of anti-parietal cell antibodies as early as 6 weeks of age, as well as an earlier and more severe onset of autoimmune gastritis marked by enhanced mononuclear cell infiltration and pronounced loss of parietal and chief cells (Nishiura et al., 2012). By 10 weeks, aggressive infiltration by CD4^+^ T lymphocytes and even greater loss of gastric gland cells were detected. By 14 weeks, autoimmune gastritis became more severe with almost complete loss of parietal and chief cells (Nishiura et al., 2012). This accelerated disease progression in TSLP-deficient mice demonstrates the critical role of epithelial-immune cell communication in maintaining gastric tolerance, which may be disrupted in human autoimmune gastritis. These CD4^+^ T lymphocytes infiltrate the gastric mucosa and produce substantial amounts of interferon-gamma, which is pivotal for disease progression (Nishiura et al., 2012). Dendritic cells play a key role in antigen presentation and in driving inflammation, and the production of the IL-12/23p40 cytokine subunit by dendritic cells was notably increased in NTx-TSLPR^−/−^ mice (Nishiura et al., 2012). Macrophages, another major type of inflammatory cell, participate in tissue destruction and amplification of local inflammation (Nishiura et al., 2012). The enhanced IL-12/23p40 production suggests activation of Th1 and Th17 pathways, which are both implicated in human autoimmune gastritis and contribute to chronic inflammation and tissue destruction (Marks et al., 2017).

In addition to models based on neonatal thymectomy, various transgenic and spontaneous mouse strains have been bred to develop experimental autoimmune gastritis. For example, T-cell receptor (TCR) transgenic mice expressing receptors specific for the alpha and beta subunits of gastric H^+^/K^+^ ATPase exhibit spontaneous autoimmune gastritis due to the abundance of autoreactive T cells (Nguyen et al., 2013). This model directly targets the same antigen (H^+^/K^+^ ATPase) that is attacked in human autoimmune gastritis, making it highly relevant for understanding the antigen-specific immune responses in human disease. Disease manifestations emerge between three and 6 months of age and feature mononuclear cell infiltration, progressive atrophy of parietal and chief cells, and chronic mucosal damage (Nguyen et al., 2013). In studies of TxA23 TCR transgenic mice: by 2 months, moderate inflammation, oxyntic atrophy, and mucosal hyperplasia/metaplasia are present, but there is little or no dysplasia; by 4 months, the inflammation and atrophy worsen, hyperplasia and metaplasia are exacerbated, and a small proportion of mice develop mild focal dysplasia; by 12 months, most mice develop severe dysplasia typical of gastric intraepithelial neoplasia, with focal pseudoinvasion into the submucosa or serosa, but not frank carcinoma (Nguyen et al., 2013). This progression from atrophy through metaplasia to dysplasia closely mirrors the natural history of human autoimmune gastritis, where patients have a 2%–3% lifetime risk of developing gastric adenocarcinoma, though carcinoid tumors are more common due to chronic hypergastrinemia (Coati et al., 2015; Jove et al., 2024). Infiltrating immune cells include CD4^+^ T lymphocytes producing both interferon-gamma and smaller amounts of interleukin-17, as well as macrophages, neutrophils, and subsets of dendritic cells (Nguyen et al., 2013). The mixed Th1/Th17 response mirrors the immune profile observed in human autoimmune gastritis, where both pathways contribute to sustained inflammation and epithelial damage (Stummvoll et al., 2008; Kamali et al., 2019).

Another transgenic approach involves the targeted overexpression of granulocyte-macrophage colony-stimulating factor (GM-CSF) in gastric parietal cells, driven by the H^+^/K^+^ ATPase alpha-subunit promoter (Field et al., 2008). GM-CSF is elevated in various human autoimmune diseases and promotes dendritic cell activation and inflammatory responses, making this model relevant for understanding cytokine-driven autoimmunity in the gastric mucosa (Shiomi and Usui, 2015; Lotfi et al., 2019). These mice rapidly develop experimental autoimmune gastritis with pronounced gastric hypertrophy, mononuclear cell infiltration, and loss of parietal and chief cells, usually becoming apparent by six to 8 weeks of age (Field et al., 2008).

Of particular interest in recent years is the investigation of the role of mucin-type O-glycosylation in maintaining gastric homeostasis and protecting against spontaneous gastritis and tumorigenesis (Liu et al., 2020). Altered mucin glycosylation is observed in human gastric cancer and precancerous lesions, where changes in O-glycan structures affect bacterial adhesion, immune recognition, and epithelial barrier function (Rossez et al., 2012; Jin et al., 2017; Freitas et al., 2019). To study this, researchers engineered mice lacking gastric epithelial O-glycans by deleting the core 1 beta1,3-galactosyltransferase 1 (C1galt1) gene in gastric epithelial cells (resulting in GEC C1galt1^−/−^ mice) (Liu et al., 2020). These mice show early features of spontaneous gastritis by 2 weeks of age, with mucosal thickening and enhanced immune cell infiltration in the antrum (Liu et al., 2020). By 8 weeks, severe chronic gastritis with marked hyperplasia and inflammation is evident, and by 6 months, the mice exhibit dysplasia in the gastric antrum (Liu et al., 2020). Strikingly, by twelve to 18 months, overt tumors and adenocarcinoma in the antrum develop in approximately 80% and 95% of these mice, respectively (Liu et al., 2020). The high penetrance of gastric cancer in this model demonstrates the critical role of proper mucin glycosylation in maintaining gastric homeostasis and preventing malignant transformation, providing insights into the glycan changes observed in human gastric cancer. The immune cell infiltrate in these models is composed of increased MPO^+^ granulocytes, F4/80^+^ macrophages, and various dendritic cells (Liu et al., 2020). Activation of the inflammasome pathway was evidenced by caspase 1 and 11 activation, implicating innate immune responses in disease progression and tumor risk (Liu et al., 2020). Inflammasome activation is increasingly recognized in human gastric cancer and autoimmune gastritis, where it contributes to chronic inflammation and tissue damage through IL-1β and IL-18 production (Zeng et al., 2023; Dawson et al., 2023; Man, 2018; Zhou et al., 2024).

Spontaneous models are also observed in certain inbred mouse strains. For instance, approximately 30% of C3H/He mice develop spontaneous autoimmune gastritis, characterized by circulating autoantibodies to parietal cells and H^+^/K^+^ ATPase, together with extensive mononuclear infiltration of the gastric mucosa (Alderuccio et al., 2002). This spontaneous model is valuable as it reflects the genetic predisposition observed in human autoimmune gastritis, where certain HLA allotypes and genetic polymorphisms increase disease susceptibility (Suarez-Trujillo et al., 2024; Lahner et al., 2015). Spontaneous gastritis in these mice typically becomes evident between six and 12 months of age (Alderuccio et al., 2002).

Another example is the SAMP1/YitFc (SAMP) mouse strain, which spontaneously develops chronic gastric inflammation that progresses to metaplasia (De Salvo et al., 2021). The SAMP strain was originally selected for senescence acceleration but develops gastric pathology that shares features with human gastric diseases, including age-related chronic inflammation and progression to metaplastic changes. At 4 weeks of age, early signs of hyperproliferation of gastric glands are observed, accompanied by the presence of eosinophils in the gastric mucosa. By 10 weeks, these mice develop chronic gastritis with increased severity, marked by the presence of interleukin-33-producing M2 macrophages (De Salvo et al., 2021). IL-33 is an alarmin that promotes type 2 immune responses and has been implicated in human gastric cancer, where it may promote tumor progression through recruitment of immunosuppressive cells (Cayrol and Girard, 2014; Eissmann et al., 2019; Yu et al., 2015). By 20 weeks, the SAMP mice exhibit advanced intestinalized SPEM with significant loss of parietal and chief cells, and increased presence of mucous neck cells (De Salvo et al., 2021). The development of intestinalized SPEM is particularly relevant as this represents a more advanced metaplastic change that may be more closely related to human intestinal metaplasia than typical murine SPEM (Lee et al., 2022; Weis et al., 2013).

The inflammatory response in SAMP mice primarily involves eosinophils and M2 macrophages. Eosinophils expand peripherally and infiltrate the gastric mucosa, while M2 macrophages are identified as significant producers of IL-33. This eosinophil-rich, type 2 immune response differs from the typical Th1-predominant pattern in classic autoimmune gastritis, suggesting that multiple immune pathways can lead to chronic gastric inflammation and highlighting the heterogeneity observed in human gastric diseases (Harakal et al., 2016; Spencer et al., 2009; Piazuelo et al., 2008). These findings highlight the dynamic interaction between immune cells and gastric tissue that contributes to disease progression in these mouse models. Supplementary Table 10 summarizes animal models of autoimmune gastritis.

In conclusion, the diverse animal models of autoimmune gastritis highlighted offer invaluable insights into the complex processes underpinning gastric inflammation and progression to malignancy. From genetically modified mice to spontaneously occurring models, these systems reveal the critical roles of immune cell interactions, cytokine production, and autoantibodies in disease manifestation and advancement. While these models successfully recapitulate many aspects of human autoimmune gastritis, important differences remain, including the timeline of disease progression, the predominant immune pathways involved, and the cancer risk profile. Nevertheless, these models have been instrumental in understanding the immunological mechanisms of gastric autoimmunity and continue to provide platforms for testing therapeutic interventions that may be relevant to human disease.

#### 3.5.8 Multigenic mutation or deletion models in specific glandular cells

Recent advances in gastric carcinogenesis research have been made by developing multigenic mutation or deletion models in specific glandular cell populations in the mouse stomach. These models represent a significant advancement in recapitulating the molecular complexity of human gastric cancer, where multiple genetic alterations typically accumulate over decades to drive progression from normal epithelium through precancerous lesions to invasive carcinoma and metastasis. Utilizing the Cre/LoxP system in combination with novel stomach-specific inducible CreERT2 mouse lines, researchers have achieved controlled gene manipulation in targeted tissues upon tamoxifen induction (Seidlitz et al., 2019). Notably, the annexin A10 (Anxa10) gene, which is specifically expressed in the stomach epithelium, was selected as the driver for CreERT2 expression, thereby allowing highly precise modeling of human gastric cancer subtypes (Seidlitz et al., 2019). ANXA10 is specifically expressed in gastric epithelial cells and is frequently downregulated in human gastric cancer, making this an appropriate driver for gastric-specific genetic manipulation (Lu et al., 2011; Ishikawa et al., 2020).

To recapitulate distinct subtypes of human gastric cancer, three primary mouse models were established. This approach is groundbreaking as it represents the first systematic attempt to model the distinct molecular subtypes of human gastric cancer identified through comprehensive genomic analyses, including TCGA classification. The first group, termed the chromosomal instability model, was generated with the genotype Anxa10-CreERT2; Kras^G12D/+^; Tp53^R172H/+^; mothers against decapentaplegic homolog 4 (Smad4)^fl/fl^ (Seidlitz et al., 2019). This combination specifically models human gastric cancers with chromosomal instability, which represents approximately 50% of human gastric adenocarcinomas and is characterized by aneuploidy, frequent copy number alterations, and mutations in TP53, KRAS, and SMAD4, typically associated with intestinal-type morphology and better prognosis than other subtypes (Comprehensive molecular characterization of gastricadenocarcinoma, 2014; Nemtsova et al., 2023; Maleki and Röcken, 2017). Following tamoxifen induction, CIN mice displayed dysplastic transformation of the gastric epithelium by 3 weeks post-induction (Seidlitz et al., 2019). This was followed by the development of early-stage (T1/T2) cancer with submucosal and muscularis propria invasion during weeks two to eight (Seidlitz et al., 2019). By eight to 10 weeks post-induction, tumors had invaded the subserosa, advancing to T3/T4 stages, and beyond 10 weeks, metastases became detectable in both lung and liver (Seidlitz et al., 2019). The progression through T-stages mirrors the TNM staging system used in human gastric cancer, and the pattern of hepatic and pulmonary metastases reflects the typical metastatic sites observed in human intestinal-type gastric cancer.

The second model, called the genomically stable–TGF-β pathway (GS-TGBF) subtype, used the genotype Anxa10-CreERT2; Cdh1^fl/fl^; Kras^G12D/+^; Smad4^fl/fl^ (Seidlitz et al., 2019). This model specifically recapitulates human diffuse-type gastric cancer, which is characterized by CDH1 loss, intact chromosomes, and frequent alterations in TGF-β pathway components, representing approximately 20% of human gastric cancers and associated with younger age at diagnosis, female predominance, and poor prognosis (Pinheiro et al., 2010; Luo et al., 2018; Nadauld et al., 2014). In these mice, T1 cancerous lesions appeared as early as 1 week post-induction, rapidly advancing to T2 cancer by weeks two to eight (Seidlitz et al., 2019). From week eight onward, advanced T3/T4 lesions with both peritoneal carcinomatosis and lung metastases were observed (Seidlitz et al., 2019). The rapid progression and peritoneal carcinomatosis accurately reflect the clinical behavior of human diffuse-type gastric cancer, which typically presents at advanced stages and has a predilection for peritoneal metastasis, leading to poor survival outcomes.

The third model, GS-Wnt, was established with Anxa10-CreERT2; Cdh1^fl/fl^; Kras^G12D/+^; Apc^fl/fl^ (Seidlitz et al., 2019). This model targets the Wnt signaling pathway, which is dysregulated in approximately 30% of human gastric cancers, particularly in the genomically stable subtype, where APC mutations and β-catenin activation drive adenomatous transformation (Toshima et al., 2023; White et al., 2012). In these mice, dysplastic changes in the epithelium became evident by 4 weeks post-induction, with early (T1a/T1b) cancers invading the lamina propria and submucosa after 4 weeks. Eventually, by week twenty-five, large tumor masses occupied the stomach lumen, leading to obstruction. However, these tumors did not exhibit metastatic behavior (Seidlitz et al., 2019). The lack of metastatic potential reflects the generally better prognosis of human gastric cancers with Wnt pathway activation, which tend to grow locally but have lower metastatic potential compared to other subtypes.

Across all three models, lesions were primarily located in the corpus and antrum regions of the stomach (Seidlitz et al., 2019). Pathologically, the CIN model closely resembled human intestinal-type gastric cancer with glandular morphology, whereas the GS-TGBF model produced poorly differentiated tumors with signet ring cells, mimicking diffuse-type gastric cancer (Seidlitz et al., 2019). These histological features precisely mirror the Lauren classification of human gastric cancer, with intestinal-type tumors showing glandular architecture and diffuse-type tumors characterized by signet ring cells and discohesive growth patterns. The GS-Wnt model gave rise to adenomatous tumors with characteristic serrated, tooth-like architectures (Seidlitz et al., 2019). The serrated architecture resembles gastric adenomas observed in humans with Wnt pathway activation and familial adenomatous polyposis (Fu et al., 2009; Hashimoto et al., 2015; McGowan et al., 2023).

Each model demonstrated specific patterns of invasiveness and metastatic potential. The CIN and GS-TGBF tumors were highly invasive: the CIN mice exhibited liver and lung metastases, while the GS-TGBF model presented with both peritoneal and lung metastases (Seidlitz et al., 2019). These metastatic patterns accurately reflect clinical observations in human gastric cancer, where intestinal-type tumors typically metastasize hematogenously to liver and lungs, while diffuse-type tumors show a predilection for peritoneal spread (Koemans et al., 2020; Díaz Del Arco et al., 2022). Although the GS-Wnt tumors did not metastasize, their size and location led to gastric outlet obstruction due to large intraluminal growths (Seidlitz et al., 2019). Gastric outlet obstruction is a recognized complication of human gastric cancer, particularly in antral tumors, and represents a significant cause of morbidity requiring palliative intervention (Keränen et al., 2013).

Another research group also investigated gastric tumorigenesis by employing a tamoxifen-inducible Pepsinogen C (Pgc)-specific CreERT2 system to introduce various combinations of oncogenic mutations, including KrasG12D, Apc^fl/fl^, and Trp53^fl/fl^, into chief cells of the stomach (Douchi et al., 2021). Pepsinogen C is specifically expressed in gastric chief cells, making this model valuable for studying the role of chief cells as potential cells-of-origin for gastric cancer, which is increasingly recognized in human disease where chief cell loss precedes metaplastic transformation (Shen et al., 2017). In these models, tamoxifen administration triggered recombination, resulting in the formation of several distinct genotypes (Douchi et al., 2021). In Pgc-CreERT2; Kras^G12D/+^ mice, pseudopyloric metaplasia appeared within 3 months, characterized by increased expression of Muc5ac, Tff2, and Pdx1, along with the depletion of parietal cells (Douchi et al., 2021). The expression of PDX1 in gastric metaplasia is particularly significant as this pancreatic transcription factor is ectopically expressed in human gastric intestinal metaplasia and may drive transdifferentiation toward an intestinal phenotype (Ma et al., 2008; Sue et al., 2016; Sousa et al., 2016). Notably, this metaplasia peaked at 6 weeks after induction before gradually subsiding (Douchi et al., 2021). The lesions were also marked by prominent infiltration of F4/80-positive macrophages, indicating an inflammatory reaction (Douchi et al., 2021). The macrophage infiltration reflects the tumor-associated macrophage accumulation observed in human gastric cancer, where these cells promote tumor progression through growth factor production, angiogenesis, and immune suppression (Wei et al., 2020; Zhang et al., 2017; Harada et al., 2018).

When Apc was inactivated together with Kras activation (Pgc-CreERT2; Kras^G12D/+^;Apc^fl/fl^), the mice developed more severe pathologies, such as intramucosal dysplasia and carcinoma at 9 months. These lesions showed increased proliferation, CD44v10 positivity, nuclear β-catenin accumulation, and persistent inflammation with abundant macrophage infiltration (Douchi et al., 2021). The nuclear β-catenin accumulation and CD44v10 expression are key markers of Wnt pathway activation and cancer stem cell properties in human gastric cancer, where they are associated with enhanced proliferation, invasion, and therapeutic resistance (Udhayakumar et al., 2011; Yong et al., 2016; Tsugawa et al., 2019). With combined deletion of Trp53 and activation of Kras (Pgc-CreERT2; Kras^G12D/+^;Trp53^fl/fl^), pseudopyloric metaplasia occurred alongside sustained macrophage infiltration starting at 3 months (Douchi et al., 2021). The most aggressive disease was observed when all three genetic alterations were present (Pgc-CreERT2; Kras^G12D/+^;Apc^fl/fl^;Trp53^fl/fl^), as these mice consistently developed invasive and metastatic gastric carcinoma by 9 months (Douchi et al., 2021). This triple-hit combination reflects the multi-step carcinogenesis model in human gastric cancer, where accumulation of multiple genetic alterations drives progression from benign epithelium to invasive carcinoma. Tumors in this group invaded muscle layers, liver, lymph nodes, and diaphragm and were associated with severe inflammation and extensive macrophage infiltration (Douchi et al., 2021). The metastatic pattern including lymph node involvement accurately reflects human gastric cancer progression, where lymph node metastasis is a critical prognostic factor and determines treatment strategies.

Extending this strategy, researchers have employed the Cldn18-IRES-CreERT2 mouse model to direct genetic recombination specifically within the gastric epithelium (Fatehullah et al., 2021). As discussed previously, Cldn18 is specifically expressed in gastric epithelial cells and represents a novel therapeutic target in human gastric cancer, making this an appropriate driver for gastric-specific genetic manipulation (Tojjari et al., 2024). By crossing Cldn18-IRES-CreERT2 mice with conditional Apc^fl/fl^, KrasG12D, and Trp53^fl/fl^ strains, they developed the Cldn18-ATK model, which simultaneously activates Wnt and Kras signaling and inactivates p53 in gastric epithelial cells (Fatehullah et al., 2021). This combination targets three of the most frequently altered pathways in human gastric cancer and represents the convergence of multiple oncogenic mechanisms. Upon tamoxifen induction, these mice exhibited early “antralization” of the corpus gland mucosa, evidenced by increased expression of Muc5ac and Tff2 as early as 1 week post-induction (Fatehullah et al., 2021). By 1 month, multifocal neoplasia, including T1 gastric carcinoma, was observed. The disease rapidly advanced; within 2 months, many mice developed T3 gastric carcinomas with serosal invasion. In final stages, aggressive T3–T4 carcinomas extended into the serosa, mesentery, and pancreas, frequently accompanied by metastases to the liver and lungs (Fatehullah et al., 2021). The involvement of adjacent organs including the pancreas reflects the locally advanced disease observed in human gastric cancer, where direct extension to surrounding structures significantly impacts staging and prognosis. Notably, while polymorphonuclear cell levels remained unaltered throughout disease progression, a marked increase in tumor-associated macrophages was evident, underscoring the critical contribution of the myeloid microenvironment in the progression of gastric cancer (Fatehullah et al., 2021). The selective increase in tumor-associated macrophages without neutrophil expansion suggests a shift toward an immunosuppressive M2-like phenotype that characterizes the tumor microenvironment in human gastric cancer.

Similarly, Li et al. (2016) focused on the inactivation of Smad4 and phosphatase and tensin homolog (PTEN) specifically in Lgr5^+^ stem cells, which are abundant within the gastric epithelium. Lgr5^+^ cells are well-characterized stem cells in the gastrointestinal tract, and their role in gastric cancer is of particular interest as stem cell dysfunction is thought to be a key initiating event in human carcinogenesis (Leushacke et al., 2017; Jang et al., 2013; Syu et al., 2016). Their findings demonstrated that disruption of these genes in Lgr5^+^ gastric antrum stem cells promoted rapid adenoma formation, which subsequently progressed to invasive carcinoma *in situ*. Some of the resulting tumors penetrated the muscularis layer, with lesions primarily arising in the gastric antrum and antral-body junction (Li et al., 2016). The antral predominance reflects the anatomical distribution of many human gastric cancers, particularly those associated with *Hp* infection and intestinal metaplasia, which typically begin in the antrum before extending to the corpus.

Fang et al. (2023) reported that the combined deletion of serine/threonine kinase 11 (STK11, also known as LKB1) and PTEN in parietal cells resulted in the formation of hyperplastic gastric polyps after about 20 weeks. STK11/LKB1 is a tumor suppressor that regulates the mTOR pathway and is associated with Peutz-Jeghers syndrome in humans, where gastric polyps and increased cancer risk are observed (Altamish et al., 2020; Li R. et al., 2018; Resta et al., 2013). Over the following 20 weeks, these polyps underwent malignant transformation, developing into adenocarcinomas that ultimately led to death (Fang et al., 2023). Lesions primarily developed in the gastric body and antrum, and, as disease advanced, tumors frequently invaded the duodenum (Fang et al., 2023). The progression from hyperplastic polyps to adenocarcinoma mirrors the adenoma-carcinoma sequence observed in human gastric cancer, while duodenal invasion reflects the locally aggressive behavior of advanced gastric cancers. Supplementary Table 11 summarizes glandular cell–targeted multigenic models used to study gastric precancerous lesions.

Collectively, these sophisticated mouse models clearly demonstrate that the introduction of defined genetic alterations in specific gastric epithelial cell populations can faithfully recapitulate the full spectrum of human gastric carcinogenesis--from early metaplasia and dysplasia to invasive carcinoma and distant metastasis. These models represent a major advancement in gastric cancer research as they successfully capture the molecular heterogeneity, histological diversity, and clinical behavior of human gastric cancer subtypes. Moreover, these studies emphasize the essential role of the tumor microenvironment, particularly inflammatory cells such as macrophages, in modulating and promoting cancer progression within the genetically altered gastric mucosa. The ability to model specific human gastric cancer subtypes with their characteristic genetic alterations, histological features, and metastatic patterns provides unprecedented opportunities for studying disease mechanisms, identifying therapeutic targets, and testing novel treatment strategies that may be directly translatable to human patients.

## 4 Conclusion

This comprehensive review underscores the substantial contributions that diverse animal models have made to our understanding of gastric precancerous lesions and their progression toward gastric cancer. The range of models discussed—from *Helicobacter* infection and chemical carcinogens to genetically engineered mice—together illuminate the complex, multifactorial nature of gastric carcinogenesis (Supplementary Tables 1–11). While each model presents unique insights, it is important to recognize that they also bring distinct limitations that must be carefully considered during both experimental design and data interpretation.

Developing a simple, controllable, and stable animal model of gastric precancerous lesions is fundamental for unraveling the mechanisms that drive gastric cancer development and for conducting research on secondary prevention interventions. Among the various models, those induced by *Helicobacter* infection, particularly with *Hf* in C57BL/6 mice, most closely recapitulate the Correa cascade observed in humans, progressing from chronic gastritis through metaplasia to dysplasia. However, it is important to note that these models primarily produce SPEM rather than true intestinal metaplasia, which limits their translational relevance. In contrast, chemical carcinogen-induced models—such as those using MNU or MNNG—reliably yield tumors but often bypass the stepwise progression evident in human disease. Meanwhile, genetically engineered mouse models allow for precise mechanistic investigation of specific molecular pathways but may not fully reproduce the environmental and microbial influences pivotal to human gastric carcinogenesis.

Recently, advances in genetic engineering have enabled the development of increasingly sophisticated animal models. By editing individual or multiple genes at the molecular level, most gene-edited mouse models are able to recapitulate the full spectrum of pathological stages described in the Correa model of gastric carcinogenesis. These models typically exhibit high-risk precancerous lesions involving both the gastric antrum and body, as well as demonstrating features such as tumor vascular invasion and distant metastasis—thereby more closely mirroring the progression of human gastric cancer. As a result, gene-edited models are expected to become mainstream tools for investigating gastric cancer precancerous lesions and tumorigenesis itself.

However, a significant limitation of systemic gene-editing approaches is the frequent occurrence of extra-gastric lesions. These off-target effects can lead to premature death and thus hinder comprehensive observation of gastric pathological progression. To overcome this challenge, it is recommended to utilize gene-edited mouse models that specifically target gastric epithelial cells in either the body or antrum of the stomach, such as parietal cells, chief cells, or isthmus stem cells. Recent innovations in stomach-specific, inducible Cre recombinase systems—including Anxa10-CreERT2, Tff1-CreERT2, and Mist1-CreERT2—now allow for precise temporal and spatial control of gene modifications within specific gastric epithelial cell populations. When these targeted systems are combined with mutations in major oncogenes and tumor suppressor genes (such as Kras, Cdh1, Trp53, and Smad4), the resulting mouse models can faithfully reproduce a range of human gastric cancer subtypes, including both intestinal and diffuse types, with features of metastatic disease.

Moreover, combining these genetically engineered models with *Helicobacter* infection or chemical carcinogen exposure can provide more comprehensive insights into the early cellular and molecular events involved in neoplastic transformation, as well as help clarify the cellular origins of dysplasia and cancer. This integrated approach offers the potential to more accurately recapitulate the process of neoplastic transformation in the human gastric epithelium and to identify those precancerous lesions most likely to undergo malignant transformation. Ultimately, such advances will not only deepen our understanding of the mechanisms underlying gastric cancer development but will also provide a strong foundation for pharmacological studies and further mechanistic investigations.

Despite these advances, several challenges remain. Anatomical and physiological differences between rodent and human stomachs complicate direct translation of findings. Moreover, the distinction between reactive metaplasia and true precancerous lesions is still not fully established, as demonstrated by the potential reversibility of SPEM in certain contexts. Standardization of histopathological evaluation methodologies, such as those provided by the “Histologic Scoring of Gastritis and Gastric Cancer in Mouse Models” system, represents a critical step towards achieving more reliable and comparable research outcomes.

Several research directions merit further exploration. The integration of advanced technologies—such as single-cell RNA sequencing, spatial transcriptomics, and proteomics—with existing animal models promises unprecedented insights into the cellular heterogeneity and molecular mechanisms of gastric precancerous lesions. Furthermore, the development of organoid models derived from both normal and precancerous gastric tissues offers complementary approaches that may help to bridge the gap between animal models and human disease. Additionally, continued investigation into the complex interactions among genetic predisposition, *Helicobacter* infection, dietary factors, and the gastric microbiome will be essential for a more comprehensive understanding of environmental influences in gastric carcinogenesis.

Ultimately, the ideal animal model should recapitulate the full spectrum of human gastric precancerous lesions with respect to histopathology, molecular features, and clinical behavior. Although achieving this objective remains a significant challenge, the ongoing refinement of current models and development of innovative approaches will undoubtedly enhance our ability to investigate preventive strategies, identify early diagnostic biomarkers, and develop targeted interventions for gastric cancer. Such advancements are especially critical given the persistent global burden of gastric cancer, particularly in high-incidence regions such as East Asia, where early interventions at the precancerous stage could markedly reduce mortality.
