# Supplementary material for: Precancerous pathways to gastric cancer: a review of experimental animal models recapitulating the correa cascade
Source: Front Cell Dev Biol. 2025 Jul 2;13:1620756. doi: 10.3389/fcell.2025.1620756 (PMC12263962; doi:10.3389/fcell.2025.1620756)
Supplement: Supplementary file 1 [file Table1.docx]

**Table 1:** Summary of *Helicobacter* Infection–Induced Animal Models for Gastric Precancerous Lesions

| Studies | Animal Model | Modeling Method and Principle | Feeding Conditions | Model Evaluation Methods | Advantages and Disadvantages | Detailed Time Course of Pathological Stages | Anatomical Sites of Lesions | Tumor Location and Invasion/Metastasis | Inflammation and Immune Cell Infiltration | Application Scenarios for Models |
| --- | --- | --- | --- | --- | --- | --- | --- | --- | --- | --- |
| Ref. (1) | Specific pathogen–free male Mongolian gerbils (*Meriones unguiculatus*, strain MGS/Sea), 5 weeks old, obtained from Seiwa Experimental Animals (Fukuoka, Japan) | Animals were orally inoculated with 1 mL of broth culture containing 10^7.54^ CFU of *H. pylori* strain TN2GF4 (cagA^+^, VacA^+^, urease^+^, catalase^+^, oxidase^+^). The model induces chronic active gastritis, ulcers, and intestinal metaplasia, followed by adenocarcinoma over a period of 62 weeks, thereby mimicking multistep human gastric carcinogenesis | - Temperature: 23 ± 2°C - Humidity: 55 ± 5% - Light/dark cycle: 12/12 h - Diet: CE-2 commercial rodent chow (Clea Japan) - Water: Tap water, ad libitum | - Microbiological analysis: Quantification of H. pylori colonization via culture (CFU per gastric wall). - Histopathology: Tissues stained with H&E; AB-HID (for mucins); Grimelius and Sevier–Munger (for neuroendocrine cells). - Immunohistochemistry: p53 protein detection using monoclonal antibody. | - Advantages: Reproduces full carcinogenic cascade from infection to adenocarcinoma; mimics human intestinal-type gastric cancer. - Disadvantages: Tumors limited to pyloric region; no observed metastasis; only male gerbils used. | - Week 6: 100% had active chronic gastritis; 100% had regenerative hyperplasia. - Week 26: 100% had ulcers and invaginated glands; 60% had intestinal metaplasia. - Week 39: 80% had intestinal metaplasia; cystic dilations observed. - Week 52: 100% had intestinal metaplasia; 60% had hyperplastic polyps. - Week 62: 85% had intestinal metaplasia; 37% developed adenocarcinoma; 11% had carcinoids. | - Lesions originated in the pyloric antrum and extended to the fundic (body) region. - Pathological changes occurred in both pyloric and fundic regions. | - All adenocarcinomas were found in the pyloric region. Tumors arose from intestinal metaplasia and invaded the muscular layer, occasionally reaching the serosa. - No vascular invasion or distant metastasis was observed. | Infiltration Severe active chronic gastritis was consistently observed starting from 6 weeks post-infection. Infiltration involved:  - Neutrophils and mononuclear cells (dense infiltration in mucosa and submucosa)  - Multiple lymphoid follicles  - Macrophages are indirectly implicated: *H. pylori* upregulates nitric oxide synthase gene expression in macrophages, leading to nitric oxide production, which is associated with p53 overexpression in 40% of tumors. | - Mongolian gerbils are ideal for long-term studies on Helicobacter-induced multistage gastric carcinogenesis, including progression from gastritis to adenocarcinoma. - Their susceptibility to developing intestinal-type gastric cancer makes them a superior *in vivo* model for testing carcinogenesis mechanisms, chemoprevention, and p53-related tumor suppressor pathways. |
| Ref. (2) | Wild-type (C57BL/6) mice and group II secretory phospholipase A2–deficient (sPLA2-KO) mice were used. All mice were on the C57BL/6 background | - Mice were orally inoculated three times at 2-day intervals with 5 × 10⁸ colony-forming units (CFU) of *Helicobacter felis* (strain CS1). - The model induces gastric inflammation, increased epithelial cell apoptosis, and alterations in cellular differentiation, particularly under conditions of sPLA2 deficiency, which is hypothesized to modulate epithelial homeostasis during chronic infection. | Mice were kept under specific pathogen–free conditions, but detailed housing parameters such as temperature or humidity were not explicitly stated. Standard laboratory chow and water were provided ad libitum. | - Histology: Formalin-fixed, paraffin-embedded stomach sections stained with hematoxylin-eosin (H&E). - Apoptosis: Assessed via TUNEL assay (In Situ Cell Death Detection). - Proliferation: Ki-67 immunohistochemistry. - Differentiation: Alcian blue staining and GSII lectin histochemistry for mucous cell lineage. | - Advantages: Enables mechanistic study of host epithelial responses (apoptosis/differentiation) in sPLA2-deficient background. - Disadvantages: Study duration limited to 4–8 weeks; no neoplastic progression evaluated. | - Week 4 post-infection: Chronic gastritis and significant increase in apoptotic cells in sPLA2-deficient mice. - Week 8 post-infection: Further increase in apoptosis and significant decrease in surface mucous cell differentiation in knockout mice. - No later time points or carcinoma observed due to limited duration. | Lesions were predominantly observed in the corpus (gastric body). Histological evaluations and cellular markers (e.g., GSII for mucous cells) focused on corpus epithelial changes. | - No tumors, invasion, or metastasis were reported within the study duration (up to 8 weeks). - The study was focused on early epithelial alterations rather than tumor development. | - Chronic gastritis was noted with infiltration of mononuclear cells. - Although specific immune cells (e.g., macrophages, neutrophils) were not quantified or immunolabeled in this study, inflammatory cell presence in the corpus mucosa was confirmed histologically. - The paper emphasized epithelial response (apoptosis and differentiation) rather than characterizing immune cell subtypes. | - sPLA2-KO mice: Optimal for research on epithelial defense mechanisms, apoptosis signaling, lipid-mediated host-microbe interaction, and gastric mucosal differentiation. - Wild-type C57BL/6 mice: Serve as baseline controls in infection models; appropriate for studying typical mucosal responses, gastric epithelial cell turnover, and moderate chronic gastritis progression. |
| Ref. (3) | - C57BL/6 wild-type (WT) mice - p53^⁻/⁻^ knockout mice, bred and maintained on a C57BL/6 background | - Infection with *Helicobacter felis* (CS1 strain, 5 × 10⁸ CFU, via oral gavage, 3 times) to induce chronic gastric inflammation and carcinogenic transformation. - WT mice developed invasive gastric adenocarcinoma; p53⁻/⁻ mice did not, due to suppressed Th1 inflammation. | Animals housed under specific pathogen–free (SPF) conditions; provided standard chow and water ad libitum. Environmental controls (e.g., temperature, humidity) not explicitly detailed. | - Histology (H&E): to assess inflammation and tumor development - Immunohistochemistry: CD3 (T cells), F4/80 (macrophages), IFN-γ, IL-1β, iNOS, p53 - qRT-PCR & ELISA: cytokine expression (Th1 profile: IFN-γ, IL-1β) | - Advantages: Dissects role of Th1 immunity and p53 in carcinogenesis - Disadvantages: Does not simulate sporadic mutations; p53^⁻/⁻^ mice are not tumor-prone in this setting | - 6 months: WT mice developed high-grade dysplasia and invasive carcinoma; p53^⁻/⁻^ mice showed minimal inflammation and no dysplasia - >12 months: Advanced adenocarcinoma in ~30% of WT mice; no neoplasia in p53^⁻/⁻^ mice | Pathological changes observed in both the antrum and corpus, with more severe transformation near the gastric–antral junction. | - WT mice: Tumors showed muscularis invasion, glandular transformation, and high-grade dysplasia; no distant metastasis reported - p53^⁻/⁻^ mice: No evidence of tumorigenesis | - WT mice: Infiltration by CD3⁺ T cells, F4/80⁺ macrophages, and strong iNOS expression - p53^⁻/⁻^ mice: Marked reduction in Th1 cytokines (IFN-γ, IL-1β), decreased macrophage recruitment, and suppressed inflammation | - WT C57BL/6 mice: Suitable for modeling Th1-driven gastric carcinogenesis, especially in *Helicobacter*-associated inflammation-to-cancer progression. Ideal for evaluating immune-modulatory treatments and precancerous lesion progression. - p53^⁻/⁻^ mice: Useful to dissect the immune-suppressive role of tumor suppressor pathways, especially for research on immune evasion, macrophage recruitment, Th1/Th2 balance, and non-neoplastic inflammation resolution. They help distinguish immune-mediated from mutation-driven pathways of carcinogenesis. |
| Ref. (4) | - C57BL/6 mice, both wild-type and IL-17-deficient (IL-17^⁻/⁻^) strains were used. - All mice were bred in specific pathogen–free (SPF) facilities. | - Mice were orally inoculated with H. pylori strain SS1 (cagA⁻/vacA⁻). In WT mice, this induced both Th1 and Th17 responses. In IL-17^⁻/⁻^ mice, loss of IL-17A skewed the immune response, enhancing Th1 immunity and bacterial clearance, but reduced inflammation. - The model explores how Th17 modulates Th1 and disease outcome. | - Animals were maintained under SPF conditions with autoclaved chow and water ad libitum. - Housing conditions were not further detailed. | - CFU quantification of *H. pylori* in the stomach - Histology (H&E) for inflammation scoring - qRT-PCR & ELISA for IFN-γ, IL-1β, IL-17A, etc. - Immunostaining for CD4⁺ T cells | - Advantages: Dissects cytokine-specific roles; allows analysis of bacterial load and immune crosstalk - Disadvantages: No long-term carcinogenesis follow-up; SS1 strain lacks key virulence factors | - 6 weeks post-infection: WT mice had high *H. pylori* loads, moderate chronic gastritis, high IL-17A expression - IL-17^⁻/⁻^ mice: Reduced colonization, increased IFN-γ, lower inflammation scores | Histological inflammation occurred in both antrum and corpus, with diffuse involvement but no mention of region-specific preference. | No tumors, no invasive lesions, and no metastasis were observed in either group within the experimental timeframe. | - WT mice: Infiltration of CD4⁺ T cells, elevated IL-17A, IFN-γ, and IL-1β - IL-17^⁻/⁻^ mice: Lower inflammation despite enhanced Th1 response; macrophages not specifically quantified | - Wild-type C57BL/6 mice: Useful for studying normal Th1/Th17 interplay, bacterial persistence, and cytokine-induced pathology. - IL-17^⁻/⁻^ mice: Ideal for dissecting the role of Th17 in immune modulation, especially in immune evasion and bacterial load control. |
| Ref. (5) | C57BL/6 mice (specific age and supplier not specified); male mice used in all experiments | Mice were orally inoculated with *Helicobacter felis* (strain CS1) for long-term colonization. Evaluation periods extended to 6, 12, and 18 months. The model was designed to mimic chronic *Helicobacter*-induced gastric inflammation and histopathological progression. | Mice were housed under specific pathogen–free (SPF) conditions and fed standard chow and water ad libitum. Environmental specifics (temperature, humidity) were not detailed. | - H&E histology: evaluated chronic inflammation and glandular architecture - Warthin–Starry silver stain: assessed *H. felis* colonization - Inflammatory grading: based on the updated Sydney classification (activity, inflammation, glandular atrophy, metaplasia) | - Advantages: Mimics natural history of chronic infection; allows analysis of long-term effects - Disadvantages: No cancer progression observed within 18 months | - 6 months: Mild to moderate chronic inflammation and glandular hyperplasia - 12 months: Increased inflammatory cell infiltration and glandular atrophy - 18 months: Marked inflammatory cell accumulation and intestinal metaplasia in some mice. No dysplasia or carcinoma noted. | - Lesions were observed primarily in the gastric corpus, especially along the lesser curvature. - The antrum was less affected. | No tumors, no invasion, and no metastasis were observed throughout the 18-month infection period. | - Chronic inflammation characterized by infiltration of mononuclear cells, primarily lymphocytes and macrophages. - Severity of inflammation increased with infection duration. Inflammatory activity was positively correlated with colonization density. | - C57BL/6 mice with long-term *H. felis* colonization are suitable for studying the chronic phase of *Helicobacter*-induced gastritis, glandular remodeling, atrophy, and precancerous changes such as intestinal metaplasia. - This model is ideal for evaluating anti-inflammatory agents, host response modifiers, and bacterial persistence mechanisms. |
| Ref. (6) | - C57BL/6 - BALB/c - C3H/HeN - SJL - DBA/2 - A/J   All mice were inbred strains, SPF-housed, 4–6 weeks old, infected with *H. pylori* strain SS1. | - Oral inoculation with *H. pylori* Sydney strain 1 (SS1) three times within 1 week. - Purpose: test whether gastric atrophy is due to bacterial virulence or host genotype. - Findings: Only B6, SJL, and C3H/HeN developed corpus atrophy → lesion development is host dependent, not due to SS1 strain variability. | - Autoclaved NIH 31 rodent chow and water ad libitum. - -Housed in SPF facility at Tufts University. - Cage rotation and environmental controls maintained. | - H&E staining: assess glandular architecture and parietal cell loss - Warthin–Starry stain: detect *H. pylori* bacteria - Colony-forming units (CFUs): assess gastric colonization | - Advantages:   - Multi-strain comparative design  - Host-dependent insights into corpus-specific atrophy   - Disadvantages:   - No cytokine or T-cell marker analysis  - No tumor progression observed | - 1 month: Mild gastritis in all strains; corpus colonization confirmed - 3 months:   - C57BL/6, SJL, C3H/HeN: parietal cell loss, oxyntic atrophy  - BALB/c, A/J, DBA/2: no atrophy, only mild inflammation   - 6 months: Established glandular atrophy in B6, SJL, and C3H - 12 months: Chronic corpus inflammation and atrophy maintained in these three strains | - Lesions were region-specific:   - Corpus: B6, SJL, and C3H/HeN developed parietal cell loss and atrophy  - Antrum: All six strains had minimal inflammation only, not related to atrophy  → Confirmed that atrophic changes are restricted to the corpus and not linked to antral gastritis. | - No tumors, no dysplasia, no invasion or metastasis observed in any strain at 12 months. - Study model is limited to precancerous atrophic progression, not neoplastic transformation. | - C57BL/6, SJL, C3H/HeN: chronic corpus mononuclear infiltrate - Cell types include lymphocytes, plasma cells, and likely macrophages (not further specified) - BALB/c, A/J, DBA/2: only minimal superficial inflammation, primarily lymphocytic - Inflammation confined to lamina propria, parietal cell layer disrupted | - C57BL/6, SJL, C3H/HeN: Best for studying parietal cell loss, immune-driven corpus atrophy, and strain-dependent host response to *H. pylori* - BALB/c, DBA/2, A/J: Serve as immune-tolerant controls, useful for analyzing colonization without atrophy - Excellent model for genetic susceptibility studies of precancerous gastric lesions, but not suitable for gastric cancer modeling due to absence of neoplasia |
| Ref. (7) | - IL-10 knockout (IL-10^⁻/⁻^) mice, C57BL/6 background - Wild-type (WT) C57BL/6 mice as control group - Age at infection: 6 weeks | - Oral gavage with *Helicobacter felis* (CS1 strain) at 10⁸ CFU/mL, 0.2 mL per dose, three times over 1 week - Aim: examine impact of loss of IL-10-mediated immunosuppression on gastric pathology - Finding: IL-10 deficiency led to rapid, severe, proliferative gastritis, epithelial hyperplasia, and dedifferentiation. WT mice developed only mild gastritis. | - Standard laboratory chow and water ad libitum - Housed in SPF conditions throughout the study | - Histopathology (H&E): grading inflammation, glandular hyperplasia, dysplasia - Immunohistochemistry for PCNA (proliferation), cytokeratins (epithelial markers) - Silver staining for *H. felis* detection | - Advantages:   - Strong correlation between IL-10 loss and epithelial disruption  - Rapid progression, useful for studying early carcinogenic events   - Disadvantages:   - Model lacks invasive cancer or metastasis  - No molecular profiling of immune signaling | - 1 month: IL-10^⁻/⁻^: Moderate inflammation in corpus; WT: minimal - 3 months:   - IL-10^⁻/⁻^: Marked foveolar hyperplasia, loss of parietal cells, mucous metaplasia, and glandular elongation  - Early signs of epithelial dedifferentiation   - 6 months: Severe hyperplastic gastritis, intestinal metaplasia-like changes, but no neoplasia observed | - Lesions mainly in the corpus (fundic mucosa) - Inflammatory cell infiltration, glandular distortion, and parietal cell loss occurred in corpus only - Antrum was relatively unaffected | - No tumor formation, no invasion or metastasis noted up to 6 months - Precancerous epithelial dedifferentiation and marked hyperplasia suggest potential for progression, but carcinoma was not reached | - Massive infiltration in IL-10^⁻/⁻^ mice:   - Lymphocytes (dominant)  - Plasma cells  - Macrophages   - Inflammatory response localized to lamina propria; crypt destruction and expansion seen - In WT mice: limited lymphocytic infiltration | - IL-10^⁻/⁻^ mice: Excellent for studying immune dysregulation-driven gastric hyperplasia, epithelial transformation, and proliferation under chronic inflammation - Useful for testing anti-inflammatory or immunomodulatory therapies targeting IL-10 pathways - WT C57BL/6: Comparative model for resistance to epithelial dedifferentiation |
| Ref. (8) | - C57BL/6 background mice, genetically modified:   - NF-κB1^⁻/⁻^  - NF-κB2^⁻/⁻^  - c-Rel^⁻/⁻^  - Wild-type C57BL/6 as control | - Mice infected via oral gavage with *Helicobacter felis* (ATCC 49179) at 10⁸ CFU, three times in 1 week - Goal: evaluate how deletion of specific NF-κB subunits affects inflammation and progression to gastric neoplasia - Found that NF-κB1^⁻/⁻^ mice developed high-grade dysplasia and carcinoma, while NF-κB2^⁻/⁻^ and c-Rel^⁻/⁻^ showed significantly less progression | - Standard chow and water ad libitum - Maintained in specific-pathogen-free (SPF) conditions throughout the study | - Histopathology (H&E): inflammation, dysplasia, carcinoma grading - Immunohistochemistry: Ki-67 (proliferation), F4/80 (macrophages), CD4/CD8 T-cells - Gene expression analysis for cytokines and chemokines (qPCR) | - Advantages:   - Dissects specific immune signaling pathways in gastric cancer  - NF-κB1^⁻/⁻^ mice mimic human gastric neoplasia progression   - Disadvantages:   - Tumor progression limited to NF-κB1^⁻/⁻^ only  - Immune profiling limited to mRNA | - 6 months:   - WT & NF-κB2^⁻/⁻^: chronic gastritis, mild glandular hyperplasia  - NF-κB1^⁻/⁻^: intestinal metaplasia, severe dysplasia   - 12 months:   - NF-κB1^⁻/⁻^: invasive adenocarcinoma in the gastric corpus  - NF-κB2^⁻/⁻^ and c-Rel^⁻/⁻^: low-grade hyperplasia, no carcinoma   - Only NF-κB1^⁻/⁻^ progressed to cancer | - Major lesions occurred in the gastric corpus, especially fundic glands - Antrum had minimal involvement in all genotypes - Tumorigenesis was corpus-restricted | - NF-κB1^⁻/⁻^ mice developed adenocarcinoma with local invasion - No distant metastasis reported - NF-κB2^⁻/⁻^ and c-Rel^⁻/⁻^ mice did not develop tumors within 12 months | - All infected mice had gastric corpus inflammation, but severity differed:   - NF-κB1^⁻/⁻^: increased macrophages (F4/80⁺), neutrophils, and CD4⁺/CD8⁺ T-cells  - WT and other KO strains: moderate infiltration   - Cytokine expression (e.g., IL-1β, TNF-α, IFN-γ) was highest in NF-κB1⁻/⁻ mice | - NF-κB1^⁻/⁻^: ideal for modeling inflammation-driven gastric cancer, host immune pathway dysregulation, and testing NF-κB-targeted interventions - NF-κB2^⁻/⁻^ and c-Rel^⁻/⁻^: useful for understanding tumor suppression mechanisms, immune homeostasis, and as comparative controls - Model applicable for studying the role of chronic inflammation in gastric carcinogenesis |
| Ref. (9) | - C57BL/6 mice, 6 weeks old - Divided into groups inoculated with *H. pylori* (PMSS1) or *H. felis* | - Intragastric inoculation with *H. pylori* PMSS1 or *H. felis* CS1 at 10⁸ CFU/mL, 3 times within 1 week - Purpose: compare colonization efficiency, inflammation, and metaplasia between two bacterial species - Notably, *H. felis* induced more severe pathology, while *H. pylori* colonized poorly with mild changes | - Standard lab chow and water ad libitum - Maintained in SPF conditions, temperature and humidity controlled | - Histopathology (H&E): inflammation and metaplasia - Immunohistochemistry: TFF2, GSII for SPEM detection - qPCR: cytokine (IL-1β, TNF-α) expression - Colonization assays: bacterial load CFU/gram tissue | - Advantages:   - Side-by-side comparison of H. pylori vs H. felis effects  - Use of molecular and histologic markers of metaplasia   - Disadvantages:   - No neoplasia or invasive cancer  - Short-term observation (3 months) | - 1 month:   - *H. felis*: moderate inflammation, early mucosal hyperplasia  - *H. pylori*: minimal inflammation   - 3 months:   - *H. felis*: prominent inflammation, spasmolytic polypeptide-expressing metaplasia (SPEM), parietal cell loss  - *H. pylori*: still limited colonization, mild hyperplasia only | - Lesions occurred primarily in the gastric corpus for both strains - Antrum was relatively spared in both infection types | - No tumors, dysplasia, or metastasis were observed - Study focused on early metaplastic changes | - *H. felis* induced dense mononuclear infiltrates, especially in corpus:   - Macrophages (F4/80⁺)  - T cells (CD3⁺)  - Neutrophils also present   - Increased cytokines: IL-1β, TNF-α - *H. pylori* group had minimal immune infiltration | - *H. felis* in C57BL/6: ideal for modeling early inflammatory and metaplastic gastric changes, including SPEM and parietal cell loss - *H. pylori* in C57BL/6: represents low-colonization, low-pathology model, useful for studying bacterial adaptation or host resistance |
| Ref. (10) | - INS-GAS mice (gastrin-overexpressing transgenic mice on FVB/N background) - Known to be highly susceptible to *H. pylori*-induced gastric neoplasia | - Intragastric inoculation with *H. pylori* strain SS1 - Mice monitored for 6 to 12 months post-infection - Study goal: identify intestinal metaplasia and its molecular features as a precursor to gastric cancer | - Sterile rodent chow and water ad libitum - Maintained under SPF conditions throughout the experiment | - Histopathology (H&E): metaplasia, dysplasia, carcinoma grading - Alcian blue staining: identify intestinal metaplasia - qPCR & microarray: gene expression profiling (e.g., CDX2, MUC2) - Immunohistochemistry: marker validation (TFF3, Villin) | - Advantages:   - Direct observation of IM and gastric cancer in same model  - Genetic insight into human-like metaplastic transition   - Disadvantages:   - Not generalizable to wild-type mice  - Complex transgenic background | - 6 months post-infection:   - Development of chronic atrophic gastritis and early IM   - 12 months post-infection:   - Progression to IM with dysplasia and invasive adenocarcinoma in corpus | - Predominantly in the gastric corpus - Some cases involved corpus-antrum transition zone, but IM and carcinoma were corpus-dominant | - Tumors located in gastric corpus - Evidence of invasive carcinoma with glandular disruption - No distant metastasis reported | - Prominent mononuclear infiltration:   - T cells, macrophages, and plasma cells   - Inflammatory cytokines upregulated: IL-1β, IFN-γ, TNF-α - Inflammation preceded and persisted during metaplastic transformation | - INS-GAS mice + *H. pylori*: ideal for modeling full spectrum of gastric carcinogenesis including IM, dysplasia, and carcinoma - Suitable for gene expression studies, metaplastic marker validation, and testing anti-metaplasia therapies - Especially relevant to human-like progression of gastric cancer via intestinal-type pathway |
| Ref. (11) | - C57BL/6, BALB/c, CBA/Ca, and DBA/2 inbred mice - C57BL/6 and BALB/c showed chronic colonization and pathology; CBA/Ca and DBA/2 were less susceptible | - Inoculation with *H. pylori* Sydney Strain 1 (SS1), a mouse-adapted strain - 3 oral gavages (10⁹ CFU in 0.2 mL) over 1 week - Purpose: create a reproducible model for studying chronic *H. pylori* colonization and gastritis | - Standard laboratory chow and water ad libitum - Maintained in SPF animal facility | - Histology (H&E): evaluation of gastritis and gland changes - Quantitative culture: colonization density (CFU/g tissue) - ELISA: serum IgG titers | - Advantages:   - High reproducibility, long-term colonization  - Valid for immunological and vaccine studies   - Disadvantages:   - No cancer or metaplasia developed even after 15 months  - Lesions limited to inflammation without neoplastic transformation | - 3 months: moderate gastritis in C57BL/6 and BALB/c - 6–12 months: sustained chronic inflammation without neoplasia - 15 months: persistent lymphocytic gastritis, but no metaplasia/dysplasia | - Inflammation occurred primarily in the gastric antrum - Some involvement of corpus, but predominantly antral gastritis | No tumor, dysplasia, or invasion observed in any strain up to 15 months | - Lymphocyte-dominant infiltration in lamina propria - Presence of macrophages and neutrophils, especially in C57BL/6 - Chronic active gastritis resembling human type B gastritis | - C57BL/6 or BALB/c + SS1: ideal for studying host immune responses, vaccine testing, antibiotic eradication, and bacterial colonization dynamics - Not suitable for modeling gastric cancer or metaplasia - Best for standardized, immunologically tractable chronic infection model |

**Table 2:** Summary of Chemical Carcinogen–, Diet–, and Composite-Induction Animal Models for Gastric Precancerous Lesions

| Studies | Animal Model | Modeling Method and Principle | Feeding Conditions | Model Evaluation Methods | Advantages and Disadvantages | Detailed Time Course of Pathological Stages | Anatomical Sites of Lesions | Tumor Location and Invasion/Metastasis | Inflammation and Immune Cell Infiltration | Application Scenarios for Models |
| --- | --- | --- | --- | --- | --- | --- | --- | --- | --- | --- |
| Ref. (12) | Male Wistar rats, 8 weeks old, ~200 g at start | - Oral administration of the chemical carcinogen N-methyl-N'-nitro-N-nitrosoguanidine (MNNG or NG) via drinking water:   - 167 μg/mL for 40 weeks  - 84 μg/mL thereafter until moribund or natural death   - NG is a direct-acting mutagen that causes epithelial DNA damage and induces multistep carcinogenesis in the glandular stomach. | - Standard CLEA-2 commercial diet - NG administered in deionized water, stored cold and protected from light, changed every 2 days - Free access to food and NG-containing water - Housed in pairs | - Histopathology: H&E, PAS, Alcian green, Masson's trichrome, Gomori silver - Serial sacrifices: Day 1, 3, 7, then weekly for 5 weeks, then every 5 weeks until 50 weeks; then moribund rats killed - Morphological staging of lesions - Defined lesion types: regenerative hyperplasia, adenomatous hyperplasia, adenocarcinoma | - Advantages:   - Clear chronological mapping of precancerous to cancerous changes  - Well-established chemical carcinogenesis model  - High incidence of invasive adenocarcinoma   - Disadvantages:   - No spontaneous or immunological component (e.g., no *H. pylori*)  - No metastasis observed  - Not transplantable without modificatio | - Week 1–3: Epithelial erosion begins - Week 3–5: Regenerative glandular hyperplasia appears (base/margin of erosions) - Week 5–20: Persistent regenerative hyperplasia - Week 20–30: Adenomatous hyperplasia (mild atypia, submucosal extension) - Week 30+: Adenocarcinoma appears (progresses from mucosa to submucosa, muscularis, then serosa) - Week 60+: Almost all rats have adenocarcinoma with serosal invasion | - Antrum (lesser curvature): Primary site for all stages—regenerative hyperplasia, adenomatous hyperplasia, adenocarcinoma - Corpus/body: Mild atrophy, focal atypical glands in late stages; very limited lesions | - Adenocarcinomas showed progressive invasion into submucosa, muscularis propria, and serosa - No distant metastasis observed, but perineural and vascular invasion noted in some rats | - Early lesions (Week 1–10): Edema and round cell (lymphocyte-like) infiltration in submucosa - Later stages: Inflammation becomes less pronounced - No specific immune cell subtyping (e.g., macrophages) was performed - - No inflammation-driven carcinogenesis—purely chemically induced | - Gold-standard model for chemical gastric carcinogenesis - Study of stepwise epithelial transformation (hyperplasia → adenoma → carcinoma) - Used to evaluate mutagens/carcinogens in GI tract - Ideal for testing chemical carcinogenesis inhibitors - Reference model for invasion dynamics and morphology-based cancer staging |
| Ref. (13) | White rats, both male and female, weighing 50–100 g; specific strain not indicated, but likely standard lab outbred stock | - Repeated intragastric administration (via stomach tube) of MNNG (also called MNG), a potent direct-acting nitrosoguanidine carcinogen. - It induces squamous cell carcinoma in the forestomach through DNA alkylation. - Administered as suspension due to low solubility. | - MRC Diet 41B provided ad libitum - Water available freely - MNNG suspended in 30% aqueous ethanol (due to poor water solubility) - Total dose per rat: 10–50 mg over 3–5 doses (each ~50–100 mg/kg), at intervals of several months | - Gross pathological inspection (tumor number, size, distribution) - Histopathology (H&E staining): squamous cell carcinoma diagnosis - Time to sacrifice: 12–24 months after first dose - Observation of tumor spread and metastatic nodules | - Advantages:   - Potent chemical induction of squamous carcinoma in fore-stomach  - Demonstrates multi-centric tumor origin and invasive potential   - Disadvantages:   - No lesions in glandular stomach  - Chemical exposure model (no infection/inflammation)  - Low solubility limits precision dosing  - Lack of immunological component | - 12–24 months post-first dose: Multiple squamous tumors of forestomach - Early lesions: small, multi-centric nodules or projections - Late-stage: confluent keratinizing masses invading muscularis; one case with peritoneal metastases - Hyperplasia seen in glandular stomach but no carcinoma there | - Forestomach (non-glandular): site of all squamous carcinomas - Glandular stomach: occasional hyperplasia/abnormality, but no tumors observed | - Tumors invaded stomach wall muscles and spread submucosally - Metastases: Observed in at least one case—numerous peritoneal nodules indicating metastatic dissemination | - Not addressed in this study - No mention of inflammation or immune response - Tumorigenesis attributed entirely to direct genotoxicity of MNNG (alkylating mutagen) | - Study of direct-acting genotoxic carcinogens - Model of squamous cell carcinoma in non-glandular stomach - Investigation of multicentric tumor origin and invasion - Toxicology screening of nitrosated compounds - Historical benchmark for alkylating agents in carcinogenesis research |
| Ref. (14) | Male Mongolian gerbils (MGS/Sea), 6–7 weeks old, specific pathogen-free | - Chemical carcinogenesis model using MNU (30, 10, or 3 ppm) or MNNG (400 or 200 ppm) in drinking water. - *H. pylori* (ATCC43504) infection used to induce gastritis and simulate human disease. - Investigates interaction of chemical carcinogens with bacterial infection. | - Food: Oriental NMF diet ad libitum - Water: Freshly prepared carcinogen solutions (light-protected), ad libitum - Housed in air-conditioned biohazard room (12h light/dark cycle) | - Histopathology: H&E, Alcian blue-PAS, immunohistochemistry (anti-*H. pylori*), Con A mucin staining - Tumor classification: adenoma, well/poorly differentiated adenocarcinoma, signet ring carcinoma - Cultures for *H. pylori* detection - Survival and body weight monitoring | - Advantages:   - Accurately mimics human gastric carcinogenesis  - Allows study of inflammation–carcinogen synergy  - Supports long-term H. pylori colonization   - Disadvantages:   - MNU at 30 ppm causes early mortality  - No intestinal metaplasia seen  - No metastatic tumors reported | - Week 20:   - 30 ppm MNU group: adenocarcinoma, ulcers, epithelial dysplasia  - 400 ppm MNNG: epithelial atypia   - Week 50:   - MNU 10 ppm: 2/21 adenocarcinoma (9.5%)  - MNU 3 ppm: 1/23 adenocarcinoma (4.3%)  - MNNG 400 ppm: 7/11 adenocarcinoma (63.6%)  - MNNG 200 ppm: 1/10 adenocarcinoma (10.0%)   - *H. pylori* infection (week 20): erosion, severe gastritis, no tumors | - Glandular stomach (mostly pyloric region, some fundic): adenomas, adenocarcinomas - Forestomach: squamous cell carcinomas - Duodenum/small intestine: adenocarcinomas from Brunner's glands in MNU/MNNG groups | - Local invasion observed (submucosal and muscular penetration) - No distant metastasis reported | - *H. pylori*-infected animals:   - Heavy infiltration of lymphocytes, macrophages, neutrophils in lamina propria and submucosa  - Lymphoid follicles frequently formed   - Carcinogen-treated animals: inflammation not prominent unless combined with *H. pylori* | - Dual-factor gastric carcinogenesis model (bacteria + carcinogen) - Study of *H. pylori*-related inflammation and cancer progression - Evaluation of gastric tumor histotypes: tubular, signet ring, poorly differentiated - Chemoprevention or microbiome intervention studies - Pathophysiology of gastric epithelial transformation under chronic insult |
| Ref. (15) | - Strain: BALB/c mice - Age and Gender: 6-week-old male mice | - Model: Induction of adenocarcinomas in the glandular stomach using N-methyl-N-nitrosourea (MNU) - Principle: MNU is a potent carcinogen that induces DNA damage, leading to mutations and subsequent neoplastic transformation in the glandular stomach epithelium. | - Diet: Oriental MF (Oriental Yeast Co., Tokyo) - Water: Ad libitum - Housing: Housed in plastic cages on hardwood chip bedding in an air-conditioned room with a 12 h light-12 h dark cycle | - Groups:   - Group 1: 30 mice treated with MNU and underwent forestomach resection after the last MNU administration.  - Group 2: 30 mice treated with MNU without forestomach resection.  - Group 3: 10 mice underwent forestomach resection without MNU treatment.  - Group 4: 10 non-treated control mice.   - Sacrifice and Examination: Animals were sacrificed at weeks 20, 30, and 40. Stomachs were fixed in formaldehyde or acetone, embedded in paraffin, and stained with hematoxylin and eosin (H.E.) and the paradoxical concanavalin A (Con A) method. - Histopathological Analysis: Examination of adenomatous hyperplasia, intramucosal and invasive adenocarcinomas, poorly differentiated adenocarcinomas, and signet ring cell carcinomas. | - Advantages:   - High incidence of well-differentiated adenocarcinomas and signet ring cell carcinomas.  - Demonstrates local invasion and lymph node metastasis, mimicking human gastric cancer.  - Simple administration of MNU without the need for additional surfactants or hormones.  - Suitable for analysis of early gastric carcinogenesis.   - Disadvantages:   - High mortality rate in Group 2 due to squamous cell carcinoma development in the forestomach.  - No intestinal metaplasia observed, which may limit the model's relevance to certain human gastric cancer subtypes. | - Adenomatous Hyperplasia: Found in groups 1 and 2 from week 20 at high incidence (over 75%), increasing with time. By week 40, all MNU-treated mice exhibited adenomatous hyperplasia - Intramucosal Adenocarcinomas: Found in groups 1 and 2 at week 20 (11.1% in Group 1, 11.1% in Group 2), increasing to 100% by week 40. - Invasive Adenocarcinomas: Found in groups 1 and 2 at week 30 (22.2% in Group 1, 22.2% in Group 2), increasing to 100% by week 40. - Poorly Differentiated Adenocarcinomas: Found in 4 out of 46 effective animals at week 40. - Signet Ring Cell Carcinomas: Found in 5 out of 46 effective animals at week 20 in Group 2, with additional cases developing by week 40. | - .Primary Locations: Predominantly in the pyloric region of the glandular stomach, often presenting as ulcerative nodules with elevated borders. - Other Locations: No mention of lesions in the gastric body or cardia. | - Invasiveness: Well-differentiated adenocarcinomas showed local invasion into the stomach wall. - Metastasis: Two out of 27 adenocarcinomas metastasized to regional lymph nodes. One poorly differentiated adenocarcinoma invaded the pancreas. | - Inflammation: No specific mention of inflammatory cells or inflammation in the study. - Immune Cells: No detailed analysis of immune cell infiltration, such as macrophages, was conducted. | - Early Gastric Carcinogenesis: Suitable for studying the early stages of gastric cancer development. - Drug Screening: Can be used to evaluate the efficacy of anti-cancer drugs targeting gastric adenocarcinomas. - Genetic Studies: Applicable for investigating genetic alterations and molecular mechanisms in gastric cancer. - Metastasis Research: Useful for studying the metastatic potential of gastric cancer cells to lymph nodes and other organs. |
| Ref. (16) | - Strain: BALB/c mice - Age and Gender: 6-week-old male mice | - Model: Induction of adenocarcinomas in the glandular stomach using N-methyl-N-nitrosourea (MNU) in drinking water. - Principle: The study examines the influence of carcinogen concentration on the histopathological types and cellular differentiation of induced tumors. The induction of tumors is found to be concentration-dependent rather than total intake-dependent. | - Diet: Oriental NMF (Oriental Yeast Co., Tokyo) - Water: MNU dissolved in distilled water, provided ad libitum from light-shielded bottles. - Housing: Housed in plastic cages on hardwood chip bedding in an air-conditioned room with a 12 h light-12 h dark cycle. | - Groups: - - Group 1: MNU at 240 ppm on alternate weeks for a total exposure of five weeks. - - Group 2: MNU at 120 ppm on alternate weeks for a total exposure of ten weeks. - - Group 3: MNU at 60 ppm for 20 weeks continuously. - - Group 4: MNU at 30 ppm for 40 weeks continuously. - Sacrifice and Examination: All survivors were sacrificed at 50 weeks. Stomachs were fixed in buffered formalin, processed for paraffin embedding, and stained with hematoxylin and eosin (H.E.) for histopathological assessment. - Histopathological Analysis: Neoplastic lesions were classified as adenocarcinomas, adenomas, and sarcomas. Adenocarcinomas were further classified into well-differentiated, poorly differentiated, and signet ring cell types. - Mucin Histochemistry and Immunohistochemistry: Techniques including paradoxical concanavalin A (Con A) staining, galactose oxidase Schiff (GOS), and sialidase GOS (S-GOS) were used. Immunohistochemistry was performed using anti-pepsinogen isozyme 1 (Pg 1) antibody. | - Advantages:   - Demonstrates that tumor induction is concentration-dependent rather than total intake-dependent.  - Provides a model for studying both differentiated and undifferentiated types of gastric cancer.  - Suitable for investigating the phenotypic expression of tumor cells.   - Disadvantages:   - High mortality rate in Group 3 due to prolonged exposure to lower concentrations of MNU.  - No intestinal metaplasia observed, limiting the model's relevance to certain human gastric cancer subtypes. | - Adenomas: Found in all groups, with the highest incidence in Group 1 (63.6%). - Adenocarcinomas: Found in all groups, with the highest incidence in Group 1 (77.3%). - Well-differentiated Adenocarcinomas: Predominantly polypoid lesions in the pyloric gland region. - Poorly Differentiated Adenocarcinomas: Flat or polypoid lesions with severe cellular atypia. - Signet Ring Cell Carcinomas: Isolated tumor cells containing abundant mucin. - Sarcomas: Found in all groups, with the highest incidence in Group 4 (35.3%). - Advanced Carcinomas: Higher incidence in Group 1 (36%). - Metastasis: One well-differentiated adenocarcinoma in Group 1 metastasized to regional lymph nodes. | Predominantly in the pyloric gland region and fundic gland region of the glandular stomach. | - Invasiveness: Advanced lesions exhibited a polypoid appearance with ulceration and marked thickening of the stomach wall due to diffuse infiltration of carcinoma cells. - Metastasis: One well-differentiated adenocarcinoma in Group 1 metastasized to regional lymph nodes. | - Inflammation: No specific mention of inflammatory cells or inflammation in the study. - Immune Cells: No detailed analysis of immune cell infiltration, such as macrophages, was conducted. | - Histopathological Studies: Suitable for studying the histopathological features of gastric cancer. - Drug Screening: Can be used to evaluate the efficacy of anti-cancer drugs targeting gastric adenocarcinomas. - Genetic Studies: Applicable for investigating genetic alterations and molecular mechanisms in gastric cancer. - Concentration-Dependence Research: Useful for studying the effects of carcinogen concentration on tumor induction and differentiation. |
| Ref. (17) | - Strains Used: BALB/cA (BALB), C57BL/6N (C57BL6), CBA/JN (CBA), C3H/HeN (C3H), DBA/2N (DBA/2), and CD-1 (ICR)   Age and Gender: 7-week-old male mice | - Model: Induction of gastric tumors using N-methyl-N-nitrosourea (MNU) in drinking water. - Principle: The study examines strain differences in susceptibility to MNU-induced gastric carcinogenesis, focusing on the frequency of pepsinogen-altered pyloric glands (PAPGs) and the incidence of adenocarcinomas. It also investigates the presence of intestinal metaplasia and intestinal-type cells in gastric tumors. | - Diet: Oriental NMF (Oriental Yeast Co., Tokyo) - Water: MNU dissolved in distilled water (120 ppm), provided ad libitum from light-shielded bottles. - Housing: Housed in plastic cages with hardwood chips in an air-conditioned room with a 12 h light/12 h dark cycle. | - Experimental Design: Mice were given MNU in drinking water for 10 or 18 weeks and sacrificed at 11 or 52 weeks. Controls received tap water. - Histopathological Analysis: Tissue sections were stained with hematoxylin and eosin. Neoplastic lesions were classified into adenomas and adenocarcinomas, with further subclassification into well-differentiated, poorly differentiated, and signet-ring cell types. - Immunohistochemical and Enzyme Histochemical Analyses: Pepsinogen 1 (Pg 1) staining was used to identify PAPGs. Intestinal alkaline phosphatase (I-ALP) staining was used to detect intestinal-type cells. Mucin histochemistry was performed using alcian-blue (pH 2.5) periodic acid Schiff (AB-PAS) and paradoxical concanavalin A staining (PCS). | - Advantages:   - Demonstrates strain-specific differences in susceptibility to MNU-induced gastric carcinogenesis.  - Identifies PAPGs as a useful preneoplastic marker lesion across multiple mouse strains.  - Provides insights into the random occurrence of intestinal metaplasia in gastric tumors.   - Disadvantages:   - Limited observation period (up to 52 weeks) may not capture long-term phenotypic changes.  - Lack of detailed genetic analysis of tumor development. | - PAPGs: Highest frequency in BALB mice (19.6 ± 9.9%) and lowest in ICR mice (12.3 ± 5.7%) at 10 weeks. - Adenomas and Adenocarcinomas: Incidences at week 52 were highest in BALB mice (adenomas: 63.0%, adenocarcinomas: 59.3%) and lowest in ICR mice (adenomas: 48.1%, adenocarcinomas: 18.5%). - Histological Classification:   - BALB: Predominantly well-differentiated adenocarcinomas.  - C3H: Higher incidence of poorly differentiated adenocarcinomas.   - Depth of Invasion: BALB mice exhibited deeper invasion compared to other strains. | Primary Locations: Pyloric glands and glandular stomach. | - Invasiveness: Adenocarcinomas in BALB mice showed deeper invasion compared to other strains. - Metastasis: No specific mention of metastasis in this study. | - .Inflammation: No specific mention of inflammatory cells or inflammation. - Immune Cells: No detailed analysis of immune cell infiltration. | - Histopathological Studies: Suitable for studying strain-specific differences in gastric carcinogenesis. - Drug Screening: Can be used to evaluate the efficacy of anti-cancer drugs in different mouse strains. - Genetic Studies: Applicable for investigating genetic alterations in gastric cancer development. - Preneoplastic Marker Studies: Useful for studying the role of PAPGs as preneoplastic lesions. |
| Ref. (18) | The protocol applies to both wild-type and genetically engineered mice. Specifically mentioned strains include:   - Tsc1-deficient C57BL/6 mice (main model used) - Protocol also applicable to Mek1-deficient and Bmpr1a-deficient mouse lines. - Mice were 2–3 months old, 4 per cage. | - Gastric tumors were induced by oral administration of N-methyl-N-nitrosourea (MNU) at 240 ppm in drinking water. - Exposure was given in five 1-week cycles of MNU, alternating with 1-week distilled water, for a total of 9 weeks. - After the 5 cycles, mice received normal distilled water until week 52. - The carcinogen induces gastric tumors through DNA alkylation and mutation mechanisms. | - Mice were kept in specific pathogen-free (SPF) conditions. - Standard food and water were provided ad libitum. - 4 mice per cage, started at 2–3 months of age. | - Mice were sacrificed at 52 weeks. - Stomachs were harvested and examined macroscopically. - Histopathological evaluation was performed using H&E staining. - Tumors were classified by differentiation degree (well to poorly differentiated adenocarcinoma). | - Advantages:   - Simple and highly reproducible protocol.  - Applicable across multiple genetically engineered strains.  - Tumors obtained include various degrees of differentiation.  Disadvantages:  - Long latency (tumors develop at 52 weeks).  - No intermediate lesion data (e.g., hyperplasia, metaplasia). | - Only endpoint data at 52 weeks is provided. - Histopathology showed:   – Papillary adenomas  – Well, moderately, and poorly differentiated adenocarcinomas   - No staging of intermediate lesions (e.g., atrophy, metaplasia, dysplasia) is included. | - Tumors were localized to the glandular stomach. - No further distinction between antrum vs. corpus is provided in the article. | No data on tumor invasion into adjacent tissues or distant metastasis is reported. | - The article does not discuss inflammatory responses. - No immune cell types (e.g., macrophages, lymphocytes) or inflammatory cytokines are analyzed or described. | - Suitable for long-term chemical carcinogenesis studies in both wild-type and genetically engineered mice. - Effective for investigating gastric tumor formation, histological differentiation, and tumor suppressor gene function (e.g., Tsc1, Mek1, Bmpr1a). - Applicable to drug screening and chemoprevention evaluation. - Not suitable for studies involving infection-driven inflammation (e.g., *Helicobacter pylori* models) or immune cell dynamics. |
| Ref. (19) | Male Mongolian gerbils (6–8 weeks old, 41–50g) | - Oral infection with wild-type cagA⁺ *H. pylori* strain 7.13 (or isogenic cagA mutant) followed by 16-week exposure to either regular diet (0.75% NaCl) or high-salt diet (8.75% NaCl). - The high salt condition upregulates cagA expression, exacerbates inflammation, hypochlorhydria, and adenocarcinoma formation. | - Regular diet: Purina 5001 (0.75% NaCl) - High-salt diet: Purina 5001 + 8% NaCl (final 8.75%) - Fed 1 week before and throughout infection period. | - CFU counts for bacterial burden - Histopathology (H&E staining) for inflammation, dysplasia, carcinoma - Immunohistochemistry for H⁺/K⁺-ATPase (parietal cells) - - Gastric pH measurement - qRT-PCR for host cytokines and cagA transcription | - Advantages:   - Mimics human pathology  - High reproducibility  - Histologically defined stages   - Disadvantages:   - Species differences from humans  - Limited immunological tools for gerbils  - No metastasis evaluation beyond gastric wall. | - Week 0: Infection begins - Week 16: Sacrifice & analysis - At 16 weeks: 100% carcinoma in WT + high-salt; 58% in WT + regular diet; 0% in cagA mutant groups. Dysplasia and inflammation also appear at this point. | - Predominantly in antrum and corpus (body). - Inflammation and carcinogenic lesions occurred in both regions; more severe in high-salt WT group. | - Tumors invaded submucosa and muscularis propria. - No evidence of metastasis was mentioned in the study. | - Prominent inflammation. - Increased polymorphonuclear (acute) and mononuclear (chronic) cells. - Cytokines: ↑ IL-1β, IL-6, IL-17, IFN-γ, iNOS - Increased macrophage-associated markers (IL-1β, iNOS). - T-helper responses: Th1 and Th17. | - Studying the role of dietary factors (e.g., salt) in gastric carcinogenesis - Evaluating *H. pylori* virulence (especially CagA) and host interactions - Investigating inflammation-driven gastric cancer - Testing preventive or therapeutic strategies targeting *H. pylori*, inflammation, or dietary modulation |
| Ref. (20) | Outbred male Mongolian gerbils [Crl:(MON)], 25 weeks old at the start | - Two independent models were used: 1. Oral inoculation with *H. pylori* SS1 strain at 25 weeks of age (2.5×10⁸ CFU, three doses), sacrificed at 62 weeks (37 weeks post-infection); 2. Feeding uninfected gerbils a 2.5% salt diet for 56 weeks. - The goal was to study atrophic gastritis and intestinal metaplasia without confounding factors. | - Control & *H. pylori*-infected: 0.25% NaCl diet (standard Purina TestDiet) - High-salt group: 2.5% NaCl diet (Purina TestDiet). All diets fed ad libitum from age 5 to 62 weeks (56 weeks duration). | - Histopathology (H&E, Alcian blue/PAS, pH 2.5) - PCR to confirm *H. pylori* colonization (ureC gene) - Semi-quantitative scoring system (0–4) for: inflammation, atrophic gastritis, intestinal metaplasia | - Advantages:   - Demonstrates independent effects of diet and infection  - uses commercially available gerbils  - replicates key preneoplastic features (intestinal metaplasia, atrophy).   - Disadvantages:   - No adenocarcinoma at 37 weeks  - SS1 strain lacks complete cagPAI  - no time-point resolution for lesion progression. | - *H. pylori* infection (37 weeks post-infection): Chronic active gastritis, atrophic gastritis, intestinal metaplasia (all 5/5 animals) - High-salt (2.5%, 56 weeks): Atrophic gastritis, intestinal metaplasia (6/6 animals), but no inflammation. - Timepoints of lesion appearance within study: Only endpoint evaluated was 56 weeks; early lesion timing undetermined. | - Lesions found in both gastric body and antrum. - Most severe changes (atrophy, metaplasia) often at junction of corpus and antrum. - Intestinal metaplasia scoring was limited to the gastric body. | - No evidence of adenocarcinoma or metastasis at 37 weeks post-infection. - Some infected animals showed submucosal gland herniation but without invasion into muscle or vasculature. | - *H. pylori*-infected animals: Intense mucosal and submucosal lymphoid aggregates, neutrophils (microabscesses), chronic active gastritis. - High-salt group: No significant inflammation, suggesting non-inflammatory pathogenesis. | - Study of independent effects of diet and *H. pylori* on gastric precancerous lesions - Dissection of host vs. bacterial strain contributions (SS1, outbred vs. inbred) - Investigation of intestinal metaplasia mechanisms - Early-phase gastric carcinogenesis research without requiring tumor endpoint - Development of models for dietary intervention and chemoprevention studies |
| Ref. (21) | Female C57BL/6 mice, 8 weeks old | - Oral inoculation with *H. pylori* Sydney strain (10⁸ CFU per mouse) combined with dietary manipulation: either normal (0.25% NaCl) or high-salt (7.5% NaCl) diet. - Model evaluates epithelial hyperplasia and parietal cell loss as early preneoplastic events. | - Normal diet: 0.25% NaCl (Purina) - High-salt diet: 7.5% NaCl (isocaloric, otherwise identical to control diet) - Fed ad libitum starting 1 week before infection through the study (up to 16 weeks). | - *H. pylori* colonization: culture (CFU/g tissue), urease activity - Histopathology: H&E staining (inflammation, atrophy, pit length, parietal cell count) - Cell proliferation: BrdUrd labeling index - Serum gastrin: radioimmunoassay | - Advantages: Quantitative colonization data; detailed epithelial cell dynamics; replicates early host changes in gastric cancer; established immunological tools for C57BL/6 mice. - Disadvantages: No adenocarcinoma or intestinal metaplasia; short timeline (max 16 weeks); female mice only. | - Week 4: Increased urease activity; pit elongation begins. - Week 8: Elevated H. pylori CFU; pit elongation and parietal cell loss visible. - Week 16: Maximal effects—marked epithelial hyperplasia, parietal cell depletion, moderate to severe corpus gastritis in infected mice. - No carcinoma or dysplasia observed. | Lesions observed in both gastric corpus and antrum, but corpus more affected (inflammation, hyperplasia, parietal cell loss). | - No evidence of invasive or metastatic lesions during the 16-week study. - Only epithelial proliferation and glandular remodeling. | - Infection group: Chronic active gastritis with mononuclear cells, lymphocytes, and neutrophils. - High-salt group: No significant inflammation, despite marked epithelial changes. - Combined infection + salt: No synergistic increase in inflammation beyond *H. pylori* alone. | - Studying early gastric changes in *H. pylori* infection - Investigating dietary salt’s role in gastric pathology and bacterial colonization - Analyzing parietal cell loss and epithelial proliferation mechanisms - Precursor modeling for gastric carcinogenesis - Evaluating potential diet-based interventions in *H. pylori* infection |
| Ref. (22) | Swiss/ICR mice (both sexes), 4 weeks old at start | - Long-term feeding (up to 12 months) with rice-based diets: - Rice diet: 90% rice flour + 10% standard pellet - Salty rice diet: 80% rice flour + 10% standard pellet + 10% NaCl - Goal: to model human dietary risk factors for gastric atrophy by manipulating macronutrient and salt content. | - Standard pellet, rice, or salty rice diet fed ad libitum for 3, 10, or 12 months. - Room temperature: 23°C, 12-hour light/dark cycle - Water provided ad libitum - Some mice were given hydrocortisone (1 mg/day s.c. for 11 days prior to sacrifice in certain groups). | - Morphometry: stomach area measured from plastic tracings - Wet weight measurements of forestomach and glandular stomach - Histopathology (H&E): evaluated hyperplasia, keratosis, and parietal cell loss - Statistical analysis: Student’s t-test | - Advantages: Simple, non-infectious model of gastric atrophy; long-term dietary control; highlights steroid-mediated changes; potential insights into human dietary risks. - Disadvantages: No neoplastic or dysplastic lesions induced; no *H. pylori* infection; lesions confined to non-glandular stomach hyperplasia and glandular atrophy; no inflammation or immune data. | - 3 months: mild changes in forestomach and glandular regions - 10 months: noticeable parietal cell loss, hypertrophy in forestomach (enhanced by hydrocortisone) - 12 months: 55–75% of rice/salty rice-fed mice had macroscopic papillary growths in forestomach and marked parietal cell loss in glandular stomach - No dysplasia or carcinoma seen even after 12 months | - Forestomach (non-glandular): hyperplasia and hyperkeratosis with papillary projections - Glandular stomach (fundic and pyloric): atrophy with significant parietal cell reduction - No involvement of gastric antrum specifically mentioned | - No evidence of invasion or metastasis. - Lesions were limited to hyperplasia and atrophy without neoplasia. | - No inflammation observed. Histological changes were not associated with infiltration of immune or inflammatory cells. - Mechanism proposed involved hormonal dysregulation (especially glucocorticoids), not immune response. | - Modeling diet-induced gastric epithelial changes (e.g., atrophy, hyperplasia) - Investigating glucocorticoid-mediated gastric remodeling - Exploring early-stage morphological changes preceding carcinogenesis - Studying the impact of high carbohydrate and NaCl intake on gastric structure - Relevant to nutrition-related cancer epidemiology (e.g., salt and cancer risk in Japan) |
| Ref. (23) | - Strain: Wistar rats - Age and Gender: Male rats, initially weighing 200 g | - Model: Induction of gastric carcinomas using N-methyl-N'-nitro-N-nitrosoguanidine (MNNG) and 4-nitroquinoline-1-oxide (NQO) in the presence or absence of sodium chloride (NaCl). - Principle: The study investigates whether NaCl enhances the carcinogenic effects of MNNG and NQO in the rat stomach. The mechanism is hypothesized to involve NaCl reducing the protective mucosal barrier, allowing carcinogens to directly contact the gastric mucosa. | - Diet: Standard diet (Oriental MF, Oriental Yeast Co., Ltd., Tokyo, Japan) supplemented with 10% NaCl in some groups. - Water: MNNG or NQO solutions in drinking water, with additional NaCl in some groups. - Housing: Rats were housed 5 to a screen-bottomed cage under natural light in an air-conditioned room at 24°C. | - Experimental Design: Nine groups of rats were treated with different combinations of MNNG, NQO, and NaCl.   - Group 1: 50 mg MNNG/liter + 6 g NaCl solution/liter in drinking water + 10% NaCl in diet.  - Group 2: 50 mg MNNG/liter in drinking water + weekly 1 ml saturated NaCl solution + standard diet.  - Group 3: 50 mg MNNG/liter in drinking water + standard diet.  - Group 4: Weekly 1 mg NQO in 20% ethanol + 10% NaCl in diet.  - Group 5: Weekly 1 mg NQO in 20% ethanol saturated with NaCl + standard diet.  - Group 6: Weekly 1 mg NQO in 20% ethanol + standard diet.  - Groups 7 and 8: NaCl alone in drinking water or diet.  - Group 9: Untreated controls.   - Observation: Rats were observed for up to 85 weeks. Tissues were examined histologically after fixation in 10% formaldehyde solution and staining with hematoxylin and eosin. | - Advantages:   - Demonstrates the enhancing effect of NaCl on carcinogenesis.  - Provides insights into the role of dietary salt in gastric cancer development.  - Uses a well-established carcinogen (MNNG) and a relevant animal model (Wistar rats).   - Disadvantages:   - Limited to short-term observations (up to 85 weeks).  - Does not explore the molecular mechanisms of NaCl's enhancing effect.  - No detailed analysis of immune cell infiltration or inflammatory responses. | - Glandular Stomach: Adenocarcinomas: Developed in groups 1-3 treated with MNNG.   - Group 1: 11 of 18 rats developed adenocarcinomas (61.1%).  - Group 2: 12 of 15 rats developed adenocarcinomas (80.0%).  - Group 3: 12 of 27 rats developed adenocarcinomas (44.4%).  Time Points: - Tumors were observed after 40 weeks of treatment.   - Forestomach: Squamous Cell Carcinomas: Developed in groups 4-6 treated with NQO.   - Group 4: 7 of 18 rats developed squamous cell carcinomas (38.9%).  - Group 5: 9 of 17 rats developed squamous cell carcinomas (52.9%).  - Group 6: No malignant tumors observed.  - Time Points: Tumors were observed after 40 weeks of treatment. | - Glandular Stomach: Predominantly in the pyloric region, presenting as polypoid or ulcerative nodules. - Forestomach: Large papillary masses with hyperkeratosis. | - Glandular Stomach:   - Invasion: Some tumors adhered to the omentum and liver.  - Metastasis: One poorly differentiated adenocarcinoma metastasized to the lymph nodes in Group 1.   - Forestomach:   - Invasion: Squamous cell carcinoma invaded the muscle layer of the glandular stomach in Group 4.  - Metastasis: One squamous cell carcinoma metastasized to the intestinal lymph nodes and liver in Group 5. | - Inflammation: No significant histopathologic changes were observed in non-tumor areas of the stomach. - Immune Cells: No detailed analysis of immune cell infiltration or inflammatory factors was conducted. | - Carcinogenesis Studies: Suitable for studying the effects of dietary factors on gastric cancer development. - Drug Screening: Can be used to evaluate the efficacy of anti-cancer drugs in a relevant animal model. - Mechanistic Studies: Provides a basis for investigating the molecular mechanisms of NaCl-enhanced carcinogenesis. |
| Ref. (24) | - Strain: Male outbred Wistar rats - Age: 7-week-old | - Model: Induction of gastric carcinogenesis using N-methyl-N'-nitro-N-nitrosoguanidine (MNNG) in drinking water. - Principle: The study investigates whether sodium chloride (NaCl) enhances the carcinogenic effects of MNNG during the initiation or promotion stage of gastric carcinogenesis. | - Diet:   - Control Diet: Standard diet (Oriental MF, Oriental Yeast Co., Ltd., Tokyo)  - High Salt Diet: Standard diet supplemented with 10% NaCl (Wako Pure Chemical Ind. Ltd.)   - Water:   - MNNG Solution: 100 mg/liter MNNG in drinking water for 20 weeks  - Control Water: Tap water   - Housing: Rats were housed in plastic cages in an animal room with a 12-hour light cycle at 22±2°C and 60% relative humidity. | - Experimental Design:   - Group 1: MNNG for 20 weeks, then high salt diet for the next 20 weeks.  - Group 2: High salt diet for the first 20 weeks, then MNNG for the next 20 weeks.  - Group 3: High salt diet throughout the 40 weeks, with MNNG for the first 20 weeks.  - Group 4: High salt diet throughout the 40 weeks, no MNNG.  - Group 5: Control diet throughout the 40 weeks, with MNNG for the first 20 weeks.  - Group 6: Control diet throughout the 40 weeks, with 150 mg/liter MNNG for the first 20 weeks (positive control).   - Observation: Rats were weighed weekly and water consumption was measured daily for the first 20 weeks. Surviving rats were sacrificed at 40 weeks, and tissues were examined histologically. | - Advantages:   - Demonstrates the enhancing effect of NaCl on MNNG-induced gastric carcinogenesis.  - Provides insights into the timing of NaCl's effect (initiation vs. promotion stage).  - Uses a well-established carcinogen (MNNG) and a relevant animal model (Wistar rats).   - Disadvantages:   - Limited to short-term observations (40 weeks).  - Does not explore the molecular mechanisms of NaCl's enhancing effect.  - No detailed analysis of immune cell infiltration or inflammatory responses. | - Adenomatous Hyperplasia: Early proliferative, non-invasive mucosal lesions, predominantly in the pyloric region. - Adenocarcinoma: Well-differentiated tumors with typical glandular structures, observed at 40 weeks.   - Group 2: 6 rats with tumors >1 cm in diameter.  - Group 3: 5 rats with tumors >1 cm in diameter.   - Sarcoma: Observed in the fundic region in Group 2 (1 sarcoma) and Group 3 (2 sarcomas). | - Glandular Stomach: Predominantly in the pyloric region, presenting as plaque-like or nodular lesions with ulceration. - Small Intestine: Adenocarcinomas found mostly within 10 cm of the pyloroduodenal junction. | - Invasion: Adenocarcinomas showed varying degrees of invasiveness, from submucosa to deeper layers of the stomach wall. - Metastasis: No metastasis was found in any of the groups. | - Inflammation: No significant histopathologic changes were observed in non-tumor areas of the stomach. - Immune Cells: No detailed analysis of immune cell infiltration or inflammatory factors was conducted. | - Carcinogenesis Studies: Suitable for studying the effects of dietary factors on gastric cancer development. - Drug Screening: Can be used to evaluate the efficacy of anti-cancer drugs in a relevant animal model. - Mechanistic Studies: Provides a basis for investigating the molecular mechanisms of NaCl-enhanced carcinogenesis. |
| Ref. (25) | - Strain: Male outbred Wistar rats - Age: 6 weeks old | - Model: Induction of gastric carcinogenesis using N-methyl-N'-nitro-N-nitrosoguanidine (MNNG) in drinking water. - Principle: The study investigates the dose-dependent promoting effects of sodium chloride (NaCl) on gastric carcinogenesis initiated by MNNG. The hypothesis is that NaCl enhances tumor development in a dose-dependent manner during the promotion stage. | - Diet:   - MNNG Treatment: 100 ppm MNNG in drinking water for 8 weeks.  - NaCl Supplementation: After the initial MNNG treatment, rats were fed diets supplemented with 10%, 5%, 2.5%, or 0% NaCl for 82 weeks.   - Water: Tap water after the initial MNNG treatment. - Housing: Rats were housed in wire cages in an animal room with a 12-hour light cycle at 22±2°C and 55±5% relative humidity. | - Experimental Design:   - Groups 1-4: MNNG-treated rats fed diets with 10%, 5%, 2.5%, or 0% NaCl.  - Groups 5-8: Control rats fed diets with 10%, 5%, 2.5%, or 0% NaCl without MNNG.   - Observation: Rats were weighed every 4 weeks. Surviving rats were sacrificed at 90 weeks, and tissues were examined histologically. - Histopathological Analysis: Tumors were classified into adenomas, adenocarcinomas, and mesenchymal tumors. Urinary lipid peroxidation (LP) levels were measured using the thiobarbituric acid (TBA) reaction method. | - Advantages:   - Demonstrates a clear dose-dependent promoting effect of NaCl on MNNG-induced gastric carcinogenesis.  - Provides insights into the potential role of lipid peroxidation in NaCl-mediated tumor promotion.  - Uses a well-established carcinogen (MNNG) and a relevant animal model (Wistar rats).   - Disadvantages:   - Limited to short-term observations (90 weeks).  - Does not explore the molecular mechanisms of NaCl's promoting effect.  - No detailed analysis of immune cell infiltration or inflammatory responses. | - Adenomas: Proliferative but non-invasive lesions in the mucosal or submucosal layer. - Adenocarcinomas: Well-differentiated tumors with irregular glandular structures, observed at 90 weeks.   - 10% NaCl: 80.0% incidence of adenomas/adenocarcinomas.  - 5% NaCl: 55.6% incidence of adenomas/adenocarcinomas.  - 2.5% NaCl: 50.0% incidence of adenomas/adenocarcinomas.  - 0% NaCl: 30.0% incidence of adenomas/adenocarcinomas.   - Mesenchymal Tumors: Observed in all MNNG-treated groups, but incidence was not statistically different between groups. | - Glandular Stomach: Tumors were evenly distributed throughout both the pyloric and fundic regions. - Small Intestine: No adenomas or adenocarcinomas observed in any group. | - Invasion: Adenocarcinomas showed varying degrees of invasiveness, from submucosa to deeper layers of the stomach wall. - Metastasis: No metastasis was found in any of the groups. | - Inflammation: No significant histopathologic changes were observed in non-tumor areas of the stomach. - Immune Cells: No detailed analysis of immune cell infiltration or inflammatory factors was conducted. | - Carcinogenesis Studies: Suitable for studying the effects of dietary factors on gastric cancer development. - Drug Screening: Can be used to evaluate the efficacy of anti-cancer drugs in a relevant animal model. - Mechanistic Studies: Provides a basis for investigating the molecular mechanisms of NaCl-enhanced carcinogenesis. |
| Ref. (26) | - Strain: C57BL/6 × 129S6/SvEv (B6129) mice - Age: 8 weeks old at the start of the experiment | - Model: Induction of gastric intraepithelial neoplasia (GIN) using *Helicobacter pylori* (*H. pylori*) infection. - Principle: The study investigates whether *H. pylori* infection induces gastric neoplasia in B6129 mice and whether a high-salt diet promotes tumorigenesis. The hypothesis is that *H. pylori* infection, rather than high salt intake, is the primary driver of gastric carcinogenesis. | - Diet:   - Basal Diet: 0.25% salt  - High-Salt Diet: 7.5% salt   - Infection: Mice were gavaged with 10^8^ colony-forming units (CFU) of *H. pylori* Sydney strain-1 (SS1) or vehicle only every other day for three doses. - Housing: Mice were housed in microisolator cages on hardwood shavings with a 12-hour light/dark cycle, constant humidity, and temperature control. | - Experimental Design:   - Group 1: Sham-inoculated mice on basal diet  - Group 2: Sham-inoculated mice on high-salt diet  - Group 3: *H. pylori*-infected mice on basal diet  - Group 4: *H. pylori*-infected mice on high-salt diet   - Observation: Mice were euthanized at 6, 12, and 15 months of age. Stomachs were examined histologically, and tissues were stained with H&E, Alcian blue, and periodic acid-Schiff (PAS). - Quantitative Culture: H. pylori colonization levels were determined by quantitative culture. - Serology: Serum antibodies specific to *H. pylori* were measured by ELISA. - Histopathology: Gastric lesions were scored using a standardized scale. - Proliferation and Apoptosis: Ki-67 labeling indices (LI) and caspase-3 immunohistochemistry were used to assess cell proliferation and apoptosis. | - Advantages:   - Demonstrates *H. pylori*-induced gastric intraepithelial neoplasia in a wild-type mouse model.  - Shows no additive effect of high-salt diet on *H. pylori*-induced tumorigenesis.  - Provides insights into the role of *H. pylori* infection in gastric carcinogenesis.  Disadvantages:  - Limited to a specific mouse strain (B6129).  - No evaluation of long-term dietary effects beyond 15 months.  - No detailed analysis of immune cell infiltration or inflammatory responses. | - Inflammation: Significant increases in inflammation scores by 6 months in *H. pylori*-infected mice. - Oxyntic Atrophy: Significant increases in oxyntic atrophy scores by 6 months. - Hyperplasia: Significant increases in hyperplasia scores by 12 months. - Intestinal Metaplasia: Significant increases in intestinal metaplasia scores by 12 months. - Dysplasia: High-grade dysplasia consistent with GIN by 15 months. - Proliferation: High-salt diet increased proliferation in uninfected mice (*P* = 0.003). - Apoptosis: High rates of apoptosis and proliferation in dysplastic regions. | - Gastric Corpus and Antrum: Lesions were observed in both the corpus and antrum of the stomach. - Intestinal Metaplasia: Characterized by columnar elongation of foveolar epithelium with rare goblet cells. | - Invasion: No evidence of invasive carcinoma or lymphatic/vascular invasion. - Metastasis: No metastasis observed in any of the groups. | - Inflammation: Chronic active gastritis observed in *H. pylori*-infected mice. - Immune Cells: Increased infiltration of eosinophils in mice on the high-salt diet. - Humoral Immunity: Shift from Th1 to Th2 pattern in *H. pylori*-infected mice on the high-salt diet. | - Carcinogenesis Studies: Suitable for studying the effects of *H. pylori* infection on gastric carcinogenesis. - Drug Screening: Can be used to evaluate the efficacy of anti-cancer drugs in a relevant animal model. - Mechanistic Studies: Provides a basis for investigating the molecular mechanisms of *H. pylori*-induced carcinogenesis. |
| Ref. (27) | - Male Wistar rats, 5 weeks old, 160–180 g | - Intragastric administration of 200 mg/kg MNNG on days 0 and 14, followed by alternating administration of MNNG (600 µg/kg) and saturated NaCl (1 ml/rat) starting from week 4. - The principle is to simulate high salt intake and carcinogen exposure, inducing microcirculatory disturbances, hypoxia, and activation of the COX‑2/HIF‑1α/VEGF pathway leading to chronic atrophic gastritis and intestinal metaplasia. | Housed at 25±2 °C, 60±2% humidity, 12-hour light/dark cycle, with ad libitum access to food and water. | - Histopathology (H&E, AB‑PAS staining) - Gastric mucosal blood flow (GMBF) via laser Doppler - Western blot and qPCR for COX‑2, HIF‑1α, VEGFR1/2 | - Advantages:   - High success rate (100% at 35 weeks)  - No mortality  - Simple and reproducible   - Disadvantages:   - Requires long-term induction (≥25 weeks)  - Molecular variability between replicates. | - Week 5: Normal histology. - Week 15: Onset of GMBF reduction. - Week 25: Mild chronic atrophic gastritis, no intestinal metaplasia. - Week 35: Moderate/severe chronic atrophic gastritis + intestinal metaplasia clearly present. | - Fundus, gastric body, and antrum. - Intestinal metaplasia especially confirmed in the antrum. | No evidence of invasion or metastasis was reported; model simulates precancerous stages only. | - Inflammatory marker COX‑2 was upregulated. - The paper does not specify immune cell types such as macrophages or lymphocytes. | - Study of gastric precancerous lesions (chronic atrophic gastritis and intestinal metaplasia). - Mechanistic studies on microcirculation, hypoxia, COX‑2/HIF‑1α/VEGF signaling. - Drug screening and development for chronic atrophic gastritis/intestinal metaplasia.. |
| Ref. (28) | Specific Pathogen-Free (SPF) C57BL/6 male mice, 6 weeks old. | - Oral administration of N-methyl-N-nitrosourea (MNU) at 200 ppm in drinking water (alternate weeks for 10 weeks = 5 weeks total exposure), followed by inoculation with *H. pylori* SS1 (strain: cagA⁺, vacA s2/m2). - Purpose: evaluate if *H. pylori* promotes chemically induced gastric cancer. | - Diet: Sterilized CE-2 commercial pellets - Water: Autoclaved distilled water - Housing: Plastic cages, 12 h light/dark cycle, air-conditioned biohazard room. | - Macroscopic observation of polypoid tumors (>2 mm) - Histopathology: H&E and PAS staining - Classification: Adenocarcinoma, adenomatous hyperplasia, gastritis - *H. pylori* detection: culture & ELISA for serum IgG. | - Advantages:   Clean SPF model, well-controlled infection   - Disadvantages:   - No enhancement of tumorigenesis by *H. pylori* SS1  - Only mild inflammatory response observed; not ideal to mimic severe pathology. | - Week 54: Final assessment of gastric lesions.  1. MNU-alone Group:   - Polypoid lesions: 67% (10/15)  - Adenocarcinomas: 47% (7/15)  - Adenomatous hyperplasia: 80% (12/15)   1. MNU+*H. pylori* Group:   - Polypoid lesions: 31% (8/26)  - Adenocarcinomas: 23% (6/26)  - Adenomatous hyperplasia: 35% (9/26) | Lesions were observed in the antrum and/or the border of the antrum and corpus. | No invasion beyond muscular layer; no metastasis observed. | - Severe gastritis only in *H. pylori*-infected group (19%) - Infiltrates: neutrophils and mononuclear cells - MNU-alone group: no severe gastritis. | - Study of strain-specific bacterial effects (cagA⁺/vacA s2/m2) - Testing host vs. bacterial factors in gastric carcinogenesis - Model for moderate gastritis, not invasive cancer. |
| Ref. (29) | C57BL/6J male mice, 5–6 weeks old. | - Mice were administered N-methyl-N-nitrosourea (MNU) in drinking water (120 ppm, alternate weeks for 5 weeks) to induce gastric tumors. - Groups B and D were infected with *H. pylori* (Sydney strain 1), while Groups C and D received a high-salt diet (10% NaCl in basal CE-2 diet). - This combination (Group D) was designed to mimic human gastric carcinogenesis involving carcinogen exposure, infection, and high-salt intake. | - All mice were housed under SPF conditions with free access to food and water. - Groups C and D received a 10% NaCl-supplemented CE-2 diet. - Food and water were provided ad libitum. | Evaluation included histopathological examination (H&E staining) for adenoma and adenocarcinoma classification, incidence and multiplicity analysis, oligonucleotide microarray for gene expression, and quantitative RT-PCR for validating key gene expressions (Cd177, Reg3g, Muc13). | - Advantages:   Compatible with gene expression profiling; mimics multifactorial human gastric cancer; allows evaluation of tumor multiplicity.   - Disadvantages:   Instability of *H. pylori* virulence factors in mice (e.g., cag PAI); limited invasion compared to gerbil models. | - Stomach tissues were harvested at 40 weeks. Pathological stages included: (1) adenoma, (2) adenocarcinoma with cellular atypia and submucosal invasion. - The article does not specify intermediate timepoints or stage evolution chronology. | - Tumors developed in the glandular stomach, particularly in the pyloric region (antrum). - Images and descriptions confirm localization to the pyloric region. | - Tumors classified as adenocarcinoma demonstrated invasion into the submucosa. - There is no mention of metastasis in mice. | - Inflammatory infiltration, especially neutrophils, was noted in gastric cancer tissues. - CD177 expression was observed in neutrophils and also in tumor cells, suggesting immune involvement. - Expression of Ela2 (neutrophil-specific gene) was unchanged. - The study did not quantify macrophages or other immune cells explicitly. | - This mouse model is suitable for studying gene expression changes, identifying prognostic biomarkers (e.g., CD177), and evaluating interactions between infection, diet, and carcinogen exposure in gastric cancer development. - It is valuable for understanding tumor multiplicity, inflammation-driven tumorigenesis, and potential intervention targets. |
| Ref. (30) | Wistar rats (SPF-grade, male, 100–130 g, 4-week-old) | Induction of precancerous lesions of gastric cancer using multiple factors: 120 μg/ml MNNG (a carcinogen) in water, 0.05% ranitidine in feed (acid suppression), 2% sodium salicylate gavage (mucosal injury), and irregular diet (abrosia twice weekly). This mimics chronic inflammation, gastric mucosal injury, and PPI abuse — all risk factors in human gastric carcinogenesis. | - SPF facility, 12 h light/dark cycle, temperature 22 ± 2°C, humidity 60 ± 5%. - Control group: normal diet and water. - Model group: exposed to MNNG, sodium salicylate, ranitidine, and irregular feeding. | - Histopathological analysis via H&E staining. - Inflammation scored using a 4-point scale. - Bodyweight monitoring. - Serum cytokine levels via Luminex 23-Plex assay. - Gut microbiota analyzed via 16S rRNA sequencing. - Untargeted metabolomics on feces using UPLC-Q-TOF/MS. | - Advantages:   - Simulates multiple risk factors for gastric cancer.  - Provides insights into the interaction between gut microbiota, inflammation, and carcinogenesis.   - Disadvantages:   - Complex induction process.  - Limited to rat model, may not fully translate to humans. | - Lesions assessed at week 32. - Histopathology revealed chronic gastritis with polymorphonuclear infiltration in mucosa, submucosa, and muscularis. - No time-resolved staging provided for progression from gastritis to dysplasia, only endpoint (32 weeks) reported. | - The full stomach was removed and examined. - The paper does not specify if the lesions occurred in antrum, corpus, or both; only generalized mucosal injury is described. | - The model is for precancerous lesions, not cancer. - No invasion or metastasis described; changes remain within mucosal and submucosal layers. | - Significant infiltration by polymorphonuclear cells (likely neutrophils), lymphocytes, and macrophages. - Cytokines elevated: IL-1β, IL-4, IL-6, IL-10, IFN-γ, TNF-α, M-CSF. CXCL1 decreased. - Key immune cell involvement includes macrophages, as M-CSF and CXCL1 are macrophage-related. | - Study of gastric precancerous pathology under multifactorial stress. - Investigation of microbiota–immune–metabolism interactions. - Screening of preventive or therapeutic agents for early gastric carcinogenesis. - Study of dietary and drug effects on gastric mucosal health. |
| Ref. (31) | Male Wistar rats (from Pasteur Institute of Iran). | - Rats received MNNG (N-methyl-N-nitro-N-nitrosoguanidine) in drinking water ad libitum for 34 weeks. - Sodium chloride (10%) was administered weekly for 6 weeks to promote carcinogenesis. - This chemical model mimics differentiated-type gastric adenocarcinoma development. | - Animals were housed under controlled temperature with a 12:12 h light/dark cycle and had free access to food and water. - Propolis-treated groups were given food supplemented with ethanol extract of propolis (EEP) two weeks prior to MNNG administration. | - Gross pathology (tumor incidence, number, size) - Histological scoring (nuclear/cytoplasmic ratio, nuclear dispolarity, structural abnormality) via H&E staining - Immunohistochemistry (IHC) for Bax, Bcl-2, β-catenin. | - Advantages:   Well-characterized MNNG model for gastric adenocarcinoma; EEP intervention shows measurable chemoprotective effect; molecular markers (Bax, Bcl-2, β-catenin) validated.   - Disadvantages:   Long study duration (36 weeks); geographic differences in propolis composition may limit reproducibility. | - Tumors were assessed at week 36. Lesions included dysplasia and adenocarcinoma. - No intermediate stages (e.g., intestinal metaplasia, atrophic gastritis) or exact timepoints before week 36 were detailed. | - Lesions were predominantly found in the stomach. - Fewer lesions appeared in the intestines or colon. - Metastases were primarily observed in para-cecal and para-aortic lymph nodes. | Metastatic lesions were found in para-cecal and para-aortic lymph nodes, indicating the model supports tumor invasion and dissemination. | There was no specific reporting on immune cell types (e.g., macrophages, neutrophils), though apoptosis-related changes are emphasized. | - Evaluation of chemoprotective effects of natural compounds - Study of gastric cancer pathogenesis - Biomarker validation (β-catenin, Bcl-2, Bax) - Testing plant-derived anticancer agents (e.g., CAPE in propolis) - Investigating apoptosis regulation in gastrointestinal cancers. |
| Ref. (32) | Male C57BL/6J mice and INS-GAS mice | - Mice were infected with *H. pylori* and exposed to cigarette smoke (CS) within 1 week of infection. - CS exposure was conducted 5 days/week for 8 weeks. - The principle is based on the interaction between *H. pylori* infection and CS exposure to study their combined effects on gastric inflammation, atrophy, and metaplasia development. | - Mice were administered standard chow and water ad libitum. - *H. pylori*-infected mice received two consecutive doses of 10^9^ bacteria spaced 24 hours apart via oral gavage. - CS exposure was conducted in whole-body inhalation chambers. | - Histological examination of gastric tissues using hematoxylin and eosin (H&E) staining. - Immunohistochemical analysis for markers such as cH2AX, Ki67, and CD44v9. - Flow cytometry to analyze immune cell subsets in the gastric corpus. - RNA-seq analysis to identify differentially expressed genes (DEGs) in the gastric corpus. - Quantitative PCR (qPCR) for specific gene expression and *H. pylori* density. | - Advantages:   - Provides insights into the combined effects of *H. pylori* infection and CS exposure on gastric pathology.  - Uses a well-established *H. pylori* infection model and CS exposure protocol.   - Disadvantages:   - Limited to a relatively short exposure duration (8 weeks).  - Focuses on male mice, limiting the generalizability to females.  - Uses a modest CS exposure level, which may not fully reflect long-term smoking effects. | - Mice were euthanized immediately after the final smoke exposure (8 weeks). - Gastric tissues were examined for inflammation, atrophy, metaplasia, and DNA damage. - Preneoplastic lesion development was assessed in INS-GAS mice after 2 months of exposure. | Gastric corpus and antrum were examined; metaplasia and dysplasia mainly described in the corpus. | - The study did not specifically investigate tumor invasion or metastasis. - However, preneoplastic lesions were advanced in CS-exposed INS-GAS mice. | - CS exposure significantly reduced *H. pylori*-induced leukocyte recruitment, including T cells, B cells, macrophages, eosinophils, and neutrophils. - Immunostaining and flow cytometry showed reduced immune cell infiltration in CS-exposed mice compared to filtered air (FA)-exposed controls. | - Investigating the mechanisms of interaction between *H. pylori* infection and CS exposure in gastric carcinogenesis. - Evaluating the effects of CS on *H. pylori*-induced inflammation and gastric pathology. - Assessing the potential benefits of screening and treatment for *H. pylori* infection in smokers to reduce gastric cancer risk. |

**Table 3:** Summary of Acute Chemical Injury Induction Models for Gastric Precancerous Lesions

| Studies | Animal Model | Modeling Method and Principle | Feeding Conditions | Model Evaluation Methods | Advantages and Disadvantages | Detailed Time Course of Pathological Stages | Anatomical Sites of Lesions | Tumor Location and Invasion/Metastasis | Inflammation and Immune Cell Infiltration | Application Scenarios for Models |
| --- | --- | --- | --- | --- | --- | --- | --- | --- | --- | --- |
| Ref. (33) | CD-1 rats and Sprague–Dawley rats | - DMP 777 was administered orally to CD-1 rats at doses of 200 mg/kg/day. - The principle is based on the ability of DMP 777 to act as a protonophore, specifically targeting parietal cell acid-secretory membranes, leading to reversible oxyntic atrophy. | - DMP 777 was formulated as a suspension in 0.5% methylcellulose and administered orally by gavage (5.0 mL/kg) once daily. - Rats were fasted for 24 hours before pyloric ligation for acid secretion assays. | - Histological and immunohistochemical analysis of gastric mucosa. - Measurement of serum gastrin levels by radioimmunoassay. - *In vivo* gastric acid output measurement in pylorus-ligated rats. - Aminopyrine accumulation in isolated rabbit parietal cells. - H^+^, K^+^-ATPase and pNPPase assays in isolated tubulovesicles. - Acridine orange accumulation in isolated tubulovesicles. - Electron microscopy of gastric mucosa. | - Advantages:   - Fully reversible oxyntic atrophy (unique among models).  - Specific parietal cell targeting without broad cytotoxicity.  - Mimics human chronic gastritis phenotypes (foveolar hyperplasia, metaplasia).   - Disadvantages:   - High drug doses required (200 mg/kg).  - No significant inflammatory infiltrate (unlike *H. pylori*-associated gastritis).  - No neoplastic progression observed (limited to pre-neoplastic changes). | - Acute Phase (1–10 days):   - Parietal cell necrosis (observed at 24 hours).  - Foveolar hyperplasia and BrdU⁺ progenitor cell expansion (by 2 days).  - Emergence of basal mucous metaplasia (by 10 days).   - Chronic Phase (3–6 months):   - Sustained oxyntic atrophy, foveolar hyperplasia.  - Loss of chief cells, ECL cells, and somatostatin cells.   - Recovery Phase (3 months post-withdrawal):   Complete restoration of normal mucosal lineages. | - Fundic mucosa (corpus) only. - Antral mucosa showed no histological changes except increased gastrin cell numbers. | No evidence of adenocarcinoma or metastatic potential, even after 6 months of dosing. | - Inflammatory Cells: Minimal infiltrate; only a slight increase in mucosal eosinophils noted during acute necrosis. - No significant mononuclear infiltrate (e.g., macrophages, lymphocytes) or cytokine-driven inflammation. | - Study of parietal cell biology and lineage differentiation. - Investigation of reversible oxyntic atrophy and metaplasia mechanisms. - Testing therapeutic interventions for gastric mucosal repair. - Not suitable for studying *H. pylori*-associated inflammation or neoplastic progression. |
| Ref. (34) | - Mist1^CreER/+^/Rosa26R^LacZ^ mice (for lineage tracing of chief cells). - C57BL/6 mice (for *Helicobacter felis* infection and L-635 treatment). - Wild-type controls. | Methods:  - DMP-777: Parietal cell-specific protonophore (200 mg/kg/day, oral gavage; no inflammation).  - L-635: Structurally related β-lactam compound (350 mg/kg/day, 3 days; induces inflammation).  - *H. felis* Infection: Chronic infection (6 months; chronic inflammation).   - Principle:   - Parietal cell loss triggers transdifferentiation of mature chief cells into SPEM (spasmolytic polypeptide-expressing metaplasia).  - Inflammation (L-635 or *H. felis*) accelerates SPEM expansion and proliferation. | - Diet: Regular mouse chow, water ad libitum. - Tamoxifen Induction: For lineage tracing, 3 doses (1 mg/0.1 mL corn oil, every other day). - Fasting: Overnight before *H. felis* inoculation or acid secretion studies. | - Lineage Tracing:   β-galactosidase (X-gal) staining in Mist1^CreER/+^/Rosa26R^LacZ^ mice to track chief cell-derived SPEM.   - Histopathology:   H&E, PAS, TFF2 (SPEM marker), H^+^/K^+^-ATPase (parietal cells), intrinsic factor (chief cells).   - Proliferation Markers:   MCM2, Ki-67, BrdU labeling.   - Inflammation Assessment:   - CD45 (lymphocytes), F4/80 (macrophages), MCA771G (neutrophils).  - Cytokine PCR: TNF-α, IL-1β, IL-4, IL-10, IFN-γ.   - Electron Microscopy: Parietal cell necrosis. | - Advantages:   - First direct evidence that SPEM arises from transdifferentiated chief cells.  - Reversible (DMP-777) and inflammatory (L-635/*H. felis*) models for comparative studies.   - Disadvantages:   - No intestinal metaplasia (unlike humans; mice develop only SPEM).  - No neoplastic progression in these models (limited to pre-neoplastic SPEM). | - DMP-777 (No Inflammation):   - Day 1–3: Parietal cell necrosis.  - Day 10–14: SPEM emerges at gland bases (chief cell-derived).   - L-635 (Acute Inflammation):   Day 3: Parietal cell loss + SPEM expansion (entire gland length).   - *H. felis* (Chronic Inflammation):   6 Months: SPEM dominates glands, proliferative (MCM2^+^/Ki-67^+^). | - Fundic mucosa (corpus) exclusively. - Antrum: No metaplasia; only G-cell hyperplasia (increased gastrin). | - No adenocarcinoma or metastasis reported in these models. - *H. felis* infection in C57BL/6 mice can progress to dysplasia after >9 months. | - DMP-777: Minimal inflammation (neutrophil elastase inhibition). - L-635:   - Acute infiltrate: Neutrophils, macrophages, T/B cells (CD45^+^, F4/80^+^, MCA771G^+^).  - Cytokines: ↑ IL-1β, IL-10.   - *H. felis*:   - Chronic infiltrate: Lymphocytes, macrophages.  - Cytokines: ↑ TNF-α, IL-1β, IL-4. | - Study chief cell plasticity and transdifferentiation. - Investigate inflammatory drivers of metaplasia. - Test interventions to block SPEM progression. - Not suitable for studying intestinal metaplasia or late-stage carcinogenesis. |
| Ref. (35) | - Primary Strains:   - C57BL/6 (most commonly used).  - BALB/c (more sensitive to tamoxifen toxicity).  - FVB/N.   - Age: 6–8 weeks (effective up to 6 months, but response diminishes with age). | - Method: Intraperitoneal (IP) injections of tamoxifen (5 mg/20 g body weight for 3 consecutive days). - Principle:   - Tamoxifen induces acute parietal cell apoptosis (>90% loss).  - Triggers chief cell transdifferentiation into SPEM (spasmolytic polypeptide-expressing metaplasia).  - Reversible upon discontinuation (normal histology restored within weeks).   - Key Feature: Estrogen-independent mechanism (unrelated to hormonal effects). | - Diet: Standard mouse chow, water ad libitum. - Tamoxifen Preparation:   - Dissolved in 10% ethanol/90% sunflower seed oil.  - Stored at 4°C (3 days) or –20°C (long-term). | - Histopathology:   - H&E: Parietal cell loss, apoptotic bodies.   - Immunostaining:   - H^+^/K^+^-ATPase (parietal cells).  - GSII lectin (mucous neck cells; shifts to gland bases post-treatment).  - TFF2 (SPEM marker). | - Advantages:   - Rapid induction (SPEM by 3 days).  Reversible (unlike *Helicobacter* models).  - Minimal inflammation (unlike L-635 or *H. felis*).   - Disadvantages:   - BALB/c mice may die from toxicity.  - No neoplastic progression (limited to pre-neoplastic SPEM). | - Day 1–3:   - Acute parietal cell apoptosis.  - Chief cell reprogramming begins.   - Day 3:   - SPEM fully developed (TFF2^+^ cells at gland bases).  - Progenitor cell proliferation increases.   - Post-treatment (weeks):   Complete histological recovery. | - Fundic mucosa (corpus) exclusively. - Antrum: No metaplasia or inflammation reported. | - No adenocarcinoma or metastasis reported. - Tamoxifen alone induces only SPEM; chronic inflammation (e.g., Helicobacter) is required for dysplasia*.* | - Inflammatory Infiltrate: Scant (minimal immune cell recruitment). - Cytokines: Not profiled in this study (unlike L-635/*H. felis* models). | - Study acute parietal cell loss and SPEM initiation. - Investigate reversibility of metaplasia. - Not suitable for studying chronic inflammation-driven carcinogenesis. |

**Table 4:** Summary of Gastrin-Related Animal Models for Gastric Precancerous Lesions

| Studies | Animal Model | Modeling Method and Principle | Feeding Conditions | Model Evaluation Methods | Advantages and Disadvantages | Detailed Time Course of Pathological Stages | Anatomical Sites of Lesions | Tumor Location and Invasion/Metastasis | Inflammation and Immune Cell Infiltration | Application Scenarios for Models |
| --- | --- | --- | --- | --- | --- | --- | --- | --- | --- | --- |
| Ref. (36) | - Wild-type (WT): C57BL/6 - Gastrin-deficient (G^−/−^): 129/Sv crossed with C57BL/6 - Somatostatin-deficient (SOM^−/−^): C57BL/6 | - Method: Genetic knockout of the gastrin gene (G^−/−^ mice). - Principle:   - Gastrin deficiency leads to hypochlorhydria (low stomach acid), causing bacterial overgrowth and chronic inflammation.  - Chronic inflammation progresses to parietal cell atrophy, intestinal metaplasia, dysplasia, and finally adenocarcinoma in the antrum.  - The model recapitulates the human Correa cascade (chronic gastritis → atrophy → metaplasia → dysplasia → cancer). | - Mice were housed in sterile microisolator cages in non-barrier conventional rooms. - No specific diet mentioned; standard laboratory conditions were maintained. | - Gross morphology & histology: H&E staining for atrophy, metaplasia, dysplasia, and tumor formation. - Immunohistochemistry (IHC):   - Ki67 (proliferation), TUNEL (apoptosis), MUC2 & villin (intestinal metaplasia).  - CD4^+^ T cells and polymorphonuclear neutrophils (PMNs) for inflammation.   - Western blot: RUNX3 (tumor suppressor), STAT3/STAT1 phosphorylation (oncogenic signaling). - Functional assay: Anchorage-independent growth in soft agar (confirms neoplastic transformation). - Gastric acidity & plasma gastrin levels: Measured via titration and radioimmunoassay (RIA). | - Advantages:   - Recapitulates human gastric cancer progression (Correa cascade).  - No *Helicobacter* infection required (unlike INS-GAS model).  - Functional validation via soft agar assay confirms transformation.   - Disadvantages:   - Slow progression (tumors develop by 12 months).  - No metastasis observed (tumors remain localized). | - Chronic gastritis: Starts early (exact time not specified, but inflammation is present before atrophy). - Parietal cell atrophy: Observed by 12 months. - Intestinal metaplasia: Confirmed by MUC2 & villin expression at 12 months. - Dysplasia & adenocarcinoma: Developed in 60% of G^−/−^ mice by 12 months. | - Atrophy & metaplasia: Fundus (acid-secreting region). - Tumors: Exclusively in the antrum (6/10 G^−/−^ mice). - SOM^−/−^ mice showed fundic hyperplasia but no tumors. | - Local invasion: Submucosal invasion observed. - No metastasis: No tumor spread to other organs. | - Inflammation present: Due to bacterial overgrowth from hypochlorhydria. - Immune cell infiltration:   - Polymorphonuclear neutrophils (PMNs): Increased in the antrum.  - CD4^+^ T cells: Elevated in the antrum.  - IFNγ levels: Significantly higher in tumor tissue (linked to STAT3 activation). | - Mechanistic studies: Role of inflammation (IFNγ/STAT3) in gastric cancer. - Pre-cancerous progression: Studying atrophy, metaplasia, and dysplasia. - Therapeutic testing: Anti-inflammatory or STAT3-targeting drugs. - Comparative studies: Contrasting with *Helicobacter*-dependent models (e.g., INS-GAS). |
| Ref. (37) | - Wild-type (WT): C57BL/6 - Gastrin-deficient (Gastrin KO): Generated by targeted disruption of the gastrin gene, maintained on a C57BL/6 background. | - Method: Acute oxyntic atrophy was induced using DMP-777, a parietal cell-specific protonophore that ablates parietal cells. - Principle:   - Parietal cell loss leads to hypochlorhydria, triggering hypergastrinemia in WT mice.  - Foveolar hyperplasia (surface mucous cell expansion) occurs in WT mice due to gastrin-driven proliferation.  - Spasmolytic Polypeptide-Expressing Metaplasia (SPEM) arises from transdifferentiation of chief cells, independent of gastrin.  - Gastrin-deficient mice lack foveolar hyperplasia but develop accelerated SPEM (within 1 day post-DMP-777). | - Mice were maintained on regular mouse chow in a temperature-controlled room with a 12:12-h light-dark cycle. - DMP-777 was administered orally (350 mg/kg/day) as a gavage suspension in 0.5% methylcellulose. | - Histology & Immunohistochemistry (IHC):   - H&E staining for parietal cell atrophy, foveolar hyperplasia, and SPEM.  - DR-PAS staining for surface mucous cells.  - BrdU labeling to assess proliferation.  - TFF2 (SPEM marker) and intrinsic factor (IF, chief cell marker) staining.   - Serum gastrin measurement: Radioimmunoassay (RIA) confirmed hypergastrinemia in WT mice. - In situ hybridization & qRT-PCR: Quantified TFF2 and IF mRNA expression in laser-capture microdissected fundic cells. - Double immunofluorescence: Identified cells co-expressing TFF2 and IF (transdifferentiation evidence). | - Advantages:   - Rapid induction of oxyntic atrophy (within 2 days).  - Distinguishes gastrin-dependent (foveolar hyperplasia) and gastrin-independent (SPEM) pathways.  - Recapitulates human metaplastic progression (e.g., SPEM as a pre-neoplastic lesion).   - Disadvantages:   - No progression to dysplasia or cancer in this acute model (long-term studies needed).  - SPEM reversal occurs in WT mice post-treatment but persists in gastrin-deficient mice. | - Parietal cell loss:   - WT mice: 72% reduction by Day 1, 85–87% by Day 3.  - Gastrin KO mice: 30% reduction by Day 1, 66% by Day 3.   - Foveolar hyperplasia (WT only):   Significant increase in DR-PAS+ cells by Day 1, peaks at Day 3 (219% of baseline).   - SPEM emergence:   - WT mice: Appears at Day 7, peaks at Day 14.  - Gastrin KO mice: Detected by Day 1, sustained through Day 14.   - Recovery:   - WT mice fully recover parietal cells and normalize SPEM by 14 days post-treatment.  - Gastrin KO mice show incomplete SPEM resolution. | - Atrophy & hyperplasia: Fundic mucosa (acid-secreting region). - SPEM: Base of fundic glands (transdifferentiated chief cells). | This study focused on acute metaplastic changes; no tumors or metastasis observed. | - No significant inflammation reported in this acute model. - DMP-777 is a neutrophil elastase inhibitor, but neutrophil infiltration was not highlighted. - Chronic inflammation models (e.g., *H. felis* infection) were referenced for SPEM-cancer progression but not studied here. | - Mechanistic studies: Roles of gastrin and parietal cell-derived factors in lineage differentiation. - Pre-neoplastic metaplasia: SPEM as a precursor to gastric cancer. - Therapeutic testing: Targeting gastrin pathways or metaplastic transitions. - Comparative studies: Contrasting with *Helicobacter*-dependent models (e.g., INS-GAS mice). |
| Ref. (38) | - Wild-type (WT): FVB and C57BL/6 backgrounds. - Hypergastrinemic (INS-GAS): FVB background, overexpressing human amidated gastrin. - Gastrin-deficient (GAS⁻/⁻): Generated by targeted disruption of the gastrin gene. - TFF1-deficient (Tff1⁻/⁻ and Tff1⁺/⁻): C57BL/6 background, with homozygous or heterozygous loss of TFF1. | - Method: Gastric carcinogenesis was induced using N-methyl-N-nitrosourea (MNU), a DNA alkylating agent, with or without *Helicobacter felis* infection. - Principle:   - MNU induces epigenetic silencing of TFF1 (a tumor suppressor) via histone modifications (H3K9 methylation, H3 deacetylation) and promoter hypermethylation.  - Gastrin counteracts this silencing by reversing TFF1 repression, thereby suppressing tumor initiation and progression.  - *H. felis* infection exacerbates TFF1 silencing and tumorigenesis, mimicking human *H. pylori*-associated gastric cancer. | - Mice were housed under standard conditions with regular chow. - MNU was administered in drinking water (five 1-week courses). - *H. felis* infection was introduced via oral gavage. | - Histopathology: H&E staining to assess tumor grade (hyperplasia, adenoma, adenocarcinoma). - Immunohistochemistry (IHC):   - Ki-67 (proliferation), cleaved caspase-3 (apoptosis), Dclk1 (progenitor cell marker).  - TFF1 protein expression in antral mucosa and tumors.   - Epigenetic Analysis:   - Bisulfite sequencing for TFF1 promoter DNA methylation.  - Chromatin immunoprecipitation (ChIP) for histone marks (H3K9me2/3, H3ac).   - Gene Expression:   qRT-PCR for TFF1, gastrin, and other markers in isolated epithelial cells.   - Serum Gastrin & Gastric pH: Measured to correlate hormonal effects with tumor suppression. | - Advantages:   - Demonstrates direct hormonal regulation of epigenetics in cancer suppression.  - Recapitulates human intestinal-type gastric cancer progression (TFF1 loss → metaplasia → adenocarcinoma).  - Combines chemical (MNU) and infectious (*H. felis*) carcinogenesis models.   - Disadvantages:   - MNU alone does not induce inflammation, requiring *H. felis* for full pathological mimicry.  - No metastasis observed; limited to local antral tumors. | - Early Stage (18 weeks post-MNU):   - TFF1 repression via H3K9 methylation (no DNA methylation yet).  - Increased proliferation (Ki-67^+^) and progenitor cells (Dclk1^+^) in WT mice.   - Late Stage (36 weeks post-MNU):   - TFF1 promoter hypermethylation in advanced tumors.  - Adenocarcinomas in WT mice; suppressed tumors in INS-GAS mice.   - Gastrin-Deficient Mice (GAS⁻/⁻):   Develop microadenocarcinomas by 20 weeks (vs. WT at 36 weeks). | Exclusively antral (pyloric region); corpus tumors were rare and unrelated to TFF1. | - Local invasion observed in advanced adenocarcinomas. - No metastasis reported. | No significant increase in tissue infiltration by neutrophils or mononuclear cells observed in MNU-treated mice. | - Testing potential therapeutic interventions targeting gastrin or Tff1 pathways. - Investigating the role of epigenetic modifications in cancer initiation and progression. - Developing strategies to prevent gastric cancer through modulation of gastrin levels or Tff1 expression. |
| Ref. (39) | - Primary Strain: INS-GAS transgenic mice (expressing human gastrin under the rat insulin I promoter). - Control Strain: Wild-type FVB/N mice. | - Method:   - Hypergastrinemia: INS-GAS mice exhibit chronic moderate hypergastrinemia due to pancreatic secretion of human gastrin (G-17).  - *Helicobacter* Infection: Accelerated model involves inoculation with Helicobacter felis at 4 weeks of age.   - Principle:   - Hypergastrinemia initially increases acid secretion and parietal cell numbers but later induces parietal cell loss, atrophy, and metaplasia.  - *H. felis* infection synergizes with hypergastrinemia to accelerate atrophy and neoplastic progression via inflammation and growth factor upregulation (e.g., TGF-α, HB-EGF). | - Housing: Microisolator, solid-bottomed polycarbonate cages. - Diet: Standard pelleted diet, water ad libitum. - Infection Protocol: *H. felis* (ATCC 49179) inoculated at 4 weeks; infection confirmed via ELISA (IgG antibodies) and gastric urease assays. | - Physiological:   - Serum gastrin levels (RIA).  - Gastric acid secretion (pyloric ligation model, titration with NaOH).   - Histopathological:   - H&E, alcian blue/PAS staining for metaplasia.  - Immunocytochemistry (parietal cells: H^+^/K^+^-ATPase; ECL cells: histamine; proliferation: BrdU).  - Electron microscopy for carcinoma confirmation.   - Molecular:   - Western blot (CCK-B receptor, HB-EGF, TGF-α).  - RIA for TGF-α in mucosal extracts. | - Advantages:   - Recapitulates human gastric carcinogenesis stages (metaplasia → dysplasia → invasive cancer).  - Synergy with *H. felis* mimics *H. pylori*-associated human gastric cancer.  - No surgical/drug-induced hypochlorhydria (avoids confounding variables).   - Disadvantages:   - Slow progression in uninfected INS-GAS mice (~20 months for cancer).  - Limited metastasis (no lymph node/distant organ involvement reported).  - Mouse-specific gastrin processing (low G-gly levels) may not fully mirror human pathophysiology. | - Uninfected INS-GAS Mice:   - 1–4 months: Increased acid secretion, parietal/ECL cell hyperplasia.  - 5–12 months: Declining acid secretion, parietal cell loss, foveolar hyperplasia.  - 14–20 months: Gastric atrophy, metaplasia (intestinal mucin phenotype), dysplasia.  - >20 months: Intramucosal carcinoma (13%), invasive carcinoma (6/8 mice).   - *H. felis*-Infected INS-GAS Mice:   -6–7 months post-infection: Severe atrophy, dysplasia, intramucosal carcinoma (85%), submucosal (54%), and intravascular invasion (46%). | - Primary Site: Fundus/corpus (acid-secreting region). - Metaplasia/Dysplasia: Expands from glandular base to surface. - Cancer: Invades submucosa and vasculature. | Invasive gastric carcinoma observed, but no evidence of metastasis to lymph nodes or other tissues. | - *H. felis* infection leads to significant colonization and inflammation in the gastric mucosa. - Increased expression of TGF-β and HB-EGF associated with immune response and tissue remodeling. | - Study gastrin’s role in gastric carcinogenesis. - Investigate *Helicobacter*-host interactions in cancer progression. - Test chemopreventive/therapeutic agents targeting atrophy or metaplasia. - Model for *H. pylori*-induced human gastric cancer. - Explore growth factor pathways (e.g., TGF-α/HB-EGF) as therapeutic targets. |
| Ref. (40) | - Primary Strain: INS-GAS transgenic mice (FVB/N background), expressing human gastrin under the rat insulin I promoter. - Control Strain: Wild-type FVB/N mice. - Additional Strain: C57BL/6 mice (used for gender comparison in *H. felis* infection). | - Method:  1. Hypergastrinemia: Chronic moderate hypergastrinemia due to pancreatic secretion of human gastrin (G-17). 2. *Helicobacter Infection*:   *- H. pylori* strains: Wild-type B128 (cag⁺) and isogenic cagE⁻ mutant.  *- H. felis* (positive control for accelerated carcinogenesis).   1. Gender-Specific Analysis: Male and female mice infected at 4 weeks of age.  - Principle:   - Synergy between hypergastrinemia and Helicobacter infection accelerates gastric carcinogenesis.  - cagE (a cag pathogenicity island gene) and host IL-1β polymorphisms modulate inflammation and cancer progression. | - Housing: Specific pathogen-free, microisolator cages. - Diet: Standard pelleted diet, water ad libitum. - Infection Protocol:   - Oral gavage with *H. pylori* (10¹⁰ CFU/mL) or *H. felis*.  - Infection confirmed via ELISA (IgG), culture, PCR, and histology (Warthin-Starry stain). | - Histopathology:   - H&E and Alcian blue staining for metaplasia/dysplasia.  - Injury scores (0–4 scale) for inflammation, atrophy, metaplasia, and dysplasia.   - Bacterial Load:   Quantitative culture, real-time PCR (ureB gene), and fluorescent in situ hybridization (FISH).   - Cytokines:   -IL-1β levels measured by ELISA in gastric mucosa.   - Immunohistochemistry:   *H. pylori* identification (polyclonal anti-*H. pylori* IgG). | - Advantages:   - Recapitulates human intestinal-type gastric cancer progression (inflammation → metaplasia → dysplasia → carcinoma).  - Gender disparity mirrors human epidemiology (male predominance).  - Evaluates microbial (cagE) and host (IL-1β, gender) interactions.   - Disadvantages:   - Cancer primarily in corpus (vs. human antrum-predominant).  - No metastasis observed.  - Transient IL-1β response complicates mechanistic interpretation. | - Uninfected INS-GAS Mice:   24 weeks: Mild-moderate corpus gastritis (transgene effect), no cancer.   - *H. pylori*-Infected INS-GAS Mice (Male):  1. 6 weeks: Corpus inflammation, atrophy, intestinal metaplasia, dysplasia. 2. 12 weeks: Increased IL-1β levels. 3. 24 weeks:   - Wild-type B128: Carcinoma in situ (100%), invasive carcinoma (40%).  - B128 cagE⁻: Delayed progression but same endpoint (invasive carcinoma in 0% at 24 weeks, though dysplasia present).   - *H. felis*-Infected INS-GAS Mice (Male):   24 weeks: Adenocarcinoma (100%), submucosal/vascular invasion (78%).   - Female Mice:   No cancer at 24 weeks, regardless of infection. | - Primary Site: Gastric corpus (proximal to cardia). - Antrum: Minimal inflammation/injury. - Metaplasia/Dysplasia: Expands from corpus/cardia junction. | - Invasiveness:   - Submucosal and vascular invasion in *H. felis*-infected males (78%).  - Less frequent in *H. pylori*-infected males (40% wild-type, 0% cagE⁻).   - Metastasis: None reported. | - Inflammation:   - Cells: Lymphocytes (dominant), polymorphonuclear cells (near epithelium).  - Cytokines: IL-1β peaks at 12 weeks, declines by 24 weeks (Figure 8A).   - Gender Difference:   - Males: Higher injury scores, IL-1β-independent.  - Females: Lower inflammation, no cancer. | - Study *H. pylori* virulence (cag island) and host (IL-1β, gender) interactions. - Test chemopreventive agents targeting inflammation or metaplasia. - Model for gender disparity in gastric cancer. - Explore IL-1β or gastrin as therapeutic targets. |
| Ref. (41) | - Primary Strain: INS-GAS transgenic mice (FVB/N background), expressing human gastrin under the rat insulin I promoter. - Experimental Groups:   - Germ-free (GF)  - Monoassociated with *H. pylori* (mHp)  - Restricted Altered Schaedler’s Flora (rASF; 3 species: *Clostridium* ASF356, *Lactobacillus murinus* ASF361, *Bacteroides* ASF519)  - rASF + *H. pylori* (rASFHp)  - Intestinal flora (IF; undefined specific pathogen-free microbiota)  - IF + *H. pylori* (IFHp) | - Method:  1. Hypergastrinemia: Chronic moderate hypergastrinemia due to pancreatic secretion of human gastrin (G-17). 2. Microbial Colonization:   - rASF or IF alone, or co-colonized with *H. pylori*.  - *H. pylori* strain: Not specified in the abstract, but methods describe experimental infection.   1. Gender-Specific Analysis: Male and female mice compared.  - Principle:   - Gastric colonization with commensal microbiota (rASF or IF) synergizes with *H. pylori* to promote inflammation, atrophy, and neoplasia.  - rASF mimics the pro-carcinogenic effects of diverse IF, suggesting microbial diversity is less critical than colonization efficiency. | - Housing: Gnotobiotic (GF, rASF, mHp) or specific pathogen-free (IF, IFHp) conditions. - Diet: Standard pelleted diet, water ad libitum. - Infection Protocol:   - Oral gavage with *H. pylori* or rASF/IF.  - Necropsy at 7 months post-infection (mpi). | - Histopathology:   - Gastric Histologic Activity Index (GHAI) scoring (0–4 scale) for inflammation, atrophy, metaplasia, dysplasia, and neoplasia.  - H&E and Alcian blue staining.   - Microbial Load:   qPCR for *H. pylori* (ureB gene) and ASF species.   - Immune/Inflammatory Markers:   - mRNA expression of proinflammatory genes (IL-17, Tnf-α, Nos2, Cxcl1, Ccl2) and cancer-related genes (IL-11, Ptger4, Tgf-β).  - Serum cytokines (ELISA).   - Serology:   IgG responses to *H. pylori* outer membrane antigens. | - Advantages:   - Recapitulates human gastric carcinogenesis stages (inflammation → metaplasia → dysplasia → carcinoma).  - Demonstrates gender disparity (male predominance) mirroring human epidemiology.  - Isolates effects of specific commensals (rASF) vs. diverse microbiota.   - Disadvantages:   - No metastasis observed.  - Limited to corpus-predominant lesions (vs. human antrum-predominant cancer).  - Transient IL-1β response complicates mechanistic interpretation. | - Uncolonized (GF) or rASF/IF Alone:   7 mpi: Mild-moderate gastritis, atrophy, and dysplasia (no neoplasia).   - *H. pylori*-Infected (mHp):   7 mpi: Severe gastritis, atrophy, and dysplasia (no neoplasia).   - Co-Colonized (rASFHp or IFHp):  1. 7 mpi: 2. Male Mice:   - rASFHp: 46% high-grade dysplasia (GIN), 23% invasive carcinoma.  - IFHp: 53% GIN, 40% invasive carcinoma.   1. Female Mice: No GIN or carcinoma. | - Primary Site: Gastric corpus (proximal to cardia). - Antrum: Minimal pathology. | - Invasiveness:   Submucosal and intramucosal invasion in male rASFHp (23%) and IFHp (40%) mice.   - Metastasis: None reported. | - Inflammation:   - Cells: Lymphocytes (dominant), polymorphonuclear cells (near epithelium).  - Cytokines: Elevated IL-17, Tnf-α, Nos2, Cxcl1, and IL-11 in males.   - Gender Difference:   - Males: Higher inflammation, IL-11, and Tgf-β expression.  - Females: Lower inflammation, no neoplasia. | - Study host-microbe interactions in gastric carcinogenesis. - Test interventions targeting specific commensals or inflammatory pathways. - Model for microbiome-driven cancer risk in *H. pylori*-infected patients with gastric atrophy. - Explore gender-specific therapies (e.g., estrogen modulation). |

**Table 5:** Summary of Inflammatory Mediator–Induced Animal Models for Gastric Precancerous Lesions

| Studies | Animal Model | Modeling Method and Principle | Feeding Conditions | Model Evaluation Methods | Advantages and Disadvantages | Detailed Time Course of Pathological Stages | Anatomical Sites of Lesions | Tumor Location and Invasion/Metastasis | Inflammation and Immune Cell Infiltration | Application Scenarios for Models |
| --- | --- | --- | --- | --- | --- | --- | --- | --- | --- | --- |
| Ref. (42) | - Primary Strain: C57BL/6J - Transgenic Lines:   - Line 19: High-expressing hIL-1β  - Line 42: Low-expressing hIL-1β   - Crossed Strains:   - IL-1β;NF-κBEGFP: IL-1β transgenic × NF-κBEGFP knockin mice (C57BL/6J background).  - IL-1β;Rag2⁻/⁻: IL-1β transgenic × Rag2⁻/⁻ mice (C57BL/6J background). | - Method:   - Stomach-specific overexpression of human IL-1β via the H/K-ATPase β-subunit promoter (targeting parietal cells).  - Optional *Helicobacter felis* (*H. felis*) infection to accelerate carcinogenesis.   - Principle:   - IL-1β activates NF-κB in myeloid-derived suppressor cells (MDSCs), inducing proinflammatory cytokines (IL-6, TNF-α) and chemokines (SDF-1).  - Chronic inflammation drives progression from gastritis → atrophy → metaplasia → dysplasia → adenocarcinoma. | - Standard Diet: Not explicitly stated, but mice were housed in AAALAC-approved facilities at Columbia University. - Infection Protocol:   *H. felis*: Administered by oral gavage (1 × 10⁸ CFU, every other day for 3 doses). | - Histopathology: H&E staining for gastritis, hyperplasia, atrophy, metaplasia, dysplasia, and carcinoma. - Immunohistochemistry (IHC):   β-catenin, c-Myc, NF-κB p65, TFF2/SP, F4/80^+^ macrophages, Gr-1^+^CD11b^+^ MDSCs.   - Flow Cytometry (FACS):   Quantification of MDSCs (CD11b^+^Gr-1^+^), T cells (CD4^+^/CD8^+^), macrophages (F4/80^+^).   - ELISA:   Cytokines (IL-1β, IL-6, TNF-α, SDF-1) in serum/stomach tissue.   - RT-PCR/qPCR:   mRNA levels of IL-1β, NF-κB target genes, and inflammatory markers.   - Drug Interventions:   IL-1RA (IL-1 receptor antagonist) or NF-κB inhibitor (Bay 11-7085) to block progression. | - Advantages:   - Provides a direct link between IL-1β overexpression and gastric cancer development. - Demonstrates the role of MDSCs in early stages of carcinogenesis.  - Offers a model for testing anti-inflammatory and anti-cancer therapies.   - Disadvantages:   - The study focuses on a single cytokine (IL-1β) and may not fully represent the complexity of human gastric cancer.  - The model does not fully replicate human gastric cancer, particularly in terms of anatomic location and progression. | - 2 months: Early signs of gastritis and increased MDSCs in peripheral blood, spleen, and stomach. - 6 months: Development of chronic atrophic gastritis and increased MDSCs. - 12 months: High-grade dysplasia and carcinoma observed in some IL-1β transgenic mice. - *H. felis* Infection: Accelerated progression to gastric atrophy and cancer within 5-12 months. | - Primary Site: Stomach body (targeted by H/K-ATPase promoter). - No mention of antrum-specific lesions. | Invasive gastric cancer observed in some IL-1β transgenic mice, particularly those infected with *H. felis*. | Key Inflammatory Cells:  - MDSCs (CD11b^+^Gr-1^+^): Dominant early infiltrate (2 months).  - Macrophages (F4/80^+^): Increased in later stages (6–12 months).  - Neutrophils (p40-Phox+): Present but less prominent.   - Cytokines: Elevated IL-6, TNF-α, SDF-1 in stomach/serum. | - Studying the role of IL-1β in gastric inflammation and cancer development. - Investigating the activation and function of MDSCs in chronic inflammation and cancer. - Evaluating the effects of IL-1β inhibition on gastric cancer progression. |
| Ref. (43) | - Primary Strain: C57BL/6J (backcrossed from SJL × C57BL/6 F2 embryos). - Transgenic Lines:   - H/K-IFN-γ^944^: High IFN-γ expression.  - H/K-IFN-γ^53^: Lower IFN-γ expression.   - Promoter: H^+^/K^+^ ATPase β subunit (targets IFN-γ to gastric parietal/pre-parietal cells). - Crossed Strains:   - H/K-IFN-γ;Rag1^−/−^: To assess T/B-cell independence.  - H/K-IFN-γ;Gli1-lacZ: To monitor Hedgehog (Hh) pathway activity. | - Method:   - Stomach-specific overexpression of murine IFN-γ via the H/K ATPase β promoter.  - Optional *Helicobacter felis* (*H. felis*) infection (oral gavage, 10^8^ CFU, 3 doses).   - Principle:   - IFN-γ drives chronic inflammation (Th1-polarized immune response).  - Induces pSTAT1/pSTAT3 signaling in epithelial/stromal cells, upregulating pro-inflammatory cytokines (IL-6, IL-1β, TNF-α).  - Leads to parietal/chief cell atrophy, spasmolytic polypeptide-expressing metaplasia (SPEM), and dysplasia. | - Standard Diet: Not specified, but mice were housed under standard conditions at the University of Michigan. - Infection Protocol:   *H. felis*: Administered at 2 months of age (3 oral gavages over 3 days)*.* | - Histological Analysis: Examination of gastric tissues for inflammation, atrophy, metaplasia, dysplasia, and tumor development. - Immunostaining: Detection of cell markers (e.g., Ki-67, cleaved caspase 3, TFF2, H/K-ATPase, intrinsic factor) and signaling molecules (e.g., pSTAT1, pSTAT3). - RT-qPCR: Quantification of mRNA expression for IFN-γ target genes and cytokines. - Immunoblotting: Detection of protein expression for proliferation, differentiation, and cell death markers. - Flow Cytometry: Analysis of myeloid-derived suppressor cells (MDSCs). | - Advantages:   - Provides a model for studying the role of IFN-γ in gastric tumorigenesis.  - Demonstrates the progression from inflammation to dysplasia and tumor development.  - Highlights the importance of IFN-γ in driving preneoplastic changes.   - Disadvantages:   - The model does not fully replicate human gastric cancer, particularly in terms of anatomic location and progression.  - The study focuses on a single cytokine (IFN-γ) and may not fully represent the complexity of human gastric cancer. | - Chronic Gastritis: 7 weeks; inflammation, myeloid cell infiltration. - SPEM/Atrophy: 5–6 months; parietal/chief cell loss, TFF2^+^ metaplasia. - Dysplasia: 3–5 months; 46–50% of mice; progresses to 65% by 12 months. - Antral Tumors: >12 months; 10% developed polyps/adenocarcinoma (β-catenin^+^). | - Primary Site: Gastric corpus (IFN-γ targeted to parietal cells). - Antrum: Advanced lesions (polyps/adenocarcinoma) occurred here despite corpus-specific transgene expression. | Advanced lesions including adenocarcinomas were observed in the antrum of some H/K-IFN-γ mice. | - Significant increase in inflammatory cells (T cells, macrophages, neutrophils) and MDSCs in the gastric corpus. - Activation of STAT1 and STAT3 signaling pathways in epithelial and mesenchymal cells. - Upregulation of pro-inflammatory cytokines (IL-6, TNF-α, IL-1β). | - Studying the role of IFN-γ in gastric inflammation and cancer development. - Investigating the activation and function of MDSCs in chronic inflammation and cancer. - Evaluating the effects of IFN-γ inhibition on gastric cancer progression. |
| Ref. (44) | - Primary Strain: C57BL/6 (backcrossed from C3H × C57BL/6 F1 hybrids). - Transgenic Lines:   K19-C2mE-2 and K19-C2mE-8: Both express COX-2 and microsomal prostaglandin E synthase-1 (mPGES-1) under the cytokeratin 19 (K19) promoter (targets gastric epithelial cells). | - Method:   - Simultaneous overexpression of COX-2 and mPGES-1 in gastric epithelial cells to elevate PGE₂ production.  - Optional antibiotic treatment (streptomycin + cefoperazone) or *Helicobacter felis* infection to modulate bacterial flora.   - Principle:   - PGE₂ recruits macrophages to gastric mucosa.  - Bacterial TLR4 activation (via LPS) on epithelial cells induces proinflammatory cytokines (TNF-α, IL-1β, IL-6), activating macrophages.  - Macrophage-derived cytokines drive epithelial hyperplasia, metaplasia, and tumorous growth. | - Standard Diet: Not specified, but mice were housed under standard conditions. - Interventions:   - Antibiotics: Streptomycin (5 mg/mL in water) + cefoperazone (100 mg/kg/day, s.c.) for 3 weeks.  - COX-2 inhibitor: NS-398 (10 mg/kg/day, s.c.) for 4 weeks.  - *H. felis* infection: Oral gavage (5 × 10⁸ CFU). | - Histopathology: H&E staining for hyperplasia, metaplasia, tumors. - Immunohistochemistry (IHC):   - COX-2, mPGES-1, F4/80 (macrophages), TNF-α, CXCL14 (chemokine).  - Proliferation: BrdU labeling.   - ELISA/RIA:   PGE₂ levels in gastric mucosa.   - RT-PCR/qPCR:   Cytokines (TNF-α, IL-1β, IL-6), chemokines (CXCL14, MIP-2), TLR4.   - Bacterial Counts: Aerobic/microaerophilic/anaerobic cultures. | - Advantages:   - Recapitulates PGE₂-driven inflammation and macrophage recruitment in gastric tumorigenesis.  - Demonstrates TLR4-dependent macrophage activation by gastric flora.  - Validates COX-2/mPGES-1 as therapeutic targets (NS-398 suppresses hyperplasia).   - Disadvantages:   - No invasive carcinoma: Tumors were hyperplastic/benign (no metastasis).  - Limited tumor incidence: Tumors develop only in aged mice (>48 weeks).  - Antibiotic sensitivity: Phenotype partially dependent on bacterial flora. | - Aberrant differentiation: Detected at 12 weeks (elongated gastric pits, expansion of mucous cell population) - Hyperplasia: Increased BrdU labeling index detected at 12 weeks, significant gastric hypertrophy by 20 weeks - Tumor development: Large tumorous growths found at 48 weeks, characterized as benign metaplastic hyperplasia | - Primary Site: Proximal glandular stomach (targeted by K19 promoter). - Antrum: No significant lesions reported. | Tumors were metaplastic hyperplasia without submucosal invasion or metastasis. | - Inflammation: Present, with heavy macrophage infiltration and increased levels of proinflammatory cytokines (TNF-α, IL-1β, IL-6) and chemokines (CXCL14, MIP-2). - Inflammatory cells: Predominantly macrophages (F4/80-positive), with some dendritic cells (CD11c-positive) and T cells (CD3e-positive) in the submucosa. | - Study PGE₂-macrophage crosstalk in gastric tumorigenesis. - Investigate TLR4 signaling in epithelial-stromal interactions. - Test COX-2/mPGES-1 inhibitors or antibiotics for chemoprevention. - Model *H. pylori*-associated gastric cancer (PGE₂ synergy with infection). |
| Ref. (45) | - K19-Wnt1: Transgenic mice expressing Wnt1 in the gastric mucosa under the keratin 19 (K19) promoter. - K19-C2mE: Transgenic mice expressing cyclooxygenase-2 (COX-2) and microsomal prostaglandin E synthase-1 (mPGES-1) in the stomach, leading to elevated prostaglandin E2 (PGE₂) levels. - K19-Wnt1/C2mE: Compound transgenic mice generated by crossing K19-Wnt1 and K19-C2mE, simultaneously activating Wnt and PGE₂ pathways. | - Method:   - K19-Wnt1: Wnt1 expression driven by the K19 promoter suppresses epithelial differentiation, expanding undifferentiated progenitor cells.  - K19-C2mE: COX-2 and mPGES-1 expression increases PGE₂, inducing metaplasia (SPEM) and hyperplasia.  - K19-Wnt1/C2mE: Combined activation of Wnt and PGE₂ pathways converts preneoplastic lesions into dysplastic tumors.   - Principle:   - Wnt activation stabilizes β-catenin, promoting progenitor cell proliferation.  - PGE₂ induces metaplasia, inflammation, and angiogenesis, synergizing with Wnt to drive tumorigenesis. | Not explicitly described in the article. | - Histopathology: H&E staining for dysplasia, metaplasia, and tumor invasion. - Immunohistochemistry (IHC):   - β-catenin (nuclear/cytoplasmic accumulation).  - Ki-67 (proliferation marker).  - F4/80 (macrophage marker).  - von Willebrand factor (vWF; angiogenesis marker).   - BrdU Labeling: Proliferation index. - In Situ Hybridization: TFF2 expression for SPEM detection. - Immunoblotting: Levels of unphosphorylated β-catenin and COX-2. - Microvessel Density (MVD): Quantified via vWF staining. | - Advantages:   - Recapitulates human gastric carcinogenesis (metaplasia → dysplasia → invasive tumors).  - High tumor incidence (100% in K19-Wnt1/C2mE mice by 20 weeks).  - Models Wnt and PGE₂ pathway cooperation, relevant to *H. pylori*-associated cancer.   - Disadvantages:   - No metastasis observed (limited to local invasion).  - Requires compound transgenic strains, complicating breeding. | - 5 Weeks:   Mucous cell metaplasia (Alcian blue-positive SPEM) in both K19-C2mE and K19-Wnt1/C2mE mice.   - 7–18 Weeks:   - K19-Wnt1: Preneoplastic lesions (undifferentiated epithelium, macrophage accumulation).  - K19-Wnt1/C2mE: Dysplastic cells emerge within metaplastic lesions by 10 weeks.   - 20 Weeks:   K19-Wnt1/C2mE: Dysplastic tumors with adjacent SPEM; 50% show muscle layer invasion.   - 30–50 Weeks:   K19-Wnt1/C2mE: Tumors dominate; 75–100% invasion; no metastasis. | - Glandular Stomach: Includes corpus and antrum (specific sub-regions not specified). - Proximal Stomach: K19-C2mE mice show hyperplasia here. | - Invasiveness: Tumor invasion into the smooth muscle layers was observed in K19-Wnt1/C2mE mice. - Metastasis: No metastatic tumors were found in other tissues (lymph nodes, liver, lung, peritoneum). | - Macrophages:   - Abundant in K19-Wnt1/C2mE tumors and K19-Wnt1 preneoplastic lesions (F4/80^+^).  - Sparse in wild-type stomach. | - Study Wnt/PGE₂ crosstalk in gastric cancer. - Test chemopreventive agents (e.g., COX-2 inhibitors). - Investigate metaplasia-dysplasia sequence and tumor microenvironment (macrophages, angiogenesis). - Target PPARδ or EP2 receptors (Wnt/PGE₂ intermediates). - Validate anti-inflammatory strategies. |
| Ref. (46) | - COX-2 Transgenic (TG) Mice: Generated by inserting the full-length human COX-2 cDNA under the cytomegalovirus (CMV) promoter in C57BL/6 mice. - Wild-Type (WT) Littermates: Used as controls. | - Method:   - Chemical Carcinogenesis: Mice were treated with N-methyl-N-nitrosourea (MNU, 240 ppm) in drinking water for 10 weeks (alternate weeks).  - High-Salt Diet: 10% NaCl was administered weekly alongside MNU to some groups.   - Principle:   - COX-2 Overexpression: Promotes PGE₂ production, disrupting cell kinetics (increased proliferation/reduced apoptosis).  - High Salt: Induces chronic inflammation and inflammatory cytokines (TNF-α, IL-1β, IL-6, IFN-γ), synergizing with MNU. | - Standard Diet: Presumed unless specified. - High-Salt Diet: 10% NaCl weekly for 10 weeks (Groups C and F). - MNU: 240 ppm in drinking water, alternated weekly for 10 weeks. | - Histopathology: H&E staining for adenocarcinoma, atrophy, metaplasia, and inflammation (Sydney classification). - Immunohistochemistry (IHC):   - COX-2 expression (rabbit monoclonal antibody).  - Ki-67 (proliferation) and TUNEL (apoptosis).   - Molecular Analysis:   - PGE₂ levels (ELISA).  - Inflammatory cytokines (TNF-α, IFN-γ, IL-1β, IL-6, IL-10) via RT-PCR. | - Advantages:   - Recapitulates human gastric cancer progression (inflammation → metaplasia → adenocarcinoma).  - Demonstrates COX-2’s promotional (not initiatory) role in carcinogenesis.  - High-salt diet mimics a known epidemiological risk factor.   - Disadvantages:   - No additive effect of COX-2 and high salt observed.  - Lack of a NaCl-only control group (potential oversight in isolating salt effects).  - No metastasis reported (local tumors only). | - Week 0–10:   MNU and/or NaCl exposure.   - Week 50 (Endpoint):   - WT + MNU (Group B): 25% cancer incidence.  - WT + MNU + NaCl (Group C): 46.9% cancer.  - COX-2 TG + MNU (Group E): 47.5% cancer.  - COX-2 TG + MNU + NaCl (Group F): 48.1% cancer.   - Precancerous Changes:   Chronic inflammation, atrophy, and intestinal metaplasia precede adenocarcinoma. | Primarily in the glandular stomach. | - Invasiveness: Confirmed histologically (adenocarcinoma with glandular invasion). - Metastasis: Not observed in any group. | - Chronic Inflammation:   - Correlated with cancer incidence (highest in Groups C and F).  - Dominated by mononuclear infiltrates (lymphocytes, macrophages).   - Cytokines:   NaCl upregulated TNF-α, IFN-γ, IL-1β, IL-6, and IL-10.   - PGE₂: Elevated in COX-2 TG and NaCl-treated mice. | - Studying the molecular mechanisms of COX-2 and high-salt intake in gastric carcinogenesis - Investigating the role of chronic inflammation and PGE2 in gastric cancer development - Screening and testing potential chemopreventive agents for gastric cancer - Understanding the effects of dietary factors on gastric cancer risk |
| Ref. (47) | - MT-TGFα (Line MT100): Transgenic mice overexpressing human TGFα under the metallothionein (MT) promoter, leading to widespread TGFα expression in the stomach and other tissues. - Control (FVB/N): Wild-type littermates used as controls. | - Method:   - TGFα overexpression driven by the MT promoter disrupts gastric epithelial differentiation.  - The model mimics Ménétrier’s disease, characterized by hypertrophic gastropathy and achlorhydria (loss of acid secretion).   - Principle:  1. TGFα binds to the EGF receptor, altering progenitor cell proliferation and differentiation. 2. Key effects:   - Expansion of surface mucous cells (neutral mucin-producing).  - Depletion of parietal cells (acid-secreting) and chief cells (pepsinogen-secreting).  - No significant change in endocrine cells (e.g., D and G cells). | - Standard diet: Ziegler Brothers mouse chow and water ad libitum. - No additional dietary manipulations (e.g., high salt or carcinogens) were used. | - Histopathological analysis: H&E staining, PAS staining, and immunohistochemical detection of BrdU incorporation for DNA synthesis. - In situ hybridization: Localization of transcripts for H^+^,K^+^-ATPase, spasmolytic polypeptide, somatostatin, and TGFα. - RNA blot hybridization: Analysis of RNA levels for pepsinogen C, H^+^,K^+^-ATPase, somatostatin, gastrin, and spasmolytic polypeptide. - Quantification of cellular changes: Measurement of gastric mucosa thickness and volume fractions of different cell types. - Gastrin radioimmunoassay: Measurement of serum gastrin levels. | - Advantages:   - Recapitulates Ménétrier’s disease (hypertrophic gastropathy + achlorhydria).  - Demonstrates TGFα’s role in disrupting differentiation (not just proliferation).  - Useful for studying EGF receptor signaling in gastric homeostasis.   - Disadvantages:   - No tumorigenesis or invasive lesions (limited to hyperplastic changes).  - Broad TGFα expression (MT promoter) may not reflect physiological TGFα regulation. | - Neonatal (0–3 weeks):   Minimal TGFα transgene expression; normal parietal/chief cell populations.   - Juvenile (3–6 weeks):   - TGFα expression peaks; increased proliferation in glandular isthmus.  - Parietal/chief cell depletion begins (↓ H+,K+-ATPase & pepsinogen C).   - Adult (>2 months):   - Severe mucosal thickening (surface mucous cell accumulation).  - Achlorhydria (pH neutral, no response to pentagastrin). | - Fundus: Most severely affected (TGFα expression highest here). - Antrum: Sparsely affected (minimal TGFα expression). | - No tumors or metastasis observed. - Pathology limited to hyperplasia and differentiation defects. | - No significant inflammation reported. - Immune cells: Not a focus, but apoptosis/necrosis slightly increased in the pit region. | - Studying the molecular mechanisms of TGFα in gastric mucosa differentiation and proliferation. - Investigating the role of TGFα in gastric acid production and achlorhydria. - Screening and testing potential therapeutic agents for conditions like Ménétrier’s disease. - Understanding the effects of TGFα overexpression on gastric cell renewal and differentiation. |
| Ref. (48) | - MT-TGFα (Line Tg(IMt-1, TGFα)Bri149): Transgenic mice overexpressing rat TGFα under the metallothionein (MT) promoter, induced by cadmium (CdSO₄). - Control: Non-transgenic C57BL/6xDBA littermates. | - Method:   - TGFα overexpression induced by cadmium injections (0.5 mg/kg) starting at 13 days of age.  - Examined gastric mucosa at 13, 21, and 28 days post-induction.   - Principle:  1. TGFα disrupts gastric lineage differentiation, leading to:   - Hyperplasia of surface mucous cells (pS2⁺).  - Depletion of parietal/chief cells (↓ H⁺/K⁺-ATPase, pepsinogen).   1. Mimics Ménétrier’s disease (hypertrophic gastropathy). | - Standard diet; no specific dietary modifications. - Cadmium sulfate (CdSO₄) administered via intraperitoneal injections to induce MT promoter-driven TGFα expression. | - Histopathology:   H&E, diastase-resistant PAS (D/PAS) for mucin detection.   - Immunohistochemistry (IHC):   TGFα, spasmolytic peptide (SP), PCNA (proliferation marker).   - In Situ Hybridization:   Localized mRNA for TGFα, pS2, SP, intestinal trefoil factor (ITF).   - Key Markers:   - pS2: Surface mucous cells.  - SP: Mucous neck cells.  - PCNA: Progenitor zone proliferation. | - Advantages:   - Recapitulates Ménétrier’s disease (mucous cell hyperplasia + achlorhydria).  - Demonstrates TGFα’s role in lineage-specific differentiation defects.   - Disadvantages:   - No tumorigenesis or invasive lesions.  - Broad TGFα expression (MT promoter) may not reflect physiological regulation. | - 13 days:   TGFα mRNA localized to gland base; minimal mucosal changes.   - 21 days (8 days post-induction):   - Expanded foveolar compartment (pS2⁺ surface cells).  - Progenitor zone shifts to gland base (PCNA⁺).   - 28 days (15 days post-induction):   Severe hyperplasia of pS2⁺ cells; near-complete loss of parietal/chief cells. | - Fundus: Primary site of TGFα-driven hyperplasia. - Antrum: Minimal involvement (no significant TGFα expression). | - No tumors or metastasis observed. - Pathology limited to hyperplasia and differentiation defects. | - No significant inflammation reported. - Focus on epithelial remodeling, not immune infiltration. | - Study TGFα’s role in gastric lineage differentiation. - Model for Ménétrier’s disease and mucous cell hyperplasia. - Test agents targeting TGFα/EGF-R signaling. - Investigate mucosal repair mechanisms. |
| Ref. (48) | - Strain: MT-TGF-α transgenic mice [Tg(Mt-1, TGF-α)Bri149]. - Genetic Background: Generated by crossing transgenic males with C57BL/oxDBA females | - Method: Overexpression of transforming growth factor-alpha (TGF-α) driven by the metallothionein promoter/enhancer (MT-TGF-α). - Principle: TGF-α overexpression alters gastric cell lineage differentiation, promoting foveolar hyperplasia (similar to Ménétrier’s disease) and reducing parietal cell numbers. This mimics a pre-cancerous state characterized by mucosal hyperplasia and altered differentiation. | - Mice were maintained with standard laboratory chow and water ad libitum. - Cadmium sulfate (0.5 mg/kg) was administered interperitoneally starting at 13 days of age to induce metallothionein promoter activity. | - Histology:   Diastase-resistant, periodic acid–Schiff (DR-PAS) staining for mucous cells.   - Immunohistochemistry for H/K-ATPase (parietal cells) and BrdU (proliferative cells). - Cell Proliferation: BrdU labeling to identify S-phase cells and progenitor zones. - Apoptosis Detection: Terminal deoxytransferase assay for DNA chain breakage. - Protein Levels: Radioimmunoassay for TGF-α and gastrin in serum and tissue. - Morphometry: Quantification of mucosal height, cell counts per gland, and distribution of cell types. | - Advantages:   - Recapitulates human Ménétrier’s disease and pre-cancerous hyperplasia.  -Clear lineage-specific alterations (e.g., mucous vs. parietal cells).  -Useful for studying TGF-α’s role in gastric differentiation and tumorigenesis.   - Disadvantages:   - Limited to pre-cancerous hyperplasia; no spontaneous tumor formation reported.  - Dependence on heavy metal (cadmium/zinc) for transgene induction.  - No evidence of metastasis or invasion. | - Early Hyperplasia (Day 0–3): Increased DR-PAS-positive mucous cells, reduced parietal cells. - Progressive Hyperplasia (Day 8–15): Further expansion of foveolar compartment, mucosal height increase. | - Primary Site: Fundic mucosa (corpus). - Secondary Site: Antral mucosa showed altered gastrin cell distribution but no hyperplasia. | No evidence of invasive or metastatic behavior reported in the study. | - Inflammation: Not explicitly described in the study. - Immune Cells: No mention of specific immune cell infiltration (e.g., macrophages). | - Study TGF-α’s role in gastric lineage differentiation. - Model Ménétrier’s disease and pre-neoplastic hyperplasia. - Investigate mechanisms of mucosal repair and regeneration. - Explore therapeutic interventions for hypertrophic gastropathies. |
| Ref. (49) | - Strain: MT-TGF-α transgenic mice [Tg(Mt-1, TGF-α)Bri149] and Pdx1^LacZ/+^ mice. - Genetic Background: Pdx1^LacZ/+^/MT-TGF-α bigenic mice were generated by crossing Pdx1^LacZ/+^ mice with MT-TGF-α mice. | - Method: Overexpression of TGF-α driven by the metallothionein promoter (MT-TGF-α), with transgene expression induced by zinc (ZnSO₄) in drinking water. - Principle: TGF-α overexpression leads to fundic mucosal "antralization," characterized by oxyntic atrophy (loss of parietal cells), foveolar hyperplasia, and ectopic expression of the antral transcription factor Pdx1 in the fundus. This mimics the pre-cancerous state seen in Ménétrier’s disease. | - Mice were maintained with standard laboratory chow and water ad libitum. - Zinc sulfate (25 mmol/L) was administered in drinking water to induce TGFα expression. | - Histopathological analysis: H&E staining, β-galactosidase histochemistry, immunohistochemistry for Pdx1, TFF2, and gastrin. - Quantitative PCR: Evaluation of Pdx1 and gastrin mRNA expression in mouse and human tissues and AGS cells. - Gene Expression in AGS Cells: Treatment of AGS cells with TGFα and analysis of Pdx1 and gastrin mRNA expression. - Clinical Specimens: Biopsy specimens from Ménétrier’s disease patients and normal controls for immunohistochemistry and quantitative PCR. | - Advantages:   - Recapitulates human Ménétrier’s disease and pre-neoplastic antralization.  - Demonstrates TGF-α’s role in lineage reprogramming (Pdx1 induction).  - Useful for studying EGFR signaling in gastric differentiation and disease.   - Disadvantages:   - No spontaneous tumor formation or metastasis reported.  - Requires zinc induction for transgene expression.  - Limited to pre-cancerous hyperplasia; no invasive or metastatic progression. | - Early (2 weeks): Initial Pdx1 expression in fundus, TFF2^+^ basal mucous cells, scattered gastrin cells. - Intermediate (8 weeks): Prominent Pdx1 expression, cystic glandular dilatations (gastritis cystica profunda), reduced TFF2^+^ cells. - Late (20 weeks): Extensive cystic changes, loss of parietal cells, minimal TFF2^+^ or gastrin^+^ cells. | - Primary Site: Fundic mucosa (corpus). - Secondary Site: Antral mucosa showed normal Pdx1 expression but no hyperplasia. | No evidence of invasive or metastatic behavior in MT-TGF-α mice or Ménétrier’s patients. | - Inflammation: Not a prominent feature in MT-TGF-α mice or non-inflammatory Ménétrier’s disease. - Immune Cells: No specific immune cell infiltration (e.g., macrophages) reported. | - Study TGF-α/EGFR signaling in gastric lineage differentiation. - Model Ménétrier’s disease and pre-neoplastic antralization. - Investigate therapeutic interventions (e.g., EGFR inhibitors). - Explore Pdx1’s role in gastric metaplasia and cancer. |
| Ref. (50) | - Strain: IL-8Tg mice (transgenic mice expressing human IL-8 via a bacterial artificial chromosome [BAC]). - Genetic Background: CBA/C57BL/6. | - Method: Transgenic expression of human IL-8 under its native regulatory elements, induced by inflammatory stimuli (e.g., *H. felis* infection or gastrin overexpression). - Principle: IL-8 mobilizes immature myeloid cells (IMCs, CD11b+Gr-1+), exacerbating inflammation and remodeling the gastric microenvironment to promote carcinogenesis. | - Mice were maintained with standard laboratory chow and water ad libitum. - Treatment:   - *H. felis* infection: Mice were inoculated at 8 weeks of age and monitored for 6–18 months.  - Gastrin overexpression: Crossed with INS-GAS mice (gastrin-overexpressing model).   - Control: Wild-type (WT) littermates under identical conditions. | - Histopathological analysis: H&E staining, immunostaining for CD11b, F4/80, α-SMA, and endomucin. - Quantitative PCR: Evaluation of IL-8 mRNA expression in gastric tissues. - ELISA: Measurement of IL-8 protein levels in serum. - Flow cytometry: Analysis of CD11b^+^Gr-1^+^ IMCs in peripheral blood, spleen, and bone marrow. | - Advantages:   - Recapitulates human IL-8 physiology (tight regulation, myeloid-specific expression).  - Demonstrates IL-8’s role in IMC mobilization and tumor microenvironment remodeling.  - Validates IL-8 as a therapeutic target in gastric cancer.   - Disadvantages:   - No spontaneous gastric tumors without additional triggers (e.g., *H. felis* or gastrin).  - Limited to pre-metastatic stages; invasive/metastatic potential not assessed. | - 6 months post-infection: Gastric dysplasia detected only in IL-8Tg mice. - 12 months post-infection: Pseudopyloric metaplasia, foveolar hyperplasia, and dysplasia significantly increased in IL-8Tg mice. - 18 months post-infection: Continued elevation of IL-8 levels and gastric dysplasia in IL-8Tg mice. | - Primary Site: Gastric corpus (fundus). - Secondary Site: Antral involvement not reported. | The study did not report on tumor invasiveness or metastasis in the stomach. | - Inflammation: Increased inflammatory cell infiltration in the gastric mucosa of IL-8Tg mice. - Inflammatory cells: Increased mobilization of CD11b^+^Gr-1^+^ IMCs, which include myeloid-derived suppressor cells (MDSCs). - Macrophages: Increased F4/80^+^ macrophages in the gastric mucosa of IL-8Tg mice. | - Studying the molecular mechanisms of IL-8 in gastric carcinogenesis. - Investigating the role of IL-8 in mobilizing IMCs and its impact on the tumor microenvironment. - Screening and testing potential therapeutic agents targeting IL-8 for gastric cancer. - Understanding the effects of IL-8 overexpression on gastric inflammation and cancer development. |
| Ref. (51) | - Primary Strain: H/K-ATPase-β/SDF-1 transgenic mice (SDF-Tg), generated by overexpressing murine SDF-1α in gastric parietal cells. - Additional Strains Used:   - CXCR4-EGFP mice (to identify CXCR4-expressing cells).  - αSMA-RFP reporter mice (to label myofibroblasts).  - H/K-ATPase-IL-1β mice (to study synergy with inflammation).  - TFF2 knockout mice (to examine CXCR4 antagonism).  - UBC-GFP mice (for bone marrow transplantation studies).   - Background: Initially mixed (C57BL/6 × CBA), later backcrossed to C57BL/6J. | - Method:   - SDF-1 Overexpression: Driven by the H/K-ATPase-β promoter to target parietal cells.  - *Helicobacter felis* Infection: Oral gavage (3 inoculations of 0.2 mL, 10¹⁰ CFU/mL, every other day).  - IL-1β Synergy: Crossed SDF-Tg mice with H/K-ATPase-IL-1β transgenic mice.   - Principle:   SDF-1/CXCR4 axis promotes dysplasia by:  - Expanding stromal myofibroblasts (αSMA^+^) and epithelial progenitors (CXCR4^+^).  - Recruiting Gremlin 1^+^ mesenchymal stem cells (MSCs) to the gastric niche.  - Synergizing with *H. felis* or IL-1β to amplify inflammation and myeloid cell recruitment. | - Standard laboratory diet and housing conditions. - *H. felis*-infected mice were monitored for up to 18 months post-infection (MPI). | - Histopathology: Scoring (0–4 scale) for inflammation, atrophy, hyperplasia, and dysplasia. - Immunohistochemistry (IHC):   Ki67 (proliferation), αSMA (myofibroblasts), F4/80 (macrophages), CD11b (myeloid cells), p-Akt/p-Erk (signaling pathways).   - Flow Cytometry: Immune cell profiling (e.g., CD45^+^, CD11b^+^ cells). - qRT-PCR/ELISA: SDF-1, IL-1β, IL-6, Gremlin 1, and other cytokines. - Migration Assays: Boyden chamber for MSC chemotaxis. - Bone Marrow Transplantation: UBC-GFP mice to track BM-derived cell recruitment. | - Advantages:   - Recapitulates human gastric dysplasia via stromal-epithelial crosstalk.  - Spontaneous dysplasia in SDF-Tg mice (28.6% incidence at 18 months).  - Synergy with *H. felis* or IL-1β mimics inflammation-driven carcinogenesis.   - Disadvantages:   - Low inflammation in SDF-Tg alone; requires additional triggers (e.g., *H. felis*) for robust phenotypes.  - Long latency (12–18 months for dysplasia). | - Early Stages (2–6 months):   - Epithelial hyperproliferation (Ki67^+^), mucous neck cell hyperplasia.  - Expansion of αSMA+ myofibroblasts and CXCR4^+^ progenitors.   - Late Stages (12–18 months):   - SDF-Tg Alone: Spontaneous dysplasia (28.6% at 18 months), cystic gland dilation.  - SDF-Tg + *H. felis*: Severe pseudo-pyloric metaplasia, oxyntic atrophy, dysplasia (15–18 MPI).  - SDF-Tg + IL-1β: Accelerated atrophy, metaplasia, and inflammation (1–1.5 months). | Corpus and Antrum:   - Dysplasia and tumors observed in both regions (e.g., cystic glands in corpus, antral tumors). - Progenitor zone (isthmus) in corpus and lower antral glands are primary sites. | The study focuses on preneoplastic dysplasia; invasive or metastatic potential was not evaluated. | - SDF-Tg Alone: Minimal inflammation early; late increase in F4/80^+^ macrophages and myeloperoxidase activity (12+ months). - Synergistic Models:   - SDF-Tg + *H. felis*: Significant F4/80^+^ macrophages and CD11b^+^ myeloid cells.  - SDF-Tg + IL-1β: Severe inflammation with IL-6 upregulation. | - Study SDF-1/CXCR4 in stromal-epithelial interactions and progenitor niche modulation. - Test chemopreventive agents targeting CXCR4 (e.g., AMD3100). - Investigate inflammation-cancer transition (e.g., IL-1β or *Helicobacter* synergy). - Validate SDF-1/CXCR4 as a therapeutic target in gastric cancer. - Model early-stage dysplasia for biomarker discovery. |

**Table 6:** Summary of Oncogene Mutation–Induced Animal Models for Gastric Precancerous Lesions

| Studies | Animal Model | Modeling Method and Principle | Feeding Conditions | Model Evaluation Methods | Advantages and Disadvantages | Detailed Time Course of Pathological Stages | Anatomical Sites of Lesions | Tumor Location and Invasion/Metastasis | Inflammation and Immune Cell Infiltration | Application Scenarios for Models |
| --- | --- | --- | --- | --- | --- | --- | --- | --- | --- | --- |
| Ref. (52) | - Primary Strain: K19-K-ras V12 transgenic mice, generated by fusing the cytokeratin 19 (K19) promoter (active in gastric mucous neck cells) to a mutant human K-ras gene (Gly→Val at codon 12). - Genetic Background: B6 × SJL hybrid (for microinjection), maintained as transgenic lines. - Founder Lines: Three independent lines (A, B, C) with transgene integration confirmed by PCR/Southern blot. | - Method:   - Transgene Design: K19 promoter drives mutant K-ras (V12) in gastric mucous neck cells (stem cell zone).  - Targeted Cells: Mucous neck cells (precursors to pit/zymogenic lineages).   - Principle:  1. Mutant K-ras disrupts gastric epithelial homeostasis, causing:   - Mucous neck cell hyperplasia (increased proliferation).  - Parietal cell loss (atrophy-like changes).  2. Mimics early gastric adenocarcinoma precursors (e.g., chronic atrophic gastritis). | The article does not provide specific details on feeding conditions. | - Histopathology: H&E staining for gastric hyperplasia/atrophy. - Immunohistochemistry (IHC):   - AAA lectin: Labels mucous neck cells.  - H^+^/K^+^-ATPase: Marks parietal cells (loss quantified).   - BrdU Labeling: Proliferation in mucous neck cells. - Molecular Analysis:   - Western blot: Ras activity, downstream effectors (e.g., cyclin D1).  - RT-PCR: N-cadherin (adhesion marker). | - Advantages:   - Recapitulates human gastric preneoplasia (hyperplasia → atrophy).  - Tissue-specific (K19 avoids off-target effects).  - Early preneoplastic stages (3–6 months).   - Disadvantages:   - No invasive cancer—requires additional mutations (e.g., p53 loss).  - Limited inflammation (immune cell infiltration not prominent in stomach). | - Mucous neck cell hyperplasia observed as early as 3–6 months and persisted up to 18 months. - Decrease in parietal cell mass observed concurrently with mucous neck cell hyperplasia. | - Mucous neck cell compartment (isthmus, stem cell zone). - Corpus/Antrum: Not specified. | - No invasive or metastatic lesions observed. - Model captures preneoplastic changes only. | - No significant inflammation reported. - Immune cells (e.g., macrophages) not highlighted in gastric lesions. | - Study K-ras signaling in gastric stem/progenitor cells. - Test chemopreventive agents targeting hyperplasia/atrophy. - Model early gastric carcinogenesis for biomarker discovery. - Combine with other mutations (e.g., p53) to study progression. |
| Ref. (53) | - Primary Strain: K19-K-ras-V12 (K19-kras) transgenic mice, generated using a 2.1-kb K19 promoter-driven mutant human K-ras (Val 12) gene. - Background: Backcrossed to C57BL/6 for at least three generations. - Control Strains: Wild-type (WT) C57BL/6 mice, some infected with *H. felis* for comparison. | - Method: Transgenic expression of oncogenic K-ras-V12 in gastric epithelial progenitor cells (marked by keratin 19, K19). - Principle:   - K-ras mutation mimics chronic inflammation (similar to *H. felis* infection), activating proinflammatory cytokines (e.g., IL-6, CXCL1) and recruiting bone marrow-derived cells (BMDCs).  - Mutant K-ras drives expansion of putative progenitor cells (Dcamkl1^+^) and disrupts normal glandular architecture, leading to metaplasia, dysplasia, and neoplasia. | - The article does not provide specific details on feeding conditions. - Infection: For controls, WT mice were infected with *H. felis* via oral gavage (ATCC 49179 strain). - Bone Marrow Transplantation (BMT): Some K19-kras mice received GFP-labeled bone marrow from β-actin-EGFP donors. | - Histopathology: H&E staining graded for inflammation, atrophy, metaplasia, hyperplasia, and dysplasia. - Immunohistochemistry (IHC): Staining for K19, Dcamkl1, Ki67, TFF2, αSMA, vimentin, CD45, and GFP. - Molecular Analysis:   - RT-PCR for cytokines (IL-6, IL-1β, CXCL1, CXCL5) and growth factors (HB-EGF, amphiregulin).  - In situ hybridization for K19 mRNA and Y-chromosome (Y-FISH).   - Flow Cytometry: To identify GFP^+^ BMDCs (CD45^+^ leukocytes or CD45− stromal cells). - Gross Examination: Stomach weight, mucosal thickness, and tumor formation. | - Advantages:   - Recapitulates human gastric carcinogenesis stages (inflammation → metaplasia → dysplasia → carcinoma).  - K-ras mutation alone induces inflammation, bypassing need for *Helicobacter* infection.  - Useful for studying stromal-tumor interactions (BMDC recruitment).   - Disadvantages:   - Slow progression (takes ~20 months for invasive carcinoma).  - Limited metastasis (only intramucosal invasion observed).  - Dcamkl1’s role as a true progenitor marker remains unproven. | - 3 Months:   Mucous metaplasia (Alcian blue+ cells), mild inflammation, pit cell expansion.   - 6–12 Months:   Oxyntic atrophy, pseudopyloric metaplasia, hyperplasia, lymphoid infiltration.   - 12–18 Months:   Low-grade dysplasia, stromal remodeling (αSMA^+^ myofibroblasts).   - 16–20 Months:   High-grade dysplasia, intramucosal carcinoma (rare submucosal invasion). | Lesions are observed in the oxyntic mucosa of the stomach, specifically in the neck/isthmus region where K19^+^ cells reside. | - Invasiveness: Limited to intramucosal carcinoma with focal submucosal penetration (no distant metastasis reported). - Metastasis: Not observed in this model. | - Chronic Inflammation: Present in both K19-kras and *H. felis*-infected mice. - Key Immune Cells: - - Macrophages (F4/80^+^), lymphocytes (CD45^+^), and neutrophils (recruited via CXCL1/CXCL5). - - Stromal cells: Bone marrow-derived αSMA^+^ myofibroblasts and vimentin+ fibroblasts. - Cytokines: IL-6, IL-1β, CXCL1, CXCL5 upregulated. | - Study inflammation-driven gastric carcinogenesis (independent of *Helicobacter*). - Investigate stromal-tumor crosstalk (BMDC recruitment, CAF role). - Test therapies targeting K-ras pathways or inflammatory cytokines (e.g., CXCL1 inhibitors). - Not suitable for studying antral tumors or metastatic gastric cancer. - Slow progression may delay therapeutic testing. |
| Ref. (54) | - Model: CK19^CreERT^; LSL-Kras^G12D^ mice (C57BL/6J background). - Targeting: Tamoxifen-inducible Kras^G12D^ expression in K19^+^ gastric epithelial cells (primarily in the neck/isthmus progenitor zone). | - Method: Adult mice (6–8 weeks old) received tamoxifen to activate Kras^G12D^ in gastric epithelium. - Principle:   - Kras^G12D^ drives foveolar hyperplasia and pseudopyloric metaplasia, mimicking early gastric preneoplasia.  - Unlike *H. pylori* models, this is a genetic inflammation-independent pathway to metaplasia. | The article does not provide specific details on feeding conditions. | - Gross: Stomach weight/morphology. - Histopathology:   - H&E: Foveolar hyperplasia, oxyntic atrophy.  - PAS/Alcian blue: Mucin patterns (diffuse, non-intestinal).   - IHC:   - TFF2: Mislocalized mucous neck cells.  - Phosphohistone H3/Ki67: Proliferation zone shifted to gland base.   - PCR: Confirmed Kras^G12D^ recombination in stomach. | - Advantages:   - Pure genetic model (no infection/inflammation confounders).  - Targets fundic progenitors (K19^+^ neck/isthmus cells).  - Recapitulates early metaplasia (TFF2 mislocalization).   - Disadvantages:   - No progression to cancer (pre-neoplastic only).  - Limited to fundus (antrum unaffected).  - No inflammatory component (unlike *H. pylori* models). | - 4–6 Months Post-Tamoxifen:   - Foveolar Hyperplasia: Extended PAS^+^ mucous zone.  - Oxyntic Atrophy: Parietal cell loss.  - Metaplasia: Basal TFF2^+^ mucous neck cells (abnormal localization).   - Beyond 6 Months: No dysplasia/carcinoma reported. | - Exclusively Fundus:   - Lesser curvature: 10–50% glands affected.  - Severity decreases toward greater curvature.   - Antrum: No pathology observed. | Non-Neoplastic: Lesions remained hyperplastic/metaplastic (no dysplasia or invasion). | No Inflammation Reported:   - Immune cells: Not mentioned (no macrophages/lymphocytes described). - Cytokines: Not analyzed (unlike cytokine-driven models). | - Study Kras-driven metaplasia without inflammatory confounders. - Investigate progenitor cell plasticity (TFF2^+^ cell expansion). - Not suitable for cancer progression or immune-microenvironment studies. - Requires combinatorial models (e.g., Kras^G12D/+^ Tp53 loss) for malignancy. |
| Ref. (55) | - Strain: LSL-K-ras^G12D/+^ mice (C57BL/6 and 129/Sv mixed background) crossed with Ubc9 promoter-driven Cre-ERT2 mice (C57BL/6 background). - Genotype: LSL-K-ras^G12D/+^; Ubc9 Cre-ERT2 (inducible systemic K-ras activation). | - Method:   Tamoxifen (TAM)-induced Cre recombinase excises the LoxP-STOP-LoxP (LSL) cassette upstream of the K-ras^G12D^ allele, activating oncogenic K-ras ubiquitously.   - Principle:   - K-ras^G12D^  activates MAPK/ERK and p38 pathways, driving hyperproliferation, metaplasia, and inflammation.  - Targets gastric stem/progenitor cells (marked by Dcamk11, CD44) and induces intestinal metaplasia, a precancerous lesion. | Mice were fed tamoxifen (TAM) at 200 mg/kg body weight/day by gavage for two consecutive days, followed by one day off, and then for two more consecutive days. | - Histopathology: H&E staining for hyperplasia, metaplasia. - Immunostaining:   - BrdU (proliferation), H^+^/K^+^ ATPase (parietal cells), CDX2 (intestinal metaplasia), CD44/Dcamk11 (stem/progenitor markers), CD45/CD68 (immune cells), COX-2 (inflammation).   - Western Blot: Phospho-MEK/ERK/p38, CD44, Lgr5, CDX2. - Alcian Blue: Acidic mucins (intestinal metaplasia marker). - Blood Glucose: Measured via tail vein. | - Advantages:   - Rapid induction (≤15 days) of precancerous lesions resembling human gastric carcinogenesis.  - Endogenous K-ras expression (physiologically relevant).  - Useful for studying early molecular events (e.g., MAPK activation, stem cell dynamics).   - Disadvantages:   - High mortality (100% by 18 days post-TAM) limits long-term studies.  - Systemic K-ras activation affects multiple organs, though stomach is primary target.  - No frank carcinoma observed within the study period. | - Hyperplasia:   - Squamous forestomach: Evident by 15 days.  - Glandular stomach: Evident by 15 days (shifted BrdU^+^ cells to crypts).   - Metaplasia (intestinal metaplasia):   Glandular stomach: Alcian blue^+^ cells and CDX2 upregulation by 13–16 days.   - Parietal/Enteroendocrine Cell Loss:   Near-complete depletion by 15 days (H^+^/K^+^ ATPase-, chromogranin A-). | - Forestomach: Squamous hyperplasia. - Glandular Stomach:   - Adjacent to forestomach: Prominent metaplasia.  - Body/Corpus: Reduced parietal cells, metaplasia.  - Pylorus: Not explicitly mentioned, but CD44^+^ cells expanded in crypts. | - No evidence of invasion or metastasis within 18 days. - Stomach mass was localized hyperplasia/metaplasia. | - Immune Cells: CD45^+^ hematopoietic cells, CD68^+^ macrophages infiltrated submucosa and epithelium. - Inflammatory Factors: COX-2 upregulated in epithelial and immune cells. | - Study early molecular events in gastric carcinogenesis (e.g., MAPK signaling, stem cell roles). - Investigate links between chronic inflammation (COX-2, immune infiltration) and metaplasia. - Test chemopreventive agents targeting precancerous lesions. - Not suitable for late-stage cancer studies (no carcinoma formed). - Acute model; chronic models may better mimic human progression. |
| Ref. (56) | - Primary Strain:  1. Mist1-CreERT2 (knock-in mice with tamoxifen-inducible Cre recombinase under the Mist1 promoter). 2. Crossed with:   - R26-mTmG, R26-TdTomato, R26-Confetti (lineage tracing reporters).  - LSL-Kras^G12D^, Apc^flox/flox^, Cdh1^flox/flox^ (oncogenic/tumor suppressor models).  - Cxcr4-EGFP, Cxcl12-dsRED (niche marker reporters).   - Background: Mixed (C57BL/6 and 129/Sv). | - Method:  1. Lineage Tracing: Tamoxifen (TAM) induces Cre-mediated recombination in Mist1^+^ stem cells, activating reporters (e.g., GFP) or oncogenes (e.g., Kras^G12D^). 2. Cancer Induction:   - Intestinal-type gastric cancer (IGC): Combined Kras^G12D^ activation and Apc deletion in Mist1^+^ cells.  - Diffuse-type gastric cancer (DGC): Cdh1 (E-cadherin) deletion in Mist1^+^ cells + *H. felis* infection or IL-1β overexpression to induce inflammation.   - Principle:   - Mist1^+^ quiescent stem cells in the corpus isthmus are the origin of gastric lineages and cancers.  - Niche Dependency: Cxcl12^+^ endothelial cells and Cxcr4^+^ innate lymphoid cells (ILCs) form a perivascular niche supporting stem cells via Wnt5a/RhoA signaling. | - Tamoxifen Administration:   1–5 mg in corn oil (oral gavage or injection), dose-dependent on experiment.   - Additional Treatments:  1. Inflammation Models: *H. felis* infection or IL-1β transgenic overexpression. 2. Drug Interventions:   - AMD3100: Cxcr4 inhibitor.  - Anti-CD90.2 antibody: Depletes ILCs.  - Dexamethasone: Anti-inflammatory.   - Cell Ablation:   - Diphtheria toxin (DT): Targets Lgr5^+^ cells.  - 5-Fluorouracil (5-FU): Kills proliferating isthmus cells. | - Lineage Tracing:   Fluorescent reporters (GFP, TdTomato) to track Mist1^+^ progeny.   - Histopathology:   - H&E staining for hyperplasia, metaplasia, and cancer.  - Alcian blue for intestinal metaplasia.   - Immunostaining:   - Stem/Progenitor Markers: Mist1, Lgr5, CD44, Dcamk11.  - Differentiation Markers: H^+^/K^+^ ATPase (parietal cells), chromogranin A (enteroendocrine cells).  - Niche Components: Cxcl12 (endothelium), Cxcr4 (ILCs), Wnt5a.   - *In Vitro* Assays:   - Organoid Culture: Assess stem cell potential under Wnt/Notch modulation.  - Soft-Agar Assays: Anchorage-independent growth of cancer cells.   - Molecular Analysis:   qRT-PCR, Western blot (e.g., phospho-ERK, RhoA activation). | - Advantages:   - Identifies Mist1^+^ isthmus cells as the origin of both normal homeostasis and cancer.  - Recapitulates human DGC (E-cadherin loss + inflammation) and IGC (Kras/Apc mutations).  - Demonstrates niche dependency (Cxcl12/Cxcr4/Wnt5a), offering therapeutic targets.   - Disadvantages:   - DGC Model: Requires prolonged inflammation (e.g., 18 months post *H. felis* infection).  - IGC Model: Needs dual genetic hits (Kras + Apc).  - Lineage Tracing Artifacts: Tamoxifen may perturb stem cell activity. | - Normal Homeostasis:   Mist1^+^ cells divide every ~5 days, generating all gastric lineages over 540 days.   - Intestinal-Type Cancer (IGC):   - Hyperplasia/Metaplasia: 1–2 months post Kras^G12D^ activation.  - Dysplasia/Carcinoma: 4 months with additional Apc loss.   - Diffuse-Type Cancer (DGC):   - Early Atypical Foci: 10 days post Cdh1 deletion.  - Signet-Ring Cell Expansion: 6–18 months with chronic inflammation (*H. felis* or IL-1β). | - Corpus Isthmus: Primary site of Mist1^+^ stem cells and early lesions. - Glandular Stomach:   - IGC: Metaplasia/dysplasia spreads from isthmus to entire glands.  - DGC: Signet-ring cells originate in isthmus, infiltrate diffusely. | - IGC: Localized intramucosal tumors (no metastasis reported). - DGC: Invasive signet-ring cell carcinoma (with Trp53 mutation, invasion occurs by 9 months). | - Chronic inflammation induced by *H. felis* infection is crucial for DGC development - Cxcl12^+^ endothelial cells and Cxcr4^+^ ILCs form the perivascular niche supporting gastric stem cells. - Wnt5a produced by ILCs promotes DGC development. | - Studying the cellular origin and molecular mechanisms of gastric cancer development. - Investigating the role of the Cxcl12/Cxcr4 perivascular niche in gastric stem cell maintenance and cancer progression. - Testing therapeutic interventions targeting the Cxcl12/Cxcr4 axis and Wnt5a signaling for DGC prevention and treatment. |
| Ref. (57) | - Triple Conditional (Tcon) Mice:   - Genotype: Atp4b-Cre/Rosa26LSL-YFP; Cdh1^fl/fl^; Trp53^fl/fl^; LSL-Kras^G12D/+^  - Background: Mixed (C57BL/6 and others).  Double Conditional E-cadherin Heterozygous (Dcon-Ecad het) Mice: Genotype: Atp4b-Cre/Rosa26LSL-YFP; Cdh1^fl/+^; Trp53^fl/fl^; LSL-Kras^G12D/+^ | - Method:   Genetic Engineering: Crossbreeding of mice with conditional alleles:  - Oncogenic Kras: LSL-KrasG12D (activated by Cre recombinase).  - p53 Loss: Trp53^fl/fl^ (floxed alleles deleted by Cre).  - E-cadherin Loss: Cdh1^fl/fl^ (complete loss) or Cdh1^fl/+^ (heterozygous).  - Lineage-Specific Cre: Atp4b-Cre (targets gastric parietal cell lineage).  - Reporter: Rosa26LSL-YFP (marks recombined cells).   - Principle:   - Oncogenic Kras + p53 Loss: Drives rapid tumorigenesis.  - E-cadherin Loss: Accelerates tumor progression and metastasis by disrupting cell adhesion and enhancing RAS/MAPK signaling. | - Standard Diet: Purina 5010 mouse chow. - MEK Inhibitor Treatment: PD0325901 mixed into chow (7 mg/kg) for intervention studies. | - Survival Analysis: Median survival tracked (Tcon: 76 days; Dcon-Ecad het: 123 days). - Histopathology: H&E staining for tumor classification (intestinal/diffuse/mixed-type). - Immunofluorescence: YFP (tumor cells), E-cadherin, β-catenin localization. - Metastasis Detection: YFP^+^ nodules in lymph nodes, lung, liver. - Molecular Analysis:   - RAS Activity: RAF-RBD pull-down assay.  - Western Blot: Phospho-MEK/ERK, β-catenin targets.  - qRT-PCR: WNT/β-catenin pathway genes (e.g., LEF1, c-Myc).  - Microarray: Gene expression profiling (KRAS/WNT signatures). | - Advantages:   - Rapid Tumorigenesis: Median survival of 76 days (Tcon).  - Metastasis: Recapitulates human disease (lymph nodes, lung, liver).  - Mixed-Type Tumors: Mimics human intestinal and diffuse subtypes.  - Genetic Control: Tunable E-cadherin expression (heterozygous vs. knockout).   - Disadvantages:   - Aggressive Progression: Limits time for intervention studies.  - Mixed Background: Potential variability due to genetic heterogeneity. | - 3 Weeks:   Loss of parietal cells; high-grade dysplasia/intramucosal carcinoma (100% mice).   - 6 Weeks:   Invasive carcinomas (40% mice).   - 9 Weeks:   Invasive carcinomas (100% mice); metastases (lymph nodes, lung, liver). | Lesions observed throughout the stomach, with metastases in lymph nodes, lung, and liver. | - Invasion: Yes (intramucosal to invasive carcinomas by 6–9 weeks). - Metastasis:   - Perigastric Lymph Nodes: 100% of Tcon mice.  - Mediastinal Lymph Nodes: 50%.  - Lung: 100%.  - Liver: 20% (micrometastases). | The article does not detail inflammatory infiltrates or specific immune cells (e.g., macrophages) in tumor microenvironments. | - Investigating the molecular mechanisms of gastric adenocarcinoma progression. - Testing new therapies targeting primary gastric cancer and metastatic lesions. - Studying the role of E-cadherin and the RTK/Ras signaling axis in gastric cancer. |
| Ref. (58) | TFF1-Cre BAC Transgenic Mice:   - Genotype: TFF1-Cre; LSL-Kras^G12D^ (Kras activation), TFF1-Cre; Pten^flox/flox^ (Pten deletion), or TFF1-Cre; Cdh1^flox/flox^ (E-cadherin deletion). - Background: Not specified (likely C57BL/6). - Reporter: Rosa26-LacZ or Rosa26-EYFP for lineage tracing. | - Targeted Cells: Gastric pit lineage (TFF1-expressing cells) in the corpus and antrum, with minor recombination in chief/parietal cells. - Genetic Drivers:   - Kras Activation: Induces foveolar hyperplasia, oxyntic atrophy, and pseudopyloric metaplasia (SPEM).  - Pten Deletion: Mimics Kras activation via PI3K-AKT pathway dysregulation.  - Cdh1 Deletion: Leads to glandular epithelium loss and squamous metaplasia.   - Principle:   - Kras/Pten: Drive metaplasia via MAPK/ERK and PI3K-AKT signaling.  - Cdh1: Loss disrupts epithelial integrity, promoting squamous replacement. | - Standard Diet: Specific pathogen-free (SPF) conditions. - Interventions:   Clodronate liposomes (macrophage depletion) tested in Kras model. | - Histopathology: H&E, Alcian blue (mucins), PAS, and immunofluorescence (TFF1, TFF2, GSII, CD44v6, etc.). - Lineage Tracing: X-gal (LacZ) and YFP reporter assays. - Molecular Analysis:   - qRT-PCR for gastric markers (Muc5ac, Atp4b, Pgc) and cytokines (Areg, Cxcl2).  - Western blot for phospho-ERK, phospho-AKT.   - Organoid Culture: Derived from corpus glands to study epithelial proliferation/differentiation. | - Advantages:   - Tff1-Cre line allows for specific gene manipulation in the gastric pit lineage.  - Suitable for long-term analyses of gene modification in the stomach.  - Provides insights into the role of pit cells in metaplasia and cancer development.   - Disadvantages:   - Recombination activity is not restricted to mature pit cells and occurs in other cell lineages.  - Limited to studying the corpus and antrum regions of the stomach. | - Kras Activation (TFF1-Cre; LSL-Kras^G12D^):   - 3 Weeks: Ectopic mucin expression, early oxyntic atrophy.  3 Months: - Full SPEM, pseudopyloric metaplasia, oxyntic atrophy.  - 12 Months: No progression to cancer.   - Pten Deletion (TFF1-Cre; Pten^flox/flox^):   Similar timeline to Kras model (SPEM by 3 months).   - Cdh1 Deletion (TFF1-Cre; Cdh1^flox/flox^):   - 1 Week: Epithelial shedding, signet ring-like cells.  - 4 Weeks: Dysplastic glandular epithelium, inflammation.  - 12 Weeks: Complete squamous replacement. | - Kras/Pten Models: Corpus-predominant (oxyntic atrophy, SPEM). - Cdh1 Model: Whole glandular stomach (squamous metaplasia spreads from squamocolumnar junction). | - No Invasion or Metastasis: Observed in any model (even at 12 months for Kras/Pten). - Cdh1 Model: Lacks glandular epithelium by 12 weeks; no traditional tumor formation. | - Kras Model:   - Macrophages: Increased F4/80^+^ cells.  - Cytokines: Upregulated Cxcl2 and Areg (amphiregulin).  - Neutrophils/Myofibroblasts: Not significantly altered.   - Cdh1 Model:   Severe Inflammation: F4/80^+^ macrophages, MPO^+^ neutrophils, α-SMA^+^ myofibroblasts.   - Macrophage depletion did not attenuate metaplasia (cell-autonomous effects dominant). | - Studying the molecular mechanisms of gastric metaplasia and cancer development. - Investigating the role of Kras activation, Pten deletion, and Cdh1 deletion in gastric pathology. - Testing therapeutic interventions targeting these genetic alterations. |
| Ref. (59) | TFF1-CreERT2 BAC Transgenic Mice:   - Genotype:   - TFF1-CreERT2; KrasLSL-^G12D/+^ (Kras activation).  - TFF1-CreERT2; BrafLSL-V600E/+ (Braf activation).  - TFF1-CreERT2; gp130^F/F^ (STAT3 hyperactivation).  Compound mutants: TFF1-CreERT2; KrasLSL-^G12D/+^; gp130^F/F^.   - Background: Mixed C57BL/6 × 129/Sv. - Reporter: Rosa26-LacZ or Rosa26-YFP for lineage tracing. | - Targeted Cells: Gastric pit lineage (TFF1-expressing cells) in the corpus and antrum. - Induction: Tamoxifen administration (1 mg/20 g body weight for 5 days) activates CreERT2. - Genetic Drivers:   - Oncogenic Kras/Braf: Induces metaplasia, inflammation, and adenomas via MAPK/ERK.  - gp130^F/F^ Mutation: Hyperactivates STAT3, mimicking inflammation-driven tumorigenesis.   - Principle:   - Kras/Braf + STAT3: Cooperate to promote tumor progression and invasion.  - Inflammation: Required to overcome oncogene-induced senescence. | - Standard Diet: Specific pathogen-free (SPF) conditions. - Tamoxifen Protocol: Dissolved in ethanol/sunflower oil, injected intraperitoneally. | - Lineage Tracing: β-Gal (LacZ) and YFP reporter assays. - Histopathology: H&E, Alcian blue (mucins), PAS, and immunohistochemistry (TFF2, pY-STAT3, pERK). - Molecular Analysis:   - qRT-PCR for metaplasia markers (MUC2, CDX2).  - Western blot for pERK, pSTAT3.   - Tumor Burden: Quantified by tumor number/weight and submucosal invasion. | - Advantages:   - Stomach-Specific: TFF1-CreERT2 targets long-lived stem/progenitor cells.  - Tumor Progression: Recapitulates metaplasia → adenoma → submucosal invasion.  - STAT3 Dependency: Validates inflammation’s role in Kras-driven tumorigenesis.   - Disadvantages:   - Tamoxifen Toxicity: High doses cause transient parietal cell atrophy.  - Latency: Tumors develop slowly (9 months for Kras; 8 months for Braf). | - Kras/Braf Activation:   - 3 Months: Metaplasia (SPEM, pseudointestinal) and chronic gastritis.  - 9 Months (Kras) / 8 Months (Braf): Antral adenomas (30–67% penetrance).   - gp130^F/F^;Kras^G12D^ Compound Model:   3 Months: Increased tumor burden, submucosal invasion, and severe intestinal metaplasia. | - Primary Site: Antrum (adenomas with glandular/goblet-like structures). - Corpus: Mild inflammation, dilated glands (no tumors). | The study does not report invasive or metastatic gastric cancer in any of the models. | Presence of mononuclear cell infiltrates in the lamina propria of the antrum. | - Studying the molecular mechanisms of gastric tumorigenesis. - Investigating the role of Kras and Stat3 in gastric cancer development. - Testing therapeutic interventions targeting Kras and Stat3 pathways. |
| Ref. (60) | - Primary Model:   Iqgap3-2A-CreERT2; Kras^G12D/+^ mice (genetically engineered to express oncogenic Kras^G12D^ in Iqgap3^+^ stem cells upon tamoxifen induction).   - Other Strains Used:   - eR1-CreERT2; Rosa-tdTomato (Runx1 enhancer-marked stem cells).  - Iqgap3-2A-tdTomato (reporter for Iqgap3 expression).  - Wild-type C57BL/6Jlnv mice (controls). | - Method:   - Tamoxifen-Induced Injury: High-dose tamoxifen (HDT; 5 mg/20 g body weight) causes parietal cell death, leading to atrophy, metaplasia, and proliferation of Iqgap3^+^ stem cells.  - Oncogenic Kras Activation: Tamoxifen induces Kras^G12D^ expression in Iqgap3^+^ cells, driving pseudopyloric metaplasia (a preneoplastic lesion).   - Principle:   Iqgap3-Ras-Erk Axis: Iqgap3 promotes Ras/ERK signaling, essential for stem cell proliferation. Injury triggers Iqgap3 upregulation, mimicking early carcinogenesis. | - Not explicitly mentioned in the article. - Tamoxifen administered via intraperitoneal injection (single dose for lineage tracing; HDT for injury). | - Histopathology: H&E staining for gland morphology, metaplasia, and tumorigenesis. - Immunofluorescence (IF): Staining for markers (Ki67, Iqgap3, CD44v9, Muc5ac, H,K-ATPase). - Lineage Tracing: tdTomato labeling to track Iqgap3^+^ cell progeny. - RNA-Seq & qPCR: Transcriptomic analysis of stem/progenitor cells and cancer signatures. - Organoid Culture: Assess stem cell potential and differentiation. - Human Tissue Correlation: IQGAP3 expression in gastric cancer patient samples. | - Advantages:   - Targets specific stem cells (Iqgap3^+^ isthmus/chief cells) linked to human gastric cancer.  - Recapitulates metaplasia-to-cancer progression via Ras activation.  - Tamoxifen injury provides a reproducible inflammation model.   - Disadvantages:   - Slow progression (3 months for metaplasia; no full carcinoma reported).  - Limited metastasis data (not observed in the study).  - Tamoxifen may have off-target effects (e.g., systemic toxicity). | - Homeostasis: Normal gland structure (untreated mice). - Acute Injury (24–48 hrs post-HDT):   - Parietal cell loss, Ki67^+^ proliferation at isthmus/base.  - Iqgap3^+^ cell expansion and chief cell dedifferentiation.   - Repair (14 days post-HDT):   - Gland regeneration, parietal cell recovery.   - Oncogenic Kras Activation (3 months post-tamoxifen):   - Pseudopyloric metaplasia (Muc5ac^+^ surface cell hyperplasia, parietal cell loss).  - No invasive carcinoma or metastasis reported. | Corpus (Body): Primary site of Iqgap3+ stem cell activity, metaplasia, and Kras-driven lesions. | - No evidence of invasion or metastasis in the model. - Lesions were localized to the corpus with metaplastic changes. | CD45^+^ leukocytes (likely macrophages) infiltrate proliferative regions at the gland base | - Studying Gastric Stem Cell Dynamics: Understanding the behavior of isthmus stem cells during homeostasis and tissue repair. - Cancer Initiation Mechanisms: Investigating the early events leading to gastric cancer, including the role of Iqgap3 and Ras signaling. - Drug Screening: Testing potential therapeutic agents targeting Iqgap3 or Ras pathways in gastric tissue regeneration and cancer prevention. - Tissue Engineering: Developing strategies for gastric tissue repair and regeneration using stem cell-based approaches. |
| Ref. (61) | - Primary Strain: Mist1-CreERT2Tg/+:LSL-K-Ras(G12D)Tg/+ (Mist1-Kras mice)   - Lineage Tracing Strain: Mist1-CreERT2Tg/+:LSL-K-Ras(G12D)Tg/+:R26RmTmG/+ (Mist1-Kras-mTmG mice)  - Control Strain: Mist1-CreERT2Tg/+:R26RmTmG/+ (Mist1-mTmG mice)   - Background: C57BL/6 - Genetic Modifications:   - Mist1-CreERT2: Targets gastric chief cells for tamoxifen-inducible Cre recombinase activity.  - LSL-K-Ras(G12D): LoxP-STOP-LoxP (LSL) cassette controls expression of oncogenic Kras(G12D).  - R26RmTmG: Reports Cre activity via membrane-bound GFP (post-excision) or tdTomato (pre-excision). | - Method:   - Tamoxifen (5 mg/day, 3 consecutive days, subcutaneous) induces Cre-mediated excision of the STOP cassette in chief cells, activating Kras(G12D).  - MEK Inhibition: Some mice received the MEK inhibitor selumetinib (2 mg/kg/day, intraperitoneal, 14 days) to assess regression of metaplasia.   - Principle:   - Activated Kras(G12D) drives transdifferentiation of chief cells into spasmolytic polypeptide-expressing metaplasia (SPEM), progressing to intestinal metaplasia.  - Key Pathway: Ras/MAPK signaling promotes metaplastic transitions and proliferation. | - Diet: Standard chow (not specified in the study). - Tamoxifen Administration: Dissolved in corn oil, injected subcutaneously at 8 weeks of age. - MEK Inhibitor: Selumetinib dissolved in DMSO, diluted to 1 mg/mL for intraperitoneal injection. | - Histopathology: H&E staining for gland morphology, Alcian blue (AB)/periodic acid-Schiff (PAS) for mucin detection. - Immunostaining:   - SPEM Markers: Clusterin, GSII-lectin, CD44v.  - Intestinal metaplasia Markers: TFF3, Muc2, Cdx1.  - Proliferation: Ki-67.  - Lineage Tracing: GFP (R26RmTmG reporter).   - Electron Microscopy: Confirmed goblet cell morphology in intestinal metaplasia. - qPCR: Quantified intestinal metaplasia marker transcripts (TFF3, Muc2, Cdx1, Cdx2). - Macrophage Analysis: F4/80 (pan-macrophage), CD163 (M2 subtype), Ly6B.2 (neutrophils). | - Advantages:   - Recapitulates human gastric carcinogenesis (SPEM → intestinal metaplasia → dysplasia).  - Lineage tracing confirms chief cell origin of metaplasia.  - MEK inhibition reverses metaplasia, demonstrating therapeutic potential.   - Disadvantages:   - Limited to 4-month observation due to salivary gland hyperplasia (precluding long-term cancer studies).  - Mosaic GFP expression in Mist1-Kras-mTmG mice may underrepresent metaplastic lineages. | - 1 Week: Normal glands; phospho-ERK1/2 activation in chief cells (15.6%). - 1 Month:   - SPEM: 94.76% of glands show parietal cell loss, clusterin^+^/GSII^+^/CD44v^+^ cells.  - TFF3^+^ luminal cells (88% of glands).   - 2 Months:   SPEM with Intestinalizing Features: Gland fission (9.6%), Muc2+ goblet cells (13%).   - 3 Months:   intestinal metaplasia: Goblet cells (Muc2^+^/TFF3^+^) in 20% of glands; nuclear Cdx1 expression.   - 4 Months:   Invasive Metaplasia: Observed in 13% of mice (2/15). | Gastric corpus (fundic mucosa). | - Invasive Lesions: Present at 4 months (13% of mice). - Metastasis: Not reported (study terminated at 4 months). | - Macrophages:   - M2 Subtype (CD163^+^): Increased at 2–4 months, localized at gland bases.  - F4/80^+^: Co-stained with CD163, indicating M2 polarization.   - Neutrophils (Ly6B.2^+^): No significant change vs. controls. - Key Finding: M2 macrophages correlate with SPEM-to-intestinal metaplasia progression. | - Study metaplastic progression (SPEM → intestinal metaplasia) and early gastric carcinogenesis. - Test MEK inhibitors for metaplasia regression. - Investigate chief cell plasticity and cryptic progenitor roles. - Preclinical validation of Ras/MAPK pathway inhibitors. - Exploration of macrophage-targeted therapies in metaplasia. |
| Ref. (62) | Primary Strain: Mist1-CreERT2Tg/+:LSL-K-Ras(G12D)Tg/+ (Mist1-Kras mice)   - Background: C57BL/6 - Genetic Modifications:   - Mist1-CreERT2: Targets gastric chief cells for tamoxifen-inducible Cre recombinase activity.  - LSL-K-Ras(G12D): LoxP-STOP-LoxP (LSL) cassette controls expression of oncogenic Kras(G12D). | - Method:  1. Concomitant *H. pylori* (*Hp*) Infection and KRAS Activation:   - Mice were infected with *H. pylor*i strain PMSS1 (CagA⁺, active type IV secretion system) via oral gavage.  - Tamoxifen (5 mg/day for 3 days, subcutaneous) induced KRAS(G12D) expression in chief cells.   1. Antibiotic Eradication: Some mice received triple therapy (tetracycline, metronidazole, bismuth) to assess reversibility of metaplasia/dysplasia.  - Principle:   - KRAS Activation: Drives transdifferentiation of chief cells into SPEM and progression to intestinal metaplasia/dysplasia.  - *Hp* Coinfection: Exacerbates inflammation, alters immune cell profiles (e.g., T-cell exhaustion, M2 macrophage reduction), and accelerates dysplasia. | - Diet: Standard chow (LabDiet). - *Hp* Infection: 5 × 10⁷ CFU *H. pylori* in Brucella broth via oral gavage. - Antibiotics: Triple therapy (metronidazole, tetracycline, bismuth) administered orally for 2 weeks. | - Histopathology: H&E staining for inflammation, gland architecture, and dysplasia. - Immunostaining:   - SPEM Markers: GSII-lectin, CD44v.  - Intestinal metaplasia Markers: TFF3, MUC2.  - Dysplasia Marker: TROP2.  - Proliferation: Ki-67.   - Gene Expression: NanoString Mouse Immunology Panel (561 immune-related genes). - Immune Cell Profiling: Multiplex IHC for T cells (CD3, CD4, CD8α, FOXP3, PD-1) and macrophages (F4/80, MHC-II, CD163). - Bacterial Load: Quantitative culture of *H. pylori* from stomach homogenates. | - Advantages:   - Recapitulates human gastric carcinogenesis (SPEM → intestinal metaplasia → dysplasia) with *H. pylori* coinfection.  - Demonstrates *H. pylori*’s role beyond initiation (sustained infection worsens pathology).  - Antibiotic reversal validates therapeutic potential of *H. pylori* eradication.   - Disadvantages:   - Limited to 12-week observation (salivary gland tumors in Mist1-Kras mice preclude longer studies).  - Does not fully model invasive adenocarcinoma due to time constraints. | - 2 Weeks:   Mild inflammation, no significant differences between Hp^+^/KRAS^+^ and Hp^–^/KRAS^+^ mice.   - 6 Weeks:   - SPEM: GSII^+^/CD44v^+^ cells at gland bases.  - Early intestinal metaplasia: TFF3^+^ luminal cells, rare MUC2^+^ goblet cells.  - Dysplasia: TROP2^+^ glands (0–3.6%).   - 12 Weeks:   - Advanced intestinal metaplasia: Increased MUC2^+^ glands (20%).  - Severe Dysplasia: TROP2^+^ glands (1.5–9.1%, higher in *Hp*^+^/KRAS^+^).  - Hyperproliferation: Ki-67^+^ cells throughout glands. | Gastric corpus (fundic mucosa). | - Invasive Lesions: Not observed within 12 weeks. - Metastasis: Not reported (study terminated at 12 weeks). | - T Cells:   CD4^+^ Dominance: Increased in *Hp*^+^/KRAS^+^ mice, with FOXP3^+^ regulatory T cells and PD-1^+^ exhausted T cells.   - Macrophages:   - M1 (F4/80^+^/MHC-II^+^): Increased in *Hp*^+^/KRAS^+^ mice.  - M2 (F4/80^+^/CD163^+^): Reduced in *Hp*^+^/KRAS^+^ mice (vs. *Hp*^–^/KRAS^+^).   - Key Finding: *Hp* alters macrophage polarization and exacerbates T-cell exhaustion. | - Study *H. pylori*’s role in metaplastic progression and immune modulation. - Test interventions (e.g., antibiotics, immunotherapy) for preneoplastic reversal. - Validate *H. pylori* eradication as a strategy to prevent dysplasia. - Explore immune checkpoint inhibitors targeting PD-1/FOXP3 pathways. |
| Ref. (63) | - Mist1-CreERT; Kras (LSL-G12D); p53^fl/fl^; Klhl21^fl/fl^ (primary model for metaplasia-to-dysplasia transition). - Mist1-CreERT; Apc^fl^/^fl^; p53^fl^/^fl^; Klhl21^fl^/^fl^ (used for gastric adenocarcinoma). - Wild-type C57BL/6 (for HDT/MNU-induced dysplasia). - Il1rl1 (St2)-KO (to study IL-13/IL-33 signaling). - Control strains: Rosa26-LSL-Tdtomato (lineage tracing), Klhl21fl/+ (heterozygous controls). | - Methods:   - Kras-driven metaplasia: Mist1-CreERT; Kras (LSL-G12D) mice induced SPEM via tamoxifen.  - Chemical carcinogenesis: Wild-type C57BL/6 treated with high-dose tamoxifen (HDT) and N-methyl-N-nitrosourea (MNU) to induce dysplasia.  - Genetic deletion: Klhl21fl/fl crossed with Mist1-CreERT to study KLHL21’s role in STAT3-driven tumorigenesis.   - Principles:   - Kras activation drives SPEM (precancerous metaplasia).  - HDT induces acute, reversible SPEM; MNU causes DNA damage leading to dysplasia.  - Klhl21 deletion promotes STAT3 hyperactivation via PABPC1-PIK3CB axis, accelerating metaplasia-to-dysplasia transition. | - Diet: Not explicitly stated. - HDT/MNU administration: - HDT: Single high-dose tamoxifen (acute SPEM). - MNU: Cyclic administration in drinking water (chronic dysplasia). | - Histopathology: H&E staining for metaplasia (SPEM), dysplasia, and adenocarcinoma. - Immunostaining:   - SPEM markers: TFF2, MUC6, GS-II, GIF.  - Proliferation/DNA damage: Ki67, γ-H2AX, BrdU.  - STAT3 pathway: p-STAT3 (Tyr705), PIK3CB, PABPC1.   - Lineage tracing: Rosa26-Tdtomato in Mist1^+^ cells. - Organoid culture: Derived from metaplastic/dysplastic tissues. - Omics: scRNA-seq, ribo-seq, mass spectrometry (KLHL21-PABPC1 interaction). | - Advantages:   - Recapitulates human intestinal-type gastric carcinogenesis (metaplasia → dysplasia → cancer).  - Genetic (Klhl21 deletion) and chemical (HDT/MNU) models allow mechanistic studies.  - STAT3 inhibition (TTI-101) validates therapeutic targets.   - Disadvantages:   - Kras-driven SPEM alone does not progress to cancer (requires p53/Apc loss).  - MNU/HDT models are time-consuming (weeks to months).  - Pancreatic cancer in Mist1-CreERT; Kras (LSL-G12D) mice complicates survival studies. | - Metaplasia (SPEM): Acute HDT; 48–72 hrs post-HDT - Dysplasia: Genetic + tamoxifen; 60 days post-Tmx (Mist1-CreERT; Kras; p53^fl/fl^) - Adenocarcinoma: Genetic + tamoxifen; 60 days post-Tmx (Mist1-CreERT; Apc^fl/fl^; p53^fl/fl^) | Gastric corpus (isthmus and gland base). | Not explicitly detailed | - Inflammation: The article mentions the involvement of IL-33 and IL-13 in the development of metaplasia, suggesting an inflammatory context. - Inflammatory Cells: Innate lymphoid cell populations (ILC2s) are mentioned as stimulators of IL-33 and IL-13 release, which initiate SPEM. - Macrophages are not explicitly mentioned. | - To study the molecular mechanisms of gastric tumourigenesis, the role of specific genes like KLHL21, and the transition from metaplasia to dysplasia. - To test the efficacy of potential anti-cancer drugs, such as STAT3 inhibitors, in preventing or reversing gastric metaplasia and tumourigenesis. - To evaluate the therapeutic potential of targeting specific pathways involved in gastric cancer development. |
| Ref. (64) | - Primary Strain:  1. Tff2-CreERT2 knock-in mice (generated for this study, with Cre recombinase under the Tff2 promoter). 2. Crossed with:   - Rosa26-tdTomato (lineage tracing).  - KrasLSL-^G12D/+^ (oncogenic Kras mutation).  - Lgr5-DTR-GFP (chief cell ablation).  - Gif-rtTA-eGFP (chief cell-specific labeling).  - Rosa26-DTA (diphtheria toxin-mediated ablation of Tff2^+^ cells).   - Control Strains:   - Wild-type (WT) C57BL/6J.  - Tff2-CreERT; Kras^+/+^; Rosa26-tdTomato. | - Acute Injury Models (SPEM Induction):   - High-dose tamoxifen (HDT, 300 mg/kg, 2 doses) → Parietal & chief cell loss → SPEM.  - DMP777 (500 mg/kg, oral gavage) → Parietal cell depletion → SPEM.  - Diphtheria toxin (DT) in Lgr5-DTR-GFP mice → Chief cell ablation → Enhanced SPEM from Tff2^+^ progenitors.   - Chronic Injury Models (Dysplasia/Cancer Progression):   *- H. pylori* infection (SS1 strain, 2×10^9^ CFU, 3 doses) → Chronic gastritis → Metaplasia → Low-grade dysplasia.  - Kras(G12D) mutation in Tff2^+^ progenitors → Direct dysplasia bypassing metaplasia.  - Combined *H. pylori* + Kras(G12D) → Accelerated moderate/severe dysplasia (3 months).   - Principle:   - Tff2^+^ isthmus progenitors (not chief cells) are the primary source of SPEM after injury.  - Kras mutation drives dysplasia directly from progenitors, while SPEM is a terminal, non-proliferative state. | - Standard SPF conditions (specific pathogen-free). - Doxycycline (0.2 mg/mL in drinking water) for Gif-rtTA and tetO-Cre systems. - Normal chow diet, no dietary modifications reported. | - Histopathology: H&E, PAS/Alcian blue (mucins), Steiner stain (*H. pylori*). - Immunofluorescence (IF):   Markers: TFF2, GIF, GS-II, CD44v9, Ki-67, TROP2 (dysplasia), OLFM4 (stemness).   - Lineage Tracing: tdTomato+ cell tracking post-tamoxifen. - Single-cell RNA-seq: Identified progenitor → SPEM → dysplasia trajectories. - Spatial Transcriptomics: Human/mouse tissue mapping of TFF2^+^ cells. - Organoid Culture: Proliferation/differentiation assays of Tff2^+^ progenitors. | - Advantages:   - Challenges the "chief cell transdifferentiation" dogma; shows Tff2^+^ progenitors as SPEM origin.  - Demonstrates direct dysplasia from progenitors (bypassing metaplasia).  - Human-mouse translational relevance (validated by scRNA-seq/spatial transcriptomics).   - Disadvantages:   - Tff2-CreERT labels other tissues (pancreas, lung, duodenum), reducing specificity.  - Mouse models may not fully replicate human gastric carcinogenesis (e.g., no intestinal metaplasia reported).  - Limited mechanistic insights into Tff2 downregulation in dysplasia. | - Acute Injury (SPEM): HDT/DMP777/DT; SPEM peaks at 3–7 days - Chronic SPEM: *H. pylori* infection; Metaplasia at 2 months - Low-grade Dysplasia: *H. pylori* alone; 6 months - Moderate/Severe Dysplasia: Kras(G12D) + *H. pylori*; 3 months (accelerated) - Cancer (in situ): Kras(G12D); 6–12 months | - Exclusively gastric corpus (body). - No involvement of the antrum in metaplasia/dysplasia. | The article does not provide specific information on the invasiveness or metastatic potential of gastric tumors in these models. | - *H. pylori* infection:   Chronic inflammation with immune cell infiltration (exact subtypes not specified).   - Immune factors:   - TFF2 has anti-inflammatory roles; its loss exacerbates *H. pylori*-driven carcinogenesis.  - No detailed immune profiling (e.g., macrophages, T cells) was performed. | - Study SPEM origins (progenitor vs. chief cell plasticity). - Investigate Kras-driven dysplasia bypassing metaplasia. - Test preventive/therapeutic strategies targeting Tff2^+^ progenitors. - Lineage tracing: Tff2-CreERT; tdTomato for gastric cell fate mapping. - Drug screening: DMP777/HDT for acute injury repair mechanisms. - Oncogenic modeling: Kras(G12D) + *H. pylori* for accelerated dysplasia. |

**Table 7:** Summary of Tumor Suppressor Gene Mutation–Induced Animal Models for Gastric Precancerous Lesions

| Studies | Animal Model | Modeling Method and Principle | Feeding Conditions | Model Evaluation Methods | Advantages and Disadvantages | Detailed Time Course of Pathological Stages | Anatomical Sites of Lesions | Tumor Location and Invasion/Metastasis | Inflammation and Immune Cell Infiltration | Application Scenarios for Models |
| --- | --- | --- | --- | --- | --- | --- | --- | --- | --- | --- |
| Ref. (65) | - Primary Strain Used: BALB/c background Runx3-deficient mice (Runx3^−/−^). - Comparison Strain: Wild-type (WT) BALB/c mice. - Other Strains Mentioned:   - C57BL/6 background Runx3^−/−^ mice (neonatal lethality noted).  Apc^Min/+^ mice (for Wnt pathway comparison). | - Method:   Genetic - Knockout: Runx^−/−^ mice were generated to study spontaneous precancerous changes.  - Chemical Induction: N-methyl-N-nitrosourea (MNU) was administered via drinking water (120 ppm for 10 weeks, starting at 8 weeks of age) to accelerate tumorigenesis in Runx3^−/−^ mice.  Principle:   1. Runx3 is a tumor suppressor in gastric epithelium. Its loss leads to:   - Hyperplasia due to increased proliferation and reduced apoptosis.  - Metaplasia (SPEM: spasmolytic polypeptide-expressing metaplasia) with Muc6/TFF2 expression.  - Intestinal phenotype (Cdx2/Muc2 upregulation) via Wnt/β-catenin pathway activation.   1. MNU acts as a mutagen, synergizing with Runx3 deficiency to induce adenocarcinoma. | - Standard Diet: Not explicitly described, but mice were housed under standard laboratory conditions. - MNU Treatment: Administered in drinking water (120 ppm) for 10 weeks (ages 8–18 weeks). Mice were sacrificed at 52 weeks (1 year) for analysis. | - Histopathology: H&E staining for hyperplasia, metaplasia, dysplasia, and adenocarcinoma. - Immunohistochemistry (IHC):   - Markers: Pepsinogen (chief cells), Muc6/TFF2 (SPEM), Muc2/Cdx2 (intestinal metaplasia), - Ki67/BrdU (proliferation), caspase assays (apoptosis).  - β-catenin localization (nuclear accumulation indicates Wnt activation).   - Molecular Analysis:   - Western blot/qRT-PCR: Wnt targets (Axin2, c-Myc, CD44), inflammatory cytokines (IL-6, TNF-α, COX-2, IL-1β).  - Chromatin immunoprecipitation (ChIP): β-catenin/Tcf4 binding to Cdx2 promoter.   - Functional Assays:   - Luciferase reporter assays for Cdx2 promoter activity.  - Tumorigenicity in nude mice (GIF cell lines). | - Advantages:   - Recapitulates human gastric carcinogenesis stages (hyperplasia → metaplasia → dysplasia → cancer).  - Runx3 loss mimics frequent epigenetic silencing in human gastric cancer.  - SPEM and intestinal phenotype align with human precancerous lesions.  - MNU accelerates tumor formation, enabling shorter study timelines.   - Disadvantages:   - Limited spontaneous tumorigenesis (requires MNU for consistent adenocarcinoma).  - BALB/c Runx3^−/−^ mice survive only ~1 year, restricting long-term studies.  - No *Helicobacter pylori* involvement, unlike human gastric cancer etiology. | - 6 Months (Adult Mice):   - Chief Cell Loss: Pepsinogen-negative, Muc6/TFF2-positive SPEM.  - Hyperplasia: Increased Ki67/BrdU labeling in fundic glands.  - Intestinal Phenotype: Cdx2/Muc2 expression (no goblet cells).   - 10 Months:   Dysplastic lesions observed in Runx3^−/−^ stomachs.   - 52 Weeks (MNU-Treated):   Adenocarcinoma: Invasive tumors in fundus/pylorus (60–80% incidence in Runx3^−/−^). | - Fundic Glands: Primary site for SPEM, hyperplasia, and adenocarcinoma. - Pyloric Glands: Less affected by metaplasia but showed increased proliferation (BrdU^+^ cells). | - Invasiveness:   - Adenocarcinomas invaded submucosa and muscle layers.  - Confirmed by cytokeratin 8^+^ cells in muscularis mucosae.   - Metastasis: Not reported in this study. | - No lymphocyte infiltration or elevated proinflammatory cytokines (IL-6, TNF-α, COX-2, IL-1β) in Runx3^−/−^ stomachs. - Bone marrow transplantation experiments confirmed inflammation-independent SPEM. | - Study early molecular events in gastric carcinogenesis (Wnt/β-catenin, Cdx2 dysregulation). - Test chemopreventive/therapeutic agents targeting metaplasia or Wnt signaling. - Investigate Runx3’s role in TGF-β resistance and apoptosis evasion. - Not suitable for *H. pylori*-related studies or inflammation-driven models. - BALB/c background may limit comparative studies with C57BL/6-based models. |
| Ref. (66) | - Primary Strain Used: C57BL/6 background RUNX3^R122C^ knock-in mice (homozygous: RUNX3^R122C/R122^; heterozygous: RUNX3^R122C/+^). - Reporter Strain: Crossed with Iqgap3-2A-tdTomato mice to label isthmus stem/progenitor cells. - Control Strain: Wild-type (WT) C57BL/6 mice. | - Genetic Knock-in: Introduced the R122C mutation in the DNA-binding Runt domain of RUNX3 via homologous recombination. - Principle:   RUNX3^R122C^ disrupts the tumor-suppressor function of RUNX3 by impairing interactions with BRD2 and p300, leading to:  - Dysregulated cell-cycle restriction point (R-point): Uncontrolled proliferation of isthmus stem/progenitor cells.  - Maturation arrest: Failure of terminal differentiation into pit, parietal, and chief cells.  - Metaplasia: SPEM (spasmolytic polypeptide-expressing metaplasia) with Muc6/TFF2 expression.  - Inflammation: Increased macrophage infiltration and proinflammatory cytokines (IL-6, TNF-α). | Mice housed under standard conditions with ad libitum access to water and chow. | - Histopathology: H&E and periodic acid–Schiff (PAS) staining for hyperplasia and metaplasia. - Immunofluorescence (IF):   - Proliferation: Ki67, MCM2.  - Differentiation Markers: Atp4a (parietal cells), GIF (chief cells), Muc5ac (pit cells), GSII (mucous neck cells).  - Metaplasia: TFF2, Muc6, CD44v10.  - Inflammation: CD45 (leukocytes), CD68/F4/80 (macrophages).   - Organoid Culture: Assessed stem cell activity using corpus-derived organoids. - Flow Cytometry: Sorted Iqgap3-tdTomato^high^ isthmus stem cells for transcriptomic analysis. - RNA-seq & GSEA: Identified enriched pathways (e.g., MYC targets, IFN-γ response). - Co-immunoprecipitation: Validated disrupted RUNX3^R122C^-BRD2/p300 interactions. | - Advantages:   - Recapitulates human gastric precancerous lesions (SPEM, hyperplasia) driven by RUNX3 mutation.  - Demonstrates inflammation-independent and -dependent phases of metaplasia.  - Iqgap3-tdTomato reporter enables precise isolation of stem/progenitor cells.   - Disadvantages:   - Low penetrance of macroscopic lesions (7/22 mice at 6 months).  - No spontaneous adenocarcinoma development (even at 1.5 years).  - C57BL/6 background may limit comparative studies with BALB/c models. | - 6–8 Weeks: Normal gastric morphology in all genotypes. - 6 Months:   RUNX3^R122C/R122C^ Mice:  - Hyperplasia: Ki67^+^ cells expanded throughout glands.  - SPEM: TFF2^+^/Muc6^+^ metaplasia, loss of chief/parietal cells.  - Inflammation: CD68^+^/F4/80^+^ macrophages (MZ-type dominant).   - >1 Year: No tumors observed, suggesting need for secondary hits. | Primary Site: Corpus (fundic region). | Even in aged mice (>1.5 years), suggesting the model captures early precancerous stages only. | - Macrophages: Increased CD68^+^ /F4/80^+^ cells, predominantly MZ-type (F4/80^+^/CD163^+^). - Cytokines: Elevated IL-6, TNF-α, IL-1β, Cox-2 mRNA. | - Study early RUNX3-driven dysregulation of stem cell proliferation/differentiation. - Investigate R-point dysfunction in precancerous metaplasia. - Test interventions targeting MYC or Wnt pathways. - Not suitable for *H. pylori* co-culture studies or late-stage cancer modeling. |
| Ref. (67) | - Strain: p27-deficient (p27^−/−^) mice backcrossed to the C57BL/6 background. - Control: Wild-type C57BL/6 mice. | - Method:   - Mice (6–8 weeks old) were orally gavaged with 0.1 mL PBS containing 10^9^ colony-forming units of *H. pylori* (mouse-adapted Sydney strain, SS1) three times over 5–7 days.  - Uninfected controls received PBS alone.   - Principle:   - p27kip1 is a tumor suppressor regulating cell cycle, apoptosis, and inflammation. Its deficiency leads to increased proliferation and decreased apoptosis in gastric epithelium.  *- H. pylori* infection further exacerbates these effects, promoting inflammation, metaplasia, dysplasia, and cancer. | - Diet: Harlan Teklad Global Diet 2018 (standard chow), provided ad libitum. - Housing: Specific pathogen-free (SPF) conditions in microisolator cages. | - Histopathology:   - Stomachs were scored for hyperplasia, dysplasia (0–4 scale), and inflammation (Sydney system, 0–3).  - Intestinal metaplasia was confirmed via Alcian blue/periodic acid–Schiff staining.   - Cell Dynamics:   - Proliferation: Bromodeoxyuridine (BrdU) labeling index.  - Apoptosis: TUNEL assay and cleaved caspase-3 staining.   - Bacterial Load:   - Quantitative PCR for *H. pylori* SSA gene.  - Immunohistochemistry for bacterial density scoring (0–4).   - Serology:   - Anti-*H. pylori* IgG, IgG1 (Th2), and IgG2c (Th1) ELISA.  - Serum gastrin levels by radioimmunoassay. | - Advantages:   - Recapitulates human intestinal-type gastric carcinogenesis (metaplasia → dysplasia → cancer).  - No spontaneous cancer in uninfected p27^−/−^ mice, ensuring *H. pylori*-specific effects.  - Useful for studying chemoprevention and eradication strategies.   - Disadvantages:   - Long latency (60–75 weeks for cancer).  - No metastatic spread observed.  - Requires SPF conditions and specialized *H. pylori* strain (SS1). | - Uninfected p27^−/−^ Mice:   - 15 weeks: Mild hyperplasia.  - 45 weeks: Focal metaplasia/low-grade dysplasia (rare).   - *H. pylori*-Infected p27^−/−^ Mice:   - 15 weeks: Moderate inflammation, hyperplasia, early mucous metaplasia.  - 30 weeks: Intestinal metaplasia (40%), mild dysplasia (10%).  - 45 weeks: Intestinal metaplasia (67%), dysplasia (17%).  - 60 weeks: High-grade dysplasia/intramucosal carcinoma (83%).  - 75 weeks: Polypoid adenomas, intramucosal carcinoma (33%).   - Wild-Type Mice:   Only mild metaplasia at 75 weeks (50%); dysplasia rare (13%). | Corpus and antrum (both involved). | - Invasiveness: Intramucosal carcinoma observed (penetration of muscularis mucosae). - Metastasis: Not reported (no metastatic lesions described). | - Inflammatory Cells:   - Mononuclear cells (lymphocytes, plasma cells).  - Neutrophils (acute inflammation).  - Lymphoid follicles (more frequent in p27^−/−^ mice).   - Immune Factors:   No Th1/Th2 skewing (IgG1/IgG2c ratios similar to wild-type). | - Study *H. pylori*-induced carcinogenesis mechanisms (e.g., p27 role in apoptosis/proliferation). - Test chemopreventive agents or eradication therapies. - Investigate host-pathogen interactions (immune response, bacterial virulence factors). - Validate biomarkers for early gastric cancer detection. - Evaluate therapeutic targets (e.g., Skp2-p27 degradation pathway). |
| Ref. (68) | - Primary Model:   - USF1-deficient mice (Usf1^⁻/⁻^) on a mixed C57BL/6 × 129Sv background.  - Control: Wild-type (Usf1^⁺/⁺^) littermates.   - Additional Model:   INS-GAS mice (transgenic mice overexpressing gastrin, used to validate findings). | - Method:   - Mice were orally infected with *H. pylori* SS1 strain at 6–8 weeks of age.  - Infection status and histopathology were monitored at 9 and 12 months post-infection (pi).   - Principle:   - USF1 is a transcription factor that stabilizes p53, a tumor suppressor critical for DNA repair.  *- H. pylori* infection downregulates USF1, leading to p53 degradation, impaired DNA repair, and accumulation of mutations.  - USF1 deficiency exacerbates *H. pylori*-induced gastric lesions by disrupting p53-mediated genome stability. | - Diet: Standard chow (specific diet not detailed in the study). - Housing: Specific pathogen-free (SPF) conditions. | - Histopathology:   H&E staining for inflammation, metaplasia, and dysplasia.   - Immunofluorescence (IF) for USF1 and p53 localization. - Molecular Analysis:   - qRT-PCR and Western blot for USF1, TP53, and target genes (e.g., CSA, HR23A, GADD45A).  - Proximity ligation assay (PLA) to detect USF1/p53 nuclear complexes.   - DNA Damage Assessment:   γH2AX staining (marker of DNA double-strand breaks).   - Human Data Correlation:   TCGA analysis of USF1 and TP53 expression in gastric cancer (GC) patients. | - Advantages:   - Recapitulates human intestinal-type gastric carcinogenesis (metaplasia → dysplasia → cancer).  - Demonstrates USF1’s tumor-suppressive role *in vivo*.  - Links *H. pylori* infection to p53 degradation and genomic instability.   - Disadvantages:   - Long latency (9–12 months for significant lesions).  - No metastasis observed (limited to intramucosal carcinoma).  - Mixed genetic background (Usf1^⁻/⁻^ mice) may introduce variability. | - Uninfected Usf1^⁻/⁻^ Mice:   No significant lesions (baseline hyperplasia minimal).   - *H. pylori*-Infected Usf1^⁻/⁻^ Mice:  1. 9 months pi:   - Intestinal metaplasia (score: 2.5) and parietal cell loss.  - Focal dysplasia (absent in wild-type mice).   1. 12 months pi:   Severe dysplasia and intramucosal carcinoma.   - Wild-Type Mice:   Only mild metaplasia at 12 months pi (no significant dysplasia). | - Primary Site: Corpus and antrum (diffuse involvement). - Metaplasia/Dysplasia: Observed in both regions. | - Invasiveness: Intramucosal carcinoma (penetrates muscularis mucosae). - Metastasis: Not reported (study focused on early carcinogenesis). | - Inflammation Severity:   More severe in Usf1^⁻/⁻^ mice vs. wild-type (higher scores at 9/12 months pi).   - Immune Cells:   - Mononuclear infiltrates (lymphocytes, plasma cells).  - Neutrophils (acute inflammation).   - Cytokines:   Th1/Th2 balance (assessed via serum IgG1/IgG2c) unchanged between strains. | - Study USF1/p53 axis in *H. pylori*-induced genomic instability. - Test chemopreventive agents targeting USF1 stabilization or p53 pathways. - USF1 expression as a biomarker for GC risk in *H. pylori*-infected patients. - Identify high-risk patients for early intervention (e.g., *H. pylori* eradication). |

**Table 8:** Summary of Glandular Cell Injury–Induced Animal Models for Gastric Precancerous Lesions

| Studies | Animal Model | Modeling Method and Principle | Feeding Conditions | Model Evaluation Methods | Advantages and Disadvantages | Detailed Time Course of Pathological Stages | Anatomical Sites of Lesions | Tumor Location and Invasion/Metastasis | Inflammation and Immune Cell Infiltration | Application Scenarios for Models |
| --- | --- | --- | --- | --- | --- | --- | --- | --- | --- | --- |
| Ref. (69) | - Strain Name: Stomach-type claudin-18 knockout mice (Cldn18^⁻/⁻^). - Genetic Background: Generated by targeting the stomach-specific isoform of claudin-18 (variant 2). | - Method: Genetic knockout of stomach-type claudin-18 (Cldn18^⁻/⁻^). - Principle:   - Claudin-18 is a tight junction (TJ) protein critical for forming a paracellular barrier against H⁺ leakage in gastric epithelia.  - Its deficiency causes H⁺ leakage, leading to chronic acid-induced inflammation (gastritis), cytokine upregulation (e.g., IL-1β), and metaplasia (SPEM), which are precancerous conditions. | - No specific diet mentioned; mice were maintained under conventional laboratory conditions. - Interventions:   - Acid Challenge: Oral administration of hydrochloric acid (low concentration) on postnatal day 1 to induce inflammation.  - Omeprazole Treatment: Administered twice daily for 2 weeks to inhibit H⁺ secretion and assess acid dependency of gastritis. | - Histology: H&E staining, immunofluorescence (e.g., H⁺/K⁺-ATPase, pepsin C, TFF2). - Electron Microscopy: Freeze-fracture and ultrathin-section analysis of TJ morphology. - Electrophysiology: Transepithelial conductance and H⁺ titration to measure paracellular H⁺ leakage. - Molecular Analysis: qRT-PCR for cytokine/gene expression (IL-1β, COX-2, KC, etc.), ELISA for serum cytokines. - Flow Cytometry: Immune cell profiling (e.g., Gr-1⁺ neutrophils). | - Advantages:   - Direct link between TJ dysfunction (claudin-18 loss) and gastritis, mimicking human atrophic gastritis.  - Spontaneous progression to metaplasia without external carcinogens.  - Useful for studying H⁺-mediated inflammation and precancerous changes.   - Disadvantages:   - No progression to gastric cancer observed (limited to metaplasia).  - Requires genetic manipulation; not suitable for acute studies. | - Postnatal Day 3: Onset of gastric acid secretion and initial H⁺ leakage. - Postnatal Day 4: First detectable decrease in parietal cells. - Postnatal Day 7: Significant parietal cell loss and gastritis onset. - Postnatal Day 14: Fully developed atrophic gastritis with SPEM. - Adulthood (8–20 weeks): Chronic gastritis with neutrophil infiltration, metaplasia, but no neoplasia. | - Corpus (Body): Severe pathology (parietal cell loss, SPEM, inflammation). - Antrum: Mild inflammation (IL-1β upregulation) but no metaplasia. | No invasive or metastatic tumors reported in Cldn18^⁻/⁻^ mice (up to 20 weeks). | - Inflammatory Cells:   - Dominant: Neutrophils (Gr-1⁺/CD11b⁻).  - Minor: No significant macrophage or lymphocyte infiltration.   - Cytokines: Upregulated IL-1β, TNF-α, COX-2, KC (neutrophil chemoattractant). | - To investigate the mechanisms of chronic gastritis and the role of tight junction proteins in maintaining gastric epithelial barrier function. - For testing potential anti-inflammatory or barrier-protective compounds. - To understand the progression from gastritis to metaplasia and the potential for dysplasia and neoplasia. |
| Ref. (70) | - Strain Name: CLDN18-knockout mice (CLDN18KM), generated on a B6:129S5/SvEvBrd background. - Genetic Modification: Targeted deletion of CLDN18 (both stomach- and lung-specific isoforms, CLDN18A1/A2). | - Method: Genetic knockout of CLDN18 (both isoforms). - Principle:   - Loss of CLDN18 disrupts basolateral tight junction integrity, leading to:  - Increased proliferation (via CD44, EPHB2, YAP1/HIPPO pathways).  - Metaplasia (SPEM) → Dysplasia → Neoplasia (independent of *H. pylori*).   - Unlike Cldn18A2-specific KO (Hayashi et al.(69)), this model progresses to invasive cancer due to complete CLDN18 loss. | - Standard Diet: No specific diet mentioned; mice housed under conventional conditions. - Interventions:   - *H. pylori* infection (strains SS1/PMSS1) at 6 weeks old (for comparison with CLDN18KM).  - No colonization in CLDN18KM: *H. pylori* failed to colonize due to mucosal changes. | - Histopathology: H&E, Alcian Blue/PAS staining, dysplasia scoring. - Immunostaining: Proliferation (Ki67), metaplasia (GSII/UEAI), stem cell markers (CD44, EPHB2). - Electron Microscopy: Ultrastructural defects in glandular epithelium. - RNAseq: Pathway analysis (Wnt, YAP1, IL-33, p53). - Flow Cytometry: Limited immune cell profiling (neutrophils dominant). | - Advantages:   - Spontaneous progression to invasive gastric cancer without carcinogens.  - Recapitulates human CLDN18 loss in gastric cancer (poor prognosis subset).  - Useful for studying basolateral TJ dysfunction in tumorigenesis.   - Disadvantages:   - No *H. pylori* colonization in CLDN18KM (limits infection-cofactor studies).  - Slow progression (tumors at 2 years). | - 7–10 weeks:   - SPEM (TFF2^+^/GSII^+^ metaplasia).  - Increased proliferation (Ki67+ cells in glands).   - 20–30 weeks:   - High-grade dysplasia (gland distortion, nuclear crowding).  - Early submucosal invasion.   - 2 years:   - Polypoid tumors (85% glandular involvement).  - Serosal invasion (no distant metastasis). | - Corpus (Body):   Severe pathology (SPEM, dysplasia, tumors).   - Antrum:   Minimal changes (no metaplasia). | - Invasive Glands: Present in submucosa (30 weeks) and serosa (2 years). - No Metastasis: Despite lymphatic invasion, no lymph node/distant organ involvement. | - Immune Cells:   - Neutrophils (dominant; CXCL1/KC upregulation).  - No significant macrophages/T-cells (unlike H. pylori models).   - Cytokines:   - Upregulated: IL-1α/β, IL-33, CXCL1/5.  - No change: TNF-α, IFN-γ, IL-6, IL-17. | - To investigate the mechanisms of gastric cancer development in the absence of CLDN18. - For testing potential anti-cancer or anti-proliferative compounds. - To understand the progression from premalignant lesions to neoplasia and the role of various signaling pathways. |
| Ref. (71) | - stCldn18^-/-^ mice: Stomach-type claudin-18 knockout mice - Wnt1-Tg mice: Wnt1-overexpressing transgenic mice. - Double-mutant (DM) mice: Cross between stCldn18^-/-^ and Wnt1-Tg mice. | - Method: Genetic knockout of stomach-type claudin-18 (stCldn18), a tight junction protein critical for gastric epithelial barrier function. - Principle:   - stCldn18 deficiency causes paracellular H^+^ leakage, leading to chronic active gastritis.  - Chronic inflammation activates cytokine-, stemness-, and Wnt-signaling pathways, promoting gastric tumorigenesis independent of *H. pylori* infection. | - Mice were bred and maintained in specific pathogen-free (SPF) conditions. - Confirmed negative for *Helicobacter pylori* infection. | - Histopathology: Hematoxylin and eosin (H&E) staining for gastritis and tumor assessment. - Immunofluorescence/Immunohistochemistry: Detection of markers like Ki-67 (proliferation), CD44 (stemness), CXCL5 (EMT), and β-catenin (Wnt signaling). - Flow cytometry: Quantification of immune cell infiltration (e.g., Gr1^+^ neutrophils, CD3^+^ T lymphocytes). - qRT-PCR: Analysis of inflammatory cytokines (e.g., IL-1β, TNF-α, CCL28, CXCL5) and stemness markers (e.g., SOX2, c-Myc). - Administration of inhibitors: POL7085 (CCL28-CCR10 cascade inhibitor) and cimetidine (H2-blocker) to validate mechanistic roles. | - Advantages:   - Recapitulates human gastric tumorigenesis without *H. pylori* infection, isolating the role of epithelial barrier dysfunction.  - Demonstrates multistage progression from gastritis to tumors, useful for studying microenvironmental contributions.  - DM mice (stCldn18^-/-^ × Wnt1-Tg) show accelerated tumorigenesis, enabling rapid studies.   - Disadvantages:   - Tumor incidence is 20–30% in aged stCldn18^-/-^ mice, requiring large cohorts for statistical power.  - Limited metastasis or invasive cancer observed, restricting studies on advanced disease. | - stCldn18-/- mice:   - 8 weeks: Acute oxyntic atrophic gastritis (pseudopyloric metaplasia, H^+^ leakage).  - 40 weeks: Transition to chronic active gastritis (lymphocyte infiltration, CCL28 expression).  - 60–100 weeks: Gastric tumors (20–30% incidence, CXCL5+ EMT, SPEM cells).   - DM mice (stCldn18^-/-^ × Wnt1-Tg):   40 weeks: 100% tumor incidence with dysplastic changes. | - stCldn18^-/-^ mice: Pathologies primarily observed in the gastric corpus (oxyntic mucosa). - DM mice: Tumors develop in the corpus with back-to-back glandular dysplasia. | - No evidence of invasion or metastasis in stCldn18^-/-^ or DM mice. - Ectopic gastric glands (resembling "gastritis cystica profunda") were noted in aged stCldn18^-/-^ mice but lacked nuclear atypia. | - stCldn18^-/-^ mice:   - 8 weeks: Gr1^+^ neutrophils dominate (acute gastritis).  - 40–100 weeks: CD3^+^ T lymphocytes (CD4^+^, CD8^+^), neutrophils, and CCL28^+^ cells (chronic active gastritis).   - DM mice: Similar immune infiltration but accelerated CXCL5^+^ EMT. | - Study epithelial barrier dysfunction in gastric tumorigenesis. - Investigate roles of Wnt signaling, cytokines (e.g., CCL28, CXCL5), and stemness pathways (e.g., CD44, TLR2). - Test therapeutic targets (e.g., POL7085 for CCL28 inhibition, cimetidine for acid suppression). - Compare *H. pylori*-independent vs. -dependent carcinogenesis mechanisms. |
| Ref. (72) | - AR^⁻/⁻^ mice: Amphiregulin knockout mice (C57BL/6 background). - TGF-α^⁻/⁻^ mice: Transforming growth factor-alpha knockout mice (control). - Wild-type (WT) mice: C57BL/6 mice (control). | - Method: Genetic knockout of amphiregulin (AR), an epidermal growth factor (EGF) receptor ligand secreted by parietal cells. - Principle:   - AR deficiency leads to oxyntic atrophy (loss of parietal cells) and spontaneous spasmolytic polypeptide-expressing metaplasia (SPEM).  - SPEM progresses to intestinal metaplasia and invasive fundic neoplasia over time, mimicking human gastric carcinogenesis. | - Mice were maintained in specific pathogen-free (SPF) conditions in sterile microisolator cages. - Fed regular mouse chow and water ad libitum. - Confirmed negative for *Helicobacter* infection. | - Gross pathology: Macroscopic tumor observation in the fundus. - Histopathology: H&E staining for metaplasia (SPEM, intestinal metaplasia) and neoplasia. - Immunohistochemistry:   - TFF2/SP (SPEM marker), MUC2/TFF3 (intestinal metaplasia markers), Pdx-1 (duodenal transcription factor).  - Ki-67 (proliferation), β-catenin (Wnt signaling).  - Dual immunofluorescence: Co-staining for TFF2/SP with MUC2 or TFF3 to track lineage progression.  - Cytokine assays: Serum and tissue mRNA levels of IL-2, IL-4, IL-5, TNF-α, IFN-γ (Th1/Th2 response). | - Advantages:   - First mouse model showing spontaneous progression from SPEM to intestinal metaplasia and neoplasia.  - Recapitulates human gastric carcinogenesis without *Helicobacter* infection.  - Demonstrates lineage plasticity (SPEM → intestinal metaplasia).   - Disadvantages:   - Tumor development requires long latency (18 months).  - Limited to fundic lesions (no antral pathology). | - AR^⁻/⁻^ mice:   - 10 months:  30% develop SPEM (TFF2^+^ cells).  - 30% show submucosal inflammation (neutrophils).  - 60% exhibit fundic hyperplasia.   - 18 months:   - 71% develop SPEM.  - 43% develop intestinal metaplasia (MUC2^+^/TFF3^+^ goblet cells).  - 29% show dysplasia and submucosal invasion. | - Exclusively fundic: - SPEM, intestinal metaplasia, and tumors occur in the gastric corpus. - Antrum remains histologically normal. | Invasive lesions:   - Submucosal invasion with cystic gland dilation and β-catenin cytoplasmic/nuclear localization (indicative of Wnt activation). - No evidence of distant metastasis. | AR^⁻/⁻^ mice:   - Submucosal infiltrates: Predominantly polymorphonuclear leukocytes (neutrophils). - Cytokines: Elevated Th1 (IL-2, TNF-α, IFN-γ) and Th2 (IL-4, IL-5) responses in serum and gastric tissue. | - Study metaplasia-to-neoplasia progression in gastric cancer. - Investigate roles of EGF receptor ligands (AR vs. TGF-α) in mucosal homeostasis. - Test therapeutic targets for SPEM/intestinal metaplasiaregression (e.g., anti-inflammatory agents). |
| Ref. (73) | - Complete knockout:   - Strain: Congenic on an S129/svJ background).  - Nomenclature: Slc26a9^⁻/⁻^ mice.   - Parietal cell-specific knockout:   - Strain: Mixed genomic background (C57BL/6J × S129/svJ).  - Nomenclature: Slc26a9^fl/fl^/Atp4b-Cre mice. | - Method:   Genetic deletion of Slc26a9 (complete or parietal cell-specific).   - Principle:  1. Slc26a9 encodes a Cl⁻ uniporter critical for parietal cell function. Its loss leads to:   - Parietal cell atrophy → hypochlorhydria → hypergastrinemia.  - Disrupted gastric stem cell differentiation → metaplasia → neoplasia (via Wnt/β-catenin activation, inflammation, and apoptosis inhibition).   1. Recapitulates the Correa cascade (atrophic gastritis → metaplasia → dysplasia → cancer). | - Standard housing under specific pathogen-free (SPF) conditions. - No dietary or chemical induction (spontaneous model). - Age-matched cohorts analyzed from 8 days to 18 months. | - Histopathology:   - Roger’s criteria for scoring premalignant/malignant lesions.  - Staining for markers of atrophy (H^+^/K^+^-ATPaseβ), metaplasia (TFF2, MUC6, CDX2), proliferation (Ki67), and apoptosis (TUNEL).   - Molecular analysis:   RNA microarray, qRT-PCR, IHC, and ISH for gene expression (e.g., Wnt1, β-catenin, Cyclin D1).   - Human correlation:   Tissue microarrays and primary gastric cancer samples analyzed for SLC26A9 expression and survival. | - Advantages:   - Spontaneous progression mimics human Correa sequence.  - Parietal cell-specific knockout confirms cell-autonomous effects.  - Reproducible timeline of lesions (useful for intervention studies).   - Disadvantages:   - Long latency (neoplasia at 14–18 months).  - No metastasis observed (limited to intramucosal lesions).  - Mixed genetic background in Slc26a9^fl/fl^/Atp4b-Cre mice may introduce variability. | - Complete knockout (Slc26a9^⁻/⁻^):   - 8 days: Normal mucosa.  - 1 month: Parietal cell loss.  - 2 months: Oxyntic atrophy (parietal/chief cell loss), dilated glands.  - 6 months: SPEM (TFF2^+^/MUC6^+^) and intestinal metaplasia (CDX2^+^/MUC2^+^).  - 14 months: High-grade intraepithelial neoplasia (HGIN), chronic inflammation, cyst formation.  - 18 months: Moderately differentiated gastric carcinoma (4/13 mice).   - Parietal cell-specific knockout (Slc26a9^fl/fl^/Atp4b-Cre):   - Similar timeline but with delayed surface epithelial defects (MUC5AC/Claudin 18.2 loss at 18 months).  - Poorly differentiated carcinoma at 18 months. | - Complete knockout:   Gastric corpus (fundus) primarily affected .   - Parietal cell-specific knockout:   Lesions also localized to the corpus.   - No explicit mention of antrum involvement. | No submucosal invasion, lymph node, or distant metastases observed in either model | - Complete knockout:   - Chronic inflammation with upregulated IL-17, IL-11, IL-1β.  - Immune cells not specified, but typical correlates include macrophages and lymphocytes (implied by cytokine profile).   - Parietal cell-specific knockout:   Similar inflammatory milieu but delayed. | - Role of ion transporters (SLC26A9, AE2) in gastric carcinogenesis. - Wnt/β-catenin signaling in metaplasia-neoplasia transition. - Interventions targeting inflammation, Cl⁻/HCO₃⁻ transport, or stem cell dysregulation. |
| Ref. (74) | Parietal cell-specific knockout:   - Strain: C57BL/6J × S129/svJ mixed background. - Nomenclature: GRIM-19^flox/flox^/Atp4b-Cre mice (crossed with Atp4b-Cre mice). - Control: GRIM-19^flox/flox^ littermates. | - Method: Conditional knockout of GRIM-19 in parietal cells. - Principle:  1. GRIM-19 (NDUFA13) is a mitochondrial complex I subunit; its loss triggers:   - ROS overproduction → oxidative stress → DNA damage.  - NRF2-HO-1 axis activation → aberrant NF-κB signaling.  - NLRP3 inflammasome activation → IL-33 release → SPEM transdifferentiation.   1. Recapitulates chronic atrophic gastritis → SPEM progression (pre-neoplastic stage). | - SPF (specific pathogen-free) housing. - No chemical/dietary induction (spontaneous model). - Age range: 8–10 weeks (intervention studies) to 18 weeks (pathology analysis). | - Histopathology:   - H&E staining for gastric mucosa thickness, inflammatory infiltration, and SPEM markers (TFF2, Clusterin-1).  - Immunofluorescence (IF) for parietal cells (VEGF-B), macrophages (F4/80, CD45), and SPEM transitional cells (GIF^+^/GSII^+^).   - Molecular analysis:   - Western blot for NRF2, HO-1, NLRP3, IL-33, NF-κB/p65.  - Luciferase assays for NRF2/ARE and NF-κB transcriptional activity.  - ELISA for inflammatory cytokines (IL-1β, IL-6, TNF-α).   - Intervention:   NLRP3 inhibitor MCC950 (10 mg/kg, i.p., 3×/week for 4 weeks). | - Advantages:   - Spontaneous SPEM without parietal cells loss, mimicking early human chronic atrophic gastritis.  - Clear ROS-NRF2-HO-1-NF-κB-NLRP3/IL-33 axis elucidated.  - Therapeutic validation via MCC950 (translational potential).   - Disadvantages:   - No intestinal metaplasia observed (limited to SPEM).  - No metastasis reported (early-stage model).  - Requires long-term aging for full pathology (18 weeks). | - GRIM-19^flox/flox^/Atp4b-Cre mice:  1. 8–10 weeks:   - Intact parietal cells (no apoptosis).  - Mild inflammatory infiltration (CD45^+^, MPO^+^ cells).   1. 14–18 weeks:   - Thickened gastric mucosa.  - SPEM markers: ↑TFF2, Clusterin-1, HE4.  - GIF^+^/GSII^+^ transitional SPEM cells.  - No intestinal metaplasia (CDX2/KLP4 unchanged). | - Gastric corpus (fundus) exclusively. - No antral involvement reported. | No invasion or metastasis observed (intramucosal lesions only). | - Inflammatory infiltrates:   - Neutrophils (MPO^+^) and macrophages (F4/80^+^).  - M1 polarization ↑ (iNOS^+^), M2 ↓ (CD206^+^).   - Cytokines:   - ↑IL-1β, IL-6, TNF-α, VEGF.  - IL-33 (key driver of SPEM). | - To investigate the mechanisms of chronic atrophic gastritis and SPEM pathogenesis. - For testing potential therapeutic compounds targeting GRIM-19, NLRP3, or IL-33. - To understand the role of GRIM-19 in maintaining gastric mucosa integrity and preventing metaplasia. |
| Ref. (75) | - Wild-type (WT): C57BL/6 strain. - Hip1r-deficient mice: Mixed 129X1 and C57BL/6 strain background. | - Method: Genetic knockout of Hip1r (Huntingtin-interacting protein 1-related), which is critical for parietal cell function. - Principle:  1. Hip1r deficiency disrupts vesicular trafficking in parietal cells, leading to apoptotic loss of parietal cells. 2. Parietal cell loss initiates secondary remodeling:   - Zymogenic cell apoptosis.  - Expansion of metaplastic mucous cells (SPEM, spasmolytic polypeptide-expressing metaplasia).  - Glandular hypertrophy and hyperproliferation due to hypergastrinemia. | - Pre-weaned mice (newborn to 3 weeks): Freely fed. - Older mice (5–8 weeks): Fasted overnight with free access to water before euthanasia. - Housing: Ventilated cages, specific pathogen-free conditions, automated watering. | - Histology: H&E staining, Oil Red O (lipid detection). - Immunostaining:   - Parietal cells: H⁺-K⁺-ATPase α-subunit.  - Mucous cells: Muc5AC, GSII lectin, TFF2.  - Zymogenic cells: Intrinsic factor (IF).  - Endocrine cells: Chromogranin A (CgA).  - Proliferation: BrdU labeling.  - Apoptosis: Cleaved caspase-3 co-staining.   - Functional assays: Gastric acid content measurement (titration).   Gene expression: qRT-PCR for lineage-specific markers (e.g., H⁺-K⁺-ATPase, Muc5AC, IF, gastrin). | - Advantages:   - Recapitulates human gastric metaplasia (SPEM) and parietal cell atrophy.  - Progressive pathology allows study of sequential events (parietal cell loss → SPEM → hyperplasia).  - Genetic model avoids chemical/physical interventions.   - Disadvantages:   - Mixed genetic background (129X1/C57BL/6) may introduce variability.  - Slow progression (weeks to months) for full pathology.  - Limited to corpus-specific changes (no antral involvement). | Hip1r-deficient model:   - Newborn (P0):   - Rudimentary glands; no mature cell types.  - Parietal cell apoptosis begins (earliest defect).   - 1 week:   - Parietal cell dysfunction (reduced acid secretion).  - No other lineage abnormalities.   - 3 weeks:   - Gland morphogenesis completes.  - Parietal cell numbers decline.  - Zymogenic cell apoptosis begins.   - 5 weeks:   - SPEM emerges (TFF2^+^ metaplastic cells).  - Mucous cell hyperplasia (increased Muc5AC).  - Glandular hypertrophy/hyperproliferation (hypergastrinemia). | - Corpus (oxyntic mucosa): Parietal cell loss, SPEM, zymogenic cell apoptosis. - No antral involvement reported. | Not described in the study. The model focuses on pre-neoplastic stages (SPEM, hyperplasia) without progression to invasive cancer. | - No overt inflammation or immune cell infiltration reported. - Secondary changes (e.g., hypergastrinemia, SPEM) are driven by parietal cell loss, not inflammatory mediators. | - Study parietal cell biology and vesicular trafficking. - Investigate mechanisms of SPEM and gastric metaplasia. - Model early stages of gastric atrophy and pre-cancerous remodeling. - Test therapeutic interventions for metaplasia/hyperplasia. - Not suitable for studying antral pathology or Helicobacter-induced gastritis. - Requires aging mice to observe full pathology (5–8 weeks). |
| Ref. (76) | - Muc6 knockout (Muc6^−/−^) mice: Generated by knocking the dsRED-2A-FlpER-STOP sequence into exon1 of the Muc6 gene. - Muc6-dsRED mice: Express dsRED specifically in Muc6^+^ cells via an IRES-dsRED-2A-FlpER cassette inserted into the 3' untranslated region of Muc6. - Other strains used:   - Golph3^−/−^.  - Hes1^flox/flox^, R26-Golgi-mCherry.  - Tff1Cre, Cosmc^flox/flox^, A4gnt^−/−^. | - Method: Deletion of Muc6 disrupts O-linked glycosylation of gastric mucins, leading to:   - Loss of αGlcNAc-modified MUC6, which normally protects against H. pylori and maintains gastric homeostasis.  - Golgi stress due to impaired glycosylation, upregulating GOLPH3, which activates the MAPK pathway (EGFR/p-ERK).  - Spontaneous inflammation, metaplasia, dysplasia, and invasive adenocarcinoma.   - Principle: Muc6 deficiency mimics human gastric carcinogenesis (Correa sequence) by disrupting mucin barrier function and triggering oncogenic signaling. | - Standard laboratory diet; no specific dietary modifications mentioned. - For *H. pylori* acceleration experiments: Mice were orally infected with *H. pylori*. | - Histology: H&E staining for hyperplasia, dysplasia, and invasion. - Immunohistochemistry (IHC):   - Proliferation (Ki67), metaplasia markers (CD44v, TFF2), Golgi stress (GOLPH3, TFE3), MAPK activation (p-ERK).  - Mucin expression (MUC5AC, MUC4, GSII lectin for αGlcNAc).   - RNA-seq: Identified DEGs (e.g., upregulated Tff2, Cd44; downregulated Atp4a, Gif). - Glycomics: LC-MS and lectin arrays detected aberrant mannose-rich N-glycans. - Functional assays:   - Organoid cultures (proliferation, drug responses).  - Lectin-drug conjugates (rBanana-PE38) tested in xenografts. | - Advantages:   - Recapitulates human gastric cancer progression (inflammation → metaplasia → cancer).  - Spontaneous tumor development without carcinogens.  - Mechanistic insights into Golgi stress and glycosylation in carcinogenesis.   - Disadvantages:   - 100% penetrance of tumors may limit heterogeneity studies.  - Requires long-term maintenance (tumors develop by 12 months). | - Muc6^−/−^ mice:   - 3 months: Hyperplastic changes (antrum-predominant).  - 8 months: Dysplasia with expanded CD44v^+^/TFF2^+^ metaplasia.  - 12 months: Invasive adenocarcinoma (submucosal invasion).   - Accelerated by *H. pylori*: Larger tumors by 8 months. - Tff1Cre;Cosm^flox/flox^ and A4gnt^−/−^ mice: Similar stages but milder inflammation. | - Muc6^−/−^ mice:   - Antrum: Macroscopic tumors (100% incidence).  - Corpus: Atrophy, parietal/chief cell loss, but no macroscopic tumors.   - Tff1Cre;Cosmc^flox/flox^ and A4gnt^−/−^ mice: Antral-predominant dysplasia. | - Muc6^−/−^ mice: Develop submucosal invasive adenocarcinoma by 12 months. - No distant metastasis reported. | - Muc6^−/−^ mice:   - Early stage (3 months): F4/80^+^ macrophages dominate.  - Established tumors: Infiltration of CD3^+^/CD4^+^/CD8^+^ T cells and Foxp3^+^ Tregs.  - Corpus/antrum: Pan-gastritis with CD45^+^ leukocytes.  Tff1Cre;Cosmc^flox/flox^ and A4gnt^−/−^  mice: Less inflammation vs. Muc6^−/−^. | - Investigating the role of MUC6 in gastric cancer development. - Screening and testing new therapeutic targets and drugs for gastric cancer, such as lectin-drug conjugates. - Studying the mechanisms of gastric tumorigenesis and the effects of genetic mutations on cancer development. - Exploring the relationship between mucin deficiency, glycosylation impairment, and oncogenic signaling pathways. |
| Ref. (77) | - TFF1-knockout (TFF1-KO) mice (C57BL/6J/129/Svj background). - Control: TFF1-wild-type (TFF1-WT) mice (same background). | - Method: Genetic knockout of TFF1 (trefoil factor 1), a secreted protein critical for gastric mucosal integrity. - Principle:   - Loss of TFF1 disrupts mucosal barrier function, triggering chronic inflammation and sequential neoplastic progression.  - TFF1 silencing activates STAT3 and NF-κB pathways, driving tumorigenesis. | Not specifically mentioned in the article. | - Histopathology: H&E staining to classify lesions (hyperplasia, dysplasia, adenocarcinoma). - Immunohistochemistry (IHC): Nuclear p-STAT3 (Y705), TFF1 expression. - Molecular analysis:   - qRT-PCR for STAT3 target genes (e.g., Vegf, c-Myc, Birc5, Il17a).  - Western blot for phospho-STAT3, phospho-GP130, phospho-JAK2.  - Chromatin immunoprecipitation (ChIP) assay for STAT3 binding to target gene promoters.   - Functional assays:   - Luciferase reporter assays for STAT3 transcriptional activity.  - Proximity ligation assay (PLA) to detect protein interactions (e.g., IL6Rα-GP130).   - 3D organoid cultures: Derived from TFF1-KO gastric tissues to validate STAT3 activation *ex vivo*. | - Advantages:   - Recapitulates human gastric cancer progression (inflammation → dysplasia → adenocarcinoma).  - Clear molecular mechanism (TFF1 loss → STAT3/NF-κB activation).  - No need for exogenous carcinogens/infections (*H. pylori*).   - Disadvantages:   - Limited to TFF1-dependent pathways; may not mimic other genetic/environmental triggers.  - Time-consuming (needs ≥6 months for adenocarcinoma).  - No reported metastasis, limiting studies on advanced cancer biology. | - 2 months:   Nuclear STAT3 activation (pre-neoplastic).  No dysplasia.   - 6 months:   Low-grade dysplasia.   - 10 months:   High-grade dysplasia and invasive adenocarcinoma. | - TFF1-KO mice: Lesions localized to the antropyloric region of the stomach. - No involvement of gastric corpus/body reported. | Developed invasive adenocarcinoma, but metastasis was not reported. | - Pro-inflammatory phenotype with upregulated cytokines (IL6, IL17A, TNF-α). - Immune cells: Not explicitly identified in the study (e.g., macrophages, neutrophils). - STAT3 activation drives expression of pro-inflammatory genes (e.g., Il6, Il23, Ccl2). | - Investigating the role of TFF1 in suppressing IL6-mediated activation of STAT3 and its impact on gastric tumorigenesis. - Studying the mechanisms of TFF1 regulation of STAT3 target genes and pro-inflammatory signaling pathways. - Testing potential therapeutic interventions targeting the IL6-STAT3 axis in gastric cancer. - Exploring the role of TFF1 in maintaining gastric mucosal integrity and its anti-inflammatory functions. |
| Ref. (78) | - TFF1-knockout (TFF1-KO) mice (C57BL/6J/129/Svj background). - Control: TFF1-wild-type (TFF1-WT) mice (same background). | - Method: Genetic knockout of TFF1 (trefoil factor 1), a tumor suppressor gene. - Principle:   - Loss of TFF1 leads to chronic inflammation, NF-κB activation, and aberrant AURKA overexpression, driving gastric tumorigenesis.  - AURKA directly phosphorylates IκBα, promoting NF-κB nuclear translocation and pro-inflammatory signaling. | - Standard diet: No specific diet mentioned; implied conventional laboratory chow. - No exogenous carcinogens or infections (e.g., *H. pylori*) were used. | - Histopathology: H&E staining to classify lesions (gastritis, dysplasia, adenocarcinoma). - Immunohistochemistry (IHC): AURKA, NF-κB p65, Ki-67, cleaved caspase-3. - Molecular analysis:   - qRT-PCR for Tnf-a, AURKA, and NF-κB target genes (Bcl-xL, Mcl1, Xiap).  - Western blot for p-NF-κB p65 (S536), p-IκBα (S32), AURKA.   - Functional assays:   - Luciferase reporter for NF-κB activity.  - *In vitro* kinase assay to confirm AURKA-IκBα interaction.   - *In vivo* therapy: Treatment with AURKA inhibitor MLN8237 (8 days or 12 weeks). - Imaging: Micro-PET with [¹⁸F]FLT to monitor tumor regression. | - Advantages:   - Recapitulates human gastric cancer progression (inflammation → metaplasia → adenocarcinoma).  - Clear molecular link between TFF1 loss, AURKA, and NF-κB.  - Useful for testing AURKA inhibitors (e.g., MLN8237).  Disadvantages:  - Slow progression (requires 12–14 months for adenocarcinoma).  - No metastasis reported.  - Limited to TFF1-dependent pathways. | - 8 weeks: Gastritis (mild gland elongation). - 16 weeks: Low-grade dysplasia (50% of mice). - 12 months: High-grade dysplasia/adenocarcinoma (>50% of mice). | - TFF1-KO mice: Lesions primarily in the antrum. - No corpus/body involvement reported. | TFF1-KO mice: Develop invasive intramucosal/submucosal adenocarcinoma, but no metastasis reported. | - Chronic inflammation (gastritis) by 8 weeks. - Elevated pro-inflammatory cytokines (Tnf-a, Il-1a, Il-1b). - Immune cells: Not explicitly identified (e.g., macrophages, neutrophils). | - Investigating the role of AURKA in promoting inflammation and gastric tumorigenesis. - Studying the mechanisms of AURKA regulation of NF-kB activation and its impact on inflammation and cell survival. - Testing potential therapeutic interventions targeting AURKA in gastric cancer. - Exploring the role of AURKA in maintaining gastric mucosal integrity and its anti-inflammatory functions. |
| Ref. (79) | - Strain: C57BL/6J/129/SvJ mixed genetic background. - Model: Tff1-knockout mice (generated by targeted deletion of the Tff1 gene). | - Method: Genetic knockout of Tff1, a tumor suppressor gene encoding a trefoil factor peptide. - Principle: Loss of Tff1 disrupts mucosal integrity and activates NF-κB-mediated inflammation, driving progression from gastritis to adenocarcinoma. | - Standard diet: Autoclaved rodent chow and water. - Antibiotic treatment: Some mice were treated with ciprofloxacin (0.34 mg/mL) and metronidazole (0.68 mg/mL) in drinking water from weaning (2 weeks) to 12 weeks to exclude pathogen-driven inflammation. | - Histopathology: H&E staining to assess lesions (hyperplasia, dysplasia, adenocarcinoma). - Immunohistochemistry (IHC): Ki-67 (proliferation), TFF1, and p-NF-κB-p65 (Ser536) expression. - Gene expression: Microarray and qRT-PCR for inflammatory genes (e.g., Tnfα, Il1b, Cxcl1). - Inflammation scoring: Chronic/acute inflammation graded histologically. - Functional assays: NF-κB luciferase reporter, immunofluorescence (nuclear p65 localization), Western blot (IKK/IκB phosphorylation). | - Advantages:   - Recapitulates human gastric carcinogenesis (multistep progression).  - Spontaneous inflammation and tumorigenesis without chemical induction.  - Useful for studying NF-κB-driven inflammation and Tff1’s tumor suppressor role.   - Disadvantages:   - Mixed genetic background may introduce variability.  - Lesions are restricted to the antropyloric region, not the entire stomach. | - Hyperplasia:   - Time: 2 months.  - Features: Elongated pits occupying mucosal thickness.   - Low-grade dysplasia (LGD):   - Time: ~6 months.  - Features: Crypt branching, crowded nuclei with maintained polarity.   - High-grade dysplasia (HGD):   - Time: ≥8 months.  - Features: Nuclear hyperchromasia, loss of polarity, glandular irregularity.   - Invasive adenocarcinoma:   - Time: ≥8 months (5/5 mice).  - Features: Submucosal invasion through muscularis mucosa gaps. | - Exclusive to antropyloric region (pyloric antrum). - No lesions observed in corpus, esophagus, colon, liver, spleen, or thymus. | - Invasive adenocarcinoma: Present in Tff1-KO mice (≥8 months), with submucosal invasion. - Metastasis: Not mentioned in the article. | - Chronic inflammation: Progressive with age, scored higher than wild-type. - Immune cells: Inflammatory cell infiltration in submucosa and glandular epithelia (H&E staining). - Pro-inflammatory factors: Upregulated Tnfα, Il1b, Cxcl1, etc.. - Specific immune cells: Not explicitly identified (e.g., macrophages, neutrophils), but NF-κB activation suggests myeloid cell involvement. | - Study NF-κB-driven inflammation in gastric cancer. - Test anti-inflammatory drugs (e.g., celecoxib reduced dysplasia by 50%). - Investigate Tff1’s role in mucosal protection and tumor suppression. - Validate human gastric cancer biomarkers (e.g., TFF1 loss and NF-κB activation). |
| Ref. (80) | - Strain: C57BL/6-129/Ola mixed genetic background. - Model: Muc5ac-knockout mice (generated by deleting exons 21–31 of Muc5ac). | - Method: Genetic knockout of Muc5ac, a major gastric mucin gene. - Principle: Loss of Muc5ac disrupts the protective mucus layer, leading to spontaneous antro-pyloric hyperplasia/adenomas and attenuates *H. pylori*-induced corpus mucous metaplasia. | - Standard diet: Specific pathogen-free (SPF) conditions (free of *Helicobacter spp*., *Citrobacter rodentium*, etc.). - Infection protocol: Mice were orally gavaged with *H. pylori* Sydney strain (SS1) or sham doses (Brucella broth) at 7 weeks of age. | - Histopathology: H&E staining to score lesions (inflammation, hyperplasia, dysplasia, adenomas). - Immunohistochemistry (IHC): Staining for mucins (Muc5ac, Muc1, Muc2), trefoil factors (Tff1, Tff2), GSL II (mucous metaplasia), and Clusterin (SPEM marker). - qPCR: Quantification of *H. pylori* colonization (ureB gene) and cytokine mRNA levels (Tnfα, Il-17A, etc.). - Fluorescent lectin staining: GSL II to identify mucous neck cells. | - Advantages:   - Recapitulates spontaneous *H. pylori*-independent antro-pyloric lesions.  - Useful for studying mucin-mediated gastric protection and *H. pylori* pathogenesis.   - Disadvantages:   - Mixed genetic background may introduce variability.  - Limited progression to invasive carcinoma (adenomas only). | - Hyperplasia:   - Time: Observed by 16 weeks post-infection (wpi).  - Features: Epithelial proliferation in antropyloric region.   - Dysplasia/Adenomas:   - Time: 32 wpi (39 weeks of age).  - Features: Low-grade adenoma (8% incidence), high-grade adenoma (8% incidence).   - Neuroendocrine-like foci: Rare, within adenomas. | - Exclusive to antropyloric region (pyloric antrum). - No lesions in corpus, forestomach, or other organs. | - Adenomas: Present but no invasive carcinoma or metastasis reported. - Neuroendocrine differentiation: Focal in one adenoma. | - Chronic inflammation: Higher in Muc5ac-KO vs. WT. - Immune cells: Leukocyte infiltration in mucosa/submucosa (H&E). - Cytokines: Reduced Tnfα and Il-17A in Muc5ac-KO vs. WT. | - Study Muc5ac's role in gastric mucosal protection. - Investigate H. pylori colonization dynamics (higher in Muc5ac-KO at 16 wpi). - Explore SPEM (Clusterin-positive metaplasia) and mucous metaplasia (GSL II-positive). |
| Ref. (81) | - A4gnt KO mice: Spontaneously develop differentiated-type gastric adenocarcinoma. - Chst4 KO mice: Deficient in sulfotransferase GlcNAc6ST-2, no significant gastric pathology. - A4gnt/Chst4 DKO mice: Generated by crossing A4gnt KO and Chst4 KO mice; lack gastric sulfomucins but develop adenocarcinoma and gastritis cystica profunda. - WT mice: Normal gastric morphology. | - Method:   - A4gnt KO mice: Loss of α1,4-N-acetylglucosaminyltransferase (α4GnT) leads to spontaneous gastric adenocarcinoma.  - A4gnt/Chst4 DKO mice: Further deletion of Chst4 (GlcNAc6ST-2) removes gastric sulfomucins, exacerbating gastric erosion and inducing gastritis cystica profunda.   - Principle:   - A4gnt KO: Loss of αGlcNAc (tumor suppressor) promotes hyperplasia → dysplasia → adenocarcinoma.  - A4gnt/Chst4 DKO: Loss of sulfomucins disrupts mucosal integrity, leading to severe erosion → gastritis cystica profunda. | - Mice were maintained in autoclaved cages under specific pathogen-free conditions. - Specific feeding conditions were not detailed. | - Histopathology:   - H&E staining (hyperplasia, dysplasia, adenocarcinoma, gastritis cystica profunda).  - Mucin histochemistry: HID-AB staining (sulfomucins), AB-PAS staining (neutral/acidic mucins).   - Immunohistochemistry:   αGlcNAc (HIK1083 antibody), Ki-67 (proliferation), Ly-6G/Ly-6C (Gr-1, granulocytes).   - Mass spectrometry:   Oligosaccharide analysis of gastric mucins (sulfated vs. neutral O-glycans).   - qRT-PCR:   Inflammation-related genes (Cxcl1, Ccl2, Il11, Fgf7, etc.).   - Ki-67 labeling index:   Measures epithelial cell proliferation. | - Advantages:   - Spontaneous tumor development (no chemical induction).  - Recapitulates human gastric cancer progression (hyperplasia → adenocarcinoma).  - A4gnt/Chst4 DKO model uniquely develops gastritis cystica profunda, a rare benign lesion linked to cancer.   - Disadvantages:   - Low incidence of adenocarcinoma in DKO mice (50% at 60 weeks vs. 100% in A4gnt KO at 50 weeks).  - No metastasis observed (tumors limited to mucosa).  - Complex genetic manipulation (double knockout required). | - A4gnt KO:   - Hyperplasia: 5 weeks.  - Low-grade dysplasia: 10 weeks.  - High-grade dysplasia: 20–40 weeks.  - Adenocarcinoma: 50 weeks (100% incidence).   - A4gnt/Chst4 DKO:   - Hyperplasia: 5 weeks.  - Low-grade dysplasia: 10 weeks (50% incidence).  - High-grade dysplasia: 20–40 weeks.  - Adenocarcinoma: 50–60 weeks (50% incidence).  - Severe gastric erosion: 3 weeks (67% incidence).  - Gastritis cystica profunda: 10–60 weeks (100% at 60 weeks). | - A4gnt KO & A4gnt/Chst4 DKO:   - Pyloric mucosa (antrum) (tumors and gastritis cystica profunda).  - Fundic mucosa & duodenum: Normal morphology.   - Chst4 KO & WT: No pathological changes. | Adenocarcinoma in both A4gnt KO and DKO mice:  Limited to mucosa (no submucosal invasion or metastasis reported). | A4gnt/Chst4 DKO mice:   - Severe gastric erosion (3 weeks onwards) with granulocyte infiltration (Ly-6G/Ly-6C^+^ cells). - Upregulated chemokines: Cxcl1, Cxcl5, Ccl2 (neutrophil recruitment via CXCR2). - No macrophages mentioned in the study. | - A4gnt KO mice:   - Study gastric adenocarcinoma progression (hyperplasia → cancer).  - Investigate αGlcNAc’s tumor-suppressive role.   - A4gnt/Chst4 DKO mice:   - Study gastritis cystica profunda pathogenesis (rare benign lesion linked to cancer).  - Explore sulfomucins’ role in mucosal protection.   - General utility:   - Drug testing (anti-inflammatory or anti-tumor therapies).  - Mechanistic studies (inflammation-cancer transition). |
| Ref. (82) | - Gkn2 knockout (Gkn2^⁻/⁻^) mice: Generated to study the role of Gastrokine-2 (GKN2) in gastric inflammation and tumorigenesis. - gp130^F/F^ mice: Hyperactivate IL-11/STAT3 signaling, leading to spontaneous antral tumors. - Gkn2^⁻/⁻^ gp130^F/F^ compound mutants: Develop proximal gastric tumors (corpus) in addition to antral tumors. - Human GKN1/GKN2 BAC transgenic mice: Overexpress human GKNs to test tumor suppression. - Wild-type (WT) controls: Normal gastric morphology. | Method:  - Gkn2^⁻/⁻^ mice: Loss of GKN2, a secreted protein from gastric surface mucus cells (SMCs), disrupts mucosal homeostasis.  - Gkn2^⁻/⁻^ gp130^F/F^ mice: Combined GKN2 loss and STAT3 hyperactivation accelerates tumorigenesis.  *- H. pylori* infection: Gkn2^⁻/⁻^ mice infected with *H. pylori* Sydney Strain 1 (SS1) show exacerbated inflammation.   - Principle:   GKN2 suppresses Th1 immunity and maintains mucosal integrity. Its loss leads to:  - Chronic inflammation → atrophic gastritis → metaplasia → dysplasia → adenocarcinoma (intestinal-type GC progression).  - Impaired myeloid-derived suppressor cell (MDSC) activation, enhancing pro-inflammatory cytokine responses. | - Housing: Specific pathogen-free (SPF) conditions. - Diet: Standard laboratory chow (not detailed). - *H. pylori* infection: Mice inoculated with 10⁷ CFU H. pylori SS1 intragastrically. | - Histopathology:   - AB-PAS staining: Detects neutral (red) vs. acidic (blue) mucins (metaplasia marker).  - H&E staining: Assesses inflammation, atrophy, hyperplasia, dysplasia, and adenocarcinoma.   - Immunohistochemistry (IHC):   GKN2, MUC5AC, TFF1, Ki-67 (proliferation), Ly-6G/Ly-6C (granulocytes).   - Flow cytometry:   Immune cell profiling (macrophages, dendritic cells, MDSCs, Tregs).   - qRT-PCR:   Th1 markers (Ifng, Tbet), cytokines (IL-1β, IL-6, IL-11), MDSC markers (S100a8, S100a9).   - Luminex array:   Quantifies pro-inflammatory cytokines (TNF-α, IL-6, CXCL1). | - Advantages:   - Recapitulates human gastric cancer progression (inflammation → metaplasia → cancer).  - Gkn2^⁻/⁻^ gp130^F/F^ mice model proximal gastric tumors, rare in other models.  - Demonstrates GKN2’s anti-inflammatory role via MDSC regulation.   - Disadvantages:   - No spontaneous tumors in Gkn2^⁻/⁻^ alone (requires secondary triggers like *H. pylori* or gp130^F/F^).  - Limited metastasis (tumors remain localized).  - Complex genetic crosses needed for tumor phenotypes. | - Gkn2^⁻/⁻^ mice: 6–30 weeks; Mild corpus mucosal defects (hypertrophy, metaplasia). - Gkn2^⁻/⁻^ + *H. pylor*: 2 months post-infection (MPI); Severe atrophic gastritis, mucous metaplasia. - gp130^F/F^ mice : 12 weeks; Antral tumors (hyperplasia → adenocarcinoma). - Gkn2^⁻/⁻^ gp130^F/F^ mice: 12 weeks; Proximal (corpus) tumors + antral tumors. | - Gkn2^⁻/⁻^ mice:   - Corpus-predominant inflammation, metaplasia, and hypertrophy.  - Antrum largely unaffected.   - gp130^F/F^ mice:   Antral tumors (classic intestinal-type gastric cancer).   - Gkn2^⁻/⁻^ gp130^F/F^ mice:   Corpus tumors (novel phenotype) + antral tumors. | Adenocarcinomas in gp130^F/F^ and Gkn2^⁻/⁻^ gp130^F/F^ mice:  Localized to mucosa/submucosa (no invasion or metastasis reported). | - Gkn2^⁻/⁻^ mice:   - Th1-skewed immunity: Elevated IFN-γ, TNF-α, IL-6.  - Reduced MDSCs (myeloid-derived suppressor cells), leading to unchecked inflammation.  - Increased macrophages and dendritic cells (APCs).   - *H. pylori*-infected Gkn2^⁻/⁻^ mice:   - Granulocyte infiltration (Ly-6G/Ly-6C⁺ cells).  - No Th17 response (no change in IL-17). | - Gkn2^⁻/⁻^ mice: Study chronic inflammation-driven GC and GKN2’s anti-inflammatory role. - Gkn2^⁻/⁻^ gp130^F/F^ mice: Model proximal gastric tumors (rare in other models). - *H. pylori*-infected Gkn2^⁻/⁻^ mice: Investigate bacteria-induced premalignant progression. - Human GKN BAC transgenic mice: Test GKN2/GKN1 restoration as therapy for gastric cancer. |

**Table 9:** Summary of Signaling Pathway Dysregulation–Induced Animal Models for Gastric Precancerous Lesions

| Studies | Animal Model | Modeling Method and Principle | Feeding Conditions | Model Evaluation Methods | Advantages and Disadvantages | Detailed Time Course of Pathological Stages | Anatomical Sites of Lesions | Tumor Location and Invasion/Metastasis | Inflammation and Immune Cell Infiltration | Application Scenarios for Models |
| --- | --- | --- | --- | --- | --- | --- | --- | --- | --- | --- |
| Ref. (83) | - Primary Model: gp130^Y757F/Y757F^ mice (homozygous for the gp130 Y757F knock-in mutation) - Derivative Models:   - gp130^Y757F/Y757F^Il11ra1^−/−^ (lacking IL-11 receptor α-subunit).  - gp130^Y757F/Y757F^Il6^−/−^ (lacking IL-6).  - gp130^Y757F/Y757F^Stat3^+/−^ (heterozygous STAT3 deletion).   - - gp130^Y757F/Y757F^Stat1^−/−^ (STAT1 knockout). | - Method: Genetic knock-in of the Y757F mutation in the gp130 receptor subunit (shared by IL-6 family cytokines). - Mechanism:   - The mutation abolishes SOCS3 binding, disabling negative feedback regulation.  - Leads to hyperactivation of STAT3 and STAT1 signaling.  - IL-11 overexpression drives chronic inflammation and tumorigenesis.  - Loss of Shp2/Ras/ERK pathway activation contributes to tumor initiation. | - Housing: Specific pathogen-free (SPF) conditions. - Diet: not specified in detail. - Age at Analysis: Typically 10–20 weeks (tumors visible by 6 weeks). | - Macroscopic: Tumor mass measurement, stomach size, lesion counting. - Histopathology: H&E staining for hyperplasia, dysplasia, and inflammation. - Immunohistochemistry (IHC):   - pY-STAT3, PCNA, BrdU (proliferation).  - CD45 (inflammatory cells).   - Molecular Analysis:   - qRT-PCR for IL-11, STAT3/STAT1 target genes (Socs3, cyclin D1, survivin, Mmp13).  - Immunoblotting for phospho-STAT3/STAT1.   - Therapeutic Intervention:   STAT3 antisense oligonucleotides (ASOs) to reduce tumor burden. | - Advantages:   - Provides a genetically defined model for studying inflammation-associated gastric tumorigenesis.  - Allows for the study of the role of specific cytokines (e.g., IL-11) and signaling pathways (e.g., STAT3, STAT1) in gastric cancer development.  - Recapitulates many histological hallmarks of human gastric cancer.   - Disadvantages:   - The model involves a global mutation in gp130, which may affect multiple tissues and complicate the interpretation of results.  - The study of specific cytokines requires additional genetic modifications (e.g., IL-11 receptor knockout). | gp130^Y757F/Y757F^:   - Chronic Gastritis: By 6 weeks; Lymphoplasmacytic infiltrates in submucosa/lamina propria. - Hyperplasia: 6–10 weeks; Expansion of proliferating (PCNA^+^) epithelial cells. - Adenomatous Polyps: 10–14 weeks; Macroscopic tumors (>3 mm) in antropyloric region. - Dysplasia/Metaplasia: >14 weeks; Glandular distortion, metaplastic changes. | - Primary Site: Antrum (antropyloric region). - Affected Regions:   - gp130^Y757F/Y757F^: Tumors localized to the antrum (fundus unaffected).  - Inflammation: Present in submucosa & lamina propria. | - No spontaneous metastasis reported. - Local invasion: Limited to glandular disruption (no deep invasion or distant spread described). | - Inflammation Present: Yes (chronic gastritis). - Dominant Immune Cells:   Lymphoplasmacytic infiltrates (CD45^+^ cells).   - Key Cytokines: IL-11 (drives STAT3-dependent inflammation). - Models with Reduced Inflammation:   - gp130^Y757F/Y757F^Il11ra1^−/−^ (no inflammation/tumors).  - gp130^Y757F/Y757F^Stat1^−/−^ (reduced inflammation). | - Studying the molecular mechanisms underlying inflammation-associated gastric tumorigenesis. - Testing the efficacy of therapeutic agents targeting STAT3, STAT1, and IL-11 signaling pathways. - Investigating the role of specific genes and signaling pathways in gastric cancer development. |
| Ref. (84) | - Primary Model: gp130^Y757F/Y757F]^ (abbreviated as gp130^F/F^) mice with hyperactivated STAT3. - Derivative Models:   - gp130^F/F^Tlr2^−/−^ (TLR2 knockout).  - gp130^F/F^Tlr4^−/−^ (TLR4 knockout, used as control).  - Bone marrow chimeras:  gp130^F/F^ recipients reconstituted with gp130^F/F^Tlr2^−/−^  bone marrow. | - Method: Genetic knock-in of the Y757F mutation in gp130, disrupting SOCS3-mediated negative feedback. - Mechanism:   - STAT3 hyperactivation drives TLR2 upregulation in gastric epithelial cells.  - TLR2 promotes tumorigenesis via cell-autonomous proliferation/survival (not inflammation).  - Key Pathways: PI3K/Akt, ERK1/2, JNK MAPK, and NF-κB downstream of TLR2. | - Housing: Specific pathogen-free (SPF) conditions. - Diet: not detailed. - Age at Analysis:   Tumors visible by 6 weeks, analyzed up to 24 weeks. | - Macroscopic: Stomach size, tumor mass, lesion counting. - Histopathology: H&E staining for hyperplasia/dysplasia. - Immunohistochemistry (IHC):   TLR2, pY-STAT3, PCNA, TUNEL (apoptosis).   - Molecular Analysis:   - qRT-PCR for TLR2, STAT3 target genes (e.g., Socs3, Bcl2a1, Ccnd1).  - ChIP assays confirming STAT3 binding to the TLR2 promoter.   - Therapeutic Intervention:   TLR2-blocking antibody (OPN-301) reduced tumor burden. | - Advantages:   - Provides a genetically defined model for studying inflammation-associated gastric tumorigenesis. - Allows for the study of the role of specific cytokines (e.g., TLR2) and signaling pathways (e.g., STAT3) in gastric cancer development.  - Recapitulates many histological hallmarks of human gastric cancer.   - Disadvantages:   - The model involves a global mutation in gp130, which may affect multiple tissues and complicate the interpretation of results.  - The study of specific cytokines requires additional genetic modifications (e.g., TLR2 knockout). | gp130^F/F^：   - Hyperplasia: 4–6 weeks; Epithelial proliferation begins. - Tumor Initiation: 6–10 weeks;Macroscopic tumors in antrum. - Advanced Tumors: 10–24 weeks: Large (>4 mm) adenomatous polyps. | - Primary Site: Antrum (pyloric region). - Affected Regions:   - Tumors localized to the antrum (fundus unaffected).  - TLR2 expression highest in tumor epithelium. | - No spontaneous metastasis observed. - Local Invasion: Limited to glandular disruption (no deep invasion). | - Inflammation Present: Yes (chronic gastritis). - Dominant Immune Cells:   - Lymphoplasmacytic infiltrates (submucosa).  - No change in inflammation with TLR2 deletion (gp130^F/F^Tlr2^−/−^).   - Key Cytokines: IL-11 (drives STAT3), but TLR2 does not modulate inflammation. | - Study STAT3-TLR2 crosstalk in epithelial carcinogenesis. - Test TLR2 inhibitors (e.g., OPN-301 antibody). - Mimics intestinal-type gastric cancer with STAT3 hyperactivation. |
| Ref. (85) | - PPARD mice: Generated by pronuclear injection of a mouse Ppard expression construct under the control of a villin promoter (p12.4Kvill-Ppard) into fertilized FVB oocytes. Two independent founder lines (PPARD1 and PPARD2) were used, showing similar phenotypes. - Control mice: Wild-type (WT) littermates without the Ppard transgene. - Additional strains:   - LSL-tdTomato;villin-cre mice: For lineage-tracing experiments, generated by crossing LSL-tdTomato mice with villin-cre mice.  - Germ-free C57BL/6J mice: Used for *Helicobacter felis* infection experiments. | - Method: Overexpression of PPARD in villin-expressing gastric progenitor cells (VGPCs) via a villin promoter. - Principle:   - PPARD upregulates chemokines (CCL20, CXCL1), recruiting immune cells and creating a pro-inflammatory microenvironment.  - Chronic inflammation and immune cell-derived cytokines (e.g., IFNG) drive VGPC expansion, metaplasia, dysplasia, and eventual adenocarcinoma.  - A positive-feedback loop between PPARD and IFNG signaling sustains inflammation and transformation. | Not explicitly mentioned in the article. | - Histopathology: Hematoxylin and eosin (H&E) staining for hyperplasia, dysplasia, and adenocarcinoma. - Immunohistochemistry (IHC): For PPARD, villin, CD44, Ki67, E-cadherin, and metaplasia markers (e.g., TFF3, Muc2, GSII). - Lineage tracing: tdTomato fluorescence in VGPCs to track tumor origin. - Organoid culture: 3D cultures from gastric crypts to assess stemness and tumorigenicity. - Flow cytometry: Profiling of immune cells (CD45^+^, CD3^+^, CD11b^+^/Gr1^+^, etc.) in gastric tissues. - RNA sequencing: Transcriptome analysis of corpus mucosa. - Microbiome analysis: 16S rRNA sequencing to compare stomach microbiota between PPARD and WT mice. - Human tissue validation: PPARD expression in gastric cancer tissue microarrays. | - Advantages:   - Spontaneous development of invasive gastric adenocarcinoma without carcinogens or *H. pylori* infection.  - Recapitulates human GAC progression (metaplasia → dysplasia → adenocarcinoma).  - Identifies VGPCs as the cell of origin and highlights PPARD/IFNG crosstalk.   - Disadvantages:   - Limited to villin-expressing progenitor cells; may not model all gastric cancer subtypes.  - Mouse-specific differences in gastric biology compared to humans. | - 10 weeks: Normal mucosa. - 25 weeks:   - Hyperplasia of glandular epithelium.  - Low-grade dysplasia (LGD).   - 35 weeks:   - High-grade dysplasia (HGD).  - SPEM (spasmolytic polypeptide-expressing metaplasia) and intestinal metaplasia.   - 55 weeks:   - Invasive adenocarcinoma (37.5% of mice).  - Moderately/poorly differentiated foci. | - PPARD mice: Tumors initiated in the lesser curvature of the gastric corpus and expanded to the entire corpus. - Antrum involvement was minimal; VGPCs in the corpus showed greater stem-like properties. | - PPARD mice: Developed invasive adenocarcinomas penetrating gastric muscle layers. - Metastasis was not explicitly reported. | - PPARD mice: Severe chronic inflammation with infiltrating immune cells:   - T cells (CD3^+^): 23.8% of CD45+ cells.  - Granulocytes/neutrophils (CD11b^+^/Gr1^+^): 41%.  - Dendritic cells (CD11c^+^/F4/80^–^): 35.4% of myeloid cells.  - Macrophages (CD11c^–^/F4/80^+^): 6.47%.   - Cytokines: IFNG, IL-1α, IL-6, TNFα (from non-epithelial immune cells). | - Study PPARD-driven gastric carcinogenesis mechanisms. - Investigate VGPCs as cancer-initiating cells. - Explore PPARD/IFNG feedback loop in inflammation and tumorigenesis. - Test chemopreventive/therapeutic strategies targeting PPARD or VGPCs. - Model metaplasia-dysplasia-adenocarcinoma progression. - Not suitable for *H. pylori*-specific studies (microbiome was unchanged in PPARD mice). |
| Ref. (86) | - gp130757^F/F^ mice: Homozygous knock-in mice with mutations (Y757F/V760A) in the gp130 receptor, blocking SHP2 binding and enhancing STAT3 signaling. - Control: Wild-type (gp130WT/WT) and heterozygous (gp130757F/WT) littermates (129 Sv-J/C57BL/6 mixed background). | - Method: Genetic mutation of the gp130 receptor to disrupt SHP2/Erk/AP-1 signaling while hyperactivating STAT3. - Principle:   - Loss of SHP2-mediated signaling leads to unchecked STAT3 activation, driving inflammation and tumorigenesis.  - Mimics IL-6 family cytokine dysregulation (e.g., IL-11) without *H. pylori* infection. | - Standard diet: Conventional laboratory chow. - No *H. pylori* infection (confirmed pathogen-free facility). | - Macroscopic/microscopic analysis: Stomach area, adenoma size, and histopathology (H&E, Alcian blue/PAS). - Immunohistochemistry (IHC):   - Proliferation (PCNA), parietal cells (H^+^/K^+^ ATPase), ECL cells (chromogranin A), G cells (gastrin).  - Metaplasia markers (TFF2, intrinsic factor).   - Molecular analysis:   - qRT-PCR/Northern blot for TFF1, TFF2, Reg I, gastrin, EGFr, and ligands (TGF-α, HB-EGF).  - *In situ* hybridization for spatial gene expression.   - Functional assays: Gastric pH measurement, tissue gastrin RIA. | - Advantages:   - Rapid tumor development (100% penetrance by 6 weeks).  - Recapitulates human intestinal-type adenocarcinoma.  - No need for exogenous carcinogens/*H. pylori*.  - Clear STAT3/SHP2 signaling dichotomy.   - Disadvantages:   - Mixed genetic background (129 Sv-J/C57BL/6).  - Limited metastasis reported (submucosal invasion only). - Hypogastrinemia may not mimic human hypergastrinemic conditions (e.g., *H. pylori*). | - 4–6 weeks:   - Antral hyperplasia, gastritis, focal ulceration.  - Loss of TFF1/TFF2; upregulation of Reg I.   - 6–20 weeks:   - Adenomas (maximal growth by 20 weeks).  - Intestinal metaplasia, dysplasia.   - >30 weeks:   - Submucosal invasion (36% of mice).  - Fundic involvement, gastric atrophy. | - Primary site: Antrum (early stages). - Progression: Extends to fundus (lesser curvature) in advanced stages. | - Local invasion: Submucosal penetration observed after 30 weeks (with desmoplasia). - No distant metastasis reported. | - Inflammatory infiltrate: Mixed mononuclear cells and polymorphonuclear leukocytes. - Cytokines: Elevated IL-6/STAT3 signaling drives Reg I expression. - Immune cells: Not explicitly characterized (e.g., macrophages, T-cells). | - Mechanistic studies: IL-6/STAT3 vs. SHP2/Erk signaling in gastric tumorigenesis. - Therapeutic testing: STAT3 inhibitors or SHP2 activators. - Preclinical models: Intestinal-type adenocarcinoma (non-metastatic). |
| Ref. (87) | - Smad3^⁻/⁻^ mice: Generated on a B6;129/S background by mating Smad3^⁺/⁻^ heterozygous mice. - Controls: Age-matched Smad3^⁺/⁻^ (heterozygous) and wild-type (WT) littermates. - Housing: Maintained under specific pathogen-free (SPF) conditions in sterile microisolator cages with regular chow and water ad libitum. | - Method: Genetic knockout of Smad3, a downstream effector of TGF-β signaling. - Principle:   - Loss of Smad3 disrupts TGF-β-mediated tumor suppression.  - Tumors initiate at the forestomach/glandular transition zone due to unique properties of the first fundic gland (enriched with DCLK1⁺ tuft cells).  - Metaplasia (SPEM) progresses to dysplasia and invasive neoplasia via STAT3 activation. | - Standard diet: Regular mouse chow. - Environment: Temperature-controlled, 12-hour light/dark cycle. - No carcinogens or *Helicobacter* infection required (tumors develop spontaneously). | - Gross/histopathology: H&E staining of stomach sections. - Immunohistochemistry (IHC):   - Markers: TFF2/SP (SPEM), Ki-67 (proliferation), E-cadherin (adhesion), phosphoSTAT3 (oncogenic signaling), DCLK1 (tuft cells).   - Cell line studies: shRNA-mediated Smad3 knockdown in AGS/MKN28 gastric cancer cells. - Western blotting: Confirmed STAT3 activation in Smad3-deficient cells. - Quantitative analysis: DCLK1⁺ cell counts in tumors vs. normal mucosa. | - Advantages:   - Spontaneous tumorigenesis without carcinogens or pathogens.  - Recapitulates proximal gastric cancer (fundus), a clinically relevant subtype.  - Unique origin from the forestomach/glandular transition zone.   - Disadvantages:   - Limited to Smad3-driven mechanisms (may not model all gastric cancer subtypes).  - Background strain (B6;129/S) may influence phenotype. | - 4 months: No gross abnormalities. - 6 months:   - Metaplasia: Whitish exophytic growths at the forestomach/glandular junction (lesser curvature).  - SPEM: TFF2/SP⁺ metaplastic glands with branching bases.   - 10 months:   - Dysplasia: Nuclear pleomorphism, disorganization.  - Invasive neoplasia: Cystic glands penetrating submucosa/muscle layers. | Exclusive to the proximal stomach:   - Originates at the forestomach/glandular transition zone (lesser curvature). - Expands into the fundic mucosa but spares the antrum/greater curvature. | - Invasive:   - Cystic glands invade submucosa and muscle layers.  - Loss of E-cadherin membrane localization in invasive cells.   - Metastasis: Not reported. | - No overt inflammation (unlike *H. pylori*-associated models). - Immune markers:   - PhosphoSTAT3⁺ epithelial cells, indicating oncogenic signaling.  - No significant immune cell infiltration (e.g., macrophages, neutrophils) noted. | - Experimental Uses:   - Study proximal gastric cancer (fundus) pathogenesis.  - Investigate TGF-β/Smad3 signaling in tumor suppression.  - Explore DCLK1⁺ tuft cell roles in metaplasia-neoplasia progression.  - Test therapies targeting STAT3 or Smad3-related pathways.   - Limitations:   Not suitable for *H. pylori*- or inflammation-driven cancer studies. |
| Ref. (88) | Primary Strain:  Nfkb1^−/−^ (NF-κB1 knockout) on a C57BL/6 background.   - Crossed Strains:   - Il6^−/−^, Il22^−/−^, Il11Ra^−/−^, Tnf  ^−/−^, and Stat1^−/−^ mice.  - Compound mutants: Nfkb1^−/−^ Il6^−/−^, Nfkb1^−/−^ Il22^−/−^, Nfkb1 ^−/−^Il11Ra^−/−^, Nfkb1^−/−^Tnf^−/−^, and Nfkb1 ^−/−^Stat1^−/−^. | - Method: Genetic knockout of   Nfkb1 induces sterile inflammation-driven gastric tumorigenesis.   - Principle:   - Loss of NF-κB1 leads to aberrant STAT1 activation and increased cytokines (TNF, IL6, IL11, IL22).  - TNF drives STAT1 activation, promoting chronic gastritis, oxyntic atrophy, dysplasia, and invasive gastric cancer.  - IL11/IL11Rα signaling is critical for tumor invasion. | - Housing: Specific pathogen-free (SPF) facilities, free of *Helicobacter* species. - Diet: Not explicitly mentioned in the article. | - Histopathology: H&E staining to score gastritis, oxyntic atrophy, dysplasia, and invasion (0–3 scale). - Flow Cytometry: Analysis of immune cell infiltration (T cells, B cells, myeloid cells). - Western Blot: Quantification of STAT1/3 phosphorylation and total protein levels. - RNA Sequencing/qPCR: Cytokine (TNF, IL6, IL11, IL22) and gene expression (e.g., Socs3, Cyclin D1). - Immunohistochemistry: PD-L1 expression on epithelial and myeloid cells. | - Advantages:   - Recapitulates human gastric cancer stages (inflammation to invasion).  - STAT1 and TNF are mechanistically validated therapeutic targets.  - Useful for studying cytokine-driven tumorigenesis and immune checkpoint regulators (e.g., PD-L1).   - Disadvantages:   - Sterile inflammation (lacks *H. pylori* involvement, unlike human gastric cancer).  - Limited translational relevance due to mouse-specific pathways. | - Nfkb1^−/−^ Model:   - 3–6 months: Increased cytokine mRNA (TNF, IL6, IL11, IL22).  - 6 months: Chronic gastritis, oxyntic atrophy (parietal/chief cell loss).  - 12 months: Dysplasia (glandular disorganization).  - 18 months: Invasive gastric cancer (50% incidence).  - >20 months: Advanced invasion (muscularis penetration).   - Nfkb1^−/−^Stat1^−/−^:   No pathology even at 2 years.   - Nfkb1^−/−^Tnf^−/−^:   Delayed progression (reduced gastritis/atrophy at 6 months; minimal invasion at 18 months).   - Nfkb1^−/−^Il11Ra^−/^:   Reduced invasive gastric cancer at 12–18 months. | Nfkb1^−/−^ Mice:   - Body and Antrum: Both regions show gastritis, atrophy, dysplasia, and tumors. - Cytokine upregulation (e.g., IL6/IL11 in body; IL22 in antrum). | Nfkb1^−/−^ Mice:   - Invasive gastric cancer confirmed by histopathology (submucosal/muscularis mucosae penetration). - No metastasis reported. | - Nfkb1^−/−^ Mice:  1. Inflammation: Chronic gastritis with dense leukocyte infiltrates. 2. Immune Cells:   - Myeloid: CD11b^+^ cells (classical/resident monocytes, neutrophils).  - Lymphoid: CD4^+^/CD8^+^ T cells, B cells, Tregs.  - Epithelial/Myeloid PD-L1 upregulation (STAT1/TNF-dependent).   - Nfkb1^−/−^Tnf^−/−^: Reduced T/B cells and neutrophils. | - Study NF-κB/STAT1 crosstalk in inflammation-driven gastric cancer. - Test anti-TNF/IL11Rα therapies (e.g., monoclonal antibodies). - Evaluate immune checkpoint inhibitors (e.g., PD-L1 blockade). - Explore cytokine redundancy (IL6/IL11/IL22) in tumor progression. |
| Ref. (89) | - Primary Strain: Keratin 19 promoter-driven IL-11 transgenic mice (K19-IL11Tg) on a C57BL/6J background. - Control: Wild-type (WT) C57BL/6J littermates. - Backcrossing: Transgenic founders were backcrossed for ≥8 generations to ensure genetic uniformity. | - Method: Stomach-specific IL-11 overexpression via the K19 promoter, which targets gastric epithelial cells (surface mucus and isthmic progenitor zones). - Principle:   - IL-11 drives STAT3 hyperactivation, leading to chronic inflammation, atrophy, metaplasia, and hyperplasia.  - Independent of *Helicobacter pylori* infection or oncogenic gp130-JAK-STAT mutations.  - IL-11 overexpression mimics human gastric cancer progression linked to elevated IL-11 levels. | - Housing: Specific pathogen-free (SPF) facilities, free of *Helicobacter* species. - Diet: details not specified). | - Histopathology: H&E staining to score gastritis, atrophy, metaplasia, and hyperplasia (0–5 scale). - Immunohistochemistry: Ki-67 (proliferation), TFF2 (metaplasia), H^+^-K^+^-ATPase (parietal cell loss). - Flow Cytometry: Splenic immune cell profiling (myeloid/lymphoid subsets). - qPCR/Western Blot: IL-11, STAT3, and target genes (Socs3, Reg3b). - ELISA: Serum IL-11 levels. - Morphometry: Lesion area, mucosal thickness. | - Advantages:   - Recapitulates human premalignant stages (atrophy → metaplasia → hyperplasia).  - STAT3-driven pathology validates IL-11 as a therapeutic target.  - Useful for studying cytokine-driven tumorigenesis without *H. pylori*.   - Disadvantages:   - No invasive carcinoma or metastasis observed (limited to premalignant stages).  - Splenomegaly and systemic IL-11 effects may confound local gastric pathology. | - K19-IL11Tg Mice:   - 12 weeks: Mild atrophic gastritis (parietal/zymogenic cell loss).  - 36 weeks: Overt epithelial hyperplasia, mucus neck cell expansion.  - 52 weeks: Severe hyperplasia with adenoma-like lesions (20% of mice); no invasion.   - With *H. pylori* infection:   Accelerated metaplasia/hyperplasia at 2 months post-infection. | - Corpus: Primary site of pathology (hyperplasia, atrophy, metaplasia). - Antrum: Involved in older mice (52 weeks). | - Non-invasive: Hyperplastic lesions remain intramucosal (no submucosal invasion). - No metastasis reported. | - Inflammation: Chronic gastritis with dense leukocyte infiltrates. - Immune Cells:   - Myeloid: Splenic expansion of macrophages, neutrophils, MDSCs (CD11b^+^ subsets).  - Lymphoid: No significant changes in T/B cells in stomach; systemic IL-11 elevates serum cytokines.   - Key Mediators: STAT3 activation in epithelial cells drives proliferation (Ki-67^+^ nuclei). | - Study IL-11/STAT3 signaling in premalignant progression. - Test anti-IL-11 therapies (e.g., monoclonal antibodies). - Investigate synergy with *H. pylori* infection. - Models human gastric cancer with elevated IL-11 but no gp130 mutations. - Supports IL-11 inhibition as a therapeutic strategy for early gastric cancer. |
| Ref. (90) | - Wild-type (WT): C57BL/6 mice. - MyD88-deficient (Myd88^⁻/⁻^): C57BL/6 background. | - Method: Oral gavage with *H. felis* (strain CS1, ATCC 49179). - Procedure:   - Mice inoculated with 10⁹ organisms in 300 µL brain heart infusion (BHI) broth, 3 times at 2-day intervals.  - Control mice received BHI only.   - Principle:   *- H. felis* infection induces chronic inflammation, mimicking human *H. pylori*-associated gastric carcinogenesis.  - MyD88 deficiency accelerates progression to dysplasia by disrupting TLR/IL-1R signaling, leading to uncontrolled apoptosis, proliferation, and cytokine dysregulation. | - Housing: WT and Myd88^⁻/⁻^ mice co-housed pre- and post-infection. - Diet: not specified in the study. - Environment: Pathogen-free conditions. | - Histopathology:   H&E staining scored (0–4) for inflammation, atrophy, hyperplasia, metaplasia, and dysplasia by a blinded pathologist.   - Molecular Analysis:   Real-time PCR for *H. felis* colonization (flaB gene) and cytokine expression (TNF-α, IFN-γ, IL-6, IL-1β).   - Cell Dynamics:   - Apoptosis: TUNEL assay.  - Proliferation: Ki-67 immunohistochemistry.   - Morphometry:   Mucosal thickness and gland dilation measured microscopically. | - Advantages:   - Recapitulates human *H. pylori*-induced multistep carcinogenesis (chronic inflammation → dysplasia).  - MyD88 deletion clarifies its protective role in infection-driven cancer.   - Disadvantages:   *- H. felis* (not H. pylori) may limit direct translation to human pathology.  - No spontaneous metastasis observed; lesions remain localized. | - Uninfected Mice: No pathology (baseline). - Infected WT Mice:   - 25 weeks: Severe gastritis, atrophy, metaplasia.  - 47 weeks: Mild dysplasia (1/16 mice).   - Infected Myd88^⁻/⁻^ Mice:   - 25 weeks: Dysplasia (early carcinoma).  - 47 weeks: Moderate-to-severe dysplasia (all mice). | - Gastric corpus (body) – evidenced by gland atrophy (loss of oxyntic glands) and submucosal gland dilation. - Antrum inflammation was scored separately, but severe pathology was corpus-dominant. | - Observation: No reported metastasis. - Local Invasion:   Myd88^⁻/⁻^ mice showed gland herniation into submucosa (47 weeks), suggesting local invasiveness. | - Inflammatory Cells: Neutrophils, lymphocytes, macrophages (scored collectively as "chronic inflammation"). - Cytokines: Elevated TNF-α, IFN-γ, IL-6, IL-1β in Myd88^⁻/⁻^ mice (47 weeks). - Key Finding: Inflammation scores were similar in WT vs. Myd88^⁻/⁻^ mice, but cytokine levels diverged. | - Study MyD88-dependent pathways in infection-driven cancer. - Test therapeutics targeting TLR/IL-1R signaling or cytokine dysregulation. - Investigate epithelial apoptosis/proliferation balance in carcinogenesis. - Best for early-stage neoplasia (no metastatic model). - Requires validation for *H. pylori*-specific mechanisms. |
| Ref. (91) | - Wild-type (WT): Standard background (not specified, likely C57BL/6). - RNF43 Mutant (Rnf43ᴴ²⁹²ᴿ/ᴴ²⁹⁵ᴿ): CRISPR/Cas9-engineered mice with two point mutations (H292R/H295R) in the RING domain of Rnf43, leading to Wnt pathway transactivation. | - Method: Oral gavage with Helicobacter pylori strain PMSS1 (2 × 10⁸ CFU, twice). - Procedure:   - Mice infected at 6–8 weeks old, sacrificed after 6 months.  - Controls received brain-heart infusion (BHI) broth.   - Principle:   - Rnf43 mutations disrupt Wnt signaling regulation, causing mucosal hyperplasia.  - *H. pylori* infection exacerbates pathology via inflammation (Th1/Th17), NF-κB activation, and stem cell (CD44⁺) expansion. | - Housing: Standard pathogen-free conditions. - Diet: Not specified . | - Histopathology:   - H&E, PAS, and immunohistochemistry (IHC) for atrophy, metaplasia (MUC2⁺), hyperplasia, and reactive changes.  - Scored using the updated Sydney system (0–3 for atrophy, 0–3 for metaplasia/hyperplasia).   - Molecular Analysis:   - qPCR for cytokines (Cxcl1, Ifng, Il-17), Wnt/NF-κB targets (Cxcl10, Cxcl13).  - IHC for β-catenin (nuclear), p-STAT3, CD44, p65 (NF-κB).   - Proliferation: Ki67 staining. - Bacterial Load: Colony-forming units (CFU) in stomach homogenates. | - Advantages:   - Recapitulates human *H. pylori*-driven carcinogenesis (Correa pathway).  - Demonstrates synergy between genetic (Rnf43 mutation) and environmental (*H. pylori*) factors.   - Disadvantages:   - No neoplasia observed (limited to preneoplastic stages).  - *H. pylori* PMSS1 strain may not fully mirror human *H. pylori* virulence. | - Uninfected Rnf43ᴴ²⁹²ᴿ/ᴴ²⁹⁵ᴿ Mice:   Baseline: Mucosal thickening, hyperplasia (50% of mice), focal metaplasia (MUC2⁺).   - Infected Rnf43ᴴ²⁹²ᴿ/ᴴ²⁹⁵ᴿ Mice (6 months):   - Atrophy: Corpus-specific.  - Metaplasia: Multifocal → widespread (MUC2⁺).  - Hyperplasia: 75% of mice (vs. 17% in WT).  - Reactive Changes: Nuclear atypia in 100% of mutants (vs. 25% in WT). | - Exclusively gastric corpus (no pathology in antrum). - Evidence: Atrophy, metaplasia, and hyperplasia detected only in corpus. | - Observation: No neoplasia or metastasis reported. - Local Changes:   Hyperplastic glands with nuclear atypia (premalignant). | - Inflammatory Cells:   - Lymphocytes (CD3⁺): Increased infiltration in mutants (vs. WT).  - Neutrophils: No difference (chloracetate esterase staining).   - Cytokines:   - Th1 Response: Elevated Ifng (IFN-γ) in mutants.  - Th17 Response: High Il-17 (no WT vs. mutant difference).  - Innate Immunity: Cxcl1 (IL-8 homologue) upregulated in both WT and mutants. | - Study Wnt/NF-κB crosstalk in *H. pylori*-induced preneoplasia. - Test therapeutics targeting CD44⁺ stem cells or STAT3/NF-κB pathways. |
| Ref. (92) | - FVBN WT (Wild-Type) - Rnf43^ΔEx8^ mice: Generated by introducing a 57-bp deletion in exon 8 of the Rnf43 gene using CRISPR/Cas9, leading to loss of the functional RING domain. | - Method:   *H. Pylori* infection: Mice were infected twice with 2×10^8^ *H. pylori* strain PMSS1 via oral gavage.   - Principle:   *- H. pylori* induces DNA damage (e.g., double-strand breaks) and chronic inflammation, triggering aberrant DDR (DNA Damage Response).  - Loss of Rnf43 function impairs DDR, promoting resistance to apoptosis and cellular survival, which accelerates gastric carcinogenesis. | - Diet: Rodent diet (Envigo) ad libitum. - Housing: Specific pathogen-free (SPF) conditions in individually ventilated cages (Tecniplast) with enrichment material. | - Colonization: Quantified *H. pylori* CFU/mg stomach tissue. - Histopathology:   - Inflammatory scores (updated Sydney system) for activity/chronicity in antrum/corpus.  - Immunohistochemistry (IHC) for CD3^+^ T cells, γH2AX (DDR marker), cleaved caspase-3 (apoptosis), and β-catenin (WNT signaling).   - Cytokine Profiling: qPCR for Cxcl1, Ifng, and Tnfa. - DDR Analysis: Western blot for γH2AX and phosphorylated CHK2. | - Advantages:   - Recapitulates human *H. pylori*-associated gastritis and DDR dysfunction.  - Rnf43^ΔEx8^ mice show accelerated pathology without requiring additional carcinogens.   - Disadvantages:   - Limited to *H. pylori*-driven mechanisms; may not fully model sporadic gastric cancer.  - No spontaneous metastasis reported in this model. | Rnf43^ΔEx8^ Mice + *H. pylori* Infection:   - 3 months post-infection:   - Gastric hyperplasia, cellular atypia, and thickened mucosa (no tumors yet).  - Severe inflammation with increased CD3^+^ T cells, neutrophils, and mast cells.  - Reduced DDR (↓γH2AX) and apoptosis (↓cleaved caspase-3). | Rnf43^ΔEx8^ Mice: Pathology observed in both antrum and corpus (assessed via Sydney system scoring). | - Not reported: The study focuses on premalignant stages (hyperplasia, inflammation) and in vitro/xenograft tumor growth. - No data on invasion or metastasis in Rnf43^ΔEx8^ mice. | Rnf43^ΔEx8^ Mice + *H. pylori*:   - Inflammation: Severe gastritis with elevated Cxcl1, Ifng, and Tnfa. - Immune Cells:   - Increased CD3^+^ T cells.  - Neutrophils and mast cells (chloroacetate esterase-positive).  - No mention of macrophages. | - Study DDR impairment in gastric carcinogenesis. - Test chemoresistance mechanisms (e.g., 5-fluorouracil/cisplatin). - Investigate *H. pylori*-induced inflammation and early tumorigenesis. - Validate RNF43 as a biomarker for therapy response. |

**Table 10:** Summary of Autoimmune Gastritis Animal Model

| Studies | Animal Model | Modeling Method and Principle | Feeding Conditions | Model Evaluation Methods | Advantages and Disadvantages | Detailed Time Course of Pathological Stages | Anatomical Sites of Lesions | Tumor Location and Invasion/Metastasis | Inflammation and Immune Cell Infiltration | Application Scenarios for Models |
| --- | --- | --- | --- | --- | --- | --- | --- | --- | --- | --- |
| Ref. (93) | - BALB/c nu/+ (heterozygotes) and BALB/c nu/nu (nude mice). - Neonatal thymectomy (NTx) was performed on day 3 after birth. | - Method: Neonatal thymectomy (NTx) at 3 days postpartum to disrupt T-cell tolerance. - Principle:   - NTx depletes regulatory T cells (Tregs), leading to loss of self-tolerance.  - Autoreactive T cells attack gastric parietal cells, triggering autoimmune gastritis (AIG).  - Autoantibodies to parietal cells (PCA) and delayed-type hypersensitivity (DTH) contribute to tissue damage. | - Diet: Standard rodent diet (unspecified). - Housing: Pathogen-free conditions at 25°C in isolated air-conditioned boxes. | - Serology: Detection of parietal cell autoantibodies (PCA) by immunofluorescence. - Histopathology:   - H&E staining for gastric mucosal changes (e.g., parietal/chief cell loss, mononuclear infiltration).  - Immunohistochemistry for T cells (Thy1.2^+^, Lyt1^+^, Lyt2^+^) and B cells (IgM^+^).   - Functional Tests:   - Vitamin B12 absorption (impaired in AIG mice).  - Intrinsic factor (IF) levels in stomach extracts (reduced in AIG).   - Adoptive Transfer: Spleen cells from AIG mice transferred to nude mice induced gastritis. - Delayed-Type Hypersensitivity (DTH): Foot-pad swelling after parietal cell injection. | - Advantages:   - Recapitulates human pernicious anemia with autoantibodies and T-cell infiltration.  - No exogenous antigens required (unlike immunization models).   - Disadvantages:   - No anemia development (unlike human PA).  - Limited to T-cell-mediated mechanisms; B-cell role is secondary. | - 1–2 months post-NTx:   Early chief cell loss, mild mononuclear infiltration (T-cell dominant).   - 3–6 months post-NTx:   - Parietal cell destruction, compensatory mucous neck cell hyperplasia.  - B-cell infiltration and plasma cell clusters.  - Gastric mucosal hypertrophy (resembling Ménétrier’s disease macroscopically). | - Fundus to corpus (thickened mucosa with giant rugae). - Antrum spared (no significant pathology). | Not reported. The study focused on inflammatory gastritis, not neoplasia or metastasis. | - Early Stage (1.5–3 months):   T cells (Thy1.2+, Lyt1+, Lyt2+) dominate infiltrates.   - Late Stage (6+ months):   B cells (IgM^+^) and plasma cells appear.   - Inflammatory Factors:   - DTH reaction to parietal cells (mononuclear cell infiltration).  - No mention of macrophages or neutrophils. | - Study T-cell-mediated autoimmunity and loss of self-tolerance. - Model human pernicious anemia and gastric atrophy. - Test therapies targeting autoantibodies or T-cell subsets. |
| Ref. (93) | BALB/c (H-2^d^) mice, thymectomized 3 days after birth. | - Method:   - Neonatal thymectomy (NTx) at day 3 postpartum.  - Poly I:C administration: Intraperitoneal injections (5 mg/kg) twice weekly for 4 weeks (starting 5–6 days post-thymectomy).   - Principle:   - NTx depletes regulatory T cells (Tregs), breaking self-tolerance.  - Poly I:C (a viral RNA mimic) boosts proinflammatory cytokines (e.g., IFN-γ, IL-12, TNF-α) and further reduces Tregs, exacerbating autoimmunity.  - Targets parietal cells via autoantibodies to H^+^/K^+^ ATPase. | - Diet: Standard laboratory chow. - Housing:   - 22°C, 55% humidity, 12-h light/dark cycle.  - Pathogen-free conditions. | - Histopathology:   Gastritis staged (0–3) based on mononuclear infiltration, parietal cell loss, and mucosal hypertrophy.   - Autoantibody Detection:   Indirect immunofluorescence for anti-H^+^/K^+^ ATPase antibodies.   - Parietal Cell Quantification:   Immunofluorescence staining of gastric mucosa.   - Cytokine Profiling:   Serum levels of IL-6, IL-12p70, IFN-γ, TNF-α measured via cytometric bead array (CBA).   - Flow Cytometry:   CD4^+^CD2^5+^ Treg frequencies in splenocytes.   - Functional Assays:   T-cell suppression assays to validate Treg activity. | - Advantages:   - 100% induction rate of AIG (vs. 40–70% with NTx alone).  - Recapitulates all three features of human AIG: gastritis, autoantibodies, and parietal cell loss.  - Aggressive timeline (4 weeks vs. months in traditional models).   - Disadvantages:   - Poly I:C alone cannot induce AIG (requires NTx).  - No anemia observed (unlike human pernicious anemia). | - 4 Weeks Post-Thymectomy:   - Stage 1: Mononuclear cell infiltration (subglandular).  - Stage 2: Parietal cell loss + upward infiltration.  - Stage 3: Glandular atrophy + mucosal hypertrophy.   - Poly I-Critical Window: First 2 weeks post-thymectomy for maximal effect. | - Fundus and body of the stomach. - Antrum spared (no significant pathology). | This model focuses on autoimmune inflammation, not neoplasia. | - Infiltrating Cells:   - Mononuclear cells (lymphocytes, plasma cells).  - CD4^+^ T cells dominate early; B cells appear later.   - Key Cytokines:   IFN-γ, TNF-α, IL-12p70 (Th1-driven inflammation).   - Treg Suppression:   CD4^+^CD25^+^ Tregs significantly reduced in Poly I:C + NTx mice. | - Study Treg-cytokine interplay in autoimmunity. - Test therapies targeting H^+^/K^+^ ATPase autoantibodies or proinflammatory cytokines. - Optimize timing for preventive interventions (e.g., Treg reconstitution). |
| Ref. (94) | - Primary Strain Used: BALB/c mice (both wild-type and TSLPR-deficient strains). - Genetic Modification: TSLPR-deficient mice (TSLPR^−/−^) on a BALB/c background (backcrossed for 12 generations). - Model Subgroups:   - Neonatally thymectomized (NTx) BALB/c mice (NTx-TSLPR^+/+^ and NTx-TSLPR^−/−^).  - Non-thymectomized controls (non-NTx TSLPR^+/+^  and TSLPR^−/−^). | - Method: Neonatal thymectomy (NTx) performed 3 days after birth. - Principle:   - NTx disrupts thymic selection, reducing regulatory T cells (Tregs), which are critical for immune tolerance.  - This leads to uncontrolled autoreactive CD4^+^ T-cell responses against gastric parietal cells, mimicking human autoimmune gastritis (AIG).  - TSLPR deficiency exacerbates Th1-driven inflammation by enhancing IL-12/23p40 production by dendritic cells (DCs) and IFN-γ by CD4^+^ T cells. | - Housing: Specific pathogen-free (SPF) conditions. - Diet: not specified in detail. - Starvation Protocol: Mice were starved for 24 hours before sacrifice to standardize gastric pH measurements. | - Histopathology:  1. H&E staining to assess inflammation, atrophy, and hyperplasia. 2. Semi-quantitative scoring:   - Chronic inflammation (0–3: mononuclear cell infiltration).  - Atrophy (0–3: loss of parietal/chief cells).  - Hyperplasia (0–3: mucus neck cell proliferation).   - Immunohistochemistry:   - CD4^+^ T-cell infiltration (FITC-anti-CD4).  - TSLP expression (anti-TSLP staining).   - Serology:   ELISA for anti-parietal cell autoantibodies.   - Flow Cytometry:   Quantification of CD4^+^ T cells, IFN-γ^+^ cells, and IL-12/23p40^+^ DCs.   - qRT-PCR:   Th1/Th2/Th17 transcription factors (T-bet, GATA3, RORγt) and cytokines (IFN-γ, IL-4, IL-17A).   - Gastric pH: Measured in the corpus using pH test paper. | - Advantages:   - Recapitulates human AIG (autoantibodies, CD4^+^ T-cell infiltration, parietal cell loss).  - TSLPR^−/−^ mice show accelerated disease, useful for studying Th1-driven autoimmunity.  - Clear histopathological and molecular endpoints (e.g., IL-12/23p40, IFN-γ).   - Disadvantages:   - Neonatal thymectomy is technically challenging.  - Limited to pre-neoplastic stages (no spontaneous tumor development reported).  - BALB/c background may not fully mirror human genetic diversity. | - 6 Weeks:   - Early onset: Elevated anti-parietal cell antibodies.  - Grade 2+ chronic inflammation.   - 10–12 Weeks:   - Severe mononuclear infiltration (Grade 3).  - Complete loss of parietal/chief cells.  - Hyperplasia of mucus neck cells.  - Peak IFN-γ and IL-12/23p40 production.   - 14 Weeks:   Sustained high autoantibody titers. | Gastric Corpus: Exclusive involvement. | This model mimics pre-neoplastic AIG (chronic inflammation, atrophy) but does not develop tumors or metastasis. | - Dominant Immune Cells:   - CD4^+^ T cells (major infiltrate).  - Dendritic cells (DCs: CD11c^+^CD11b^+^) producing IL-12/23p40.   - Cytokines:   - Th1-skewed: High IFN-γ, IL-12/23p40.  - Low Th2/Th17 markers (GATA3, IL-4, IL-17A unchanged).   - No Mention of: Macrophages, neutrophils, or eosinophils. | - Study Th1-mediated autoimmunity in gastric mucosa. - Investigate TSLP/IL-12/IFN-γ axis in chronic inflammation. - Test therapeutics targeting immune checkpoints (e.g., PD-1) or cytokine pathways. - Model pre-neoplastic gastric changes (atrophy, metaplasia). |
| Ref. (95) | - Primary Strain Used: BALB/cGHC mice (gastritis-susceptible background). - Genetic Modifications:   - PC-GMCSF Transgenic Mice: Express granulocyte-macrophage colony-stimulating factor (GM-CSF) in gastric parietal cells under the H^+^/K^+^ ATPase β-subunit promoter.  - IFNAR2-Deficient Mice: Knockout of interferon-α receptor 2 (IFNAR2^−/−^), backcrossed onto BALB/cGHC for ≥6 generations.   - Model Subgroups:   - PC-GMCSF transgenic mice with genotypes: IFNAR2^+/+^  (wild-type), IFNAR2^+/−^ (heterozygous), IFNAR2^−/−^ (deficient). | - Method:   Transgenic expression of GM-CSF in gastric parietal cells drives local inflammation.  - IFNAR2 deficiency was introduced to test its modulatory role in autoimmune gastritis (EAG).   - Principle:   - GM-CSF promotes dendritic cell (DC) activation and Th1/Th17 responses, breaking tolerance to gastric H+/K+ ATPase (autoantigen).  - IFNAR2 deficiency was hypothesized to alter disease progression, but no effect was observed, indicating GM-CSF overexpression overrides IFNAR2-mediated regulation. | - Housing: Monash University Animal Facility (specific pathogen-free conditions). - Diet: details unspecified. - Sacrifice Protocol: Mice euthanized at 12 weeks for analysis. | - Serology:   - ELISA: Anti-parietal cell autoantibodies (targeting H^+^/K^+^ ATPase).  - Indirect Immunofluorescence (IIF): Autoantibody binding to gastric parietal cells.   - Histopathology:  1. H&E Staining: Mononuclear cell infiltration, parietal/zymogenic cell destruction. 2. Gastritis Scoring:   - Normal/Mild: Scattered submucosal mononuclear cells.  - Moderate: Submucosal infiltrate extending into mucosa.  - Severe: Parietal/zymogenic cell loss ± mucosal hypertrophy.   - PCR Genotyping: Confirmed IFNAR2^−/−^   and PC-GMCSF transgene status. | - Advantages:   - Recapitulates human autoimmune gastritis (autoantibodies, mononuclear infiltration, parietal cell destruction).  - GM-CSF-driven model avoids neonatal thymectomy (unlike other EAG models).  - Clear endpoints (histopathology, autoantibodies).   - Disadvantages:   - Limited to pre-neoplastic stages (no tumor development).  - IFNAR2 deficiency did not alter disease, reducing utility for studying IFN-α/β pathways.  - BALB/cGHC background may not fully mirror human diversity. | - 12 Weeks:  1. Autoantibodies: 86% of PC-GMCSF mice positive (regardless of IFNAR2 status). 2. Gastritis:   - Non-destructive: Mononuclear infiltration (82–92% incidence).  - Destructive: Parietal/zymogenic cell loss (37–41% incidence).   - No Earlier Time Points assessed in this study. | - Gastric Corpus: Exclusive involvement (targeting parietal/zymogenic cells). - Antrum: Not mentioned. | This model mimics autoimmune gastritis, not neoplasia. | - Dominant Immune Cells:   - Mononuclear infiltrate: Predominantly CD4^+^ T cells (implied by Th1-driven pathology).  - Dendritic Cells (DCs): Activated by GM-CSF (source of IL-12/23p40).   - Cytokines:   - Th1 Skew: High IFN-γ (not directly measured but inferred from EAG pathology).  - No Change with IFNAR2 KO: IL-12/23p40, IFN-γ levels unaffected.   - No Mention of: Macrophages, neutrophils, or eosinophils. | - Study GM-CSF-driven autoimmunity in gastric mucosa. - Investigate immune tolerance breakdown via local cytokine overexpression. - Test therapeutics targeting Th1 pathways or DC activation. |
| Ref. (96) | - Primary Strain: BALB/c background. - Genetic Modification:   - TA23 TCR Transgenic Mice: Express a T-cell receptor (TCR) specific for the H^+^/K^+^ ATPase proton pump (autoantigen in human AIG).  - Control: Non-transgenic BALB/c littermates. | - Method:   TA23 TCR transgenic CD4+ T cells target gastric parietal cells, inducing chronic inflammation.   - Principle:   - Autoimmune Gastritis (AIG): CD4^+^ T cells (Th1/Th17) infiltrate gastric mucosa, producing IFN-γ and IL-17, leading to parietal cell destruction.  - Cancer Progression: Chronic inflammation drives metaplasia (SPEM), dysplasia, and gastric intraepithelial neoplasia (GIN), mimicking the human Correa pathway. | - Housing: Specific pathogen-free (SPF) conditions. - *Helicobacter*-Free: PCR-confirmed absence of *Helicobacter* species. | - Histopathology:   - H&E Staining: Scored for inflammation (0–4), atrophy, metaplasia, and dysplasia.  - Immunofluorescence: Ki67 (proliferation), E-cadherin (epithelial cells), GIF (chief cells), GSII (mucous neck cells).   - Flow Cytometry:   CD4^+^ T cells (IFN-γ^+^, IL-17^+^), macrophages (CD11b^+^Ly6G^−^), neutrophils (CD11b^+^Ly6G^+^).   - Molecular Analysis:   - qRT-PCR: IL-6, IL-11, gastric cancer biomarkers (HE4, TFF2, OLFM4).  - Western Blot/Immunohistochemistry: pSTAT3 (activated STAT3).   - Serology: Autoantibodies to H^+^/K^+^ ATPase (ELISA). | - Advantages:   - Directly links chronic inflammation to gastric carcinogenesis (no *Helicobacter* or chemical induction).  - Recapitulates human Correa pathway (gastritis → atrophy → metaplasia → dysplasia → neoplasia).  - Useful for studying immune-driven tumorigenesis.   - Disadvantages:   - Slow progression (neoplasia at 12 months).  - Limited to intestinal-type gastric cancer (not diffuse-type). | - 2 Months:   - Chronic Gastritis: Mononuclear infiltration (CD4^+^ T cells, B cells).  - Oxyntic Atrophy: Partial parietal cell loss.   - 4 Months:   - Metaplasia: SPEM (TFF2^+^ mucous neck/chief cell hybrids).  - Mild Dysplasia: Focal irregular glands (20% of mice).   - 12 Months:   Severe Dysplasia/GIN: Pseudoinvasion into submucosa (87.5% of mice). | - Gastric Corpus: Exclusive involvement (parietal/chief cell region). - Antrum: Unaffected (normal morphology). | - Local Invasion:   Submucosal/serosal pseudoinvasion by dysplastic glands (no distant metastasis reported).   - Neoplasia:   High-grade dysplasia (GIN) but no frank adenocarcinoma observed. | - Immune Cells:   - CD4^+^ T Cells: Th1 (IFN-γ^+^) and Th17 (IL-17^+^) dominant.  - Macrophages: CD11b^+^Ly6G^−^ (minor subset).  - Neutrophils: CD11b^+^Ly6G^+^ (rare).   - Cytokines:   - Pro-inflammatory: High IFN-γ, IL-17, IL-6.  - Oncogenic Signaling: Elevated pSTAT3. | - Study immune-driven gastric carcinogenesis (independent of *Helicobacter*). - Test therapeutics targeting Th1/Th17 pathways or STAT3/IL-6 signaling. - Investigate SPEM as a precancerous lesion. |
| Ref. (97) | - Primary Model:   - GEC C1galt1^−/−^ mice: Gastric epithelial cell-specific knockout of C1galt1 (core 1 β1,3-galactosyltransferase), generated by crossing C1galt1^fl/fl^ mice with Foxa3-Cre transgenic mice.  - Control: Wild-type (WT) littermates (C1galt1^fl/fl^).   - Secondary Model:   TKO mice: GEC C1galt1^−/−^ Casp1/11^−/−^ (triple knockout), lacking both C1galt1 and inflammasome components caspase-1/11. | - Method: Genetic ablation of C1galt1 in gastric epithelium, leading to:   - Loss of core 1-derived O-glycans.  - Exposure of truncated Tn antigen (GalNAcα-O-Ser/Thr).  - Impaired mucin (Muc5AC) expression and mucus barrier dysfunction.   - Principle:   O-glycan truncation disrupts mucosal integrity → spontaneous gastritis → inflammasome activation (Casp1/11-dependent) → gastric adenocarcinoma. | - Diet: Standard chow. - Antibiotic Treatment: Some cohorts received broad-spectrum antibiotics (ampicillin, vancomycin, neomycin, metronidazole) to test microbiota dependence. - Omeprazole (OME) Treatment: Proton pump inhibitor to assess gastric acid’s role in inflammasome activation. | - Histopathology: H&E, PAS staining for mucins, immunohistochemistry (IHC) for Tn antigen, Muc5AC, Muc1, Casp1, Ki67 (proliferation), γH2AX (DNA damage). - Molecular Analysis:   - Western blot for Casp1 p10 (active form), iNOS.  - qPCR for cytokines (II1β, II18, II6, Tnfα, Cxcl1).  - ELISA for IL1β/IL18 in serum/organ cultures.   - Microbiome: qPCR/FISH for Helicobacter spp. and total bacteria. - Functional Assays:   - Gastric pH measurement.  - Organoid cultures from antrum epithelium. | - Advantages:   - Spontaneous progression from gastritis to adenocarcinoma (~80% penetrance by 1 year).  - Microbiota-independent (unlike *H. pylori* models).  - Clinically relevant (Tn/Casp1 correlate with human gastric cancer).   - Disadvantages:   - Disease limited to antrum (not corpus/forestomach).  - Slow progression (tumors appear at 6–12 months).  - Mixed genetic background (B6/129/NOD). | - GEC C1galt1^−/−^:  1. Gastritis:   - Onset: 2 weeks (mucosal thickening, immune infiltration).  - Established: 4–8 weeks (chronic inflammation, hyperplasia).   1. Dysplasia:   6 months (early neoplastic changes).   1. Adenocarcinoma:   12–18 months (60% incidence, invasive glands in submucosa).   - TKO Mice (Casp1/11^−/−^):   Delayed tumorigenesis (50% vs. 78% at 6–12 months; 67% vs. 95% at 12–18 months). | - GEC C1galt1^−/−^:   - Antrum-specific: Thickening, gastritis, tumors (no corpus/forestomach involvement).  - Corpus: Normal chief/parietal cell morphology.   - TKO Mice: Reduced antrum-specific pathology. | Adenocarcinoma in GEC C1galt1^−/−^:   - Invasive: Dysplastic glands penetrate submucosa. - No data on distant metastasis. | - GEC C1galt1^−/−^:  1. Inflammasome Activation: Casp1/11 → IL1β/IL18 secretion. 2. Immune Infiltrate:   - Granulocytes (MPO^+^ cells) dominant.  - Elevated II6, Tnfα, Cxcl1 (qPCR).   - TKO Mice: Reduced MPO^+^ cells and cytokines. | - Study O-glycosylation’s role in gastric barrier function. - Investigate inflammasome-driven gastritis/cancer (microbiota-independent). - Test therapeutics targeting Casp1/IL1β or mucin restoration. |
| Ref. (98) | - Primary Models:  1. Lymphopenic Models: BALB/c mice thymectomized neonatally (days 2–4 post-birth) or treated with cyclosporine/cyclophosphamide. 2. Non-lymphopenic Models: Mice immunized with gastric H^+^/K^+^ ATPase (α/β-subunits) or whole stomach homogenate + adjuvant. 3. Transgenic Models:   - PC-GMCSF mice: Express GM-CSF in parietal cells (BALB/cCrSic background).  - TCR Transgenic mice: Specific for H^+^/K^+^ ATPase α- or β-subunits.  - Spontaneous Model: C3H/He mice (30% incidence in conventional housing).   - Control: Wild-type (WT) littermates or untreated mice. | - Methods:   - Lymphopenia Induction: Neonatal thymectomy, irradiation, or cytotoxic drugs (e.g., cyclophosphamide) disrupt immune regulation → autoreactive CD4^+^ T-cell expansion.  - Immunization: H^+^/K^+^ ATPase or parietal cells + adjuvant (e.g., CFA) → break tolerance.  - Transgenesis: Overexpression of GM-CSF or autoreactive TCRs in gastric epithelium.   - Principle:   Loss of immune tolerance to gastric H^+^/K^+^ ATPase (major autoantigen) → CD4+ T-cell-mediated destruction of parietal/chief cells → gastritis → pernicious anemia (vitamin B12 deficiency). | - Standard Diet: Not specified, but mice housed in conventional or specific pathogen-free (SPF) facilities. - Environmental Influence: Higher EAG incidence in C3H/He mice in conventional vs. SPF conditions (suggesting microbial triggers). | - Histopathology: H&E staining for mononuclear infiltrates, parietal/chief cell loss, and mucosal hypertrophy. - Serology:   - Anti-parietal cell antibodies (immunofluorescence).  - Anti-H^+^/K^+^ ATPase antibodies (ELISA/immunoblot).   - Cellular Analysis:   - Flow cytometry for CD4^+^ T cells, macrophages, B cells.  - T-cell proliferation assays (response to H^+^/K^+^ ATPase).   - Functional Tests: Gastric pH measurement (achlorhydria). | - Advantages:   - Recapitulates human autoimmune gastritis (antibodies, T-cell responses, pathology).  - Multiple induction methods (genetic, immunologic, surgical).  - Useful for studying immune tolerance (e.g., CD4^+^CD25^+^ Tregs).   - Disadvantages:   - Variable disease penetrance (e.g., spontaneous C3H/He model: 30%).  - Immunization-induced EAG is transient (requires repeated boosts).  Limited metastasis data (focused on gastric pathology). | - Lymphopenic Models (e.g., neonatal thymectomy):   - Gastritis Onset: 4–8 weeks post-thymectomy.  - Established Disease: 12+ weeks (parietal cell loss, mucosal hypertrophy).   - Immunization Models:   Acute Gastritis: 2–4 weeks post-immunization (resolves after adjuvant cessation).   - Transgenic Models (PC-GMCSF):   Onset: 6–8 weeks (spontaneous gastritis).   - Spontaneous (C3H/He):   Variable: 3–6 months (environment-dependent). | - Body/Fundus-Specific: Targets parietal/chief cells (H^+^/K^+^ ATPase-rich). - Antrum: Typically spared. | - Not Reported: EAG models focus on inflammation and atrophy, not neoplasia. - Human Link: Chronic autoimmune gastritis predisposes to gastric cancer, but murine models do not progress to malignancy. | - Infiltrate Composition:   - CD4^+^ T cells (dominant, Th1-polarized; produce IFN-γ).  - Macrophages/Dendritic cells (early infiltrate, APC role).  - B cells (late-stage, antibody production).   - Cytokines: IFN-γ, IL-2, IL-6 (Th1); minimal IL-4. - Key Mediators:   - Fas/FasL-mediated apoptosis of parietal cells.  - Treg Deficiency: CD4^+^CD25^+^ T-cell depletion exacerbates disease. | - Study immune tolerance breakdown (Tregs, thymic selection). - Test therapies targeting CD4^+^ T cells or autoantibodies. - Investigate vitamin B12 deficiency mechanisms. |
| Ref. (99) | - Primary strain: SAMP1/YitFc (SAMP) mice. - Control strain: AKR mice (parental control). - Other strains mentioned:   - SAMPxIl33^−/−^ (IL33-deficient SAMP).  - C57BL/6J (B6) mice (for IL33 knockout backcrossing). | - Method:   - Spontaneous model: SAMP mice develop chronic gastritis progressing to intestinalized spasmolytic polypeptide-expressing metaplasia (SPEM) without chemical/genetic manipulation.  - Induced model: Exogenous recombinant IL33 (rIL33, 33 μg/kg, intraperitoneal, daily for 1 week) in AKR mice induces moderate metaplasia and M2 macrophage infiltration.   - Principle:   - IL33/ST2 axis drives eosinophil-dependent M2 macrophage recruitment, leading to metaplasia.  - Chronic inflammation triggers chief cell transdifferentiation into SPEM, a preneoplastic lesion. | - Mice were maintained under standard conditions. - Specific diet details are not mentioned in the article. | - Histopathology:   - H&E, Alcian blue/PAS staining for SPEM/intestinal metaplasia.  - Immunohistochemistry for IL33, CD163 (M2 macrophages), MBP (eosinophils), DBA (parietal cells).   - Molecular profiling:   - qPCR for markers like Gif, Atp4a, Tff2, Clu, Cftr (SPEM/intestinalized SPEM).  - Western blot for IL33 isoforms (full-length vs. cleaved).   - Flow cytometry:   Eosinophils (CD11b^+^ Siglec-F^+^) and M2 macrophages (CD163^+^ Arg1^+^) in bone marrow/gastric tissues.   - Functional studies:   - IL33 blockade (sST2-Fc fusion protein).  - Eosinophil depletion (anti-IL5/anti-CCR3 antibodies). | - Advantages:   - Spontaneous progression mimics human chronic gastritis-to-metaplasia sequence.  - No need for *H. pylori* infection or chemical induction.  - IL33/eosinophil axis is mechanistically defined.   - Disadvantages:   - Time-consuming (requires 20 weeks for advanced SPEM).  - Limited to preneoplastic stages (no adenocarcinoma reported). | - SAMP mice:   - 4 weeks: Early gastritis, eosinophil expansion (no metaplasia).  - 10–20 weeks: Progressive parietal cell loss, SPEM (Alcian blue/PAS^+^ mucous cells), and intestinalized SPEM (CD44v/Clu^+^).   - AKR + rIL33:   1 week: Induces mucous neck cell hyperplasia and oxyntic atrophy (no inflammation). | - SAMP mice: Gastric corpus (body of the stomach). - AKR + rIL33: Corpus-specific changes (no antrum/forestomach involvement mentioned). | Not observed: The study focuses on preneoplastic metaplasia (SPEM); no adenocarcinoma or metastasis reported. | - SAMP mice:   - Chronic inflammation: Dominated by eosinophils (MBP^+^) and M2 macrophages (CD163^+^ IL33^+^).  - IL33 from epithelial cells/M2 macrophages drives Th2 polarization.   - AKR + rIL33: Minimal inflammation but induces M2 macrophage infiltration. | - Study IL33/ST2 axis in chronic gastritis and metaplasia. - Test therapeutics targeting eosinophils/M2 macrophages to halt preneoplastic progression. - Investigate chief cell transdifferentiation mechanisms. - Evaluate drugs to reverse intestinalized SPEM. - Model environmental vs. immune-driven gastric carcinogenesis. |

**Table 11:** Summary of Multigenic Mutation or Deletion Models in Specific Glandular Cells for Gastric Precancerous Lesions

| Studies | Animal Model | Modeling Method and Principle | Feeding Conditions | Model Evaluation Methods | Advantages and Disadvantages | Detailed Time Course of Pathological Stages | Anatomical Sites of Lesions | Tumor Location and Invasion/Metastasis | Inflammation and Immune Cell Infiltration | Application Scenarios for Models |
| --- | --- | --- | --- | --- | --- | --- | --- | --- | --- | --- |
| Ref. (100) | - Primary strain: Anxa10-CreERT2 mice (stomach-specific inducible Cre line). - Other strains used for crossbreeding:   - CIN model: Kras^G12D/+^; Tp53^R172H/+^; Smad4^fl/fl^.  - GS-TGFβ model: Cdh1^fl/fl^; Kras^G12D/+^; Smad4^fl/fl^.  - GS-Wnt model: Cdh1^fl/fl^; Kras^G12D/+^; Apc^fl/fl^.   - Control strain: C57BL/6 (background strain for genetic modifications). | - Method:  1. Inducible Cre/loxP system: Tamoxifen-induced CreERT2 activation in Anxa10 locus drives recombination of floxed alleles (e.g., Kras^G12D^, Tp53^R172H^, Smad4^fl^, Cdh1^fl^, Apc^fl^). 2. Subtype-specific genetic alterations:   - CIN subtype: TP53 + RTK-RAS + TGF-β pathways.  - GS-TGFβ subtype: Cell motility (CDH1 loss) + RTK-RAS + TGF-β pathways.  - GS-Wnt subtype: Cell motility (CDH1 loss) + RTK-RAS + WNT pathways.   - Principle:   - Mimics human gastric cancer subtypes (TCGA classification) by targeting dominant pathways.  - Tumors develop spontaneously in the gastric corpus post-tamoxifen induction. | - Mice were fed normal chow and water ad libitum. - No specific dietary modifications or H. pylori infection was used. | - Histopathology:   - H&E, PAS staining for tumor morphology (e.g., glandular, diffuse, serrated).  - Immunohistochemistry (IHC) for lineage markers (Ki67, PGC, VEGFβ, CK20, ANXA10).   - Molecular profiling:   - qPCR for pathway markers (e.g., Ki67, Vegfb).  - Western blot for protein validation (e.g., ERK1/2, RAS G12D).   - Functional assays:   - Organoid cultures: Derived from tumors to test drug responses (e.g., 5-FU, docetaxel, trametinib).  - Metastasis assessment: Lung, liver, and peritoneal dissemination via histology. | - Advantages:   - Subtype-specific: Recapitulates intestinal (CIN), diffuse (GS-TGFβ), and serrated adenomatous (GS-Wnt) gastric cancers.  - Metastasis models: Liver/lung (CIN) vs. peritoneal (GS-TGFβ) metastases.  - Organoid utility: Enables drug screening and mechanistic studies.   - Disadvantages:   - Tamoxifen side effects: Parietal cell depletion may confound early metaplasia studies.  - Patchy recombination: Anxa10-CreERT2 activity is not uniform across all gastric cells. | - CIN model:   - 3 weeks: Dysplasia.  - 2–8 weeks: T1/T2 invasive cancer (submucosa/muscularis propria).  - 8–12 weeks: T3/T4 advanced cancer (subserosa).  - 10+ weeks: Liver/lung metastases.   - GS-TGFβ model:   - 1 week: T1 lesions.  - 8 weeks: T2 poorly differentiated cancer.  - 16+ weeks: Peritoneal carcinomatosis/lung metastases.   - GS-Wnt model:   - 4 weeks: Dysplasia.  - 4+ weeks: T1a/T1b adenomatous cancer (luminal obstruction). | - All models: Primarily in the gastric corpus (body of the stomach). - Antrum involvement: Minimal/none reported. | - CIN model:   - Invasive: Yes (subserosal invasion).  - Metastasis: Liver and lung (hematogenous spread).   - GS-TGFβ model:   - Invasive: Yes (diffuse infiltration).  - Metastasis: Peritoneal carcinomatosis and lung.   - GS-Wnt model:   - Invasive: No (luminal growth only).  - Metastasis: None observed. | - CIN model:   - Inflammation: Not prominently described.  - Immune cells: EMT markers (vimentin↑, laminin↓) in metastatic tumors.   - GS-TGFβ model:   - Inflammation: Associated with diffuse invasion.  - Immune cells: Signet ring cells (loss of CDH1).   - GS-Wnt model:   - Inflammation: Minimal.  - Immune cells: Not highlighted. | - Study subtype-specific tumorigenesis (e.g., EMT in metastasis). - Test targeted therapies (e.g., MEK inhibitors for GS-TGFβ). - Drug screening using tumor-derived organoids. - Validate biomarkers for metastasis (e.g., CK20, ANXA10). |
| Ref. (101) | - Primary strain:   Pgc-CreERT2: A knock-in mouse model with mCherry-IRES-CreERT2 inserted into the pepsinogen C (Pgc) locus.   - Crossed strains:   - Rosa-EYFP: For lineage tracing.  - LSL-KrasG12D/+: Conditional oncogenic Kras activation.  - Apc^flox/flox^: Conditional Apc inactivation.  - Trp53^flox/flox^: Conditional Trp53 inactivation.   - Combinations:   - Pgc-CreERT2;Kras^G12D/+^  - Pgc-CreERT2;Apc^flox/flox^  - Pgc-CreERT2;Kras^G12D/+^;Apc^flox/flox^  - Pgc-CreERT2;Kras^G12D/+^;Apc^flox/flox^;Trp53^flox/flox^ | - Method:  1. Stepwise oncogenic activation:   - KrasG12D/+ activation: Induces pseudopyloric metaplasia.  - Apc inactivation: Combined with KrasG12D/+, leads to intramucosal dysplasia/carcinoma (IDC).  - Trp53 inactivation: With KrasG12D/+ and Apc loss, drives invasive/metastatic carcinoma.   1. Induction: Tamoxifen injection (6 doses of 2 mg on alternate days) in 6–8-week-old mice.  - Principle:   Targets PGC-expressing cells (chief cells, isthmal progenitors) to mimic human gastric carcinogenesis stages. | - Standard laboratory diet (not specified in detail). - No dietary modifications or chemical carcinogens used. | - Histology: H&E staining for metaplasia, dysplasia, and carcinoma. - Lineage tracing: YFP/mCherry labeling to track cell origins. - Immunofluorescence (IF): Staining for markers (e.g., Ki67, CD44v10, β-catenin, YAP). - RNA in situ hybridization (ISH): Detects gene expression (e.g., Pgc, Lgr5). - RNA sequencing: Identifies differentially expressed genes (DEGs) and enriched pathways (e.g., Wnt, Hippo). - Gene Set Enrichment Analysis (GSEA): Evaluates signaling pathways (e.g., TNFα/NF-κB, IL6/JAK/STAT3). | - Advantages:   - Recapitulates multistage gastric carcinogenesis (metaplasia → IDC → metastasis).  - Stomach-specific oncogene expression (avoids extra-gastric tumors).  - Tunable via stepwise genetic modifications.   - Disadvantages:   - Pgc-CreERT2 targets multiple cell types (not chief-cell-specific).  - Low metastatic frequency (6.9% in Pgc-CreERT2;KrasG12D/+;Apc^flox/flox^;Trp53^flox/flox^ mice).  - Tamoxifen may have off-target effects if dosed improperly. | - Pseudopyloric Metaplasia:   - Model: Pgc-CreERT2;KrasG12D/+.  - Onset: 6 weeks post-tamoxifen.  - Peak: 3 months (5.9% glands affected).   - Intramucosal Dysplasia/Carcinoma (IDC):   - Model: Pgc-CreERT2;KrasG12D/+;Apc^flox/flox^.  - Onset: 9 months post-tamoxifen.   - Invasive/Metastatic Carcinoma:   - Model: Pgc-CreERT2;KrasG12D/+;Apc^flox/flox^;Trp53^flox/flox^.  - Onset: 9 months (20.7% submucosal invasion; 6.9% metastasis). | - Corpus (gastric body): - All models (Pgc-CreERT2;KrasG12D/+, Pgc-CreERT2;KrasG12D/+;Apc^flox/flox^, etc.). - Antrum: No lesions reported. | - Invasive:   Pgc-CreERT2;KrasG12D/+;Apc^flox/flox^;Trp53^flox/flox^ mice (penetrates muscle layers).   - Metastatic:   Lymph nodes, diaphragm, liver (same model). | - Inflammation: Present in metaplastic and tumor lesions. - Immune cells:   F4/80+ macrophages: Infiltrate stroma in:  - Pgc-CreERT2;KrasG12D/+ (metaplasia).  - Pgc-CreERT2;KrasG12D/+;Apc^flox/flox^ (IDC).  - Pgc-CreERT2;KrasG12D/+;Apc^flox/flox^;Trp53^flox/flox^ (invasive tumors).   - Cytokines: Enriched TNFα/NF-κB and IL6/JAK/STAT3 pathways in IDC. | - Study gastric stem cell biology, metaplasia origins, and carcinogenesis pathways (e.g., Wnt, Ras, p53). - Test therapeutics targeting specific stages (e.g., metaplasia prevention, metastasis inhibition). - Investigate roles of YAP, RUNX3, and CD44v10 in tumor progression. |
| Ref. (102) | - Primary strain:  1. Cldn18-IRES-CreERT2: Generated by homologous recombination, targets the Cldn18 locus to drive CreERT2 expression specifically in gastric epithelium. 2. Additional strains:   - Rosa26-tdTomato (Ai14) for lineage tracing.  - LSL-KrasG12D, Apc-loxP, Trp53-loxP.  - Lgr5-DTR-GFP (for Lgr5^+^ cell ablation).   - Background: C57BL/6J. | - Method:  1. Genetic perturbations: Compound mutations targeting Wnt (Apc deletion), RTK (KrasG12D activation), and Trp53 pathways via Cldn18-CreERT2. 2. Key combinations:   - Apc^fl/fl^ + KrasG12D + Trp53^fl/fl^ (Cldn18-ATK model) → Advanced metastatic GC.  - Apc^fl/+^ + KrasG12D + Trp53^fl/fl^ → Delayed tumorigenesis with spontaneous Apc LOH.   1. Orthotopic transplantation (OT): Cancer organoids derived from Cldn18-ATK mice injected into NSG mouse stomach submucosa.  - Principle: Recapitulates human GC progression from metaplasia (SPEM) to adenocarcinoma via dysregulation of pathways frequently mutated in human GC (TCGA data). | - Diet: not specified in detail. - Housing: Specific-pathogen-free (SPF), 12-h light-dark cycle, 21 ± 1°C, 55–70% humidity, individually ventilated cages. | - Histopathology: H&E staining for tumor staging (T1–T4), invasion, and metastasis. - IHC/IF: Markers for:   - Metaplasia: TFF2 (SPEM), CDX2 (IM).  - Proliferation: Ki67.  - EMT: E-cadherin loss, vimentin gain.  - Pathway activation: β-catenin (Wnt), phospho-MAPK (Ras).   - Lineage tracing: tdTomato reporter. - Transcriptomics: RNA-seq comparison with TCGA human GC data. - Functional assays: Lgr5-DTR-GFP ablation to assess cancer stem cell (CSC) role. | - Advantages:   - High reproducibility of metastatic GC.  - Accurately mimics human GC molecular/ histological features.  - Enables study of Lgr5^+^ CSCs in tumor initiation/maintenance.   - Disadvantages:   - Cre activity in lungs (low efficiency).  - Rapid lethality in Apc^fl/fl^ models due to aggressive primary tumors. | - 1 week: Antralization (SPEM; TFF2^+^, Muc5ac^+^). - 1 month: Focal neoplasia (early adenoma). - 2 months: T1 carcinoma (invasive glands). - 3 months: T3 carcinoma (serosa invasion). - Terminal (4–6 months): T3/T4 with metastases (liver, lymph nodes, lung). - Other models:   - Apc^fl/fl^ + KrasG12D: Tumors by 1 month (no metastasis).  - Trp53^fl/fl^ + KrasG12D: Corpus adenocarcinomas with metastases by 6 months. | - Corpus-dominant:   - Cldn18-ATK: 91% invasive tumors in corpus; 93% metastasize.  - Trp53^fl/fl^ + KrasG12D: Corpus-specific tumors.   - Pylorus involvement:   - Apc^fl/fl^ alone: Pyloric tumors (low metastasis).  - Apc^fl/fl^ + KrasG12D: Whole stomach tumors. | - Metastatic models:   - Cldn18-ATK: Invades serosa, metastasizes to liver, lymph nodes, lung, pancreas (93% rate).  - Trp53^fl/fl^ + KrasG12D: Liver/pancreas metastases.   - Non-metastatic: Apc^fl/fl^ or KrasG12D alone (local invasion only). | - Immune cells:   - Macrophages: Increased F4/80^+^ tumor-associated macrophages (TAMs) in advanced tumors (Cldn18-ATK).  - Polymorphonuclear cells: No significant change across stages (H&E quantification).   - Inflammation: Chronic gastritis-like features in early stages (SPEM). | - Study GC initiation, CSC dynamics (Lgr5^+^ ablation). - Pathway analysis (Wnt/Ras/p53 crosstalk). - Therapeutic testing (e.g., 5FU + Lgr5^+^ ablation synergy). - Metastasis mechanisms (OT/splenic injection models). |
| Ref. (103) | - Lgr5-eGFP-IRES-CreERT2 knock-in mice (for targeting Lgr5^+^ stem cells). - Rosa26-loxP-stop-loxP-tdTomato (Rosa26Tomato) reporter mice (for lineage tracing). - Floxed alleles:   - Smad4^fl/fl^ (for TGF-β signaling disruption).  - PTEN^fl/fl^ (for PI3K/Akt pathway activation).   - Other strains:   - Capn8-Cre (pit cell-specific deletion).  - Atp4b-Cre (parietal cell-specific deletion).  - Lgr5-Cre;Smad4^fl/fl^;p53^fl/fl^ and Lgr5-Cre;Smad4fl/fl;KrasG12D (for combinatorial mutations). | - Method:   - Inducible Cre-LoxP system: Tamoxifen (6 consecutive days) to delete Smad4 and PTEN in Lgr5^+^ stem cells.  - Lineage tracing with Rosa26Tomato to track mutant cells and progeny.   - Principle:   - Smad4 loss disrupts TGF-β tumor suppression.  - PTEN loss hyperactivates PI3K/Akt, promoting proliferation.  - Key finding: Lgr5^+^ stem cells with dual deletion initiate invasive intestinal-type gastric cancer (IGC). | - Tamoxifen administration:   - 2 mg/day intraperitoneally for 6 days in 6-week-old mice.  - Vehicle: Ethanol/corn oil mixture.   - BrdU injection:   100 mg/kg body weight (PBS, 10 mg/ml) 2 h before sacrifice for proliferation assays. | - Histopathology: H&E staining for tumor progression. - Immunofluorescence (IF):   - GFP (Lgr5^+^ cells), RFP/tdTomato (mutant lineage), Ki67 (proliferation).  - Differentiated cell markers (Pepsinogen, DBA, UEA, Gastrin).   - IHC/Western blot:   Smad4, PTEN, p-Akt, cyclin D1, p-Smad2, p-Stat3, etc.   - TCGA database analysis:   Human IGC correlation (SMAD4/PTEN deletion, LGR5 expression). | - Advantages:   - Rapid tumorigenesis (microadenoma in 45 days, invasion by 90 days).  - Recapitulates human IGC molecular features (SMAD4/PTEN loss, LGR5^+^ expansion).  - Lineage tracing validates stem cell origin.   - Disadvantages:   - Low penetrance at gastro-forestomach junction (25% vs. 65% in antrum).  - Tamoxifen dosing may require optimization (reported low Cre activity in Lgr5-CreERT2). | - Lgr5-Cre;Smad4^fl/fl^;PTEN^fl/fl^ model:   - Day 30: Single adenomatous gland (p-Akt^+^).  - Day 45: Microadenomas (<2 mm, 60% mice).  - Day 60: Widespread microadenomas.  - Day 90: Macroscopic adenomas (>2 mm, 65% mice); invasive IGC (40% mice).   - Other models:   - Smad4^fl/fl^ alone: Polypoid adenoma at 12 months (50% mice).  - PTEN^fl/fl^ alone: Microadenoma only at 12 months (63% mice). | - Lgr5-Cre;Smad4^fl/fl^;PTEN^fl/fl^:   - Primary: Gastric antrum (65% penetrance).  - Secondary: Gastro-forestomach junction (25% penetrance).   - Differentiated cell models (Capn8-Cre/Atp4b-Cre): No tumors observed. | - Lgr5-Cre;Smad4^fl/fl^;PTEN^fl/fl^:   - Invasive IGC: Submucosal/muscularis propria infiltration (40% mice by 90 days).  - Intestinal/colon models: More aggressive invasion (complete penetrance). | - No mention of inflammatory infiltrates (e.g., macrophages, neutrophils) or cytokines. - Focus was on cell-autonomous mutations (Smad4/PTEN) without inflammatory induction. | - Mechanistic studies: Stem cell-driven tumorigenesis, SMAD4/PTEN synergy. - Therapeutic testing: Targeting LGR5^+^ cancer stem cells or PI3K/Akt pathways. - Human IGC modeling: Validates TCGA findings (SMAD4/PTEN loss in intestinal subtype). |
| Ref. (104) | - Primary strain: C57BL/6N (wild-type, immunocompetent). - Other strains used:  1. Immunodeficient: Rag2-Il2rg double-knockout (R2G2) mice (deficient in T, B, and NK cells). 2. Cre-restricted models:   - CK8-CreERT2; LSL-Cas9 (epithelial-specific).  - Atp4b-Cre; LSL-Cas9 (parietal cell-specific).   1. Nude mice: For experimental metastasis assays. | - Method: Electroporation-based genetically engineered mouse models (EPO-GEMMs). - Procedure:  1. Surgical electroporation: Plasmids encoding oncogenes (e.g., MYC) and CRISPR-Cas9 targeting tumor suppressors (e.g., Trp53, Apc, Cdh1, Msh2) are injected into the gastric epithelium of the corpus region, followed by electric pulses to facilitate uptake. 2. Genetic configurations:   - CIN subtype: MYC + Trp53 knockout.  - GS subtype: MYC + Apc or Cdh1 knockout.  - MSI subtype: MYC + Trp53 + Msh2 knockout.   - Principle: Somatic introduction of genetic lesions mimics human gastric cancer subtypes, enabling autochthonous tumor formation and metastasis. | - Pre-surgery: Fasted for 2 hours before the procedure. - Post-surgery: Fed with DietGel 76A until pre-surgery weight was regained (5–8 days). - Standard conditions: Ad libitum access to food/water, 12-h light-dark cycle, specific-pathogen-free housing. | - Tumor monitoring:   Palpation and ultrasound imaging (detects tumors ≥3 mm).   - Histopathology: H&E staining for tumor grading and precursor lesions.   Immunohistochemistry (IHC): Markers like E-cadherin, Ki67, CK8, MSH2.   - Molecular analysis:   - RNA-seq for transcriptional profiling.  - Whole-exome sequencing (WES) for mutational load.  - Sparse whole-genome sequencing for copy-number alterations (CNAs).   - Metastasis assessment: Gross examination and histology of liver, lungs, peritoneum, ovaries. - Immune profiling: Flow cytometry (CD45, CD3, CD8, CD11c) and CIBERSORT for immune infiltrates. | - Advantages:   - Flexibility: Rapid modeling of diverse genotypes (e.g., CIN, GS, MSI).  - Metastasis: Recapitulates human-like metastatic patterns (rare in traditional GEMMs).  - Host adaptability: Compatible with immunocompetent/deficient backgrounds.  - Speed: Tumors develop in weeks (e.g., median survival: 45 days for MYC-p53).   - Disadvantages:   - Technical complexity: Requires survival surgery and electroporation expertise.  - Intratumoral heterogeneity: Potential due to pooled plasmid delivery.  - Limited cell-of-origin control: Tumors may arise from mixed epithelial populations. | - All models progress through:  1. Precursor lesions:   - 24–72h post-EPO: Edema, neutrophil influx (local inflammation).  - Days 5–8: Mucinous metaplasia, hyperplasia (observed in MYC-p53 and MYC-Apc).   1. Early tumors (3–4 weeks):   Well-differentiated adenocarcinoma (MYC-p53: intestinal phenotype).   1. Late-stage tumors (6–8 weeks):   Poorly differentiated, diffuse carcinoma (MYC-p53 loses E-cadherin; MYC-Cdh1: undifferentiated).   - Specific models:   - MYC-p53: Metastases to liver (56%), peritoneum (67%), lungs (67%) by 45 days.  - MYC-Apc: Liver metastases (88%) by 40 days.  - MYC-Msh2: Reduced metastasis (30% liver, 60% peritoneum) by 53 days. | - Site of electroporation: Corpus region (fundus/antrum junction). - Models:   MYC-p53, MYC-Apc, MYC-Cdh1, and MYC-Msh2 tumors all originate in the corpus.   - Metastases: Distant organs (liver, lungs, peritoneum, ovaries). | All models are invasive and metastatic:   - Local invasion into submucosa/muscle layers (H&E-confirmed). - Metastasis patterns:   - MYC-Apc: Preferential liver metastasis (88%).  - MYC-p53: High peritoneal and lung metastasis.  - MYC-Msh2: Lower metastatic burden (immune-surveilled). | - Acute inflammation post-EPO:   Neutrophils: Dominant in early phases (24–192h).   - Chronic inflammation in tumors:   - MSI tumors: CD45^+^ leukocytes, CD3^+^ T cells (mostly CD8^+^).  - MSS tumors: Fewer immune infiltrates.   - Key immune cells:   - NK cells: Suppress metastasis (depletion increases liver metastases).  - Macrophages/dendritic cells: Present but not quantified. | - Study genotype-phenotype relationships in gastric cancer. - Investigate metastasis organotropism (e.g., liver vs. peritoneal spread). - Evaluate immune checkpoint inhibitors (e.g., anti-CTLA-4 response in MSI tumors). - Test targeted therapies (e.g., WNT or p53 pathway modulators). - Role of NK/T cells in metastasis suppression. - Tumor-immune microenvironment dynamics. |
| Ref. (105) | - Primary Strain: H^+^/K^+^ ATPase-Cre transgenic mice (stomach-specific Cre expression in parietal cells). - Genetic Modifications:   - Conditional knockout of LKB1 (exons 3–6 flanked by loxP).  - Conditional knockout of PTEN (exon 5 flanked by loxP).   - Resulting Model:   - H+/K+ ATPase-Cre; LKB1^L/L^; PTEN^L/L^  (compound knockout).  - Control groups: H^+^/K^+^ ATPase-Cre; LKB1^L/L^, H^+^/K^+^ ATPase-Cre; PTEN^L/L^, and wild-type mice. | - Method:   - Crossed H^+^/K^+^ ATPase-Cre mice with LKB1^L/L^  and PTEN^L/L^ mice to achieve stomach-specific double knockout.  - Cre recombinase excises loxP-flanked exons in parietal cells, ablating LKB1 and PTEN.   - Principle:   - LKB1 loss: Disrupts AMPK/mTOR pathway, cell polarity, and E-cadherin regulation.  - PTEN loss: Activates PI3K/AKT/mTOR pathway, promoting proliferation and survival.  - Combined loss creates a pro-tumorigenic microenvironment (proliferation, angiogenesis, EMT). | - Housing: Specific pathogen-free (SPF) conditions. - Facility: Animal center of National Sun Yat-Sen University. - Diet: not specified in the article. | - Genotyping: PCR of tail DNA for Cre, LKB1, and PTEN alleles. - Imaging:   MRI (3T scanner) to assess stomach enlargement/wall thickness.   - Histopathology:   - H&E staining for hyperplasia/adenocarcinoma.  - IHC/IF: Ki67 (proliferation), TGF-β1/IL-6 (inflammation), EMT markers (α-SMA, vimentin, E-cadherin), stemness markers (LGR5, CD44, CD133).   - Clinical Tests:   - Fecal occult blood (Hemoccult kit).  - Blood chemistry (RBC/WBC counts, lipidemia).   - Survival/Morbidity: Body weight tracking, Kaplan-Meier analysis. | - Advantages:   - Recapitulates human intestinal-type gastric adenocarcinoma.  - Stomach-specific knockout avoids systemic effects.  - Clear timeline of progression (hyperplasia → adenocarcinoma).  - Useful for studying tumor stroma, angiogenesis, and metastasis.   - Disadvantages:   - High lethality by 30 weeks (limits long-term studies).  - Anemia/complications (e.g., bleeding, chylomicronemia) may confound results.  - Parietal cell-specific Cre may not model all gastric cancer subtypes. | H^+^/K^+^ ATPase-Cre; LKB1^L/L^; PTEN^L/L^  Mice:   - 15 weeks: Normal histology (early stage). - 20 weeks: Gastric hyperplasia (preneoplastic). - 25 weeks:   - Hyperplastic polyps.  - Submucosal hemorrhage.  - 80% fecal occult blood positivity.   - 30 weeks:   - Invasive adenocarcinoma (intestinal type).  - Duodenal invasion (68% of mice).   - 40 weeks: Mortality due to cancer progression. | - H^+^/K^+^ ATPase-Cre; LKB1^L/L^; PTEN^L/L^ mice:   - Corpus: Primary site (parietal cell origin).  - Antrum: Secondary involvement (homeostasis disruption).  - Duodenum: Invasion in advanced stages (68% of mice).   - Control mice (LKB1^L/L^   or PTEN^L/L^ alone): No significant lesions. | - H^+^/K^+^ ATPase-Cre; LKB1^L/L^; PTEN^L/L^ mice:   - Local invasion: Duodenum.  - Metastasis: Not explicitly stated (but liver ischemia/necrosis noted at autopsy).   - Other models: No invasion/metastasis reported. | - H^+^/K^+^ ATPase-Cre; LKB1^L/L^; PTEN^L/L^ mice:  1. Cytokines: Elevated TGF-β1 and IL-6. 2. Immune cells:   - Infiltration of inflammatory cells (unspecified type, likely lymphocytes/macrophages due to IL-6/TGF-β context).  - Specific immune cell markers (e.g., CD68 for macrophages) were not analyzed.   - Control mice: No significant inflammation. | - Study gastric carcinogenesis (LKB1/PTEN pathways). - Evaluate tumor microenvironment (angiogenesis, stroma). - Test targeted therapies (e.g., PI3K/mTOR inhibitors). - Investigate EMT and metastasis mechanisms. - Model intestinal-type gastric cancer (Alcian blue^+^/CK7^+^/CK20^+^). |

**References**

1. Watanabe T, Tada M, Nagai H, Sasaki S, Nakao M. Helicobacter pylori infection induces gastric cancer in mongolian gerbils. Gastroenterology. 1998;115(3):642-8.

2. Wang TC, Goldenring JR, Dangler C, Ito S, Mueller A, Jeon WK, et al. Mice lacking secretory phospholipase A2 show altered apoptosis and differentiation with Helicobacter felis infection. Gastroenterology. 1998;114(4):675-89.

3. Fox JG, Sheppard BJ, Dangler CA, Whary MT, Ihrig M, Wang TC. Germ-line p53-targeted disruption inhibits helicobacter-induced premalignant lesions and invasive gastric carcinoma through down-regulation of Th1 proinflammatory responses. Cancer Research. 2002;62(3):696-702.

4. Shi Y, Liu X-F, Zhuang Y, Zhang J-Y, Liu T, Yin Z, et al. Helicobacter pylori-induced Th17 responses modulate Th1 cell responses, benefit bacterial growth, and contribute to pathology in mice. J Immunol. 2010;184(9):5121-9.

5. Lee JY, Kim N, Choi YJ, Nam RH, Choi YJ, Kwon YH, et al. Histologic Findings and Inflammatory Reactions After Long-term Colonization of Helicobacter felis in C57BL/6 Mice. J Cancer Prev. 2014;19(3):224-30.

6. Sakagami T, Dixon M, O'Rourke J, Howlett R, Alderuccio F, Vella J, et al. Atrophic gastric changes in both Helicobacter felis and Helicobacter pylori infected mice are host dependent and separate from antral gastritis. Gut. 1996;39(5):639-48.

7. Berg DJ, Lynch NA, Lynch RG, Lauricella DM. Rapid development of severe hyperplastic gastritis with gastric epithelial dedifferentiation in Helicobacter felis-infected IL-10(-/-) mice. Am J Pathol. 1998;152(5):1377-86.

8. Burkitt MD, Williams JM, Duckworth CA, O'Hara A, Hanedi A, Varro A, et al. Signaling mediated by the NF-κB sub-units NF-κB1, NF-κB2 and c-Rel differentially regulate Helicobacter felis-induced gastric carcinogenesis in C57BL/6 mice. Oncogene. 2013;32(50):5563-73.

9. Druffner SR, Venkateshwaraprabu S, Khadka S, Duncan BC, Morris MT, Sen-Kilic E, et al. Comparison of gastric inflammation and metaplasia induced by Helicobacter pylori or Helicobacter felis colonization in mice. Microbiol Spectr. 2024;12(6):e0001524.

10. Chen J, Zhu C, Wang C, Zhang X, Ni J, Czajkowsky DM, et al. Discovery and genetic characterization of intestinal metaplasia in the Helicobacter felis-infected mouse model of gastric cancer. Acta Biochim Biophys Sin (Shanghai). 2019;51(2):219-22.

11. Lee A, O'Rourke J, De Ungria MC, Robertson B, Daskalopoulos G, Dixon MF. A standardized mouse model of Helicobacter pylori infection: introducing the Sydney strain. Gastroenterology. 1997;112(4):1386-97.

12. Saito T, Inokuchi K, Takayama S, Sugimura T. Sequential morphological changes in N-methyl-N'-nitro-N-nitrosoguanidine carcinogenesis in the glandular stomach of rats. J Natl Cancer Inst. 1970;44(4):769-83.

13. Schoental R. Carcinogenic activity of N-methyl-N-nitroso-N'-nitroguanidine. Nature. 1966;209(5024):726-7.

14. Tatematsu M, Yamamoto M, Shimizu N, Yoshikawa A, Fukami H, Kaminishi M, et al. Induction of glandular stomach cancers in Helicobacter pylori-sensitive Mongolian gerbils treated with N-methyl-N-nitrosourea and N-methyl-N'-nitro-N-nitrosoguanidine in drinking water. Jpn J Cancer Res. 1998;89(2).

15. Tatematsu M, Ogawa K, Hoshiya T, Shichino Y, Kato T, Imaida K, et al. Induction of adenocarcinomas in the glandular stomach of BALB/c mice treated with N-methyl-N-nitrosourea. Jpn J Cancer Res. 1992;83(9):915-8.

16. Yamachika T, Nakanishi H, Inada K, Tsukamoto T, Shimizu N, Kobayashi K, et al. N-methyl-N-nitrosourea concentration-dependent, rather than total intake-dependent, induction of adenocarcinomas in the glandular stomach of BALB/c mice. Jpn J Cancer Res. 1998;89(4):385-91.

17. Yamamoto M, Furihata C, Ogiu T, Tsukamoto T, Inada Ki, Hirano K, et al. Independent variation in susceptibilities of six different mouse strains to induction of pepsinogen-altered pyloric glands and gastric tumor intestinalization by N-methyl-N-nitrosourea. Cancer Letters. 2002;179(2):121-32.

18. Li K, Wang A, Liu H, Li B. Protocol for chemically induced murine gastric tumor model. STAR Protoc. 2021;2(4):100814.

19. Gaddy JA, Radin JN, Loh JT, Zhang F, Washington MK, Peek RM, et al. High dietary salt intake exacerbates Helicobacter pylori-induced gastric carcinogenesis. Infection and Immunity. 2013;81(6):2258-67.

20. Bergin IL, Sheppard BJ, Fox JG. Helicobacter pylori infection and high dietary salt independently induce atrophic gastritis and intestinal metaplasia in commercially available outbred Mongolian gerbils. Digestive Diseases and Sciences. 2003;48(3):475-85.

21. Fox JG, Dangler CA, Taylor NS, King A, Koh TJ, Wang TC. High-salt diet induces gastric epithelial hyperplasia and parietal cell loss, and enhances Helicobacter pylori colonization in C57BL/6 mice. Cancer Research. 1999;59(19):4823-8.

22. Kodama M, Kodama T, Suzuki H, Kondo K. Effect of rice and salty rice diets on the structure of mouse stomach. Nutr Cancer. 1984;6(3):135-47.

23. Tatematsu M, Takahashi M, Fukushima S, Hananouchi M, Shirai T. Effects in rats of sodium chloride on experimental gastric cancers induced by N-methyl-N-nitro-N-nitrosoguanidine or 4-nitroquinoline-1-oxide. J Natl Cancer Inst. 1975;55(1):101-6.

24. Takahashi M, Kokubo T, Furukawa F, Kurokawa Y, Tatematsu M, Hayashi Y. Effect of high salt diet on rat gastric carcinogenesis induced by N-methyl-N'-nitro-N-nitrosoguanidine. Gan. 1983;74(1):28-34.

25. Takahashi M, Nishikawa A, Furukawa F, Enami T, Hasegawa T, Hayashi Y. Dose-dependent promoting effects of sodium chloride (NaCl) on rat glandular stomach carcinogenesis initiated with N-methyl-N'-nitro-N-nitrosoguanidine. Carcinogenesis. 1994;15(7):1429-32.

26. Rogers AB, Taylor NS, Whary MT, Stefanich ED, Wang TC, Fox JG. Helicobacter pylori but not high salt induces gastric intraepithelial neoplasia in B6129 mice. Cancer Research. 2005;65(23):10709-15.

27. Yin J, Yi J, Yang C, Xu B, Lin J, Hu H, et al. Chronic atrophic gastritis and intestinal metaplasia induced by high-salt and N-methyl-N'-nitro-N-nitrosoguanidine intake in rats. Exp Ther Med. 2021;21(4):315.

28. Nakamura Y, Sakagami T, Yamamoto N, Yokota Y, Koizuka H, Hori K, et al. Helicobacter pylori does not promote N-methyl-N-nitrosourea-induced gastric carcinogenesis in SPF C57BL/6 mice. Jpn J Cancer Res. 2002;93(2):111-6.

29. Toyoda T, Tsukamoto T, Yamamoto M, Ban H, Saito N, Takasu S, et al. Gene expression analysis of a Helicobacter pylori-infected and high-salt diet-treated mouse gastric tumor model: identification of CD177 as a novel prognostic factor in patients with gastric cancer. BMC Gastroenterology. 2013;13:122.

30. Chu F, Li Y, Meng X, Li Y, Li T, Zhai M, et al. Gut Microbial Dysbiosis and Changes in Fecal Metabolic Phenotype in Precancerous Lesions of Gastric Cancer Induced With N-Methyl-N'-Nitro-N-Nitrosoguanidine, Sodium Salicylate, Ranitidine, and Irregular Diet. Frontiers In Physiology. 2021;12:733979.

31. Alizadeh AM, Afrouzan H, Dinparast-Djadid N, Sawaya ACHF, Azizian S, Hemmati HR, et al. Chemoprotection of MNNG-initiated gastric cancer in rats using Iranian propolis. Arch Iran Med. 2015;18(1):18-23.

32. Morris MT, Duncan BC, Piazuelo MB, Olfert IM, Xu X, Hussain S, et al. Chronic Cigarette Smoke Exposure Masks Pathological Features of Helicobacter pylori Infection While Promoting Tumor Initiation. Cancer Prev Res (Phila). 2025;18(5):271-81.

33. Goldenring JR, Ray GS, Coffey RJ, Meunier PC, Haley PJ, Barnes TB, et al. Reversible drug-induced oxyntic atrophy in rats. Gastroenterology. 2000;118(6):1080-93.

34. Nam KT, Lee H-J, Sousa JF, Weis VG, O'Neal RL, Finke PE, et al. Mature chief cells are cryptic progenitors for metaplasia in the stomach. Gastroenterology. 2010;139(6).

35. Saenz JB, Burclaff J, Mills JC. Modeling Murine Gastric Metaplasia Through Tamoxifen-Induced Acute Parietal Cell Loss. Methods Mol Biol. 2016;1422:329-39.

36. Zavros Y, Eaton KA, Kang W, Rathinavelu S, Katukuri V, Kao JY, et al. Chronic gastritis in the hypochlorhydric gastrin-deficient mouse progresses to adenocarcinoma. Oncogene. 2005;24(14):2354-66.

37. Nomura S, Yamaguchi H, Ogawa M, Wang TC, Lee JR, Goldenring JR. Alterations in gastric mucosal lineages induced by acute oxyntic atrophy in wild-type and gastrin-deficient mice. Am J Physiol Gastrointest Liver Physiol. 2005;288(2):G362-G75.

38. Tomita H, Takaishi S, Menheniott TR, Yang X, Shibata W, Jin G, et al. Inhibition of gastric carcinogenesis by the hormone gastrin is mediated by suppression of TFF1 epigenetic silencing. Gastroenterology. 2011;140(3):879-91.

39. Wang TC, Dangler CA, Chen D, Goldenring JR, Koh T, Raychowdhury R, et al. Synergistic interaction between hypergastrinemia and Helicobacter infection in a mouse model of gastric cancer. Gastroenterology. 2000;118(1):36-47.

40. Fox JG, Wang TC, Rogers AB, Poutahidis T, Ge Z, Taylor N, et al. Host and microbial constituents influence Helicobacter pylori-induced cancer in a murine model of hypergastrinemia. Gastroenterology. 2003;124(7):1879-90.

41. Lertpiriyapong K, Whary MT, Muthupalani S, Lofgren JL, Gamazon ER, Feng Y, et al. Gastric colonisation with a restricted commensal microbiota replicates the promotion of neoplastic lesions by diverse intestinal microbiota in the Helicobacter pylori INS-GAS mouse model of gastric carcinogenesis. Gut. 2014;63(1):54-63.

42. Tu S, Bhagat G, Cui G, Takaishi S, Kurt-Jones EA, Rickman B, et al. Overexpression of interleukin-1beta induces gastric inflammation and cancer and mobilizes myeloid-derived suppressor cells in mice. Cancer Cell. 2008;14(5):408-19.

43. Syu L-J, El-Zaatari M, Eaton KA, Liu Z, Tetarbe M, Keeley TM, et al. Transgenic expression of interferon-γ in mouse stomach leads to inflammation, metaplasia, and dysplasia. Am J Pathol. 2012;181(6):2114-25.

44. Oshima H, Oshima M, Inaba K, Taketo MM. Hyperplastic gastric tumors induced by activated macrophages in COX-2/mPGES-1 transgenic mice. EMBO J. 2004;23(7):1669-78.

45. Oshima H, Matsunaga A, Fujimura T, Tsukamoto T, Taketo MM, Oshima M. Carcinogenesis in mouse stomach by simultaneous activation of the Wnt signaling and prostaglandin E2 pathway. Gastroenterology. 2006;131(4):1086-95.

46. Leung WK, Wu K-c, Wong CYP, Cheng ASL, Ching AKK, Chan AWH, et al. Transgenic cyclooxygenase-2 expression and high salt enhanced susceptibility to chemical-induced gastric cancer development in mice. Carcinogenesis. 2008;29(8):1648-54.

47. Sharp R, Babyatsky MW, Takagi H, Tågerud S, Wang TC, Bockman DE, et al. Transforming growth factor alpha disrupts the normal program of cellular differentiation in the gastric mucosa of transgenic mice. Development. 1995;121(1):149-61.

48. Goldenring JR, Poulsom R, Ray GS, Wright N, Meise KS, Coffey RJ. Expression of trefoil peptides in the gastric mucosa of transgenic mice overexpressing transforming growth factor-alpha. Growth Factors. 1996;13(1-2):111-9.

49. Nomura S, Settle SH, Leys CM, Means AL, Peek RM, Leach SD, et al. Evidence for repatterning of the gastric fundic epithelium associated with Ménétrier's disease and TGFalpha overexpression. Gastroenterology. 2005;128(5):1292-305.

50. Asfaha S, Dubeykovskiy AN, Tomita H, Yang X, Stokes S, Shibata W, et al. Mice that express human interleukin-8 have increased mobilization of immature myeloid cells, which exacerbates inflammation and accelerates colon carcinogenesis. Gastroenterology. 2013;144(1):155-66.

51. Shibata W, Ariyama H, Westphalen CB, Worthley DL, Muthupalani S, Asfaha S, et al. Stromal cell-derived factor-1 overexpression induces gastric dysplasia through expansion of stromal myofibroblasts and epithelial progenitors. Gut. 2013;62(2):192-200.

52. Brembeck FH, Schreiber FS, Deramaudt TB, Craig L, Rhoades B, Swain G, et al. The mutant K-ras oncogene causes pancreatic periductal lymphocytic infiltration and gastric mucous neck cell hyperplasia in transgenic mice. Cancer Research. 2003;63(9):2005-9.

53. Okumura T, Ericksen RE, Takaishi S, Wang SSW, Dubeykovskiy Z, Shibata W, et al. K-ras mutation targeted to gastric tissue progenitor cells results in chronic inflammation, an altered microenvironment, and progression to intraepithelial neoplasia. Cancer Research. 2010;70(21):8435-45.

54. Ray KC, Bell KM, Yan J, Gu G, Chung CH, Washington MK, et al. Epithelial tissues have varying degrees of susceptibility to Kras(G12D)-initiated tumorigenesis in a mouse model. PloS One. 2011;6(2):e16786.

55. Matkar SS, Durham A, Brice A, Wang TC, Rustgi AK, Hua X. Systemic activation of K-ras rapidly induces gastric hyperplasia and metaplasia in mice. American Journal of Cancer Research. 2011;1(4):432-45.

56. Hayakawa Y, Ariyama H, Stancikova J, Sakitani K, Asfaha S, Renz BW, et al. Mist1 Expressing Gastric Stem Cells Maintain the Normal and Neoplastic Gastric Epithelium and Are Supported by a Perivascular Stem Cell Niche. Cancer Cell. 2015;28(6):800-14.

57. Till JE, Yoon C, Kim B-J, Roby K, Addai P, Jonokuchi E, et al. Oncogenic KRAS and p53 Loss Drive Gastric Tumorigenesis in Mice That Can Be Attenuated by E-Cadherin Expression. Cancer Research. 2017;77(19):5349-59.

58. Kinoshita H, Hayakawa Y, Konishi M, Hata M, Tsuboi M, Hayata Y, et al. Three types of metaplasia model through Kras activation, Pten deletion, or Cdh1 deletion in the gastric epithelium. J Pathol. 2019;247(1):35-47.

59. Thiem S, Eissmann MF, Elzer J, Jonas A, Putoczki TL, Poh A, et al. Stomach-Specific Activation of Oncogenic KRAS and STAT3-Dependent Inflammation Cooperatively Promote Gastric Tumorigenesis in a Preclinical Model. Cancer Research. 2016;76(8):2277-87.

60. Matsuo J, Douchi D, Myint K, Mon NN, Yamamura A, Kohu K, et al. Iqgap3-Ras axis drives stem cell proliferation in the stomach corpus during homoeostasis and repair. Gut. 2021;70(10):1833-46.

61. Choi E, Hendley AM, Bailey JM, Leach SD, Goldenring JR. Expression of Activated Ras in Gastric Chief Cells of Mice Leads to the Full Spectrum of Metaplastic Lineage Transitions. Gastroenterology. 2016;150(4).

62. O'Brien VP, Koehne AL, Dubrulle J, Rodriguez AE, Leverich CK, Kong VP, et al. Sustained Helicobacter pylori infection accelerates gastric dysplasia in a mouse model. Life Sci Alliance. 2021;4(2).

63. Huang X-B, Huang Q, Jiang M-C, Zhong Q, Zheng H-L, Wang J-B, et al. KLHL21 suppresses gastric tumourigenesis via maintaining STAT3 signalling equilibrium in stomach homoeostasis. Gut. 2024;73(11):1785-98.

64. Tu R, Zheng H, Zheng B, Zhong Q, Qian J, Wu F, et al. <em>Tff2</em> marks gastric corpus progenitors that give rise to pyloric metaplasia/SPEM following injury. bioRxiv. 2025:2025.04.09.647847.

65. Ito K, Chuang LSH, Ito T, Chang TL, Fukamachi H, Salto-Tellez M, et al. Loss of Runx3 is a key event in inducing precancerous state of the stomach. Gastroenterology. 2011;140(5).

66. Douchi D, Yamamura A, Matsuo J, Lee J-W, Nuttonmanit N, Melissa Lim YH, et al. A Point Mutation R122C in RUNX3 Promotes the Expansion of Isthmus Stem Cells and Inhibits Their Differentiation in the Stomach. Cell Mol Gastroenterol Hepatol. 2022;13(5):1317-45.

67. Kuzushita N, Rogers AB, Monti NA, Whary MT, Park MJ, Aswad BI, et al. p27kip1 deficiency confers susceptibility to gastric carcinogenesis in Helicobacter pylori-infected mice. Gastroenterology. 2005;129(5):1544-56.

68. Costa L, Corre S, Michel V, Le Luel K, Fernandes J, Ziveri J, et al. USF1 defect drives p53 degradation during Helicobacter pylori infection and accelerates gastric carcinogenesis. Gut. 2020;69(9):1582-91.

69. Hayashi D, Tamura A, Tanaka H, Yamazaki Y, Watanabe S, Suzuki K, et al. Deficiency of claudin-18 causes paracellular H+ leakage, up-regulation of interleukin-1β, and atrophic gastritis in mice. Gastroenterology. 2012;142(2):292-304.

70. Hagen SJ, Ang L-H, Zheng Y, Karahan SN, Wu J, Wang YE, et al. Loss of Tight Junction Protein Claudin 18 Promotes Progressive Neoplasia Development in Mouse Stomach. Gastroenterology. 2018;155(6):1852-67.

71. Suzuki K, Sentani K, Tanaka H, Yano T, Suzuki K, Oshima M, et al. Deficiency of Stomach-Type Claudin-18 in Mice Induces Gastric Tumor Formation Independent of H pylori Infection. Cell Mol Gastroenterol Hepatol. 2019;8(1):119-42.

72. Nam KT, Lee H-J, Mok H, Romero-Gallo J, Crowe JE, Peek RM, et al. Amphiregulin-deficient mice develop spasmolytic polypeptide expressing metaplasia and intestinal metaplasia. Gastroenterology. 2009;136(4):1288-96.

73. Liu X, Li T, Ma Z, Riederer B, Yuan D, Zhu J, et al. SLC26A9 deficiency causes gastric intraepithelial neoplasia in mice and aggressive gastric cancer in humans. Cell Oncol (Dordr). 2022;45(3):381-98.

74. Zeng X, Yang M, Ye T, Feng J, Xu X, Yang H, et al. Mitochondrial GRIM-19 loss in parietal cells promotes spasmolytic polypeptide-expressing metaplasia through NLR family pyrin domain-containing 3 (NLRP3)-mediated IL-33 activation via a reactive oxygen species (ROS) -NRF2- Heme oxygenase-1(HO-1)-NF-кB axis. Free Radic Biol Med. 2023;202:46-61.

75. Keeley TM, Samuelson LC. Cytodifferentiation of the postnatal mouse stomach in normal and Huntingtin-interacting protein 1-related-deficient mice. Am J Physiol Gastrointest Liver Physiol. 2010;299(6):G1241-G51.

76. Arai J, Hayakawa Y, Tateno H, Murakami K, Hayashi T, Hata M, et al. Impaired Glycosylation of Gastric Mucins Drives Gastric Tumorigenesis and Serves as a Novel Therapeutic Target. Gastroenterology. 2024;167(3).

77. Soutto M, Chen Z, Bhat AA, Wang L, Zhu S, Gomaa A, et al. Activation of STAT3 signaling is mediated by TFF1 silencing in gastric neoplasia. Nature Communications. 2019;10(1):3039.

78. Katsha A, Soutto M, Sehdev V, Peng D, Washington MK, Piazuelo MB, et al. Aurora kinase A promotes inflammation and tumorigenesis in mice and human gastric neoplasia. Gastroenterology. 2013;145(6).

79. Soutto M, Belkhiri A, Piazuelo MB, Schneider BG, Peng D, Jiang A, et al. Loss of TFF1 is associated with activation of NF-κB-mediated inflammation and gastric neoplasia in mice and humans. J Clin Invest. 2011;121(5):1753-67.

80. Muthupalani S, Ge Z, Joy J, Feng Y, Dobey C, Cho H-Y, et al. Muc5ac null mice are predisposed to spontaneous gastric antro-pyloric hyperplasia and adenomas coupled with attenuated H. pylori-induced corpus mucous metaplasia. Lab Invest. 2019;99(12):1887-905.

81. Kawakubo M, Komura H, Goso Y, Okumura M, Sato Y, Fujii C, et al. Analysis of A4gnt Knockout Mice Reveals an Essential Role for Gastric Sulfomucins in Preventing Gastritis Cystica Profunda. J Histochem Cytochem. 2019;67(10):759-70.

82. Menheniott TR, O'Connor L, Chionh YT, Däbritz J, Scurr M, Rollo BN, et al. Loss of gastrokine-2 drives premalignant gastric inflammation and tumor progression. J Clin Invest. 2016;126(4):1383-400.

83. Ernst M, Najdovska M, Grail D, Lundgren-May T, Buchert M, Tye H, et al. STAT3 and STAT1 mediate IL-11-dependent and inflammation-associated gastric tumorigenesis in gp130 receptor mutant mice. J Clin Invest. 2008;118(5):1727-38.

84. Tye H, Kennedy CL, Najdovska M, McLeod L, McCormack W, Hughes N, et al. STAT3-driven upregulation of TLR2 promotes gastric tumorigenesis independent of tumor inflammation. Cancer Cell. 2012;22(4):466-78.

85. Zuo X, Deguchi Y, Xu W, Liu Y, Li HS, Wei D, et al. PPARD and Interferon Gamma Promote Transformation of Gastric Progenitor Cells and Tumorigenesis in Mice. Gastroenterology. 2019;157(1):163-78.

86. Judd LM, Alderman BM, Howlett M, Shulkes A, Dow C, Moverley J, et al. Gastric cancer development in mice lacking the SHP2 binding site on the IL-6 family co-receptor gp130. Gastroenterology. 2004;126(1):196-207.

87. Nam KT, O'Neal R, Lee YS, Lee YC, Coffey RJ, Goldenring JR. Gastric tumor development in Smad3-deficient mice initiates from forestomach/glandular transition zone along the lesser curvature. Lab Invest. 2012;92(6):883-95.

88. Low JT, Christie M, Ernst M, Dumoutier L, Preaudet A, Ni Y, et al. Loss of NFKB1 Results in Expression of Tumor Necrosis Factor and Activation of Signal Transducer and Activator of Transcription 1 to Promote Gastric Tumorigenesis in Mice. Gastroenterology. 2020;159(4).

89. Buzzelli JN, O'Connor L, Scurr M, Chung Nien Chin S, Catubig A, Ng GZ, et al. Overexpression of IL-11 promotes premalignant gastric epithelial hyperplasia in isolation from germline gp130-JAK-STAT driver mutations. Am J Physiol Gastrointest Liver Physiol. 2019;316(2):G251-G62.

90. Banerjee A, Thamphiwatana S, Carmona EM, Rickman B, Doran KS, Obonyo M. Deficiency of the myeloid differentiation primary response molecule MyD88 leads to an early and rapid development of Helicobacter-induced gastric malignancy. Infection and Immunity. 2014;82(1):356-63.

91. Neumeyer V, Vieth M, Gerhard M, Mejías-Luque R. Mutated Rnf43 Aggravates Helicobacter Pylori-Induced Gastric Pathology. Cancers (Basel). 2019;11(3).

92. Neumeyer V, Brutau-Abia A, Allgäuer M, Pfarr N, Weichert W, Falkeis-Veits C, et al. Loss of RNF43 Function Contributes to Gastric Carcinogenesis by Impairing DNA Damage Response. Cell Mol Gastroenterol Hepatol. 2021;11(4):1071-94.

93. Fukuma K, Sakaguchi S, Kuribayashi K, Chen WL, Morishita R, Sekita K, et al. Immunologic and clinical studies on murine experimental autoimmune gastritis induced by neonatal thymectomy. Gastroenterology. 1988;94(2):274-83.

94. Nishiura H, Kido M, Aoki N, Iwamoto S, Maruoka R, Ikeda A, et al. Increased susceptibility to autoimmune gastritis in thymic stromal lymphopoietin receptor-deficient mice. J Immunol. 2012;188(1):190-7.

95. Field J, Alderuccio F, Hertzog P, Toh B-H. GM-CSF-induced autoimmune gastritis in interferon alpha receptor deficient mice. J Autoimmun. 2008;31(3):274-80.

96. Nguyen T-LM, Khurana SS, Bellone CJ, Capoccia BJ, Sagartz JE, Kesman RA, et al. Autoimmune gastritis mediated by CD4+ T cells promotes the development of gastric cancer. Cancer Research. 2013;73(7):2117-26.

97. Liu F, Fu J, Bergstrom K, Shan X, McDaniel JM, McGee S, et al. Core 1-derived mucin-type O-glycosylation protects against spontaneous gastritis and gastric cancer. J Exp Med. 2020;217(1).

98. Alderuccio F, Sentry JW, Marshall ACJ, Biondo M, Toh BH. Animal models of human disease: experimental autoimmune gastritis--a model for autoimmune gastritis and pernicious anemia. Clin Immunol. 2002;102(1):48-58.

99. De Salvo C, Pastorelli L, Petersen CP, Buttò LF, Buela K-A, Omenetti S, et al. Interleukin 33 Triggers Early Eosinophil-Dependent Events Leading to Metaplasia in a Chronic Model of Gastritis-Prone Mice. Gastroenterology. 2021;160(1).

100. Seidlitz T, Chen Y-T, Uhlemann H, Schölch S, Kochall S, Merker SR, et al. Mouse Models of Human Gastric Cancer Subtypes With Stomach-Specific CreERT2-Mediated Pathway Alterations. Gastroenterology. 2019;157(6).

101. Douchi D, Yamamura A, Matsuo J, Melissa Lim YH, Nuttonmanit N, Shimura M, et al. Induction of Gastric Cancer by Successive Oncogenic Activation in the Corpus. Gastroenterology. 2021;161(6).

102. Fatehullah A, Terakado Y, Sagiraju S, Tan TL, Sheng T, Tan SH, et al. A tumour-resident Lgr5+ stem-cell-like pool drives the establishment and progression of advanced gastric cancers. Nat Cell Biol. 2021;23(12):1299-313.

103. Li X-B, Yang G, Zhu L, Tang Y-L, Zhang C, Ju Z, et al. Gastric Lgr5(+) stem cells are the cellular origin of invasive intestinal-type gastric cancer in mice. Cell Research. 2016;26(7):838-49.

104. Leibold J, Tsanov KM, Amor C, Ho Y-J, Sánchez-Rivera FJ, Feucht J, et al. Somatic mouse models of gastric cancer reveal genotype-specific features of metastatic disease. Nat Cancer. 2024;5(2):315-29.

105. Fang K-T, Hung H, Lau NYS, Chi J-H, Wu D-C, Cheng K-H. Development of a Genetically Engineered Mouse Model Recapitulating LKB1 and PTEN Deficiency in Gastric Cancer Pathogenesis. Cancers (Basel). 2023;15(24).
